# GPR19 Coordinates Multiple Molecular Aspects of Stress Responses Associated with the Aging Process

**DOI:** 10.3390/ijms24108499

**Published:** 2023-05-09

**Authors:** Stuart Maudsley, Claudia Schrauwen, İrem Harputluoğlu, Deborah Walter, Hanne Leysen, Patricia McDonald

**Affiliations:** 1 Receptor Biology Lab, University of Antwerp, 2610 Antwerpen, Belgium; 2 Moffitt Cancer Center, Department of Metabolism & Physiology, 12902 Magnolia Drive, Tampa, FL 33612, USA; 3 Lexicon Pharmaceuticals Inc. Research & Development, 2445 Technology Forest, The Woodlands, TX 77381, USA

**Keywords:** GPR19, receptor, aging, stress, damage, DNA, metabolism, mitochondria, longevity, adiposity

## Abstract

G protein-coupled receptors (GPCRs) play a significant role in controlling biological paradigms such as aging and aging-related disease. We have previously identified receptor signaling systems that are specifically associated with controlling molecular pathologies associated with the aging process. Here, we have identified a pseudo-orphan GPCR, G protein-coupled receptor 19 (GPR19), that is sensitive to many molecular aspects of the aging process. Through an in-depth molecular investigation process that involved proteomic, molecular biological, and advanced informatic experimentation, this study found that the functionality of GPR19 is specifically linked to sensory, protective, and remedial signaling systems associated with aging-related pathology. This study suggests that the activity of this receptor may play a role in mitigating the effects of aging-related pathology by promoting protective and remedial signaling systems. GPR19 expression variation demonstrates variability in the molecular activity in this larger process. At low expression levels in HEK293 cells, GPR19 expression regulates signaling paradigms linked with stress responses and metabolic responses to these. At higher expression levels, GPR19 expression co-regulates systems involved in sensing and repairing DNA damage, while at the highest levels of GPR19 expression, a functional link to processes of cellular senescence is seen. In this manner, GPR19 may function as a coordinator of aging-associated metabolic dysfunction, stress response, DNA integrity management, and eventual senescence.

## 1. Introduction

Emerging research has demonstrated that G protein-coupled receptor (GPCR) systems play an important, multidimensional role in the aging process [1,2,3,4,5,6,7,8,9]. GPCRs have long been characterized as controllers of cell-to-cell communication and thus the target of multiple therapeutic systems that control endocrine functionality in health and diseases [10,11,12,13,14]. In addition to the classical mode of G protein-dependent signaling an expanding repertoire of additional GPCR signaling adaptors, e.g., β-arrestin, has been gaining traction as an attractive new paradigm for effective novel drug development [1,6,8,13,15,16,17]. Along with the β-arrestin signaling capacity, the novel GPCR adaptor, GIT2 (ADP-ribosylation factor GTPase-activating protein 2), has shown promise for its association with stress-response functions of cells [6,18,19]. GIT2 is a central regulator of the aging process and has been shown to control multiple aspects of this process, including energy metabolism, mitochondrial function, circadian rhythm, immune senescence, central nervous system connectivity, and DNA damage management [6,20,21,22,23,24,25]. GIT2 single nucleotide polymorphisms in humans have also been associated with metabolic syndrome (a strong pro-aging condition) [26]. Genomic deletion of GIT2 in murine experimental models induces an accelerated aging phenotype associated with increased rates of cellular senescence, disrupted energy regulation, insulin resistance, and potentiated levels/rates of DNA damage accumulation [22,23,24]. We have previously investigated the capacity for GPCRs to be functionally associated with the GIT2 signaling paradigm. Through tissue expression analysis of genetically modified murine tissues that possess a deficiency of GIT2, we found a strong functional relationship between GIT2 and the human relaxin-3 receptor (RXFP3). In GIT2 deletion (GIT2 knockout, GIT2KO) and haplo-insufficient GIT2 heterozygous mice, a responsive reduction in RXFP3 expression was observed. Hence, in this scenario, it could be proposed that RXFP3 and GIT2 may act hand-in-hand to control and regulate these multiple aspects of the aging process. We have subsequently found that this functional system demonstrates an important novel anti-aging functionality of the RXFP3 receptor system [6,27,28]. Here, we have furthered this research to investigate additional GPCRs potentially associated with the neurometabolic aging paradigm observed in the GIT2KO murine model. In this initial manuscript, through complementary expression analysis of GIT2KO murine, we have identified a further GPCR that demonstrates a potent expression-based relationship to the energy metabolism and DNA damage management features of the GIT2KO aging model. This specific receptor is the current orphan GPR19 class A rhodopsin-like GPCR [29,30,31,32,33]. In this study, we have shown that GPR19 may intersect with aging as well as oncological paradigms through the regulation of novel interactions between proteins associated with DNA damage response (DDR) and energy metabolism and regulation. GPR19 has been proposed to be linked to the activation of mitogen-activated protein kinases (MAPKs) and the G protein-mediated inhibition of cellular levels of cyclic AMP [34]. While the peptide hormone Adropin (also known as the Energy Homeostasis Associated gene (ENHO) product) has been proposed to be the cognate ligand for GPR19 [32], several experiments have failed to confirm this [35]. Therefore, this receptor has since retained its orphan status. Here, we provide functional evidence that supports the molecular role of GPR19 in controlling the intersection between aging-related signaling and oncological activity.

## 2. Results

### 2.1. Coordinated Protein Expression Profiles of GPR19 in Advanced Aging Murine Models

**Figure 1:**
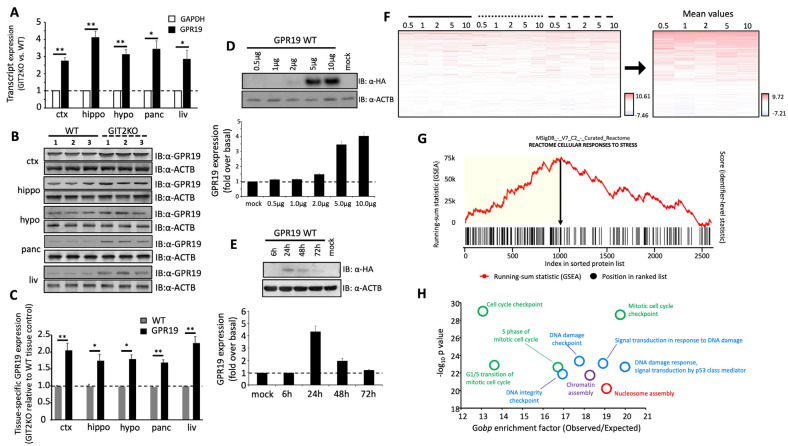
GPR19 expression is altered in an accelerated aging murine model. Using transcriptome profiling, we investigated the expression levels of GPR19 in GIT2KO (GIT2−knockout) mice (*n* = 3): (**A**). GPR19 expression levels were elevated in both central nervous system tissues (cortex (ctx), hippocampus (hippo), and hypothalamus (hypo)), as well as peripheral tissues (pancreas (panc), and liver (liv)) compared to wild-type (WT) littermates (*n* = 3). GAPDH expression was employed as an expression control. (**B**) GPR19 expression alteration results were replicated using Western blotting; beta-actin (ACTB) loading control was used. Hence, GPR19 expression was potentiated in the GIT2KO mice (#1,2,3) compared to control wild-type (WT: #1,2,3) animals (*n* = 3). The significant alterations of GPR19 expression in the advanced aging model GIT2KO are represented in the associated histogram (**C**). Ectopic expression of an HA-epitope tagged human WT GPR19 clone in HEK293 cells was demonstrated using a cDNA transfection level series from 0.5, 1, 2,5, and 10 μg (**D**). Cells were lysed at 24 h post-transfection and HA-tagged GPR19 expression was confirmed with selective Western blot. The optimal time course of expression of the median expression level (i.e., 2 μg) was assessed and found to be optimal at the standard 24 h time period following cDNA transfection (**E**). Differential compartment protein extraction was performed (cytoplasmic = solid line; plasma membrane = dotted line; nucleus/organelles = dashed line) before untargeted proteomic expression analysis (red bar indicates protein expression increase while blue bars indicate protein expression decrease—compared to the calculated proteomic baseline) was made across the stated expression level series (0.5, 1, 2, 5, and 10 μg cDNA). To simplify the data analysis of this complex expression matrix, the mean protein expression values across the different extraction conditions were calculated (**F**). The associated heatmap key indicates the range of log_2_ transformed GPR19: mock expression ratios. These mean data were then subjected to signaling pathway analysis using a Kolmogorov–Smirnoff (KS) test applied to the MSigDB Curated Reactome Database. One of the most prominent pathways populated by the total cellular perturbation response (from mean expression response data in panel (**F**)) to GPR19 expression was the Reactome “Cellular Response to Stress” (**G**). The yellow inset in panel **G** indicates the “leading edge” set of proteins that are responding to the positive KS score for “Cellular Response to Stress.” Extracting this protein set and then analyzing it for Gene Ontology Biological Process (GOBP) enrichment analysis revealed a strong phenotype for DNA-based stress management (**H**). Histogram-based data shown represent the means ± SEM (standard error of the mean). The significance level is indicated in each figure as * *p* ≤ 0.05; ** *p* ≤ 0.01.

Multiple tissues from the central nervous system (cortex, hippocampus, and hypothalamus) and the periphery (pancreas and liver) were collected from male GIT2KO mice for transcript (Figure 1A) and protein expression profiling (Figure 1B,C). We found a strong connection between GIT2 expression status and GPR19 expression. This means that with GIT2 deletion, a responsive, significant elevation of GPR19 transcript (Figure 1A) and protein expression was observed (Figure 1B,C).

### 2.2. Ectopic Expression Induced Human GPR19 Perturbagen Responses in Human Cells

Using an epitope-tagged N-terminal triple hemagglutinin (3x-HA) human GPR19 cDNA clone, a transient expression profile was achievable in HEK293 cells (Figure 1D). Peak expression of HA-tagged human GPR19 was found to occur at 24 h post transient transfection (Figure 1E). Following the application of a cDNA “dose” series of ectopic GPR19 expression (0.5, 1, 2, 5, and 10 μg of cDNA transfected), three distinct protein extractions were made using the Qiagen Qproteome Cell Compartment differential detergent fractionation (DDF) process as described previously [6]. The Qproteome DDF process generates a cytoplasmic compartment extract (solid line in Figure 1F: Supplementary Table S1), a plasma membrane compartment extract (dotted line in Figure 1F: Table S2), and a nucleus/organelle compartment extract (dashed line in Figure 1F: Table S3). Using MAXQUANT LC-MS2-based label-free protein quantification, an in-depth protein expression map (Figure 1F: Table S1) was created for GPR19 perturbagen responses to the applied over-expression of the GPR19 cDNA clones (mimicking the elevation observed in the GIT2KO model: Figure 1A). Each protein identity displayed was significantly different (*p* ≤ 0.05) and differentially expressed compared to the calculated global mean [36]. A mean expression level for all the identified differentially expressed proteins (DEPs) was generated across all three differential compartment extraction protocols (Table S4). To generate a simple functional interpretation of the mean GPR19 DEP perturbagen results, Reactome signaling pathway analysis (applying the Kolmogorov–Smirnoff (K–S) protocol) revealed a strong component of cellular resilience in the global GPR19 data. The GSEA (gene set enrichment analysis) running-sum KS score for the “Cellular Response to Stress” pathway is shown in Figure 1G. The leading-edge DEP set that is responsible for the protein subset that creates the greatest peak of the running sum is shown by a yellow block in the panel in Figure 1G. Gene ontology (GO) term enrichment analysis was then employed to investigate the functional signature of the leading-edge DEP dataset (Figure 1H: Table S5). Using quantitative GO biological process term enrichment, a strong demonstration of a DNA damage management phenotype was observed (“DNA damage response”, “signal transduction by p53 class mediator”, “signal transduction in response to DNA damage”, “DNA damage checkpoint”, “DNA integrity checkpoint”). The next most prominent GO term group concerned cell cycle regulation effects, followed by functional clusters linked to chromatin and nucleosome assembly.

### 2.3. GPR19 Perturbagen Responses Are Reminiscent of a Cancer-Associated Functional Network

**Figure 2:**
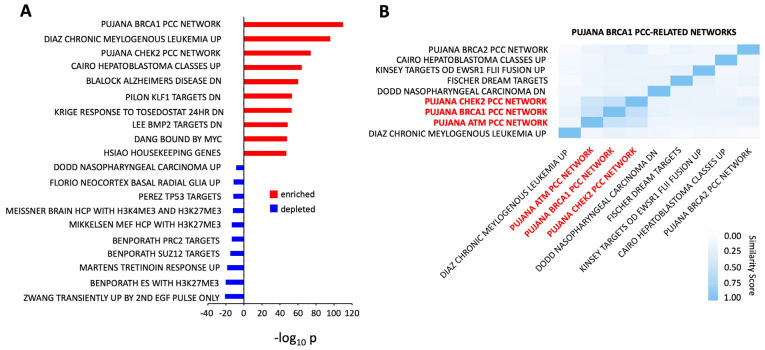
GPR19 molecular signature is associated with DNA and energy management pathways. Using a specific MSigDB C2−Chemical and Genetic and Perturbations (CGP) database that encompasses complex molecular collections of proteins associated with well-characterized specific processes, we found that the GPR19 molecular perturbation signature most closely matched the PUJANA BRCA1 PCC Network. This suggests a strong potential functional relationship between GPR19 and BRCA1-related activity (**A**). Using a similarity clustering process with GeneTrail v3.2 (https://genetrail.bioinf.uni-sb.de/: accessed on 21 February 2023) a strong local MSigDB-CGP clustering between PUJANA BRCA1 PCC NETWORK, PUJANA CHEK2 PCC NETWORK, and the PUJANA ATM PCC NETWORK was found (**B**).

The use of signaling pathway analysis and GO term enrichment has demonstrated that the cellular response to GPR19 perturbation indicates a prominent role for GPR19 in cell stress responses and DNA damage management. GSEA-based interrogation of large DEP datasets using the human-curated Chemical or Genetic Perturbation (CGP) database (http://www.gsea-msigdb.org/gsea/msigdb/human/genesets.jsp?collection=C2: accessed on 21 February 2023) is effective to correlate differential experimental datasets through a simple mathematical comparison [37,38] of differentially expressed genes/proteins. Applying this form of GSEA experimental comparison, we found the highest-scoring comparable dataset to the GPR19 perturbagen mean dataset was the PUJANA_BRCA1_PCC_NETWORK (Figure 2A–Table S6: http://www.gsea-msigdb.org/gsea/msigdb/human/geneset/PUJANA_BRCA1_PCC_NETWORK.html: accessed on 21 February 2023). Genes constituting the BRCA1-PCC network comprise transcripts whose expression is positively correlated (Pearson correlation coefficient, PCC ≥ 0.4) with that of BRCA1 across a compendium of normal tissues [39]. In a study by Pujana et al. (2007) [39], a network modeling strategy was employed to identify genes potentially associated with a higher risk of breast cancer. Starting with four known genes encoding tumor suppressors of breast cancer, they combined gene expression profiling with functional genomic and proteomic data from various species to generate a network containing 118 genes linked by 866 potential functional associations. This network shows higher connectivity than expected by chance, suggesting that its components function in biologically related pathways. Performing cluster analysis of the significantly enriched MSigDB CGP gene collections, it was evident that the highest scoring collection, i.e., the PUJANA_BRCA1_PCC_NETWORK, most closely clustered with the PUJANA CHEK2 (https://www.gsea-msigdb.org/gsea/msigdb/cards/PUJANA_CHEK2_PCC_NETWORK: accessed on 21 February 2023) and ATM (https://www.gsea-msigdb.org/gsea/msigdb/cards/PUJANA_ATM_PCC_NETWORK: accessed on 21 February 2023) functional networks. These gene collections represent datasets constituting the CHEK2 (checkpoint kinase 2) or ATM (ataxia telangiectasia mutated) PCC network of transcripts whose expression positively correlates (Pearson correlation coefficient, PCC ≥ 0.4) with that of CHEK2 or ATM ([39]: Figure 2B). These clustered enriched MSigDB collections indicate that the molecular perturbagen signature induced by GPR19 is closely allied to molecular functions linked with these important DDR proteins. It is important to note that these three primary target proteins, BRCA1, CHEK2, and ATM are all strongly associated with the aging process and stress resilience [40,41,42,43,44,45]. The numerical intersection between the GPR19 input global DEP list (2459 proteins) and these three Pujana-based data collections was assessed for significance and compared using randomly generated DEP lists (of the same 2459 element size) for the same intersection measurement. All three levels of data intersection were found to be highly significant, indicating the presence of profound BRCA1 (624 proteins)/ATM (390 proteins)/CHEK2 (356 proteins) activity in the GPR19 molecular signature (Figure S1). Using the proteins found to be common between the GPR19 molecular signature and the PUJANA-BRCA1/CHEK2/ATM collections, it was found that these data cohorts demonstrated a common link to cellular metabolism, especially the HumanCYc “superpathway of conversion of glucose to acetyl CoA and entry into the TCA cycle” pathway (https://biocyc.org/HUMAN/NEW-IMAGE?object=PWY66-407: accessed on 21 February 2023). Thus, the potent DDR/Aging/Cancer component of the GPR19 molecular signature also appears to be tightly linked to classical energy management pathways—again reinforcing the potential key status of GPR19 in pathological aging paradigms.

**Figure 3:**
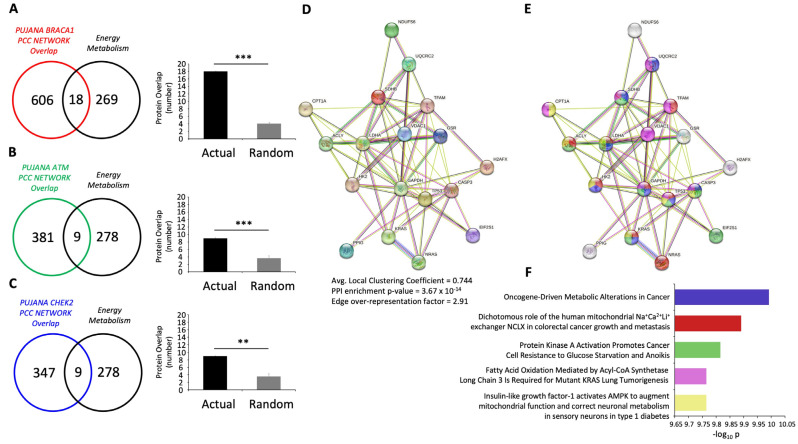
The GPR19 energy and DNA management signature is associated with oncological pathways. Using an unbiased metabolic pathways dataset (created using GLAD4U (http://glad4u.zhang-lab.org/index.php) GeneShot (https://maayanlab.cloud/geneshot/) and PubPular (https://heart.shinyapps.io/PubPular/), the protein identity overlap between the GPR19-BRCA1 (**A**)/ATM (**B**)/CHEK2 (**C**) MSigDB-CGP datasets and this metabolism dataset was found. The numerical extent of these specific dataset overlaps (black bars) was significantly greater than that expected from similarly sized random datasets (grey bars). STRING network analysis of the combined overlapping proteins (from the specific intersections in (**A**–**C**) revealed a potent multifactorial association of these DNA and energy management proteins with oncological pathways (**D**,**E**). The histogram in (**F**) depicts the most significantly populated NCBI-PubMed manuscript searches using the compounded data shown in the networks (**D**) (plain) and (**E**) (proteins color coded to the histogram bars in (**F**). Histogram-based data shown represent the means ± SEM (standard error of the mean). The significance level is indicated in each figure as ** *p* ≤ 0.01; *** *p* ≤ 0.001.

To test this in an unbiased, orthogonal manner, we created a de novo molecular signature consisting of 287 proteins related to energy metabolism (see Table S7). The signature was generated using a combination of extracted and common proteins, which were defined using natural language processing (NLP) applications, including GLAD4U (Gene List Automatically Derived For You-http://glad4u.zhang-lab.org/index.php#: accessed on 21 February 2023) [46], GeneShot (https://maayanlab.cloud/geneshot/: accessed on 21 February 2023) [47], and PubPular (https://heart.shinyapps.io/PubPular/: accessed on 21 February 2023) [48] with the following input interrogator terms: “energy metabolism”, “oxidative phosphorylation”, and “glucose metabolism”. Performing a significance analysis of the magnitude of intersection between the GPR19-BRCA1 (624 proteins)/ATM (390 proteins)/CHEK2 (256 proteins) datasets and this de novo metabolism dataset demonstrated that the level of intersection between these two data corpora was highly significant (using random datasets that mimic the 287-metabolism protein cohort) (Figure 3A–C). Collecting together (Figure S2) the three levels of protein intersection (GPR19-BRCA1 and metabolism–18 proteins; GPR19-ATM and metabolism–9 proteins; and GPR19-CHEK2 and metabolism–9 proteins) created a functional STRING (https://string-db.org/: accessed on 21 February 2023) interaction network (Figure 3D) strongly associated with cancer physiology (Figure 3E), i.e., the most enriched (FDR 1.03 × 10^−10^) associated PubMed Reference text was “Oncogene-Driven Metabolic Alterations in Cancer” [49] (Figure 3F). Thus, the strong functional intersection between the GPR19 molecular signature and DNA management pathways seems to be intricately intertwined with both metabolic and oncogenic signaling systems.

### 2.4. GPR19 Perturbagen Responses Demonstrate a Complex “Dose-Dependent” Functional Diversity

**Figure 4:**
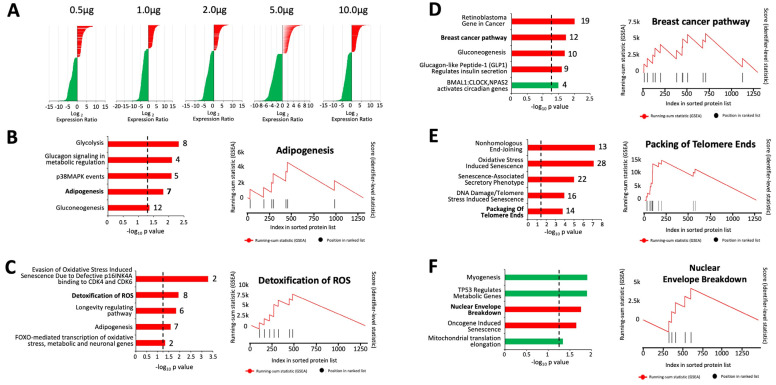
GPR19 expression level alteration reveals a progressive response system associated with aging-induced cellular pathology. The relative numbers of up− or down-regulated proteins significantly changed in cells in response to the dose-range of ectopic GPR19 expression (**A**). Protein expression bars indicated in red indicate upregulation in response to GPR19 expression; downregulated proteins are indicated by green bars. Applying Kolmogorov−Smirnoff-based pathway enrichment analysis to each of the distinct dose-dependent GPR19 response datasets revealed a strong phenotypic diversity in the molecular response to the GPR19 perturbation. Positively stimulated pathways (populated by upregulated proteins) are indicated in red, while negatively stimulated pathways (populated by downregulated proteins) are indicated in green. At the lowest expression level (0.5 μg–(**B**)), glucose and adipose regulatory pathways are dominant. At the next expression level (1 μg–(**C**)), ameliorative responses to oxidative stressors are observed. At the median expression level in the expression range (2 μg–(**D**)), the implication of GPR19 in oncological pathways is demonstrated. At the next level of GPR19 perturbagen expression (5 μg–(**E**)), DNA damage/telomere protective activity is observed. At the highest level of GPR19 expression (10 μg–(**F**)), responses to critical levels of cell stress are observed that may be linked to final cell fate decisions in the aging/damage process.

To further interrogate the nuances of the GPR19 perturbagen molecular signature, we next applied K–S based pathway analysis to each of the specific “dose” levels of GPR19 expression, i.e., 0.5 μg, 1 μg, 2 μg, 5 μg, and 10 μg levels of GPR19 cDNA transfected. For each of these different expression concentrations, a similar total magnitude of DEPs was found at each “dose” level: 0.5 μg 1335 total DEP (42.2% upregulated–57.8% downregulated); 1 μg 1465 total DEP (31.1% upregulated–68.9% downregulated); 2 μg 1369 total DEP (33.9% upregulated–66.1% downregulated); 5 μg 1563 total DEP (37.4% upregulated–62.6% downregulated); 10 μg 1513 total DEP (35.8% upregulated–64.2% downregulated) (Figure 4A). This similarity is interesting as it removes the potential for any magnitude bias in further K–S-based analyses in the panels in Figure 4B–F. At the lowest expression level (0.5 μg) pathways linked to glucose-based metabolism and adipocyte functionality were prominent (Figure 4B). At the 1 μg GPR19 expression level, there was a greater representation of ROS (reactive oxygen species) stress resistance, and longevity-regulating pathways were also prominent (Figure 4C). At the 2 µg expression level of GPR19, the presentation of breast cancer, metabolic, and circadian rhythm functions were seen (Figure 4D). At the 5 μg level of GPR19 expression, a phenotype linked to senescence, DNA damage, and pro-aging/telomere attrition was found (Figure 4E). At the very highest GPR19 expression level, profound disruptions to muscular function, p53-mediated cell control, and aging-related cellular degradation (nuclear envelope breakdown) were prominent (Figure 4F). It is noteworthy that, as the level of GPR19 expression increases, the functional responsive phenotype changes. Specifically, at the 0.5 µg expression level, alterations in energy metabolism sources (from glucose to adipose) are observed. This transition then progresses to oxidative damage resistance at 1 μg expression level, which is potentially induced by loss of glycometabolic function. At 2 μg expression level, there is an intersection between cancer/metabolism/circadian rhythm. At 5 μg expression level, DNA damage is observed, and at the highest expression level (10 μg), cell destruction and senescence occur. This progressive transition of responses based on expression level suggests that GPR19 may be playing a multidimensional facilitatory role in managing the aging process across the lifespan in an analogous manner to GIT2 [18,22,23].

### 2.5. Distinctive Compartment-Based Interpretation of Gpr19 Perturbagen Response Indicates a Persistent Molecular Signature of DNA Damage Management, Energy Regulation, and Cancer Physiology

**Figure 5:**
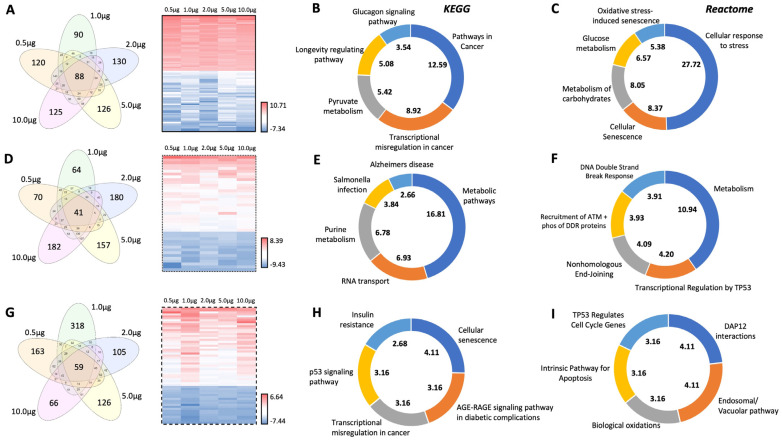
Consistent GPR19 energy-DNA management molecular signatures also occur in divergent protein extraction compartments. Separating GPR19 perturbagen datasets into cytoplasmic (solid line, (**A**−**C**), plasma membrane (dotted line, (**D**–**F**) or nuclear/organelle (dashed line, **G**–**I**), a commonly found core of proteins was identified (found at all levels of GPR19 perturbation) that when subjected to either KEGG (**B**,**E**,**H**) or Reactome (**C**,**F**,**I**) pathway analysis was again able to represent a pathway-based impression of energy and DNA integrity management. The associated heatmap key indicates the range of log_2_ transformed GPR19: mock expression ratios.

Using multiple independent mechanisms of investigating the nuances of the GPR19 molecular signature, we have shown that this receptor is tightly linked to cell stress response mechanisms linked to protecting cells against factors that promote aging and alterations of cell fate. We have demonstrated this functional phenotype using global protein analysis as well as at differential expression levels. Next, we sought to discover whether this signature was sufficiently penetrant to also exist among the three distinct cellular compartment extracts created using the aforementioned Qproteome DDF protocol. To isolate the specific variable of the protein extraction procedure (cytoplasmic, plasma membrane, nucleus/organelle), we identified the DEPs that were common across all of the varied expression levels of GPR19 in the different protein extraction cohorts (Figure 5). Concerning the cytoplasmic extraction process, we found eighty-eight specific DEPs that were found consistently across all the GPR19 expression doses (Figure 5A and Table S8). Using this 88 DEP list specific to the cytoplasmic compartment, both KEGG (Figure 5B) and Reactome (Figure 5C) pathway analysis of this DEP list revealed a consistent signaling phenotype associated with oncology (“pathways in cancer”; “transcriptional misregulation in cancer”), stress resistance (“oxidative stress-induced senescence”), glucose metabolism (“glucagon signaling pathway”, “pyruvate metabolism”, “glucose metabolism”, “metabolism of carbohydrates”), and longevity regulation and cell fate (“longevity regulating pathway”, “cellular senescence”). For the plasma membrane extract, 41 GPR19 expression-independent DEPs were found (Figure 5D and Table S9). With KEGG (Figure 5E) and Reactome (Figure 5F) pathway investigation of this DEP list again a consistent molecular phenotype was observed. This means that it is associated with glucose metabolism (“metabolic pathways”, “metabolism”), oncology (“transcriptional regulation by TP53”), and DNA damage response (“DNA double-strand break response”, “Recruitment of ATM and phosphorylation of DDR proteins”, “nonhomologous end-joining”). For the analysis of the nuclear/organelle extracted GPR19 DEP lists, 59 DEPs were found to be consistent across the different expression levels (Figure 5G and Table S10). Upon KEGG (Figure 5H) and Reactome (Figure 5I) pathway interpretation further consistent oncology (“p53 signaling pathway”, “transcriptional misregulation in cancer”, “TP53 regulates cell cycle genes”), metabolism (“insulin resistance”, “AGE-RAGE signaling pathway in diabetic complications”), stress (“biological oxidations”) and cell fate/damage (“cellular senescence”, “intrinsic pathway for apoptosis”) multidimensional phenotype was evident. Therefore, as with our previous analytical streams using global and dose-specific DEP data, the consistent GPR19 aging-associated phenotype was also found in an extract compartment-specific manner (Figure 5).

With this consistent pathway signature across the three different cellular compartment extracts, we next sought to assess the degree of direct DEP conservancy across the three protein extracts (Figure S3A). In this, we found five completely consistent proteins common across all the GPR19 expression doses in all three distinct cell compartment extracts: MDC1; MTHFD1L; PDCD5; ANP32C; MGEA5; SETBP1. Consistent with the overall molecular phenotype of GPR19, many of these proteins are linked to DNA damage [22,50,51,52], energy metabolism [53,54,55,56,57,58,59], and oncology [60,61,62,63,64,65]. Generating a heatmap of these conserved DEPs, it was evident that a diverse array of expression polarities were found for these proteins across the distinct GPR19 expression doses/compartment extracts (Figure S3B). Within this small DEP cohort, we chose MDC1 to verify its expression profile across all of the doses/cell compartments with correlation to the proteomic data (Figure S3C). The western blot MDC1 (mediator of DNA damage checkpoint protein-1) analysis confirmed the expression polarity variation of MDC1 across the different cell conditions shown in Figure S3C. We have previously shown in our research that MDC1 participates in both ATM and GIT2 functionality [22] in the context of pathological aging. In addition, MDC1 has been strongly linked by others to oncogenic pathways [60], oxidative stress effects [66], longevity [67], adipose function [68], and insulin receptor signaling [69]—all of which may serve as a microcosm of GPR19 activity. Given this multidimensional activity linked to several aspects of aging-related biology, GPCR signaling, and its significant and consistent regulation by GPR19, we next investigated how this specific factor could potentially be a critical point for GPR19 activity in aging contexts. It is of course likely that the other proteins identified in Figure S3 (MTHFD1L, PDCD5, ANP32C, MGEA5, SETBP1) also play important roles in this paradigm, and thus their further investigation will likely yield essential information regarding the stress-responsive role of GPR19 in aging. Future research may indeed reveal that it is also possible to functionally link these factors to MDC1-related pathobiology as well.

### 2.6. Extracting MDC1-Associated Signaling Components from the Global GPR19 Perturbagen Signature

**Figure 6:**
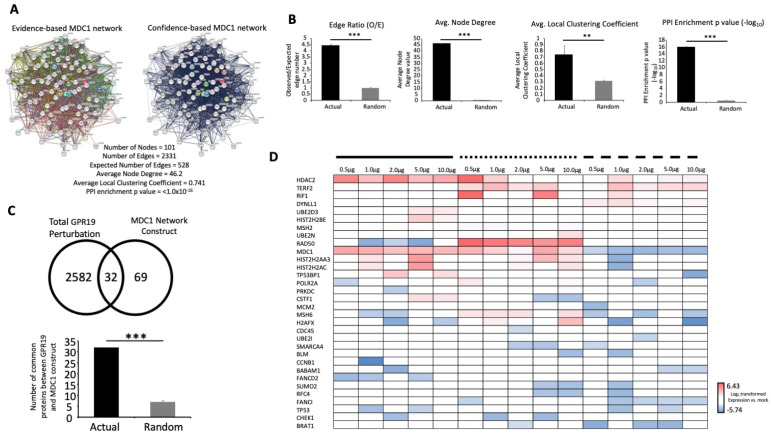
Extracting an MDC1−specific signature from the GPR19 perturbagen data corpus. Given the potential significance of MDC1 in the functional signature of GPR19, an unbiased MDC1 association network of the most proximal and significant one-hundred proteins was derived using STRING (**A**). This network was shown to be highly significant in its network characteristics compared to random data corpora of the same numerical size (**B**). Observing the intersection between proteins common to both the GPR19 perturbagen data set and the MDC1-specific network, we found that over a third of the MDC1 network (32 proteins) was significantly regulated by GPR19 perturbation (**C**). This level of overlap was significantly greater than that expected using a comparable-sized (to the actual MDC1 network) random dataset (**C**). The expression profile of GPR19−MDC1 common signature proteins (32 DEPs) is indicated in panel (**D**). The associated heatmap key indicates the range of log_2_ transformed GPR19: mock expression ratios. Histogram-based data shown represent the means ± SEM (standard error of the mean). The significance level is indicated in each figure as ** *p* ≤ 0.01; *** *p* ≤ 0.001.

As MDC1 appears to be a potentially crucial factor in the GPR19 functional perturbagen signature, we chose to create an unbiased in silico MDC1 signature based on both text mining and physical/functional association data using STRING (https://string-db.org/: accessed on 21 February 2023). Thus, a signature based on the initial seed of human MDC1 was created using a multiplex of data sources including text mining, empirical experiments, curated databases, co-expression data, chromosomal neighborhood proximity, gene-fusion data, and co-occurrence data. The first one hundred most strongly interacting protein factors (plus MDC1) were chosen to represent the most proximal functional network linked to MDC1 (Figure 6A). This network was then analyzed for multiple network parameters (observed/expected edge ratio; average node degree; average local clustering coefficient; protein–protein enrichment ratio) using comparison with similar analyses of ten random protein networks (Figure 6B). For each of these parameters, the actual MDC1 network demonstrated a significantly higher score for all the described network properties. We next assessed the numerical intersection between the actual MDC1 network and the GPR19 perturbagen DEP list (Figure 6C). Thirty-two specific GPR19 DEPs were found to be common with the unbiased MDC1 network. This degree of GPR19-MDC1 network association was significantly greater than that expected for random data lists the same size as the actual MDC1 network (Figure 6C). The expression pattern, across cDNA dose and cell compartment extracts, of the cohort of 32 MDC1-associated, GPR19-perturbed proteins is depicted in Figure 6D.

**Figure 7:**
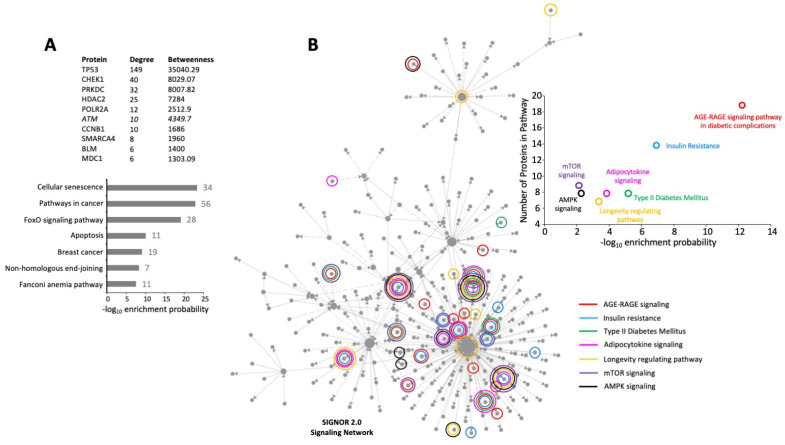
The GPR19−MDC1 intersection data corpus encapsulates a microcosm of energy-DNA management functionality. A signaling-based minimal network (SIGNOR 2.0 Database—NetworkAnalyst: https://www.networkanalyst.ca/: accessed on 21 February 2023) was created for the GPR19-MDC1 32 protein intersection and was found to be centered upon many of the input thirty-two proteins (9/10 top network-controlling proteins were from the input thirty-two protein data corpus: (**A**). Applying KEGG signaling pathway annotation to the SIGNOR 2.0 network, an encapsulation of the overall GPR19 perturbagen phenotype was found, i.e., prominent pathways linked to cancer, DNA damage, and cell fate were defined. The signaling network was additionally found to be effectively linked to multiple proteins linked to classical signs of metabolic dysfunction. This indicates a novel functionality for GPR19−MDC1 (**B**). The proteins across the signaling network associated with the metabolic pathways are color coded on the network and correlate with the associated enrichment plot (**B**).

To investigate the functional nature of the potential capacity of MDC1 signaling via GPR19, we applied a SIGNOR 2.0 Signaling Network (https://www.networkanalyst.ca/: accessed on 21 February 2023) overlay to the 32 DEP list (Figure 7A). Using a minimal network approach with the SIGNOR 2.0 database (https://signor.uniroma2.it/APIs.php: accessed on 21 February 2023), a functional signaling network for the GPR19-MDC1 intersection was generated (Figure 7A,B). The top ten most influential proteins (possessing the greatest node degree and betweenness scores) in this signaling network contained nine proteins from the direct input 32 DEP list (except for ATM: Figure 7A). Using pathway analysis of this signaling network, it was evident that this small 32 DEP cohort was able to encapsulate an effective microcosm of the overall GPR19 perturbagen phenotype, i.e., the most enriched pathways included those linked to oncology (“pathways in cancer”, “breast cancer”), cell stress and fate (“cellular senescence”, “apoptosis”), DNA damage management (“non-homologous end-joining”, “Fanconi anemia pathway”), and longevity-associated factors (“FoxO signaling pathway”). Hence it appears that the MDC1-associated components of the global GPR19 perturbagen dataset seem to potentially control and regulate many aspects of its functionality. In addition to these classical functions attributed to MDC1, i.e., linked to DDR pathways, we next assessed whether this GPR19-MDC1 intersecting network would be linked to metabolic functions linked closely to the global aging process [23,70,71,72]. Within the created GPR19-MDC1 SIGNOR 2.0 network, it was evident that multiple energy metabolism-related signaling pathways were enriched by the coordinated expression of proteins across the whole constructed network. These pathways included the “AGE-RAGE signaling pathway in diabetic complications”, “insulin resistance”, “type II diabetes mellitus”, “adipocytokine signaling”, “longevity regulating pathway”, “mTOR signaling”, and “AMPK signaling”. All of these signaling paradigms are vital to multiple aspects of the aging process [73,74,75,76,77,78,79].

### 2.7. Protein Subcomplex Analysis of the GPR19 Perturbagen Response Phenotype

**Figure 8:**
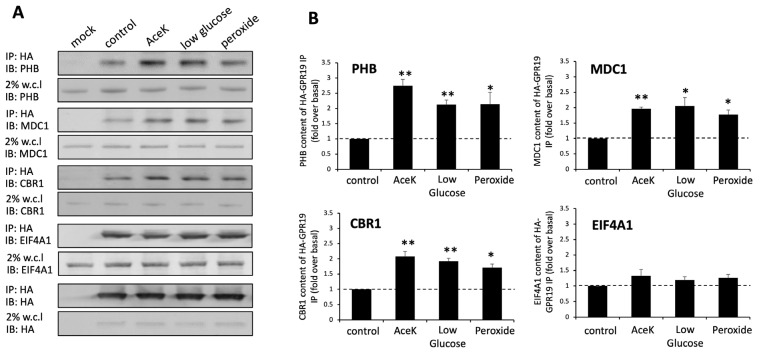
Protein subcomplex analysis of GPR19 immunoprecipitates. To assess the potential capacity for MDC1 and/or prohibitin (PHB)-associated complexes to link with GPR19, co-immunoprecipitation of MDC1, PHB, CBR1, and EIF4A1 with HA-epitope-tagged GPR19 was assessed in control and also aging-related stress conditions (AceK–Acesulfame K; low glucose; peroxide–hydrogen peroxide exposure) (**A**). Western blots indicate the specific protein presence in either the immunoprecipitated (IP) or the input whole cell lysate (w.c.l, 2% of total cell lysate loaded). Histograms indicating the mean of three co-immunoprecipitation replicates indicate stress-related potentiation of MDC1, PHB, and CBR1 (but not EIF4A1) with GPR19 (**B**). Histogram-based data shown represent the means ± SEM (standard error of the mean). The significance level is indicated in each figure as * *p* ≤ 0.05; ** *p* ≤ 0.01. Pathway enrichment probability was calculated via over-representation analysis (ORA).

As we have shown the MDC1 protein to be a potentially vital component of the GPR19 perturbagen response phenotype, we next pursued this concept further to investigate the potential for small subcomplexes to coordinate the signaling processes emanating from GPR19 [6,16,33,37,80]. Hence, we investigated the GPR19 perturbagen response data from the three distinct cellular compartment extractions using enrichment analysis of the CORUM protein microcomplex database (http://mips.helmholtz-muenchen.de/corum/: accessed on 21 February 2023) (Figure S4). Using compiled global GPR19 perturbagen response data from all fractions, we found a potent enrichment of microcomplexes linked to DNA damage responses (“condensin I-PARP-1-XRCC1 complex”, “H2AX complex”), transcriptional control (“BRG1-SIN3A-HDAC containing SWI/SNF remodeling complex”), molecular chaperoning (“prefoldin complex”) and most consistently, energy metabolism (“Prohibitin complex, mitochondrial”, “Respiratory chain complex I (intermediate), mitochondrial”, “respiratory chain complex I (incomplete intermediate), mitochondrial”, “IGF2BP1 complex”) (Figure S4A). Focusing upon the cytoplasmic extract components of the GPR19 perturbagen response, the most prominent complex enrichments were found for those associated with oncological/metastatic signaling (“MTA1-HDAC core complex”, “MTA1 complex”, “NuRD.1 complex”, “ARF-Mule complex”), DNA modification (“MLL2 complex”, “SIN3 complex”), DNA damage regulation (“DNA ligase III-XRCC1-PNK-DNA-pol III multiprotein complex”, “H2AX complex II”, “DNA ligase III-XRCC1 complex”), and again the highest scoring enriched complex was the Prohibitin complex, mitochondrial (Figure S4B). Applying CORUM complex enrichment, the plasma membrane extract data for GPR19 again identified a prominent enrichment of the Prohibitin complex in the mitochondrial (Figure S4C). In addition to this, there was a strong series of enriched complexes associated with DNA modification (“NCOR1 complex”, “BAF complex”), and DNA damage/longevity regulating functions (“BLM-TRF2 complex”, “CRBN-DDB1-CUL4A-RBX1 E3 ubiquitin ligase”, “condensin I-PARP-1-XRCC1 complex”, “BRD4-RFC complex”, “TFIIIC containing-TOP1-SUB1 complex”). Interestingly, for the nucleus/organelle protein extract, a prominent enrichment was not found for the “prohibitin complex, mitochondrial” (Figure S4D). This is while the top 10 most enriched complexes were mainly focused upon DNA modification (“MeCP2-SIN3A-HDAC complex”, “ASF1-histone containing complex”, “BRG1-SIN3A-HDAC containing SWI/SNF remodeling complex I”), DNA replication (“MCM complex”), and DNA damage management (“MDC1-H2AFX-TP53BP1 complex”, “condensin I-PARP-1-XRCC1 complex”). It was interesting to note that the highest-scoring complex enrichment in this protein extract involved MDC1 in the MDC1-H2AFX-TP53BP1 complex (Figure S4D). This finding then potentially suggests that both MDC1 and Prohibitin (PHB) complexes may be important for translating the biological activity of GPR19. PHB is a multifunctional protein that has been associated with a diverse array of functions, including mitochondrial energy production [81,82], aging [83,84], apoptosis [85,86], and cancers [87,88] especially breast cancer [89,90]. It has previously been shown that PHB can functionality associate with GPCR [6,91,92] complexes and thus we attempted to investigate whether both MDC1 and PHB may also occur in a complex with GPR19. Performing anti-HA co-immunoprecipitations in the low-detergent conditions used previously [6], we found that in control conditions (compared to mock transfected cells: Figure 8A), both MDC1 and PHB were co-enriched with the HA-tagged GPR19. In addition to these two target proteins, we also verified that GPR19 immunopositive complexes also contained EIF4A1 and CBR1. We have previously shown that the elongation factor EIF4A1 can efficiently bind the RXFP3 receptor [6]. In accordance with our findings, other groups have shown functional associations of EIF4A1 with further GPCRs, including G protein-coupled receptor, class C, group 5, member A (GPCR5A: [93]); beta2-adrenoceptor (ADRB2: [92]). In addition, CBR1 (carbonyl reductase 1) has also been shown to associate with other GPCRs including the angiotensin II receptor, type 1 (AT1R: [94]), the chemokine (C-C motif) receptor 1 (CKR1: [95]), G protein-coupled receptor 18 (GPR18: [96]), the 5-hydroxytryptamine (serotonin) receptor 2B (5-HT2B: [94]). Given the potential role(s) of MDC1 and PHB in coordinating the functions of GPR19, we also assessed whether cellular insults that mimic the aging process and metabolic dysfunction affected the interaction of these factors with the receptor. For both PHB and MDC1, their co-precipitation with GPR19 was augmented after cellular treatment with the mitochondrial disruptor Acesulfame K AceK: [97], glucose deprivation, or the oxidative stressor hydrogen peroxide (Figure 8A,B). As with MDC1 and PHB, an increase in the CBR1 content of GPR19 immunoprecipitates was observed with the applied cell stressors (Figure 8A,B). In contrast to MDC1, PHB, and CBR1, the EIF4A1 content of the GPR19 immunoprecipitates was not significantly changed by AceK, low glucose exposure, or hydrogen peroxide.

### 2.8. Verification of the Potential Biological Significance of an MDC1-PHB Functional Interaction

**Figure 9:**
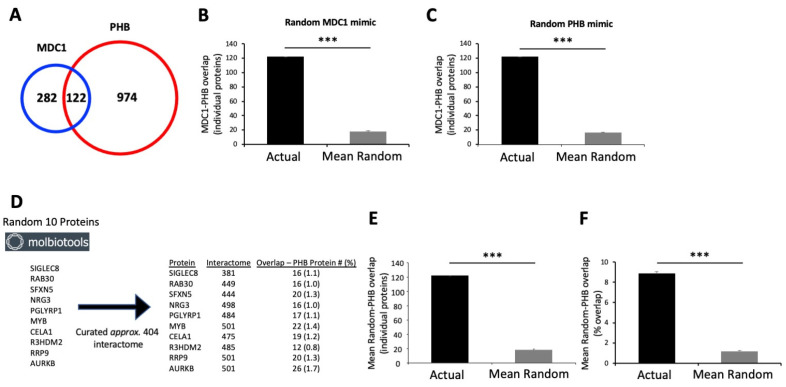
Rationalizing a potential MDC1-PHB co-complex. Using the curated physical protein-protein interaction database at BioGrid (https://thebiogrid.org/: accessed on 21 February 2023), an interaction data corpus was identified for MDC1 (404 interactors) and PHB (1096 interactors). The intersection between these two corpora represented 122 proteins (**A**). Using randomly generated protein lists to mimic either the MDC1 (**B**) or PHB (**C**) interactor data corpus, the statistical significance of the intersection between the real data (**A**) was demonstrated. This suggests that such an overlap underlies the biological likelihood of an important MDC1-PHB functionality. In addition, ten randomly chosen proteins were also assessed when their similarly sized (to the MDC1 BioGrid interactor dataset) STRING networks were intersected with the actual PHB interactor corpus (**D**). Using the mean of the actual random protein overlap with PHB (at the number (**E**) and percentage levels (**F**)), a statistically significant distinction between random proteins and MDC1 intersection with PHB was found. The significance level is indicated in each figure as *** *p* ≤ 0.001.

Given their functional cooperation, such as involvement in DNA damage management [98,99], energy metabolism [82,100], and stress resistance [101,102], and their important roles in the GPR19 functional signature (Figure 5, Figure 6, Figure 7, Figure 8 and Figure 9), we next sought to investigate how these two factors may interact with each other. Using curated empirical interaction data from the BioGrid resource (https://thebiogrid.org/: accessed on 21 February 2023), we assessed how the coterie of known interactors of MDC1 (404 proteins) and PHB (1096 proteins) intersected. Performing this initial intersection analysis, we found 122 common proteins (Figure 9A). Using a random gene list generator application (https://molbiotools.com/randomgenesetgenerator.php: accessed on 21 February 2023), two diverse groups of artificial lists (*n* = 10) of proteins were generated that were of the same numerical magnitude as the MDC1 (404 proteins) or the PHB (1096 proteins) BioGrid interactome datasets. Thus, by replacing either the MDC1 or PHB lists with randomly generated lists (*n* = 10 for each scenario) and then re-assessing the level of intersection, we found that the actual dataset intersection analysis (Figure 9A) was highly significant compared to the random datasets (Figure 9B,C). Hence, the degree of interaction between MDC1 and PHB-interacting proteins is highly likely not to be random, thus suggesting a relevant biological association. To assess this potential MDC1-PHB association via another methodology, interaction lists (of a similar numerical size to the actual MDC1 404 protein datasets) were created for ten randomly chosen (using molbiotools.com/randomgenesetgenerator.php) proteins (SIGLEC8, RAB30, SFXN5, NRG3, PGLYRP1, MYB, CELA1, R3HDM2, RRP9, and AURKB) (Figure 9D). These were then assessed for their numerical and percentage intersection with the actual PHB BioGrid interactome data (Figure 9D–F). In terms of both the numerical (Figure 9E) and percentage intersection (Figure 9F) of the random protein interactomes with the PHB empirical data, it was evident that none of these proteins possessed a level of intersection anywhere near that of the actual MDC1 data. Thus, the protein-based intersection between MDC1 and PHB appears not to be at all random.

We next performed a multi-platform signaling assessment of this 122-protein set (Figure S5A–C). Using the KEGG, WikiPathways, and Reactome signaling pathway databases, an unbiased analysis of the 122-protein set was performed. It was evident that a similar functional phenotype of activities linked to (i) energy metabolism, (ii) aging/DNA damage, and (iii) cancer biology was found in the different curated databases. Thus, the common interacting proteins between MDC1 and PHB seem, in themselves, to represent a functional phenotype highly reminiscent of the global GPR19 perturbagen response.

### 2.9. Interaction Analysis of Potential MDC1 PHB Functional Intersection

**Figure 10:**
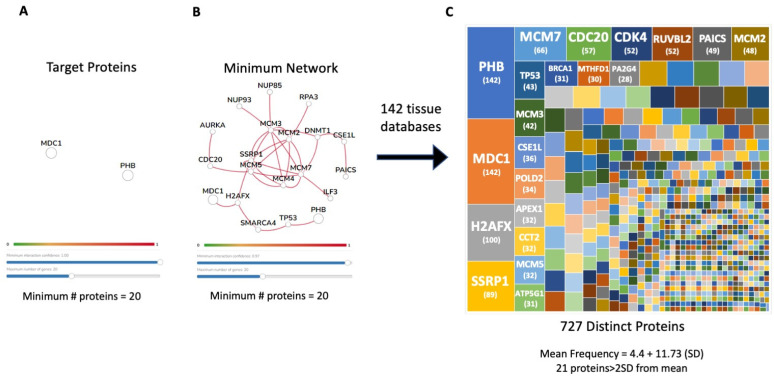
Unbiased definition of potential MDC1-PHB complexes. The tissue-independent functional association network linking MDC1 and PHB was assessed using connection dynamics across the curated tissues in the HumanBase database of interaction modules. Applying a maximum connection network at the highest confidence level, the minimal bridging network linking MDC1 to PHB was found for the 142 distinct tissue databases at HumanBase (**A**,**B**). Across the 142 tissues, 727 distinct total proteins were found, and their identification frequency (linking MDC1 to PHB) was calculated. Using class-based statistics upon these 727 proteins list, the proteins (21) with a statistically significant distinct frequency to the mean were identified (**C**). These twenty-one proteins have their identification frequencies indicated across the 142 distinct tissues represented.

To study the somatic physiological mechanisms linked with potential MDC1-PHB functionality, we initiated a protein network interaction analysis using the 142 tissue-specific protein–protein interaction datasets at HumanBase (https://hb.flatironinstitute.org/: accessed on 21 February 2023). Initially, both MDC1 and PHB are chosen as instigator targets for complex analysis (Figure 10A). With the following network creation settings, i.e., <20 proteins in the network and the highest level of network confidence, the minimal number of additional network proteins required to bridge the two targets (MDC1 and PHB) in each of the 142 curated tissue datasets [103] was assessed (Figure 10B). With the repetition of this process across the 142 tissue databases at HumanBase, a grand total of 728 proteins allowed the capacity to bridge a physical/functional interaction between MDC1 and PHB. A frequency analysis to indicate the number of tissue networks in which each of these 728 proteins were found was performed to indicate proteins that possessed a statistically considerable number of network inclusions compared to the group’s average number of tissue inclusions. The average number of networks that each protein could be involved with was 4.40 + 0.43 (mean + standard error of the mean). A total of twenty-one proteins were found to have several tissue network associations that were more than two standard deviations greater than the mean (Figure 10C). These twenty-one proteins, therefore, represent the most important tissue-independent mediators of MDC1-PHB functionality.

**Figure 11:**
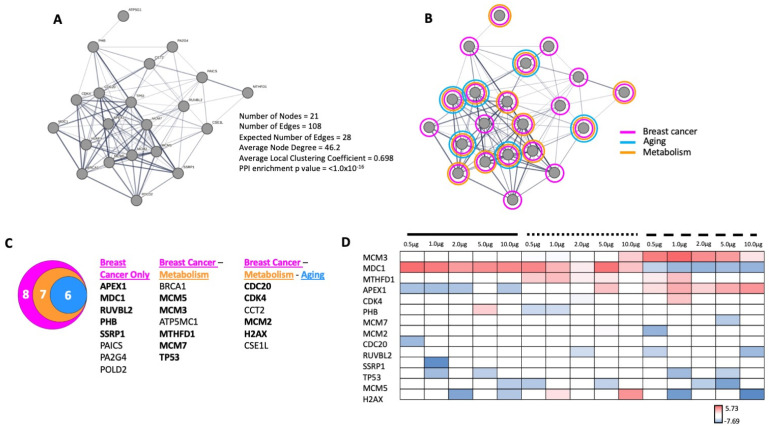
Network analyses of MDC1−PHB associated bridging factors. These tissue-independent linking factors between MDC1 and PHB were clustered using STRING (**A**) and then annotated using enrichment with the MSigDB CGP database (http://www.gsea-msigdb.org/gsea/msigdb/human/genesets.jsp?collection=CGP: accessed on 21 February 2023). The proteins that enriched the CGP database linked to breast cancer (pink), aging (blue), and metabolism (orange) cover the entire tissue-independent MDC1−PHB connecting network (**B**). Separating these proteins into these distinct functional groups identified that all the proteins lie within the group of those related to breast cancer (**C**). The proteins also linked to aging/DNA damage, and metabolism form specific subsets within the breast cancer-related networks. Of the twenty-one significant MDC1−PHB tissue-independent bridging proteins, fourteen of these (**D**) were found to be significantly altered in the initial GPR19 perturbagen dataset. The associated heatmap key indicates the range of log_2_ transformed GPR19: mock expression ratios.

When functional linkages between these proteins were assessed (Figure 11A) using STRING, we found that all of these proteins were associated with multiple signaling collections (MSigDB CGP) linked to breast cancer, aging, and energy metabolism (Figure 11B), which effectively encapsulated the global functionality estimates of the GPR19 perturbagen response phenotype (Figure 2 and Figure 5). These twenty-one proteins that populated the three groups of MSigDB CGP collections could be condensed into one global breast cancer-related dataset (Figure 11C) with two inclusive subsets (aging and energy metabolism). Of these twenty-one proteins generated entirely through curated database analysis, fourteen (Figure 11C,D) were found to be significantly altered through our in cellula GPR19 perturbation experiment.

### 2.10. Expression of GPR19 Can Attenuate Loss of Cell Viability Induced by Exogenous Cell Stressors

**Figure 12:**
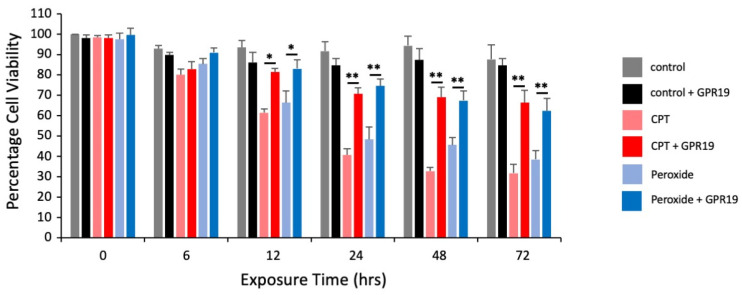
GPR19 expression elevation provides cell viability protection against aging-related stress. HEK293 cell viability was assessed using automated cell viability analysis (Luna II Automated Cell Counter) at 6, 12, 24, 48, and 72 h (h) post-transfection with the median expression level of GPR19 (2 μg), which represented a multidimensional functional capacity with respect to stress resistance. Control HEK293 cells (transfected with 2 μg of an empty vector) or cells transfected with GPR19 were then treated for the indicated time period with either camptothecin (CPT, 1 mM) or hydrogen peroxide (peroxide, 100 nM). At each time point, the effect of GPR19 expression upon control, CPT-treated, or peroxide-treated cell viability was measured. Histogram-based data shown represent the means ± SEM (standard error of the mean). The significance level is indicated in each figure as * *p* ≤ 0.05; ** *p* ≤ 0.01.

Given the potential role of GPR19 in the mediating cellular response to aging-associated insults, we next investigated how GPR19 expression affected cellular viability in response to the application of deleterious stressors, e.g., hydrogen peroxide for oxidative stress or the topoisomerase inhibitor camptothecin (CPT) that causes DNA damage. With the knowledge gained from our dose-dependent functional investigation of the GPR19 perturbation response (Figure 4), we carefully chose the 2 μg expression level of GPR19 for these assessments as this “dose” appeared to possess a multifactorial capacity to both senses and respond to aging-associated insults. Using an automated cell counter-based assessment of cell viability (Luna II Automated Cell Counter: ThermoFisher), the effect of GPR19 (2 μg transfected) expression with either no insult or peroxide/CPT exposure (initiated at the time of initial transfection and supplemented every 12 h) on HEK293 cell viability was assessed at 6, 12, 24, 48 and 72 h following transfection. Exposure of mock-transfected (with 2 μg of pcDNA3.1+ empty vector) cells to peroxide and CPT resulted in a progressive reduction in the percentage of cell viability over time (Figure 12). With the expression of GPR19, these deleterious effects of both peroxide and CPT were significantly attenuated from the 12 h time point on. Thus, the signaling activity/perturbation response to the expression of GPR19 in the HEK293 cells was able to reduce the loss of cell viability induced by both peroxide/CPT.

## 3. Discussion

Aging is a multifactorial process that affects all the tissues in the human body and involves the progressive accumulation of unrepaired damage to cellular proteins and nucleic acids [104,105,106]. The accumulation of damage is driven by the active metabolic processes of ATP synthesis, primarily through mitochondrial functionality [71,107,108,109,110]. A substantial proportion of this cellular damage occurs through the ongoing production of deleterious cellular metabolites, e.g., reactive oxygen species (ROS) [111,112,113]. ROS are likely to not be the sole source of damage, but their link to glucometabolic mitochondrial oxidative phosphorylation is continually reinforced by the demonstration that the insulinotropic glucose metabolic system controls longevity in an enormous variety of species [114,115,116,117,118,119,120]. This glucometabolic stress-related damage degrades the functionality of active signaling systems as well as reactive cytoprotective cellular systems that exist to combat the metabolically induced cellular damage [9,121,122]. In recent years, it has been demonstrated that, as with many other forms of cellular and tissue signaling [7,123,124], stress response and DNA damage repair processes are strongly controlled and regulated by signaling networks composed of multiple GPCR types [14,17,33,125,126]. Thus, well-informed therapeutic targeting of GPCRs holds a strong promise for the generation of a broad series of anti-aging therapeutics.

Given the strong correlation between aging pathophysiologies and protective GPCR systems [6,18,33], we employed a novel model of accelerated neurometabolic aging, i.e., the GIT2KO model [23], to prioritize the identification of coordinated GPCR systems that could function as vital regulators of the aging process. Previously, we identified the crucial activity of the RXFP3 system in aging using this process [6]. Here, we have further identified a novel GPCR system that also appears strongly linked to the aging process, i.e., GPR19. We found that in multiple tissues (both central and peripheral), the expression of GPR19 was significantly elevated in GIT2KO mice compared to control WT animals (Figure 1). This is in contrast to RXFP3, where it was significantly downregulated in GIT2KO mice. Thus, this may suggest that RXFP3 and GPR19 may regulate complementary molecular activities in the aging process. In this regard, it is interesting to note that our research indicated the importance of RXFP3 in glucometabolic activity [127,128,129,130], while GPR19 has been more strongly associated with lipid metabolism [131,132]. In this respect, perhaps simplistically, RXFP3 and GPR19 may function as “yin and yang” in the realm of the balance between glucose or lipid metabolic functionality [18,23,133,134,135,136]. Thus, it may be likely that GPCR systems could possess a nuanced level of interaction between the multiple stress-related factors that control molecular aging [33]. Extending this posit, it is not surprising that the regulation of stress response is a prominent component of the functional molecular perturbagen signature of GPR19 (Figure 1G). To assess the specificity of this “cellular response to stress” pathway enrichment from our GPR19 perturbagen dataset, we performed a similar analysis on a DEP list of a similar magnitude to the GPR19 dataset, generated by another rhodopsin-like GPCR reported to possess a similar G protein coupling to GPR19, i.e., the Apelin receptor (Figure S6). With this analysis, we failed to observe a similar potent enrichment of the “Cellular Response to Stress” pathway. In addition, we also performed a Reactome pathway analysis of ten randomly generated datasets the same size as the GPR19 perturbagen dataset. In none of these analyses did we observe a similar potent enrichment of the “cellular response to stress” pathway. Hence, it appears that this demonstrated function of GPR19 is indeed strongly linked to this specific receptor. Furthering this, it was noted that this stress response molecular function of GPR19 was based upon its capacity to regulate DNA damage management systems (Figure 1H). When we continued an in-depth investigation into the GPR19 molecular signature, we found that this receptor’s functional signature was closely allied to dataset collections linked to well-characterized DNA damage response (DDR) systems centered upon BRCA1 (BReast CAncer gene 1), ATM (ataxia-telangiectasia mutated), and CHEK2 (checkpoint kinase 2) (Figure 2A,B). It is interesting to note in this respect that BRCA1 and ATM have both been shown to be crucially involved in GIT2-associated DDR processes [22]. As we have previously discussed, there is a near-inevitable link in aging between metabolic dysfunction and DNA damage [6,71,137,138,139]. With this knowledge, we assessed whether the DDR-based stress response component of the GPR19 molecular signature was also linked to energy metabolic processes. In this regard, we found that a consistent energy generation phenotype was present in all three of the DDR-associated MSigDB collections (Figure S1). Hence, we further demonstrated that this interconnected functionality (energy metabolism and DDR) seen within the GPR19 molecular signature results in a potent link between GPR19 functionality and oncological signaling pathways (Figure 3). We next investigated whether there was any diversification of this functional convergence across the diverse levels of GPR19 expression in our in cellula paradigms. Given we found an elevation from the control to pathological aging model, it is likely that in healthy situations GPR19 expression may be low, and then with aging/pathophysiology the expression of GPR19 increases. To investigate how this shift of expression may control distinct aspects of aging we performed pathway investigations of the different expression levels of GPR19 in our signature paradigms (Figure 4A). Interestingly, we found a transitional variation in the expression levels of the functions of GPR19, with increasing levels. At the lowest expression level (0.5 μg cDNA), the GPR19 molecular signature was associated with both glucometabolic and lipid/adipose-based functionality (Figure 4B). This functional state could be analogized with the initial metabolic phases of aging, where there is a burgeoning glucometabolic dysfunction that then leads to the employment of lipid-based metabolic pathways to regain energy generation [23,140,141,142,143,144,145]. With a further incremental increase in GPR19 expression (1 µg), energy metabolism pathways were co-represented with signaling pathways linked to oxidative stress responses, nutrient sensation, and longevity regulation. The presence of mitochondrially-generated ROS represents the most probable cause of age-related DNA/protein damage during the aging process [71,146]. Therefore, higher GPR19 levels seem to be linked to regulatory processes that may need to be invoked to deal with greater cell stress (ROS-mediated) caused by continued dysfunction of energy metabolism. Interestingly, at the 2 µg expression level, there appeared to be a complex diversity of predicted GPR19 functionality. This means that functionality was linked to energy regulation (“glucagon-like Peptide-1 (GLP1) regulates insulin secretion”, “gluconeogenesis”), cell cycle/fate (“retinoblastoma gene in cancer”), cancer biology (“retinoblastoma gene in cancer”, “breast cancer pathway”) and circadian rhythm (“BMAL1:CLOCK, NPAS2 activates circadian genes”) (Figure 4D). Hence, at this level, there seems to be an inflexion point where the link between energy metabolic alterations and an eventual cancer-based cell fate can occur. At the higher GPR19 expression levels, GPR19 functions seem to be potentially associated with contending with deleterious cell outcomes. Hence, at 5 μg GPR19, there was a potent phenotype for DNA damage management/telomere control in response to elevated ROS, which in a physiological scenario would occur due to mitochondrial failure and the switch between glucose metabolism and lipid usage as an energy source (Figure 4E). At the highest GPR19 expression level induced (10 μg cDNA; Figure 4F), a significant population of signaling pathways appeared to be depleted. These pathways were populated by proteins whose expression was suppressed by the presence of GPR19, such as “myogenesis”, “TP53 regulates metabolic genes”, and “mitochondrial translation elongation”. All of these functional aspects suggest that GPR19 at this excessive expression level may be a physiological regulator of end-stage cell fate/terminal gerontology [147,148,149,150]. The enriched pathways (i.e., populated by GPR19-upregulated proteins) represented either oncogene-induced cell senescence or nuclear lamina breakdown. Hence, at this point, GPR19 appears to be marshaling activities linked to negative cell fates associated with oncogenesis or pro-aging phenotypes linked to nuclear breakdown that are characteristic of accelerated aging programs [151,152,153,154]. Hence, within the dose-series of GPR19 expression, we found a potent degree of linkage between GPR19 functionality and the aging-based triumvirate of “DDR-oncology-energy metabolism”. This functional triumvirate of GPR19 functions not only existed at the global level (Figure 1 and Figure 2) and the dose-dependent level (Figure 4), but also within the distinct proteomes of the DDF protein extraction datasets (Figure 5). Hence, protein expression responses within all of the extracted cellular compartments were linked to the enrichment of signaling pathways linked with DDR, oncology, and metabolic stress response pathways. Based on these investigations, we further identified a series of proteins that were highly conserved in their expression profiles across these three DDF extracts (cytoplasmic, plasma membrane, and nucleus/organelle), i.e., MDC1; MTHFD1L; PDCD5; ANP32C; MGEA5; SETBP1. We found that MDC1 was significantly altered by GPR19 expression at every level of cDNA expression in all of the DDF extracts. We continued our investigation into this factor to uncover why such a strong persistence of GPR19-regulated expression occurred. To this end, we demonstrated that there was a statistically significant MDC1-focussed subset of proteins within the global GPR19 response proteome (Figure S3, Figure 6). When this MDC1-GPR19 intersecting data subset was extracted, we found that upon network-based investigation, this data cohort was able to encapsulate a microcosm of the global GPR19 functionality, i.e., reinforcing the previously described “DDR-oncology-energy metabolism” triumvirate (Figure 7). Therefore, this data suggests that MDC1 is one of the crucial coordinators of the GPR19 aging-associated functionality. In this regard, it has been clearly demonstrated that MDC1 acts as an intrinsic linker between DDR [155], oncology [60], and metabolism [102,156].

Concerning the potential mechanisms by which GPR19 could regulate MDC1 expression, it is possible that both G protein and non-G protein mechanisms may be involved [33,157]. Downstream of these GPR19-initiated signaling start points, several processes could be entrained to control MDC1 expression or functionality. These processes include the stimulation of the ERK (extracellular signal-regulated kinase: [158]) or PI3K (Phosphoinositide 3-kinase: [159])/Akt pathways, which can regulate transcription factors associated with MDC1 expression, such as NFκB or E2F1 [160,161]. Additionally, transcription factor activity could be modulated, such as STAT3 (signal transducer and activator of transcription 3), which has been shown to regulate MDC1 expression through interaction with the ATM-CHEK2 pathways [162]. We have observed that these pathways are important for GPR19 functionality. GPR19 may also be able to regulate MDC1 expression through epigenetic mechanisms, including DNA methylation patterns [163] or histone modifications [164] at the MDC1 promoter, which can affect its accessibility to transcription factors and RNA polymerase.

With our proposal that MDC1 could be a functional linchpin for the aging-associated activity of GPR19, we sought to investigate how other protein subcomplexes may be important for GPR19 activity. We thus investigated the enrichment of CORUM-based microprotein complexes in the global (Figure S4A) and distinct DDF extracts (Figure S4B–D). With this analysis, we discovered that a persistent feature within this data output was the Prohibitin signaling complex. Prohibitin-associated protein complexes have been consistently linked to many of the core functions of GPR19, i.e., stress response [165,166,167], DDR [168,169], oncogenesis [170,171], and energy metabolism [172,173,174,175]. Interestingly, we subsequently discovered that indeed PHB and MDC1 can associate with GPR19 complexes in a manner that is also sensitive to cellular perturbations that mimic aspects of the metabolic aging process. In addition to both the PHB and MDC1 dynamic associations with GPR19, we also assessed the interaction with a binding partner we have previously investigated [6], i.e., EIF4A1. Unlike PHB and MDC1, we did not observe a significant alteration in the association of EIF4A1 with GPR19 immune complexes with oxidative stress, mitochondrial dysfunction, or nutrient starvation (Figure 8A,B). In contrast to EIF4A1, we also found that CBR1 was enriched in GPR19 immunocomplexes and was also significantly elevated in response to the pro-aging cell stressors applied. CBR1 is a short-chain dehydrogenase/reductase that primarily acts as an NADPH-dependent oxidoreductase. CBR1 is critically associated with metabolic functions as it has been shown to inhibit apoptosis and improve the survival of insulin-secreting pancreatic beta cells through its antioxidant activities [176]. Reinforcing the ramifications of this functionality, this protective activity was attenuated in pro-diabetic conditions [168]. In addition to this, CBR1 has also been implicated in DDR activities such as protecting cellular survival via the NRF2 pathway [177], as well as oncogenic activity in breast cancer [178,179]. We next investigated whether a potential functional interaction between PHB and MDC1 may be vital for GPR19 functional activity. We undertook an in silico-operated investigation (without any direct input of any of our empirical GPR19 data) into the physiological relevance of an MDC1-PHB association using empirical protein–protein interaction data (Figure 9). We found that the known MDC1 and PHB interactomes significantly intersect in a biologically relevant manner (Figure 9D–F). This common MDC1-PHB data cohort was able to encapsulate a microcosm of signaling activity highly reminiscent of our characterized GPR19 signaling triumvirate, i.e., “DDR-oncology-energy metabolism” (Figure S5). Our identified MDC1-PHB interactome association prompted us to assess how the proteins that may connect these two factors are present in a tissue-independent mode across the body. Using a network-bridging approach (to in silico link MDC1 and PHB) across 142 curated human tissue databases, we found a significantly enriched network of cell-type independent proteins (21 proteins) that routinely connect MDC1 and PHB (Figure 10). Elegantly reinforcing the molecular relevance of MDC1-PHB to the activity of GPR19, we found that this cohort of in silico-derived proteins interacts with each other to create a network of interconnected signaling functions linked to breast cancer, aging, and DDR (Figure 11A,B). Interestingly, we found that of these 21 cell type-independent proteins that connect MDC1 and PHB, fourteen of them were found in our original GPR19 DEP list (Figure 11C,D).

Collecting together our empirical and in silico data, we contend that GPR19 is potentially crucial to natural stress response management in the aging process in humans and animals. To simply assess how GPR19 may control cellular survival during pro-aging insults, we assessed how GPR19 expression (inducing both constitutive receptor activity as well as assembling crucial stress response complexes [33]) could attenuate stress-induced loss of cell viability. Using an automated high-content cell viability assessment technique, we found that GPR19 expression in our model cell line attenuated the detrimental effects induced by either peroxide-induced oxidative stress or DNA damage (Figure 12). As mitochondrial integrity and functionality are crucial to stress responsiveness, cell viability, and aging [71], we assessed how this specific functionality may be affected by GPR19 activity. To address this, we created a focused mitochondrial functionality dataset (Figure S7A) using a wide variety of database platforms as well as AI-associated text mining. We benchmarked this dataset using a Gene Ontology Biological Process annotation and demonstrated that the gene with the highest enrichment probability (*p* = 1.25 × 10^−42^) was “mitochondrial respiratory chain complex assembly” (GO 0033108). Using this mitochondrially focused dataset, we next extracted the specific GPR19 DEPs that were associated with this dataset. We found fifty-four specifically associated proteins that were common to the GPR19 and mitochondrial datasets. Investigating these DEPs, we found a bi-functional phenotype for the actions of GPR19 on mitochondrial activity (Figure S7B). This is not surprising, as we chose the median GPR19 expression range (2 µg expression) to perform cell viability experiments—this expression level appeared to sit between the levels that detect cell stress and those that appear to exacerbate cell stress (Figure 4).

Hence, concerning the effect of GPR19 on mitochondrial activity, it should be noted that one of the most downregulated proteins was citrate synthase (CS). Reductions of mitochondrial CS can have several effects on cellular metabolism, including reduced energy production and diminished levels of citrate. Citrate synthase catalyzes the formation of citrate from acetyl-CoA and oxaloacetate in the TCA cycle. Decreased citrate levels can have various downstream effects on cellular metabolism, including altered lipid metabolism and impaired gluconeogenesis [180,181]. CS has also been implicated in mitochondrial dynamic processes such as fusion and fission [182]. As with CS, we found that OPA1 (optic atrophy 1) was also strongly downregulated by GPR19 expression. OPA1 plays a crucial role in mitochondrial fusion, cristae organization, and bioenergetics. It is primarily found in the inner mitochondrial membrane and regulates mitochondrial dynamics, cristae architecture, and mitochondrial quality control [183]. Reductions in OPA1 are therefore likely to negatively affect mitochondrial network activity and potential apoptotic activity [184]. Interestingly, at the same time as the potential reductions in OPA1 and CS, there were potent increases noted in SDHA (succinate dehydrogenase complex subunit A), PPIF (peptidyl-prolyl cis-trans isomerase F), and TOMM20 (translocase of the outer mitochondrial membrane 20), all factors that could positively influence mitochondrial functionality [185,186,187]. Thus, it is likely that at differential expression levels, GPR19 can positively or negatively affect mitochondrial activity—this reinforces the importance of employing subtle in cellula experiments (such as ours described here) to investigate the multidimensional signaling activity of GPCRs.

Thus, it appears that GPR19, potentially through the control of coordinated MDC1-PHB functionality, can serve to protect cells against pro-aging stressors. This finding presents GPR19 as a potential novel therapeutic target for the amelioration of the aging process as well as other pathological processes linked to this that may involve significant dysfunctions of DNA damage management, e.g., cancer. It has been shown that increases in GPR19 expression are found in cancer cells of various lineages, e.g., lung [188,189], breast [34], adrenals [132], and pancreas [190]. It is interesting to note that for breast cancer cell lines, GPR19 demonstrates differential expression levels that are associated with differing degrees of metastatic capacity. MDA-MB-436 and HCC1954, are both breast cancer cell lines that have been extensively studied in research. While both cell lines were derived from breast cancer tumors, MDA-MB-436 cells carry a mutation in TP53, while HCC1954 cells contain mutations in both the BRCA1 and TP53 genes. In addition, MDA-MB-436 cells are HER2 (receptor tyrosine-protein kinase erbB-2), while HCC1954 cells are HER2 positive, which is associated with a more aggressive form of breast cancer. Interestingly, MDA-MB-436 cells have been reported to be more resistant to chemotherapy drugs such as paclitaxel and doxorubicin compared to HCC1954 cells [191]. Linked to these aspects, it has been found that GPR19 expression is considerably higher in HCC1954 cells compared to MDA-MB-436 cells ([192]–GEO dataset series GSE1299). Given our current data and our current knowledge about GPR19, it is likely that two different modes of GPR19 therapeutic intervention could emerge. The first mode involves the stimulation of cellular protective pathways associated with lower levels of GPR19 (Figure 4). The second mode involves the potential antagonism of deleterious pathways activated by higher levels of expression. As we have shown that GPR19 may regulate a broad range of potentially therapeutic as well as potentially pathological activities, it is vital that a more holistic view of GPCR functionality be engendered in the realm of therapeutic regulation of these crucial cellular regulators.

## 4. Materials and Methods

### 4.1. Cell Culture, Transfection, and Treatment

Human HEK293 (CRL 1573) cells were obtained from ECACC and propagated at 37 °C with 5% CO_2_ ambient tension, according to approved ECACC culture protocols. HEK293 cells were maintained in Dulbecco’s Modified Eagle Medium (DMEM; Sigma-Aldrich, St. Louis, MO, USA) with 10% fetal bovine serum (FBS)-containing propagation media, supplemented with 1% Penicillin/Streptomycin antibiotics as previously described [6]. One day before transfection, 3 × 10^6^ cells were seeded into 10 cm plates to obtain a 50–80% cell confluence the day of the transfection. Cells were counted using a Luna II automated cell counter (Invitrogen-Life Technologies, Waltham, MA, USA). The cDNAs for a hemagglutinin (3xHA)-N terminally tagged human GPR19 receptor (obtained from OriGene, Rockwille, MD, USA) and an empty plasmid (pcDNA3.1+: Invitrogen-Life Technologies) were transfected into the cells with Lipofectamine^®^3000, using the manufacturers’ instructions. To investigate the effect of differential receptor overexpression on downstream proteins, we transfected the cells with a range of cDNA concentrations (0.5, 1, 2, 5, and 10 μg). To induce oxidative stress, cells were treated with 100 nM hydrogen peroxide (H_2_O_2_/peroxide) for 90 min. DNA damage was caused using 1 μM camptothecin (CPT) for 3, and 24 h, depending on the experiment. The percentage of cell transfection routinely found (employing the simple co-expression of a GFP-containing cDNA construct) was between 75–80% 24 h post-transfection. These estimates were made with manual cell counting following transfection.

### 4.2. Cellular Protein Extraction

For generic low-definition cellular protein extraction, following a described cellular treatment, cells were washed three times with ice-cold PBS and scraped from dishes in the presence of either RIPA 0.1% or 1% SDS supplemented with phosphatase inhibitor cocktails (PhosSTOP, Roche Diagnostics, Basel, Switzerland) and protease inhibitor cocktails (Complete Mini, Roche Diagnostics), dependent on the experiment. To generate differential cell fraction protein extracts, cells were first washed as monolayers with ice-cold PBS and then subjected to a detergent-dependent fractionation process using a Q proteome extraction kit (Qiagen, Hilden, Germany) according to the manufacturer’s instructions. Before eventual analytical use, protein quantification of generated cellular lysates was performed using a standard colorimetric protein assay, i.e., the Bio-Rad RC DCTM assay (Bio-Rad, Hercules, CA, USA).

Cells were lysed 24 h post-transfection, and HA-tagged GPR19 expression was confirmed with a selective Western blot. The optimal time course of expression of the median expression level (i.e., 2 μg) was assessed and found to be optimal at the standard 24 h time period following cDNA transfection. In-depth protein extraction was then made (using three distinct extraction buffers (Q proteome extraction kit) for the cytoplasmic, plasma membrane, and nucleus/organelles) before untargeted proteomic expression analysis was made across the stated expression level series (0.5, 1, 2, 5, and 10 μg expression).

### 4.3. Quantitative Proteomic Analyses

Extracted protein lysate concentrations were determined using the RC DCTM Protein Assay (Bio-Rad). Samples for MS were prepared with the ProteoSpin™ on-column proteolytic digestion kit (Norgen Biotek, Thorold, ON, Canada). A nano-liquid chromatography (LC) column (Dionex ULTIMATE 3000) coupled online to a Q Exactive™-Plus Orbitrap (ThermoScientific, Waltham, MA, USA) was used for the MS analysis. Peptides were loaded onto a 75 μm × 150 mm, 2 μm fused silica C18 capillary column, and mobile phase elution was performed using buffer A (0.05% formic acid in Milli-Q water) and buffer B (0.05% formic acid in 80% acetonitrile/Milli-Q water, Darmstadt, Germany). The peptides were eluted using a gradient from 5% buffer B to 95% buffer B over 120 min at a flow rate of 0.3 μL/min. The LC eluent was directed to an ESI source for Orbitrap analysis. The MS was set to perform data-dependent acquisition in the positive ion mode for a selected mass range of 375–2000 *m*/*z* for quantitative expression difference at the MS1 (140,000 resolution) level, followed by peptide backbone fragmentation normalized collision energy of 28 eV, and identification at the MS2 level (17,500 resolution). Label-free quantification (LFQ) analysis of the spectral outputs was achieved using MaxQuant (https://www.maxquant.org/: accessed on 21 February 2023) [193], a widely used software platform for the analysis of shotgun proteomics data available from the Max Planck Institute of Biochemistry. MaxQuant facilitates the simultaneous identification and quantification of proteins that are differentially up- or down-regulated (at a *p* value of <0.05) in response to GPR19- and metabolism-associated perturbations. The software was connected to an Andromeda search engine: http://www.coxdocs.org/doku.php?id=maxquant:andromeda:start: accessed on 21 February 2023 [194]. Each protein was assigned a confidence score (0% to 100%) based on the confidence scores of its constituent peptides based on unique spectral patterns. Proteins were only identified from the recovery and measurement of one peptide (from MS2) that is identified with a 99% confidence level.

### 4.4. Bioinformatic Analyses

We applied a multidimensional informatics approach to the analysis of our proteomic and interactomic data. To facilitate the specific separation of complex datasets, we employed the Venn diagram platforms, VennPlex, VENNTURE [195,196], and Interactivenn (http://www.interactivenn.net/). The significantly altered proteins of the acquired datasets were functionally annotated using pathways from the Kyoto Encyclopedia of Genes (KEGG: https://www.genome.jp/kegg/), Reactome (https://reactome.org/), WikiPathways (https://www.wikipathways.org/index.php/WikiPathways) or the CORUM PPI (protein–protein interaction) databases (http://mips.helmholtz-muenchen.de/corum/). These pathways or PPI analyses were performed with either Kolmogorov–Smirnov (KS) GSEA (GeneSet Enrichment Analysis) or Hypergeometric Over Representation Analyses (ORA) using the GeneTrail v3.2 suite (https://genetrail.bioinf.uni-sb.de/start.html) [197]. For advanced network-based analysis, we employed the NetworkAnalyst (https://www.networkanalyst.ca/) application, which is designed to serve as a visual analytics platform for comprehensive gene expression profiling and meta-analysis. NetworkAnalyst allows for the creation and eventual informatics interrogation of multiple network types. Multiple types of protein interaction databases are available for interactome enrichment analysis, including the IMEx (International Molecular Exchange Consortium) consortium (http://www.imexconsortium.org/), STRING (https://string-db.org), and the CCSB-associated Rolland Interactome (http://interactome.dfci.harvard.edu/H_sapiens/). To create tissue-specific network associations, the HumanBase application (https://hb.flatironinstitute.org/) was employed. All the aforementioned platforms were accessed on 21 February 2023.

### 4.5. Immunoblots and, Immunoprecipitation

To validate proteomic data, the experiments were replicated and analyzed using immunoblotting with a standard protocol. In short, all samples were separated on 4–12% SDS-PAGE (Life Technologies, Carlsbad, CA, USA), transferred to PVDF membrane (Amersham), and blocked using 5% BLOTTO milk. Primary antibodies for immunoblots: MDC1 (Bethyl), HA-tag (ThermoScientific), ACTB, CBR1 (Sigma Aldrich), EIF4A1 PHB (GeneTex, Irvine, CA, USA). The membrane was then incubated with species-appropriate secondary antibodies conjugated to horseradish peroxidase (HRP). Immune complexes were then identified using enhanced chemiluminescence (ECL, GE Healthcare, Chicago, IL, USA) and an Amersham Imager 680 system. Western blot quantification was performed with GE-ImageQuant TL and Image J software (1.53t), using red Ponceau staining as a loading control or human beta-actin.

### 4.6. Murine Tissue RT-PCR

GIT2KO gene-trap animals [6] based on a standard C57BL/6 background, initially obtained from Duke University (Richard Premont, Durham, NC, USA), were bred at the National Institute on Aging under NIH protocol numbers 432-LCI-2015 and 433-LCI-2015, according to the approval of the Institutional Review Board. All animal studies performed were approved according to the guidelines of the NIA Animal Care and Use Committee. Mice were maintained in a 12 h light/dark cycle on an ad libitum regular diet. The Rneasy Mini Kit (Qiagen) was used for cellular mRNA extraction from multiple tissues derived from wild-type (C57Bl6) and GIT2KO mice. Reverse transcription was performed using proprietary kits (Life Technologies, Carlsbad, CA, USA). Genes were normalized to GAPDH. RT-PCR was performed using the ABI Prism 7300 Sequence Detector (Applied Biosystems, Carlsbad, CA, USA).

### 4.7. Statistical Analyses

In each histogram or figure, the data represent the means ± SEM (standard error of the mean). Statistical analyses (the student’s *t*-test) were performed using GraphPad Prism version 7.0 (GraphPad Software, San Diego, CA, USA). The significance level is indicated in each figure as * *p* ≤ 0.05; ** *p* ≤ 0.01; *** *p* ≤ 0.001.

## Data Availability

All data pertaining to primary experimental result acquisition is made available by the included data Supplementary Tables S1–S4.

## Supplementary Materials

The following supporting information can be downloaded at: https://www.mdpi.com/article/10.3390/ijms24108499/s1.
