# Supplementary material for: GPR19 Coordinates Multiple Molecular Aspects of Stress Responses Associated with the Aging Process
Source: Int J Mol Sci. 2023 May 9;24(10):8499. doi: 10.3390/ijms24108499 (PMC10218176; doi:10.3390/ijms24108499)
Supplement: Supplementary file 1 [file ijms-24-08499-s001.zip › Data-Supplement-Maudsley-et-al-2023-Figure-S2.pdf]

Data Supplement

GPR19 coordinates multiple molecular aspects of stress responses associated with the aging process

Stuart Maudsley [1*], Claudia Schrauwen[1], İrem Harputluoğlu[1], Deborah Walter[1], Hanne Leysen[1], Patricia McDonald[2]

1. Receptor Biology Lab, Department of Biomedical Science, University of Antwerp.
2. H. Lee Moffitt Cancer Center, Tampa, FL, USA

* Corresponding Author: Prof. Stuart Maudsley – stuartmaudsley@uantwerpen.be: receptorbiologylab@gmail.com

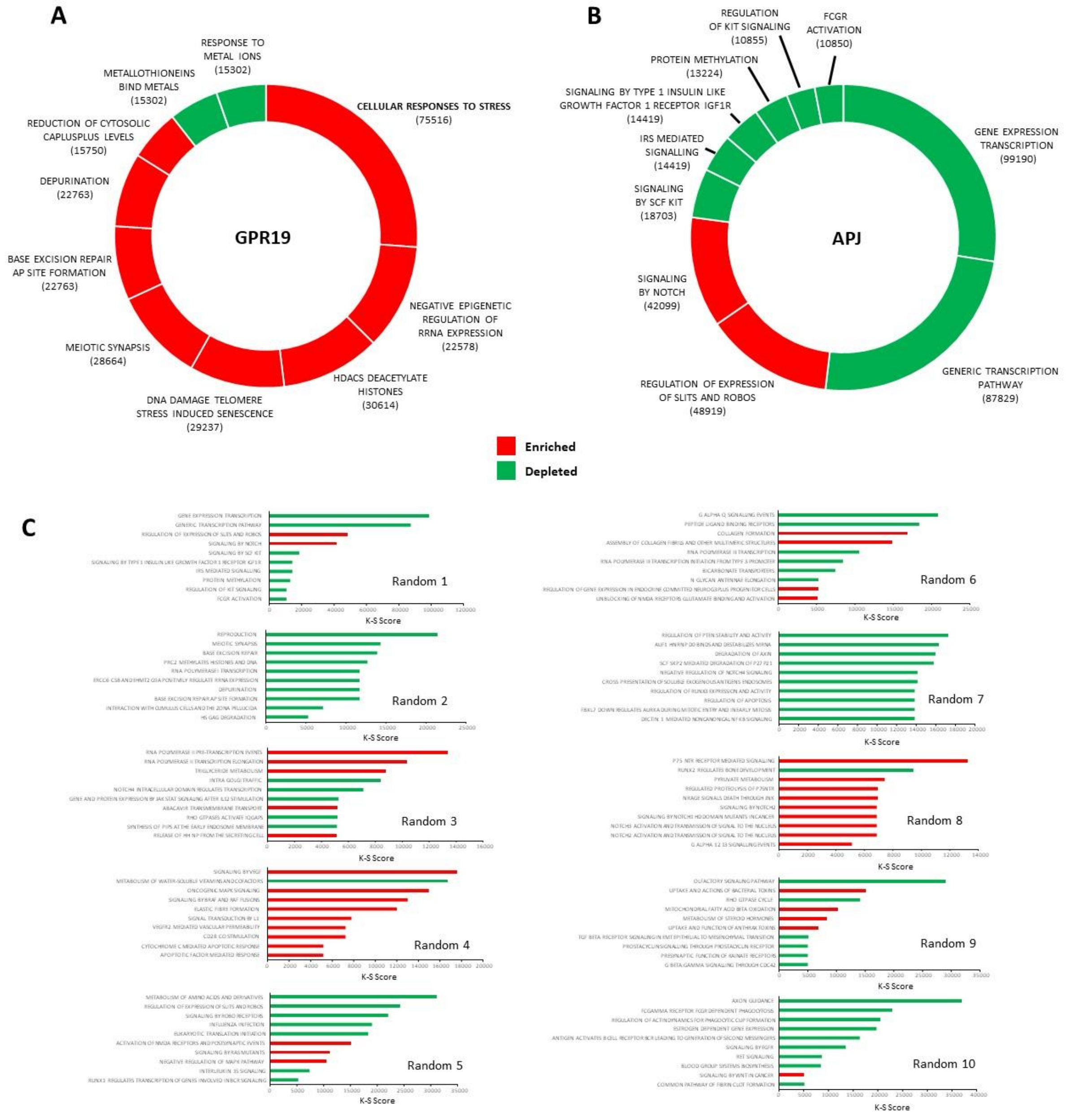

Figure S2. Reactome Pathway analysis of GPR19 perturbagen response data compared to Apelin receptor perturbagen response data and random data. To identify the specificity of the Kolmogorov-Smirnov (K-S) data analysis of the GPR19 perturbagen response data (A) we also performed a similar Reactome pathway analysis using the K-S running-sum score of similarly generated perturbagen data from the human Gαi-coupled Apelin receptor (APJ) (B). The top 10 highest scoring (using the K-S running sum statistic) Reactome pathways for both GPR19 and APJ are indicated in the diagram. 'Enriched' pathways (populated mainly by upregulated proteins) are denoted in red while 'depleted' pathways (populated mainly by downregulated proteins) are denoted in green. From the chart it is evident that the pathway analysis of the GPR19 perturbagen data yields a highly specific response despite the generic similarities between the two Gαi-coupled rhodopsin-like receptors (GPR19, APJ). To further reinforce the specificity of the GPR19 Reactome pathway analysis then randomly-generated datasets (https://molbiotools.com/randomgenesetgenerator.php), the same numerical protein size as the GPR19 dataset, were analyzed for any random pathway enrichment that was similar to that

observed for GPR19. In all of these enriched pathway results the ‘Cellular Response to Stress’ pathway was not significantly enriched (C).
