# Supplementary material for: GPR19 Coordinates Multiple Molecular Aspects of Stress Responses Associated with the Aging Process
Source: Int J Mol Sci. 2023 May 9;24(10):8499. doi: 10.3390/ijms24108499 (PMC10218176; doi:10.3390/ijms24108499)
Supplement: Supplementary file 1 [file ijms-24-08499-s001.zip › Data-Supplement-Maudsley-et-al-2023-Figure-S3.pdf]

Data Supplement

GPR19 coordinates multiple molecular aspects of stress responses associated with the aging process

Stuart Maudsley [1]*, Claudia Schrauwen[1], İrem Harputluoğlu[1], Deborah Walter[1], Hanne Leysen[1], Patricia McDonald[2]

1. Receptor Biology Lab, Department of Biomedical Science, University of Antwerp.
2. H. Lee Moffitt Cancer Center, Tampa, FL, USA

* Corresponding Author: Prof. Stuart Maudsley – stuartmaudsley@uantwerpen.be: receptorbiologylab@gmail.com

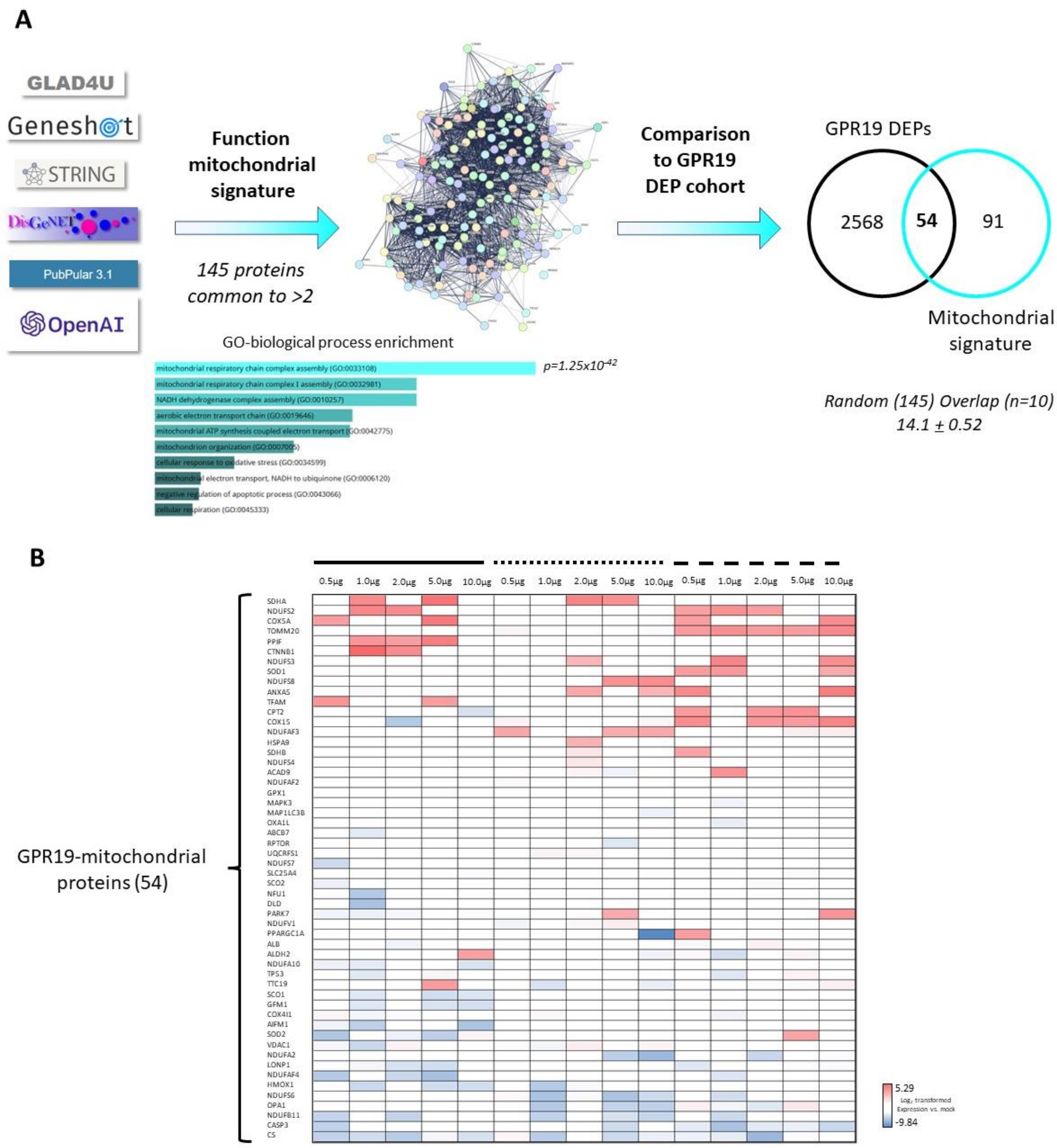

Figure S3. Specific mitochondrially-based investigation of GPR19 functions. A specific mitochondrial molecular signature dataset was created using: GLAD4U (http://glad4u.zhang-lab.org/index.php); GeneShot (https://maayanlab.cloud/geneshot/); STRING (https://string-db.org/); DisGeNet (https://www.disgenet.org/home/); PubPular (https://heart.shinyapps.io/PubPular/); OpenAI (https://chat.openai.com/chat). From these multiple databases a dataset of 145 proteins found in at least two of these databases were chosen to act as a microcosm of mitochondrial function. Gene Ontology biological process analysis of this 145-protein cohort revealed a strong bias towards mitochondrial activity. This 145-protein dataset was then investigated for its intersection with the GPR19 perturbagen response dataset. We found 54 specific proteins common to both that are indicated in the heatmap for all of the GPR19 expression doses in each of the extraction fractions depicted in panel (B). The level of intersection between random datasets (of 145 proteins) with the GPR19 dataset was found to be only 14.1 $\pm$ 0.52 (mean $\pm$ SEM). Cytosolic protein responses are indicated by the unbroken solid line, plasma membrane proteins are indicated by the dotted line and nucleus/large organelle proteins are indicated by the dashed lines. The levels of protein upregulation or downregulation are indicated by the key to the heatmap.
