# Supplementary material for: GPR19 Coordinates Multiple Molecular Aspects of Stress Responses Associated with the Aging Process
Source: Int J Mol Sci. 2023 May 9;24(10):8499. doi: 10.3390/ijms24108499 (PMC10218176; doi:10.3390/ijms24108499)
Supplement: Supplementary file 1 [file ijms-24-08499-s001.zip › Data-Supplement-Maudsley-et-al-2023-Table-S2.pdf]

Data Supplement

GPR19 coordinates multiple molecular aspects of stress responses associated with the aging process

Stuart Maudsley [1*], Claudia Schrauwen[1], İrem Harputluoğlu[1], Deborah Walter[1], Hanne Leysen[1], Patricia McDonald[2]

1. Receptor Biology Lab, Department of Biomedical Science, University of Antwerp.
2. H. Lee Moffitt Cancer Center, Tampa, FL, USA

* Corresponding Author: Prof. Stuart Maudsley – stuartmaudsley@uantwerpen.be: receptorbiologylab@gmail.com

Table S2. GPR19 induced protein expression alterations in the plasma membrane (CE2) compartment. Significantly-regulated proteins (identified by official Gene Symbols) found in cells, differential between mock-transfection and the specific GPR19 expression level (0.5, 1, 2, 5, 10µg), are represented as a log2-transformed and normalized expression ratio between the specific GPR19 expression level and the expression level found in cells receiving empty vector transfection.

Gene Symbol	CE2 0.5	CE2 1	CE2 2	CE2 5	CE2 10
RAD50	8.396166	7.69871	7.083238	6.343929	7.324503
TACC3	5.255857	5.646168	5.230488	6.347601	4.998936
PGM3	5.856697	5.963834	5.085445	5.101395	5.409277
PDAP1	9.222202	8.955604		3.87075	4.442603
DIABLO	5.956536	6.117558	6.896559	6.473151	
COLGALT1	4.255547	4.442226	4.261642	4.248381	4.5388
UBA2	7.754652		6.813613	7.129645	
CLGN		7.256758		6.912912	7.304615
RPS24	5.218748	5.433923		5.209906	5.444566
FARSB	4.224168	4.234982	4.570312	3.002996	4.990068
RTN3	2.830226	4.197738	3.638109	4.835835	4.50803
MDC1	4.897054	3.58098	2.029585	5.436453	3.1684
ETF1	4.890324	4.824556	5.130607	4.003568	
CCDC6	4.36654	2.398018		3.744082	8.004424
DDX19A	5.441245	6.157138			6.622569
NAA10	4.573187	5.639501		3.14981	4.746903
GRB2	4.527323	4.485842	4.436292		4.659344
KIAA1524	2.59011	4.156684	3.005384	4.310237	3.836749
ANP32C	4.219499	4.218817	2.080707	2.471282	4.806223
DCTN1	8.396502		2.100582	3.739887	3.223941
GMPS	3.904269	4.159791	3.007207	2.588598	2.444054
COPS4	3.806237	4.614148		2.947247	4.438288
RIF1	8.395669			7.335939	
AHSA1	4.264408	6.039251		5.175856	
RRP9	3.0736	2.589998	3.621747	2.618888	3.381173
LTA4H	2.815766	2.892392	3.123152	2.605819	3.758297
BAG6	3.386025	4.516488	3.491089	3.374618	
TERF2	3.283414	4.624034	2.906634	3.870694	
NHP2	2.533864	2.560516	2.520298	3.648656	3.400519
PSIP1	7.232805				7.136455
SSU72	3.557537	3.097957	2.600004	2.923984	2.12216
KRI1	4.524278	3.83669		2.507333	3.299088
HN1	3.702839	3.502694	3.077645		3.847221
RANBP3	3.265385	3.449178	2.209416	1.777415	3.281335
ATIC	2.630077	2.766063	3.388891	2.269906	2.89886
TPT1	3.144038	3.478618	2.276382	2.254789	2.753835
PRPSAP2	3.077188	2.422165	2.700893	2.202787	3.381976

HSPB1	8.395398		2.782095	2.552177	
EEA1	2.85673	2.62576	2.973406	2.73763	2.421728
GPI	6.680979	6.906951			
KLF12		3.011002	2.833759	2.274424	5.329515
KLF3		3.011002	2.833759	2.274424	5.329515
KLF5		3.011002	2.833759	2.274424	5.329515
KLF7		3.011002	2.833759	2.274424	5.329515
MYNN		3.011002	2.833759	2.274424	5.329515
ZKSCAN7		3.011002	2.833759	2.274424	5.329515
ZSCAN16		3.011002	2.833759	2.274424	5.329515
ZSCAN2		3.011002	2.833759	2.274424	5.329515
ACTBL2	2.89498		3.911076		6.579158
ABCF2	2.759438	2.598108	2.845067	2.699784	2.391323
LSM14A	8.398797		1.642354	3.232209	
RPS10	1.36594	2.068691	2.635922	4.785406	2.155432
ANP32A	2.807105	2.850172	2.17328	2.199548	2.798892
MGEA5	2.911222	3.078775	1.826328	2.369549	2.559235
PSMA3	2.017553	4.06889	3.505596	3.14588	
PGP	2.84083	2.91989	2.143372	1.975463	2.667839
STMN4	8.396872		1.458049	2.606697	
NSF		4.198847		4.490125	3.667606
REXO4	8.399349	3.744074			
BLVRA	3.132613	3.233212		2.617496	3.08048
HDAC2	8.395493	3.665376			
NPEPPS	3.345038	3.752324	2.212608	2.704853	
CHCHD4		3.589819		4.102385	4.160435
WDR6	2.815243	3.816346	2.16487	2.989692	
MSH6	2.762846	2.957977	2.896963		3.017527
CTTN			2.866922	4.860484	3.817058
CUL4B	3.154114	3.013078		2.628965	2.710734
DDX51				6.285229	5.18558
FLNB	2.663663	2.754046	1.543499	1.975149	2.407077
EIF4A1	2.112437	2.434614	2.537098	1.611386	2.6457
TMSB10	2.358047	3.15616		2.113698	3.636859
ATAD1	8.39645			2.62197	
NUDT5	1.554188	2.607565	1.868897	1.852188	3.08241
CTPS2	5.507572	5.451168			
ASNS	2.708524	3.149188		2.353701	2.572353
EIF3B	2.291517	2.446221		3.424307	2.447734
ESD	2.936422	2.585656		2.271308	2.731271
MTHFD1	2.933236	3.364453	1.966768	2.130307	
DBI	2.585513	2.694352		2.099327	3.007038
SMNDC1	8.398417		1.918355		
YWHAH		3.636871	3.743545	2.875597	
TSPYL1		4.12942	2.117655		4.002969

TOP2B	4.057183	3.388927			2.767654
PSMA2	3.189391	3.625871	3.235108		
DBNL	2.477241	2.689188	2.181023		2.682106
C12orf43		3.361569	3.543783		3.081793
ATF1	2.544336	2.539029	2.654205		2.245269
CREB1	2.544336	2.539029	2.654205		2.245269
CREM	2.544336	2.539029	2.654205		2.245269
TUBB3	8.395337		1.570525		
RGS10	2.78774	2.96248	2.127421	2.082464	
GLO1	2.543639	2.627275		1.79655	2.967073
TKT	3.254846		3.348993	3.296897	
STRAP	2.467938	2.649202	2.640798	2.001073	
ATP1A2			2.322769	4.28073	3.06016
HIST2H2AA3			2.083622	4.715684	2.783803
PFKP	3.171084	3.09114			3.290698
CAD	2.682196	2.644532		1.851236	2.342075
TRMT6	2.405282	2.789246		1.889109	2.337332
EIF4G1		2.235987	1.727318	3.032223	2.271037
NUP50	3.44336	2.277269			3.482992
SART3	2.044567	2.726008	2.262118	2.105084	
ADSL	2.399793	2.613935		1.785176	2.321479
VRK1	2.106812		2.177151	2.355274	2.442467
NMT1	3.506067		2.61793		2.714857
RAB11FIP1		2.641627	3.469086	2.678778	
ANAPC7	2.82991		2.982728		2.908205
ARL3	2.062439	2.208102		1.794842	2.620009
AP1M2		4.23929			4.427896
PSMC3	2.874807	3.243435		2.506897	
GSTO1	3.12412	3.250886		2.190064	
HAT1	2.655587	2.98603			2.920157
IMPDH2	2.106161	2.519863	1.882199		2.026422
RPS19		4.045033			4.455749
CBR1		3.065652	3.284494		2.100458
CBR3		3.065652	3.284494		2.100458
TXNDC12	8.397056				
STX18	8.39644				
WIZ	8.39617				
TOX4	8.395991				
DDX52	8.395844				
FDFT1	8.395719				
RIC8A	8.395663				
PDE9A	8.395463				
FAM21D	8.395418				
RNF14	8.395376				
C5orf24	8.395375				

WDR3	4.357123	2.050935			1.931114
ADNP	2.088494		1.365711	3.057401	1.812595
CPNE3	2.254952	2.291597		1.594939	2.172848
ARHGDIA	1.802579	1.656896	2.485199		2.318728
NAE1	2.288575	2.383351			3.505206
TNPO2	4.066277				4.047242
PFKL	3.055048	3.022286		1.980592	
DYNC1LI1		2.781648	2.636195		2.509732
DYNLRB1		1.400357	2.021666		4.359371
HMGN2			1.925437		5.701539
PAK1		3.583922			4.001088
CUL4A	2.822311	2.557187		2.192953	
BLVRB	2.613873	2.691149			2.246742
RRM1	2.327804	1.518488	1.545126		2.123231
IK			1.837014	1.870852	3.731015
CDC27				3.722954	3.671455
FKBP1A		3.971498	3.326964		
IDH1		3.180389	1.80409		2.286727
SRCAP		3.916234			3.249127
CCDC124	2.154575		2.730384	2.260632	
HMGN1		1.260303	1.615465	4.202282	
RAB5A				4.87823	2.154035
ANXA6		3.524212			3.496774
NSFL1C			2.492627	1.815971	2.67758
SUPT5H		2.606333		2.052731	2.242365
H2AFX		2.344584			4.554693
OSBP	3.503244	3.39253			
NCAPH	2.622689				4.26361
SDHA			3.77281	3.091626	
TBRG4		3.949745	2.882558		
GSTP1	1.831634	1.88716	3.100771		
SMS	2.342579	2.89901		1.570894	
PPAN			3.116608		3.68206
PSME3	1.675126	1.972155	3.035634		
ILK				3.172317	3.458382
PFDN2	2.296509	2.473959		1.773901	
PHPT1	2.115589	2.263457		2.154205	
EEF1D	3.248443	3.264089			
SMN1				4.286629	2.1458
SMN2				4.286629	2.1458
NDUFS8				3.055673	3.338904
PRMT1	2.142419	2.22586	2.010731		
UCK2	1.61479	1.611933	1.854843		1.19442
RPIA	2.937837				3.250943
PIN1	2.208864			1.713509	2.240623

EIF5	1.42314	1.502995	1.609835		1.550721
IKBKAP		2.322042			3.750714
DDB1		4.282996	1.675605		
IWS1	2.580707				3.364823
FERMT2	2.110109	2.277672		1.52274	
RPL3L	2.500626		3.394207		
SMC4			2.669424	1.960001	1.252565
PSMA4	2.328312	2.331153	1.218382		
MCM3	1.542041	1.402712			2.891169
NPEPPSL1		2.245471	1.864875	1.628121	
NASP	1.579377	1.254726	1.565387		1.269363
NAP1L4	2.848959		2.765722		
USP5	2.758499	2.834105			
CIRH1A		2.703142	2.87378		
STAG2			2.264161	3.310964	
NDUFAF3	1.924381			1.6416	1.872009
TXNDC17	2.625698	2.797193			
EIF2S1			1.6592	1.781232	1.970866
CANX			2.048367		3.360125
PSMC2		1.355187	2.211635		1.832703
EIF4G3	2.666532		2.630139		
CIAO1			2.643759	2.580158	
GLYR1			3.314392		1.889903
TPM4	2.154613				3.030962
NPLOC4	2.237215				2.913432
RAB35				5.135311	
DNAJC8	2.775094				2.360196
TBCB	2.823318	2.290626			
AK1	2.498222	2.590154			
DISC1	2.109011	2.937282			
TRAX	2.109011	2.937282			
TSNAX	2.109011	2.937282			
BRIX1			1.222172		3.770992
GLOD4	3.679702		1.309835		
CHD5	2.343682	2.599169			
CDKN2A			2.522094		2.41989
CDKN2B			2.522094		2.41989
HIST2H2AC			2.083622		2.783803
LDHA	2.376643		2.46049		
RGPD1	1.732433	1.693463			1.362749
FLNA					4.705229
AHI1				4.682157	
MAGOHB				4.616539	
HBS1L			1.60252		3.003525
KIF5B	2.305372	2.270194			

TUBA1B	2.256509		2.315176		
TBCE		2.205085			2.339361
COQ9	2.333146			2.18664	
CNN3	2.500506		2.009336		
MSH2	1.411942	1.427824			1.584785
POLR2A	2.577901				1.835639
MTA1	1.622628	1.155342		1.597278	
PPME1				1.582711	2.783411
XPO5				2.242301	2.119539
LCP1			2.250931	2.090113	
PDHX			1.674125		2.664591
NUP153	2.100429				2.220596
TCERG1	1.11115		1.651533		1.556375
MYH14		2.370724		1.920797	
SMC1A	1.250903	1.348833	1.667524		
DDI2	2.648164			1.607718	
DAP					4.253544
BAG2				4.225133	
CLN6				4.162955	
NANS	2.025002				2.132202
GPATCH4	1.146351		2.907607		
ACAT2	2.076233			1.947391	
CIAPIN1	1.517181		2.490515		
DKFZp686E0752			2.100582		1.888466
RBMXL3	2.045071		1.924122		
CDV3					3.942235
DKFZp586I031				3.84153	
TUBA1A	2.337513				1.446373
RBM25			1.927971		1.848004
LMAN2	1.141305	1.104857		1.52613	
PPM1G			2.06745	1.678038	
GTF3C5	3.601875				
PAK2					3.578705
VBP1		1.757927	1.79784		
PYGL	1.804683	1.732025			
DSG1				3.506583	
ADH5		3.501293			
PSMD2	1.142714	1.114172			1.218004
RNF40					3.472232
ZNF346					3.468571
ELMOD2					3.452888
RFC2	1.818761	1.629611			
AKAP8					3.432349
PHKA1					3.425716
FLII	3.42069				

STUB1				3.419912	
ROCK2				3.410404	
ESF1	1.533084				1.866348
IGF2BP2				2.149886	1.234563
HDAC6			1.776288		1.597609
RBM3			1.508934	1.862118	
MAP1S					3.368592
EIF5B		1.418818		1.938088	
OMA1					3.346786
TOM1				3.336384	
PPIE				3.317107	
HN1L	1.739822	1.553986			
EMG1		1.805193			1.483146
SREK1					3.283596
ACADVL					3.273028
CFL1					3.271146
SCRN1				3.221957	
APEX1				3.201266	
TUBB4A			3.200282		
VEZF1					3.180091
CCDC86			1.070258		2.100729
TFCP2			3.154542		
UBP1			3.154542		
MYO1B					3.102331
AASDHPPT				3.088458	
MCL1				3.074545	
ANXA5			1.864747		1.200904
ATP2A3				3.063403	
RGPD2		1.693463			1.362749
PRKAG1				3.030451	
SERPINH1				3.009685	
SMARCB1					3.006108
LDHAL6B			2.990718		
UBE2N					2.980724
PPP1CB				2.95934	
MYH10	1.556372	1.367202			
HNRNPD					2.921809
CENPV		1.195371			1.713292
HMG20B					2.902378
TLN1			1.621591		1.253681
APOBEC3C				2.872783	
CCDC47	2.870649				
TOP1MT	1.105843			1.761203	
MKI67			1.527097		1.311735
RPL18A		1.367772			1.468623

UAP1					2.822279
SEPT9					2.814901
TMEM180		2.813605			
XPO7		1.381979			1.420289
DARS	1.134166		1.663665		
SLC9A3R1			1.582212		1.205664
UBL4A			1.567043		1.190246
SMC2		2.70295			
MORF4L2				2.684399	
QARS	1.311078	1.369517			
C3orf17					2.631293
HLA-B				2.608514	
HLA-C				2.608514	
DHFR					2.528393
CHORDC1					2.486938
POLR2E				2.457537	
DCTPP1					2.404791
HTATSF1			1.271637		1.124999
TUBA1C			2.373789		
ALCAM	2.367477				
GOLIM4					2.353958
RPE				2.347019	
PRPF3				2.341057	
TUBA1A			2.339691		
CPSF3	1.123438	1.207329			
PSMD6		2.317931			
PAFAH1B1		2.310399			
PSMC5		2.306194			
ERCC6L	2.299655				
RPLP0	2.273211				
STIP1	1.106559	1.164233			
SEPT2					2.25728
NIFK	2.255057				
HIST2H3A			2.253183		
PRDX4			2.252693		
QKI			2.247704		
TES	2.245258				
LRPAP1					2.242278
EIF3I				2.226455	
TSN		2.204109			
DDX23					2.202271
TPD52				2.193949	
ACTN1				2.184697	
TCEA1					2.178464
GDI2					2.177502

EIF2B2					2.168518
KIAA1033	2.097702				
ERCC6L					2.089868
PAFAH1B1	2.067439				
ANP32B	2.053316				
TUBA4A			2.042978		
PRPF4B			2.03932		
PSMD12				2.037397	
MDH1	2.024572				
DYNLRB2			2.021666		
MRI1				2.01823	
NUCKS1	2.016247				
CCT3				2.007019	
CBR4				1.989354	
KTI12				1.964778	
SLC7A1				1.939308	
CDK12				1.932448	
NUFIP2				1.920692	
PSMD14	1.901163				
BAZ1B			1.894201		
ANXA7				1.893069	
GALK1				1.890616	
HNRNPUL1			1.889222		
CSNK2A1			1.87473		
LARP1			1.866332		
PRMT8			1.850692		
SNRNP200			1.844081		
RPL35A				1.83632	
RPS16			1.813713		
PARK7				1.811951	
UHRF1				1.806173	
TNPO1				1.778109	
TXNL1				1.764343	
ACTR3				1.761343	
UBBP4			1.758443		
PSMA8			1.756944		
ZC3H13			1.737082		
SETDB1			1.727491		
DHX36			1.697293		
NEDD8					1.692487
DDX39B			1.660506		
PRRC2A				1.633617	
GBAS				1.633366	
ARPC5L			1.632602		
HOXB9				1.62912	

MCM2				1.627222	
HSPH1				1.626328	
CBX8			1.61811		
PPP2R1B			1.616749		
FIP1L1				1.609962	
KIF4A					1.599431
APEH					1.583877
LUC7L				1.581412	
FXR1					1.580384
PRKDC					1.577578
SGTA			1.575902		
CARHSP1			1.57065		
DTYMK			1.566357		
METAP1	1.563878				
CHD3				1.562453	
FAM98A				1.557329	
EIF2A			1.551684		
DNAH17					1.548303
UBE2D3			1.529338		
ME2				1.518048	
SRRM2	1.516944				
EARS2				1.513715	
MAD1L1				1.509956	
POTEKP			1.504923		
NACA			1.50349		
NACAP1			1.50349		
LRRC47		1.488033			
FXR2					1.445578
HINT1			1.408848		
EEF1A1			1.402572		
WIBG					1.392807
RPS10P5		1.374198			
TRMT112					1.364311
AGPAT9					1.364114
HSPA9			1.335724		
PSMA6		1.333539			
PPP2R1A			1.30445		
ALDOC			1.291011		
G3BP1	1.290525				
CKS1B					1.284495
EIF5AL1			1.276704		
RNH1	1.271124				
TIAL1			1.254393		
ACTA1			1.252366		
ACTC1			1.252366		

PDCD6IP			1.23835		
PRPSAP1					1.221887
POLA2			1.216492		
FBL					1.206014
ATP6V1A					1.201298
DCP1A			1.192596		
PDS5B			1.176637		
POLR2F	1.17413				
P4HB			1.167048		
PKLR					1.155769
COTL1	1.148505				
TUBB4B	1.141674				
TAGLN3	1.13987				
NDUFS3			1.139498		
ACTG1			1.134789		
DNAJC9					1.128284
POLR1B	1.125633				
AP1B1					1.125592
RPN2			1.118738		
TUBB			1.116309		
HSPE1			1.111033		
RBM12	1.110149				
TUBA3E			1.110002		
CUL1	1.103107				
CKB			1.099887		
GTF3C1			1.093405		
POLR1A			1.088984		
TRIM28			1.076259		
ZNF280C			1.058779		
DNAJB12			1.041633		
EDC4			1.037882		
CDK4			1.008909		
RPLP2			1.00117		
PPP2CB			-1.00916		
PPP1R14B			-1.01396		
TIMM22			-1.01472		
RBM12B			-1.02965		
MLEC			-1.03495		
RUVBL2			-1.04516		
RB1			-1.06257		
MAZ			-1.07808		
SF3B4			-1.09069		
CCDC51			-1.09908		
UQCRC2					-1.1091
ZNF638		-1.10911			

NDUFB10	-1.11476				
HSPA13	-1.11566				
SPTBN4			-1.128		
BRAT1			-1.12855		
VAT1			-1.14081		
CHCHD1			-1.14543		
CAMSAP3	-1.14555				
LMNB1					-1.14797
GALNT2					-1.15508
MRPS31		-1.1554			
SLC25A13	-1.16242				
SLC25A6	-1.16807				
ACTN4	-1.16988				
STAMBP			-1.17408		
SEC24C	-1.17618				
MTX2	-1.1889				
LSM7			-1.19163		
NKTR			-1.19658		
SNTB2			-1.19795		
PGM2			-1.21145		
NOP58	-1.21251				
ERLIN2		-1.21726			
GSR			-1.22906		
LYZ			-1.23648		
DNTTIP2					-1.24395
LYPLAL1			-1.24517		
COPB1			-1.24529		
NDUFA4	-1.24583				
TMLHE			-1.27025		
RNPS1					-1.2714
SCPEP1			-1.27248		
PYCR1					-1.27453
CCDC88A	-1.27471				
PFDN5			-1.27554		
MMAB	-1.28051				
CGGBP1	-1.28207				
TIMM23			-1.28665		
TIMM23B			-1.28665		
SDHB			-1.28684		
PRSS1		-1.30976			
PRSS2		-1.30976			
PRSS3P2		-1.30976			
EXOSC5			-1.32081		
CWC15					-1.32896
BOP1		-1.33159			

SPG20					-1.33557
WDR43			-1.36443		
DIEXF		-1.37618			
CDC45			-1.40076		
TRO			-1.4076		
PDCD11	-1.41131				
PPP6R1			-1.4252		
FAF2			-1.42749		
PHC2			-1.42766		
MARK3			-1.44908		
MARK4			-1.44908		
NDUFS4			-1.44972		
RAP1GDS1			-1.46996		
EXOSC9					-1.47545
MCCC2		-1.47809			
HCFC1					-1.48259
C19orf52			-1.4936		
ERO1L		-1.49414			
POLR1C				-1.50428	
GCN1L1				-1.51573	
REEP5					-1.5168
G3BP2			-1.52082		
YME1L1			-1.52959		
EXOC3L4			-1.53352		
RGPD5			-1.55343		
XPO6			-1.55413		
ALDH1L2				-1.55708	
CORO1C				-1.56106	
TECR		-1.56485			
PTPN1			-1.57248		
GRWD1				-1.57849	
NOMO2			-1.5839		
NSUN4			-1.60201		
CSNK2A2			-1.61239		
AIG1				-1.63224	
GOSR2			-1.65214		
MYBBP1A					-1.65342
SRPRB			-1.68789		
USP14				-1.6973	
ABHD10			-1.69968		
CSTF2					-1.7048
SUCLG2		-1.73871			
SRRM1				-1.74111	
FAR1					-1.74231
HIST1H3A				-1.75195	

ETFA			-1.7546		
TTC7A			-1.75771		
REEP5			-1.76584		
DDX6			-1.7732		
ICT1			-1.82779		
DECR1			-1.83704		
ARF3			-1.85763		
SRRT			-1.86454		
RGPD3			-1.87203		
RGPD4			-1.87203		
AHCTF1			-1.9028		
ACTR2			-1.92913		
PPP6R3			-1.94344		
BCL2L10			-1.94839		
SPTBN2			-1.95511		
SNRPF			-1.95716		
ATP5O			-1.95751		
RPL27				-1.96694	
PDIA3				-1.97012	
LYRM7			-1.98578		
ACAT1				-1.99768	
GATAD2A	-2.00115				
UBE2O	-2.0075				
MRPS9			-2.01295		
PAIP1			-2.01615		
VARS					-2.02383
COMMD6	-2.03996				
CAMK2D	-2.04093				
FANCI	-2.04489				
COIL			-2.05517		
FKBP4			-2.06061		
UTP20			-2.08717		
THOC6			-2.08785		
INIP	-2.09532				
HIST3H3				-2.1374	
NPM3			-2.13896		
TMPO					-2.14128
MAN2B1			-2.14592		
TMEM205					-2.15592
LPCAT1	-2.16116				
POLR1E					-2.16166
PAK1IP1		-2.16245			
TMED7					-2.16247
TMED7-TICAM2					-2.16247
DCBLD2			-2.17121		

FHL1			-2.18492		
CSTF3		-2.18621			
SSR4					-2.19183
NDUFA3					-2.19232
PDS5A				-2.19316	
COX5B		-2.20824			
KIF16B			-2.21275		
KIF3A			-2.21275		
KIF6			-2.21275		
RABL6	-2.22525				
NIP7			-2.22542		
NDUFS1		-2.23322			
CNIH4		-2.24852			
GHITM		-2.25257			
NFKBIL1	-2.25674				
DLG1					-2.25945
GPT2		-2.26076			
PREB			-2.27082		
MIA3		-2.27298			
C12orf73					-2.29409
COX4I1		-2.31342			
HSPA4L			-2.31391		
GK				-2.31966	
GK3P				-2.31966	
ALDH5A1		-2.32574			
KRTCAP2					-2.3308
SYNJ2BP	-2.33112				
FAM114A2					-2.33745
GIGYF2				-2.3379	
NDUFAF2	-2.35618				
NOP16			-2.35907		
KHDRBS3	-2.3595				
ASMTL	-2.36187				
C7orf55-LUC7L2	-2.37358				
SUPT6H				-2.37395	
ACOT7				-2.37895	
ATP6V1B1		-2.37952			
BPNT1			-2.38095		
PSMB3				-2.38387	
MANBAL					-2.38513
AP3M1				-2.39502	
PTCD3			-2.40615		
HLA-A					-2.42323
LAMP2		-2.42551			

BUD31					-2.44734
NCAPD3			-2.45663		
DES	-1.21598		-1.24522		
NOL11	-2.47084				
PRPH	-1.30613		-1.17451		
H2AFV	-2.48235				
UTP11L	-2.48239				
PHB	-1.37903	-1.10556			
SLC33A1		-2.51406			
TIMM10B		-2.52139			
TMOD3			-2.527		
MKLN1			-2.53646		
AGPS					-2.55315
SYNJ2BP-COX16		-2.55547			
PSAP					-2.56886
RGPD8			-2.57272		
SSR1		-2.57662			
OSBPL8			-2.58392		
CNOT1			-2.58404		
MRPL45		-2.58432			
ATP5SL	-1.35189				-1.24605
ATP5J		-2.62367			
WBP11					-2.62712
ELAVL1		-2.62984			
MRPL22	-2.65908				
GNA11			-2.68151		
GNA14			-2.68151		
GNAQ			-2.68151		
MRPS25			-1.56416		-1.12077
SLC39A9					-2.69056
SLC25A12	-1.17503		-1.53207		
CTCF	-2.71075				
GNG5	-1.4157				-1.30281
PPIAL4A	-2.72575				
SRSF11	-2.75083				
ATP5A1		-1.24159		-1.5355	
HACL1			-2.78187		
UBQLN1				-1.72742	-1.12263
MRPS7		-2.90146			
DDT			-2.90901		
DDTL			-2.90901		
NIPSNAP1			-1.11274		-1.81062
MRPL45	-2.96006				
NUP88				-2.96026	
CTR9			-2.97888		

SPIN1					-2.99229
AGK	-1.17916		-1.8191		
VDAC3	-1.51585		-1.49345		
NUP93		-3.01937			
AMOT				-3.02075	
TARS2					-3.02639
LOC102724023					-3.0301
GEMIN6					-3.03303
DACH2					-3.03316
ERAL1	-1.66199				-1.37161
SUCLA2					-3.03472
ATG3				-3.03867	
CHTOP			-1.83287		-1.20788
NDUFS5					-3.05744
GADD45GIP1			-1.74697		-1.31996
MRPS2					-3.06916
EPRS					-3.07368
UTP18					-3.07788
EIF1AX				-3.07791	
RPL6					-3.07829
NT5C3B					-3.08342
KRT5				-3.09786	
KARS				-3.11038	
HSPBP1					-3.11635
TRAFD1					-3.11694
PPP2R2A					-3.11802
PPP2R2C					-3.11802
RPS28				-3.11829	
S100A13				-3.11925	
UROD					-3.12246
MYH1					-3.12378
MYH13					-3.12378
MYH2					-3.12378
CEP170			-3.12517		
RAB18					-3.12607
ANKLE2				-3.13059	
YIPF5					-3.13226
LIN7B					-3.13237
MRPL49			-1.60293	-1.53817	
U2AF2					-3.14343
CLASP2					-3.15945
HSP90B2P					-3.16336
SEC23B				-3.16532	
ALDH2					-3.17587
BCAP31			-1.72292		-1.45334

ZNF330				-3.17876	
BLMH			-1.73566		-1.44336
TMEM41B					-3.1829
DACH1					-3.18506
MAP1LC3B					-3.18523
MAP1LC3B2					-3.18523
HIST2H3PS2					-3.19092
SNX18				-3.19827	
RSU1					-3.21191
NCAPD2					-3.21349
SLC25A10			-1.8962		-1.32037
OGFR					-3.22563
FAF1				-3.2377	
SRP19	-1.67919	-1.55882			
BAG3					-3.24052
WBP2					-3.24064
RELA					-3.24188
YWHAE				-3.2432	
GLG1	-1.64478	-1.60339			
GNB2L1		-1.2349		-2.01328	
MRPS6	-1.58098	-1.69447			
MRPL1					-3.28206
MRPS17				-2.03559	-1.24748
ALDH16A1					-3.28612
SDF4				-3.30313	
QPCTL				-3.30868	
KIAA1429					-3.30881
RPS5					-3.3094
ITGB1				-3.33129	
NDUFA13					-3.34779
UFD1L					-3.35071
HSP90B1					-3.36368
HIST1H2AA			-3.36735		
HIST2H2AB			-3.36735		
GMPR2					-3.36912
RQCD1					-3.37351
NUP214		-1.14842		-2.22845	
CWF19L1				-3.39796	
S100A11				-3.40122	
SPCS2				-3.41001	
TFIP11				-3.41337	
LRRFIP1				-3.41549	
SMPD4					-3.4164
CAST					-3.43104
RPS26P11					-3.43205

CCAR1	-1.81673	-1.61559			
SNX6					-3.43502
DDX3X					-3.43704
RPS26					-3.44696
MRPS26				-3.45324	
PSMD13				-3.46632	
POLR3C				-3.46868	
KRT16				-3.47708	
SRSF2					-3.48141
ACYP1					-3.48797
STAU1					-3.4887
SNRPB					-3.5004
SNRPN					-3.5004
WARS2					-3.50336
CDK5RAP3					-3.50359
ZPR1					-3.50776
ZNF24					-3.5127
TMX1					-3.52259
SRSF8					-3.55126
CSTF2T			-3.555		
DYNC1I2				-3.56886	
RBBP6			-3.59617		
UFL1					-3.62016
ZNF326	-1.97816	-1.64671			
DIS3		-1.90975		-1.72691	
XRCC1			-1.1455	-2.49524	
HOXA13					-3.64491
HOXC13					-3.64491
TXLNG				-3.66183	
ACADM				-2.56152	-1.11827
MLLT4	-1.41889	-1.13276			-1.13229
HECTD3					-3.72409
CLTB					-3.73507
HIBADH				-3.74184	
PPP4C					-3.78508
RPS6KA3					-3.79231
SURF4	-2.70421		-1.09424		
TRIP12			-2.17492	-1.6313	
EIF2AK2					-3.80849
TFIP11					-3.81579
PPFIA1				-3.82195	
CALU					-3.82231
CNBP			-2.31735	-1.51214	
CYB5B	-2.13833			-1.69774	
NME4				-3.85053	

RPTOR				-3.85209	
XPO1				-3.86861	
ARL8B				-3.89402	
ADO				-3.90859	
SUMF2			-2.25588		-1.65465
PGRMC2		-2.21803		-1.70362	
PARN				-3.93113	
SYNCRIP	-2.19762	-1.743			
PDHB			-1.05296	-2.89142	
UBR7				-3.95849	
BLM					-3.98184
RECQL					-3.98184
PPM1A		-4.01172			
PPM1B		-4.01172			
EZH2			-4.01851		
H2AFZ	-2.48235		-1.54095		
CPOX			-4.03028		
MAP4				-4.03182	
MEAF6			-4.03853		
EXOC4				-4.05024	
POLR2C				-4.06338	
CIRBP	-1.68602		-2.3889		
LIN28B					-4.07615
C4orf27					-4.10092
FASN					-4.12602
GNG12				-4.15533	
CALR					-4.15868
KIAA0101			-4.17962		
EEF1E1					-4.18144
NDUFC2					-4.18403
PRPS2					-4.18426
PDHA1	-2.21009		-1.98701		
SCAF8				-4.19766	
PPA2	-1.49032	-1.38529			-1.32398
ACAA1	-1.47217	-1.15649	-1.57531		
TMA16		-4.22697			
TALDO1		-4.23275			
CCDC43		-4.24178			
TBL3	-1.54275			-2.70846	
TRUB1				-4.2708	
HSP90AA2P					-4.27412
CYB5A		-2.33338	-1.9424		
RPL39			-4.28311		
RPL39P5			-4.28311		
RPL36				-4.2835	

ANKFY1				-4.29324	
NDUFA8		-2.33454		-1.9783	
ARHGAP17					-4.31711
ELP2			-4.32408		
LBR				-4.33215	
SMARCD1				-4.34978	
OXSM	-2.39439			-1.95754	
UQCR10	-2.00223	-2.35803			
SLC25A11	-1.86443	-1.10765			-1.39476
UQCRFS1		-2.07789	-2.29524		
CHRAC1	-1.27144	-1.74736	-1.36014		
COX15	-2.11758				-2.26192
FKBP8			-1.01069		-3.37283
TSFM				-4.38379	
VTA1	-2.055	-2.33322			
HDAC3				-4.38967	
RPL9		-2.27986			-2.13205
AMOTL2					-4.42853
TRUB1		-4.43838			
MAGED2	-2.06713	-2.3916			
TCEB2					-4.46867
U2AF1		-4.47581			
PHF3			-2.11051		-2.38098
ZFR				-4.4941	
LRBA		-4.51033			
SH3BGRL3				-4.52144	
AGAP3				-4.52213	
NFKB1					-4.53294
JUP			-1.78991		-2.75553
SFXN1					-4.54743
XPNPEP1					-4.55798
SF3B1					-4.57216
CASP3				-4.57589	
RPS29					-4.57615
U2AF1L4			-2.777		-1.80043
ABCD3		-2.26008	-2.32592		
PLAA					-4.59461
YWHAB				-4.5992	
TIMM13				-4.60816	
TBC1D15				-4.60841	
CEBPZ		-3.38003	-1.25068		
DCAF7		-2.92246		-1.71666	
KRT76				-4.64438	
CTBP1				-4.64995	
SDE2			-4.65022		

NOL7				-4.66555	
GMPPA				-4.66765	
NT5DC1				-4.67206	
DDX18	-1.51537	-1.37748		-1.79523	
EIF2B4				-4.70733	
ERP29		-1.11872		-3.59091	
PDE12	-2.15025	-2.56032			
THUMPD3				-4.71389	
KRT26				-4.71562	
MAEA				-4.72551	
PPAN-P2RY11				-4.7386	
SRA1				-4.76655	
TOMM20	-2.21888				-2.55522
PNO1				-4.78687	
MCM5	-2.79547			-2.01653	
VHL			-4.81411		
AP3S1				-4.81824	
POLDIP3	-2.49825				-2.32863
TMEM33				-4.82711	
TPM2				-4.82843	
EIF3F	-1.62669	-1.40272		-1.80361	
HNRNPUL2-BSCL2		-1.13266		-2.33118	-1.37867
CD81	-2.13584	-2.71173			
NNT		-2.23606			-2.61283
USP39				-4.84904	
RBM19		-4.85682			
ASPH	-2.24851	-2.63497			
AHSG				-4.88612	
TOR1AIP1				-4.8882	
USP34	-2.82085		-2.08015		
PROSC				-4.90439	
RCN2	-2.22305				-2.73821
GGCT				-4.96207	
RBM14	-2.04076	-2.92151			
NSA2			-4.9708		
NVL			-4.97567		
PSMC6		-4.99497			
RTN1	-3.29104			-1.70514	
DNAJC7			-1.8517	-3.18239	
DHX29				-5.04615	
TAF15	-1.11609	-1.711		-2.22342	
BAX				-5.05475	
RABL3				-5.05571	
SLC25A31	-1.3977				-3.70341
HNRNPL	-2.15736				-2.96078

CHMP2A				-5.12912	
GARS		-5.1418			
PRKCSH				-3.88253	-1.26171
RALY		-1.28507		-1.75179	-2.13179
MT-CO2	-2.41993	-2.77347			
NDUFV2	-1.26997	-1.36095		-2.57023	
PEF1		-5.20455			
TPT1P8		-5.22209			
KRT13				-5.25169	
ACAD9			-2.15766	-3.10984	
RAVER1					-5.30869
NAA40					-5.33189
TARDBP	-1.19714				-4.18102
TMED2					-5.38251
SAFB	-2.16012	-1.3745		-1.85451	
TMEM33		-5.39322			
CPD		-2.70313	-2.70265		
TRUB1					-5.41943
TAGAP				-5.42428	
MAP2K3					-5.43039
MAP2K6					-5.43039
SH3BGRL3		-5.44046			
COPS3		-5.48223			
BCL2L13		-3.32675		-2.17674	
ATAD3A	-1.60541	-1.15037	-1.52031		-1.24343
ATP6V0C	-2.73912	-2.78425			
BCAT2			-1.80275	-1.56661	-2.16978
GEMIN4		-5.54959			
ARID1A		-5.56581			
HNRNPH1					-5.59638
THOC2		-5.59912			
BCKDHA		-5.63313			
TMEM9		-5.68152			
TMEM9B		-5.68152			
PLIN3		-1.11877	-1.985	-2.60175	
RRP8	-1.70817				-4.00527
CD99		-5.71952			
U2SURP	-2.10316			-3.62931	
RPS25	-5.80139				
RUVBL1			-2.66818	-1.8059	-1.33291
ATAD5			-5.81575		
CD59				-2.14399	-3.67455
EHMT1			-5.84813		
MT1X		-5.85808			
MT2A		-5.85808			

NUP205			-3.55359		-2.33282
TRA2B		-5.89274			
KLC2			-2.66411		-3.23637
HSPD1		-2.79805		-3.12388	
HAX1		-2.69389			-3.2492
DDX47		-2.62691			-3.32607
SMARCA4			-2.73311	-3.22152	
C2orf47	-5.96365				
DHX40				-6.00532	
KDM2A				-6.05091	
RAP2A	-2.07935	-4.01827			
RAP2B	-2.07935	-4.01827			
RAP2C	-2.07935	-4.01827			
STRBP				-3.01574	-3.1323
NOL6	-2.55517			-3.61345	
PYCR2			-1.55106	-4.66974	
TMEM230				-3.00098	-3.23006
NOP2			-1.54071	-1.62757	-3.07817
TIMM50			-1.08509		-5.17657
MRPL48	-2.13757		-1.9629		-2.27709
HMGCS1		-1.30097		-1.8795	-3.21101
TMEM261		-4.09466	-2.30723		
COX7A2L	-2.06724	-2.24255			-2.1101
RSL1D1				-3.17901	-3.25101
TXN2		-2.421	-1.84366	-2.16848	
IPO5				-3.14492	-3.30814
FAM21A				-3.17259	-3.29602
FAM21B				-3.17259	-3.29602
FAM21C				-3.17259	-3.29602
COX7A2	-2.07222	-2.21869			-2.20402
RFC5		-5.41493	-1.10643		
NIT1			-1.2565	-5.35202	
NOSIP				-3.5572	-3.06229
MTCH2		-1.34063	-1.57513	-2.35446	-1.38127
STARD9				-3.06675	-3.61982
FOXK2				-3.53532	-3.1841
CSTF1				-3.15913	-3.56433
NAT10	-2.25839		-1.93023	-2.54465	
DHRS7	-2.28325			-2.20695	-2.26981
SPAG9				-3.06006	-3.71783
PTBP1	-3.70981			-3.09116	
NELFE				-3.10898	-3.69972
PAWR				-3.30653	-3.54287
RPL34				-3.53235	-3.33301
EXOC8				-3.3176	-3.55322

LTV1				-3.31905	-3.55864
KHDRBS2	-2.10822			-4.77704	
SUMO2				-3.14314	-3.74358
WFS1				-3.12814	-3.78196
MECP2					-6.92443
COA3				-3.80588	-3.15464
DDX39A				-3.01825	-3.94828
ASH1L			-2.69874	-4.29071	
MDN1				-3.63795	-3.36677
RFC4				-3.41543	-3.62315
MAGT1				-3.27053	-3.77396
PHIP					-7.05966
ESYT2				-3.42465	-3.64985
CMC2	-2.21818	-2.38715			-2.47959
CNN2				-3.18052	-3.95121
PTMA	-1.395	-5.75057			
WDR36		-2.84368		-4.30302	
LMNB2	-2.59598	-2.65637		-1.89723	
SLC4A1AP				-3.00956	-4.1833
RHOA				-3.13484	-4.07863
RHOC				-3.13484	-4.07863
TPX2	-3.08298	-4.16594			
NDUFV1	-2.99511		-2.43244	-1.84207	
YWHAG				-3.39118	-3.87865
INTS7				-4.07894	-3.1932
AP2B1				-3.24324	-4.07281
CLNS1A				-3.11551	-4.20326
SLC7A5				-3.17012	-4.14879
UQCRH	-1.45192	-1.17514		-4.70018	
RAB25				-3.17212	-4.15641
RRBP1				-3.56851	-3.77629
TBCA			-2.27609		-5.09262
IDH3A				-3.30479	-4.07024
NDUFAB1			-2.67535	-2.03737	-2.66465
UQCRB	-2.30159	-2.38262			-2.69701
NCDN				-3.49103	-3.89492
PTMS				-3.7107	-3.70088
FKBP10			-3.55708	-2.18462	-1.69113
AIP				-3.45806	-3.97713
PPP1R12A		-5.73357			-1.75707
RPL23				-3.24746	-4.25723
WDR44				-3.24214	-4.26653
CSNK1E				-3.12889	-4.39302
RBM17	-2.19874	-2.2254			-3.10311
RPS4X				-3.34056	-4.19123

RPL29				-3.28229	-4.2508
TIA1				-3.12812	-4.4088
KIAA0415				-3.64867	-3.89251
ATP6AP2	-2.33239	-2.67079			-2.53825
MT-ATP6				-2.29688	-5.24547
WDR41		-4.086		-3.47256	
GINS2				-3.3788	-4.20023
UQCRHL		-2.44412		-5.14707	
TTC19		-4.16135			-3.4374
ITPA				-3.45766	-4.16155
ABHD14B				-3.10897	-4.52472
WNK1				-3.36277	-4.29584
SLC25A3	-1.82749	-1.57369	-4.26902		
SAMM50	-2.2448	-2.26097	-1.07063		-2.17178
MTX1				-3.68037	-4.07373
CKAP2				-3.47532	-4.27892
PAF1		-4.00318			-3.78314
PRPF31				-3.39862	-4.41143
LIMA1			-1.84207	-3.71416	-2.28907
MCM4	-2.25471	-5.59441			
AP3B2		-4.5168			-3.34889
HRNR				-3.05084	-4.81883
CD2BP2				-3.43418	-4.45231
HNRNPK				-4.12255	-3.77508
RBM26				-4.73399	-3.16759
ILF3		-1.70511		-4.70543	-1.49148
PDCD5	-2.12104	-5.8223			
GTPBP6				-3.39885	-4.54457
PLCG1		-7.96511			
TRIP6		-4.24489		-3.7342	
AKR1B15				-3.45482	-4.53834
SLAIN1				-3.95419	-4.09425
PEX19				-3.47768	-4.5816
PTTG1IP				-3.8285	-4.23669
CDC37			-2.66621		-5.42974
GPR180		-4.13587			-3.96937
YWHAZ				-4.42053	-3.69965
FARSA			-4.02699	-4.1994	
GNL2		-4.31173		-3.93002	
PSMD4				-4.61839	-3.62627
LDHAL6A		-8.25561			
MT1G	-2.40236	-5.85808			
MAP1B		-4.05975			-4.20494
DNAJC10		-4.30739		-3.96035	
DDX27	-3.1148		-3.22569	-1.92927	

LSM4		-5.25875			-3.01484
RAB11A				-5.04698	-3.23679
EIF3K		-3.00265		-2.74588	-2.55473
H3F3C	-2.75761			-2.36342	-3.19092
RABGGTB				-3.53706	-4.7778
SRP9				-4.74538	-3.58857
NKAPL		-4.88678		-3.46807	
PLAA		-4.31177		-4.07584	
PTGES2				-3.66506	-4.72322
YWHAQ				-4.08177	-4.35623
HMOX1		-5.7123	-2.73206		
HIST1H1C			-2.40476	-6.08997	
COX6C	-2.81842		-1.71594		-3.96451
CKAP5				-3.74204	-4.76417
GUK1		-4.49596			-4.06162
SBDS		-4.84746		-3.72805	
AMOTL1		-4.14956			-4.42853
ANXA2		-4.839		-3.74508	
BTF3		-5.63142		-2.96992	
CBS	-1.63566	-5.75762			-1.23532
COX6A1	-3.98046	-4.65076			
AGO1				-8.65215	
AGO2				-8.65215	
SRSF7			-1.13101	-3.43668	-4.08921
PDIA4				-3.93264	-4.74989
SLC25A1	-1.50294	-5.60842	-1.60133		
CISD2		-2.35429	-2.07909		-4.31148
GART				-5.25238	-3.51233
MAP2K2		-4.05906			-4.71988
GINS1		-4.85955		-3.95648	
PUM1				-4.8133	-4.01103
PUM2				-4.8133	-4.01103
TROVE2		-4.14842			-4.68103
CYC1	-2.15197	-2.23469		-1.92864	-2.52988
TMUB1		-5.05682		-3.82673	
CHEK1		-4.51662		-4.38117	
MRPS14			-2.12948	-3.51832	-3.25495
C1orf131		-4.53457			-4.39226
CDK9		-4.87376		-4.0882	
IST1		-5.13033		-3.8535	
CCDC40			-4.82984		-4.16978
GLMN				-5.04418	-3.97501
HP1BP3			-3.14241	-5.88943	
SAP18	-3.49771		-2.18656		-3.35724
RPL8		-4.84678		-4.20286	

GTF3C2		-4.60952		-4.45098	
H3F3A	-2.12328	-2.23795		-4.71127	
H3F3B	-2.12328	-2.23795		-4.71127	
SYNGR1		-5.02074		-4.07627	
OGT		-4.92156	-4.24194		
BRD4		-5.02508	-4.14031		
RPS15				-4.86189	-4.30683
NEFH	-2.87052	-4.17303			-2.16106
NEFL	-2.87052	-4.17303			-2.16106
TPM3		-4.39601			-4.82147
HBA1	-2.50671	-2.53685		-1.56821	-2.62687
SUB1		-5.57142			-3.69733
VDAC2	-2.30841	-2.82183	-1.81102		-2.38939
RANBP9				-4.73833	-4.60493
RRAS2				-4.17827	-5.17082
DKFZp434B156		-4.63979			-4.71204
WDR92		-4.63979			-4.71204
SEC62				-4.84079	-4.55023
VDAC1	-2.53362	-2.92457	-1.78329		-2.15886
ST13				-4.78378	-4.63474
SOAT1		-3.49965	-3.48529		-2.46681
RUFY2		-4.60552		-4.88068	
GPATCH11					-9.49448
RBM39		-4.49496	-3.17059	-1.83783	
LMNA	-3.79581		-5.72582		
RAC2		-5.57203		-3.96436	
RHOJ		-5.57203		-3.96436	
RHOQ		-5.57203		-3.96436	
CEP41		-5.141			-4.42394
YLPM1	-2.45473	-5.40533			-1.71539
SF3A2		-4.4419			-5.13753
CTBP2				-4.49793	-5.16507
PPHLN1		-5.10481			-4.57532
HMGA2		-3.65848	-6.02335		
TNPO3					-9.70358
HBA2	-2.72749	-2.92646		-1.61603	-2.4367
TRIAP1		-5.35211		-4.35707	
RPS27L		-5.29828		-4.42754	
ARL2BP		-4.7356			-4.99929
HM13		-5.05068			-4.69261
MGST3	-3.14725	-2.9052		-1.57709	-2.11466
DNAJB2		-4.84711			-4.9008
DNAJB8		-4.84711			-4.9008
DNAJC11	-3.65274	-3.00881			-3.1818
PPARGC1A					-9.84385

CLTA		-5.30729		-4.54192	
HGS		-5.25833		-4.5978	
SF3B3				-4.62915	-5.25897
EPS15L1		-2.87605	-7.09056		
PES1		-5.55959		-4.42919	
CRYZL1				-4.83561	-5.18759
SKIV2L2				-4.69759	-5.35572
MEPCE		-5.42665		-4.63997	
TFRC		-5.57094		-4.49902	
RBM28				-3.00508	-7.07548
NUP98		-5.01518		-5.07406	
KPNB1	-3.16513			-3.0591	-3.90285
FSCN1		-5.6395		-4.49059	
DDX4		-4.80487			-5.36272
MRPS28		-5.12944			-5.08914
LSM8				-4.97028	-5.25816
CPSF7		-5.69817		-4.5391	
RAB1C	-5.91716		-2.5459	-1.79511	
NCL		-5.16809			-5.13221
STX7		-5.65907		-4.68951	
MT1E		-5.65408		-4.70997	
MT1M		-5.65408		-4.70997	
CDCA8			-5.2425		-5.13705
CHAMP1	-2.23695		-2.9058	-2.21022	-3.02848
WDR18		-5.24793			-5.16225
RRP12	-2.08871	-4.06382			-4.26062
GNG5		-5.57425		-4.84613	
FAM175B				-5.1112	-5.32811
SNW1		-5.64928		-4.80456	
PLD3		-5.40644			-5.06644
RAB11B				-5.04698	-5.45634
DDX56			-5.34119	-5.17294	
WHSC1		-5.16936			-5.36385
VASP		-5.70242		-4.8337	
RNF2	-2.33388	-2.36074		-1.98143	-3.86897
EIF4G2		-5.48206			-5.08561
MRPL17			-1.97804	-3.95169	-4.64126
HMGN4			-2.72288	-7.87682	
MTDH	-1.90429			-3.49204	-5.22578
MOV10		-5.68855		-4.93964	
UBE2Z		-5.697		-4.94406	
RPL36AL		-5.67877		-4.96381	
39692		-5.68145		-4.96534	
UBE2M		-4.54843	-1.93107	-4.1678	
EDC3		-5.75713		-4.90637	

COX6B1		-5.9008		-4.83895	
SLC1A5		-5.35869			-5.40056
BZW1		-5.44741			-5.33136
RBMX		-5.53896			-5.2477
COX7C		-2.76849	-2.31884	-1.65038	-4.06832
DLST	-2.48366	-2.4251	-2.5143		-3.39986
TCOF1	-1.45164	-4.49974			-4.93249
POLB		-7.79241	-3.1356		
SNRPC		-5.67795			-5.37456
MRPS18B	-2.90244	-2.86728		-2.89248	-2.41458
SUMO3		-4.06265		-3.18767	-3.89006
UQCRQ	-2.11449		-4.67424	-1.56468	-2.81289
GAA		-4.06222		-3.24698	-3.85976
HK1		-4.09056		-3.54464	-3.55586
SUGP2		-4.41057		-3.24987	-3.54935
RBX1		-5.77827			-5.44682
RPL28		-4.23497		-3.82166	-3.18338
ZMYND8		-7.02508	-4.37559		
RPL31		-4.06908		-3.75689	-3.62684
HADHB		-4.07579		-3.30736	-4.07987
PELP1		-4.07796		-3.23482	-4.15884
RPS11		-4.09635		-3.86837	-3.59124
ARF4		-4.09117		-3.37664	-4.0977
TEX264		-5.50303		-4.673	-1.51501
SON			-1.91385	-4.93533	-4.85748
RAB1B			-2.13808	-5.01685	-4.60889
CHP1		-5.28051		-3.95732	-2.56045
FAH		-5.1045		-3.01662	-3.75371
DDX54		-4.23121		-3.12502	-4.52775
NOLC1	-1.35961	-5.73305		-4.85054	
RPL36A		-4.42927		-4.15136	-3.36456
P3H1		-4.0658		-3.24516	-4.64703
LEPRE1		-4.0658		-3.49117	-4.41562
APIP		-5.75006			-6.28807
DPP9		-4.628		-3.3206	-4.09365
PPIH		-4.06963		-4.36541	-3.63771
COASY		-4.06602		-3.46803	-4.55141
MARCKS		-4.3254		-3.57386	-4.22823
NIN		-4.08223		-3.45684	-4.63058
SUCLG1		-4.25366		-3.38215	-4.55791
NDUFA2				-5.37595	-6.82106
SAR1B		-4.30769		-3.1988	-4.73889
ATXN2L		-5.6015		-4.98815	-1.71639
MPP6		-4.22852		-3.31354	-4.81562
REXO2		-4.14807		-3.63487	-4.6216

SEC31A			-7.1201		-5.3122
TSPAN6	-3.15139	-3.43666	-2.16084		-3.84898
MINOS1	-2.92028	-3.00144	-1.87482	-1.84714	-2.97948
UTS2		-4.74526		-3.31108	-4.57745
TOMM40	-3.06733	-2.23684	-4.87165		-2.46254
NDRG3		-6.44112	-6.21141		
RPS23		-5.02267		-4.46723	-3.22179
TIMM9	-2.7743	-3.23198	-1.96532	-1.54536	-3.19724
ATP5D		-4.71738		-3.42953	-4.59229
RCN1		-5.86473	-5.18567	-1.7513	
SRPK3		-4.3332		-3.73565	-4.73814
POR		-4.53556		-3.72029	-4.60571
LDHC		-4.32451		-3.8309	-4.71956
SEC63		-4.76043		-3.75306	-4.40971
BRD2	-2.34625	-2.28683	-3.44393	-1.57751	-3.31565
EIF3G		-4.03649		-4.68852	-4.31772
GSTM2		-4.6047		-3.66107	-4.8046
GSTM5		-4.6047		-3.66107	-4.8046
PCBP2		-4.24451		-4.34613	-4.57209
RPL10	-2.01409	-5.72739			-5.44023
FAM136A	-2.05619	-4.64206	-1.89956		-4.64167
STIM2		-4.89143		-3.65666	-4.77094
GIPC1		-4.5397		-3.82047	-4.97017
DHCR7		-4.39681		-4.15341	-4.79363
LAMP1		-4.47683		-4.07177	-4.80425
AKAP12		-4.41787		-4.0751	-4.91019
DEK		-5.78112	-1.94338	-1.8031	-3.89237
GFAP	-2.64665	-4.53738	-3.99645	-2.26319	
C11orf98		-4.16106		-4.73401	-4.58209
CUL3		-4.38649		-4.38975	-4.7468
RPL37A		-4.49149		-4.30362	-4.73411
GNAI2		-5.05678		-3.81338	-4.69627
MECR		-4.9728		-3.75859	-4.87063
OXCT1		-4.63161		-4.13724	-4.86413
SEC61A1		-4.66178		-4.28688	-4.71282
SEC61A2		-4.72953		-4.28688	-4.76385
CAP1		-5.38433		-5.23839	-3.20845
UGDH		-5.01554		-3.7477	-5.08656
SDAD1		-4.25271		-5.14734	-4.45345
PTBP2				-7.02535	-6.8366
IDH2		-4.95027		-4.18505	-4.72868
TOMM22	-2.28308	-4.30146	-2.08185		-5.20308
ATXN2		-4.52649		-4.69733	-4.66636
EIF4B		-4.7178		-4.38881	-4.78759
PNPT1		-5.0439		-4.17426	-4.70661

CHMP1A		-4.42856		-4.70928	-4.82755
SNRPD2		-4.05667		-5.22175	-4.72206
MMS19		-4.49643		-4.49933	-5.03348
CDC42		-4.92876		-4.28839	-4.86137
HARS2		-5.03531		-4.04506	-5.09817
MRPL12	-2.04634	-5.88883	-1.18183	-5.06965	
CLPTM1		-4.89544		-4.36164	-4.96857
WARS		-4.75253		-4.6263	-4.86475
ARFIP2		-5.10755		-4.2102	-4.95206
RPA2		-4.81456		-4.22974	-5.32955
CHMP2B		-5.13502	-2.03458	-3.13415	-4.08104
NDUFB11		-5.20045		-4.18187	-5.01165
EPHA1	-6.26831			-4.0743	-4.06859
DPM3		-5.25123		-4.27561	-4.89013
SLC35A4		-5.3384		-4.1907	-5.00722
RPL37AP8		-5.22795		-4.16979	-5.14358
FTH1		-5.22908		-4.45327	-4.86123
RDX		-4.14441	-1.64209	-4.38709	-4.40888
PICALM		-5.05316		-4.56811	-4.96402
ARPC3		-5.09544		-4.36831	-5.15753
DDX3Y		-5.08661		-4.54167	-4.99445
HCFC2		-5.26044		-4.33127	-5.08168
PTGES3		-5.30283		-4.39515	-5.07585
REEP5		-5.83261		-5.19482	-3.75053
RBM34	-3.8921	-3.50372	-2.50876	-2.1255	-2.74909
MATR3		-5.32108		-4.42417	-5.07043
UBE2C		-5.14944		-4.42236	-5.25073
PSMB7		-4.96915		-4.41089	-5.45651
HNRNPM		-4.70853	-2.98092	-3.54394	-3.60499
BCLAF1		-4.0649	-2.94167	-3.36038	-4.48002
UFM1		-5.19118		-4.40005	-5.2638
NEMF		-5.28942		-4.66339	-4.98257
IQGAP3		-5.52992		-4.20857	-5.21442
RPL13		-5.27446		-4.83607	-4.87123
DPYSL5		-5.27055		-4.44352	-5.27812
TARS		-5.45412		-4.40651	-5.16896
HGH1		-5.43261		-4.53145	-5.07159
M6PR	-6.04555		-3.98995	-5.0373	
GNB3		-5.3995		-4.58742	-5.11406
GNB4		-5.3995		-4.58742	-5.11406
RAD23A		-5.33987		-4.46506	-5.29901
40057		-5.46499		-4.50708	-5.14838
IMPDH1		-5.45426		-4.47254	-5.21175
EXOSC4		-5.42979		-4.48563	-5.23036
ATP1A1	-2.524	-5.66697	-1.60389		-5.41985

CAPZA2		-5.48623		-4.60675	-5.21904
MRPS5		-5.56579		-4.61271	-5.15872
UBL5		-5.33386		-4.69828	-5.3341
NPM1		-5.08287	-2.22782	-3.04395	-5.05716
ACTR3B		-5.7271		-4.39891	-5.32881
COMT		-5.71595		-4.57047	-5.18447
HDGFRP2	-5.26517	-5.70614		-4.50539	
SNRNP70		-5.57603		-4.68392	-5.22716
CKMT1A	-2.3101	-5.85969	-1.84		-5.50847
CKMT1B	-2.3101	-5.85969	-1.84		-5.50847
ADRM1		-5.44569		-4.69544	-5.391
RBBP4		-5.48494		-4.79432	-5.27444
RBBP7		-5.48494		-4.79432	-5.27444
CKMT2	-2.3101	-5.85969	-1.89499		-5.50847
SACM1L		-5.55736		-4.73477	-5.32369
DENR		-5.63939		-5.10305	-4.89386
RPL30		-5.54595		-4.76832	-5.32568
MRPL43		-5.66423		-4.75568	-5.23463
SNRPA		-5.56998		-4.84685	-5.24271
DDX46		-5.62514		-4.77444	-5.27114
SP100		-5.49745		-4.88202	-5.29697
YTHDF1		-5.5768		-4.77046	-5.37453
NUDT4		-5.64325		-4.72062	-5.37497
CS	-2.24014	-5.52664		-4.70282	-3.32916
SPTLC1		-5.56894		-4.85376	-5.38499
NEFM	-2.87052	-3.50362		-4.50024	-4.94739
STAU2		-5.60862		-4.83702	-5.37716
ARF5		-5.65143		-4.79241	-5.38654
PSMA1		-5.546		-4.84956	-5.43485
C14orf166		-5.6902		-4.79525	-5.35687
TRAP1		-5.62501		-4.88721	-5.33105
USP47		-5.63435		-4.90651	-5.32244
EIF4A3		-5.60138		-4.8479	-5.51706
ME3		-5.64892		-4.92704	-5.39613
RPS3		-5.70902		-4.85466	-5.43766
SNRPF		-5.73926		-5.14189	-5.33137
OPA1		-5.7994		-5.16309	-5.28601
TCEA2		-5.79077		-5.05073	-5.49063
PRPF40A	-1.68373	-5.18964		-4.61975	-5.10291
CD63	-3.44268	-3.69317	-3.23669	-2.8397	-3.47028
SF1		-5.22209	-2.19975	-4.36768	-5.07027
NOMO1		-5.51891	-1.09195	-5.13443	-5.27709
PRRC2C	-1.56179			-7.61845	-8.41453
PHB2	-1.86453	-5.74685		-4.81701	-5.25121
UQCRFS1P1		-5.66671	-2.21142	-4.53859	-5.26609

DBN1	-1.28424	-5.11847	-2.24577	-4.05272	-5.01118
RUFY1		-4.60552	-1.68794	-4.88068	-6.5686
RUFY3		-4.60552	-1.68794	-4.88068	-6.5686
PLRG1		-5.75876	-1.86596	-4.91366	-5.24453
JAGN1	-1.59523	-5.78354		-4.99844	-5.42537
ATPIF1	-2.46938	-5.39197		-4.93296	-5.06111
TSC2		-4.64575	-3.29363	-4.72018	-5.23535
DKFZp434B2017		-4.30769	-5.75457	-3.1988	-4.73889
EIF3H	-1.49807	-5.56097	-5.60409		-5.36799
PNN	-2.17213	-5.66261		-4.92362	-5.37639
GLS	-3.29643	-2.92437	-3.29424	-4.94899	-3.70304
IMMT	-1.36714	-5.81508	-1.31111	-5.04069	-5.14899
YY2	-2.58352	-5.71604		-4.96666	-5.42228
NME1		-5.17076	-5.52859	-3.06628	-5.12523
YY1	-2.96481	-5.7204		-5.05237	-5.42446
CNNM3		-5.70257	-3.45691	-4.35879	-5.87534
SETBP1	-4.63812	-4.44592	-2.86359	-2.62184	-5.06662
ERH	-1.20376	-5.72022	-3.42412	-4.36445	-5.29725
EBP		-9.37221		-8.51453	-2.17
TLE4			-6.4333	-6.94597	-7.27674
SMARCE1		-4.75258	-4.76505	-5.00203	-6.13952
SLC25A24	-9.53643	-5.10331		-4.55124	-2.16841
NDUFS6	-2.35716	-6.13723	-2.40031	-6.16306	-4.64045
TJP1	-9.28902	-1.67118	-1.93635	-5.88508	-4.65555
NUMA1		-6.06183	-3.38665	-6.61923	-7.56077
CBX1	-9.43251	-4.51135	-3.71741	-3.66025	-2.40244
THOC5	-4.52908	-4.64561	-5.09969	-4.86277	-4.91247
