# Supplementary material for: GPR19 Coordinates Multiple Molecular Aspects of Stress Responses Associated with the Aging Process
Source: Int J Mol Sci. 2023 May 9;24(10):8499. doi: 10.3390/ijms24108499 (PMC10218176; doi:10.3390/ijms24108499)
Supplement: Supplementary file 1 [file ijms-24-08499-s001.zip › Data-Supplement-Maudsley-et-al-2023-Table-S3.pdf]

Data Supplement

GPR19 coordinates multiple molecular aspects of stress responses associated with the aging process

Stuart Maudsley [1]*, Claudia Schrauwen[1], İrem Harputluoğlu[1], Deborah Walter[1], Hanne Leysen[1], Patricia McDonald[2]

1. Receptor Biology Lab, Department of Biomedical Science, University of Antwerp.
2. H. Lee Moffitt Cancer Center, Tampa, FL, USA

* Corresponding Author: Prof. Stuart Maudsley – stuartmaudsley@uantwerpen.be: receptorbiologylab@gmail.com

Table S3. GPR19 induced protein expression alterations in the nucleus/organelle compartment (CE3). Significantly-regulated proteins (identified by official Gene Symbols) found in cells, differential between mock-transfection and the specific GPR19 expression level (0.5, 1, 2, 5, 10μg), are represented as a log2-transformed and normalized expression ratio between the specific GPR19 expression level and the expression level found in cells receiving empty vector transfection.

Gene Symbol	CE3 0.5	CE3 1	CE3 2	CE3 5	CE3 10
TUBB4A		8.242553		9.351084	9.720522
GATC	4.763431	5.843191	5.230535	5.95175	5.139965
MTHFD1L	5.03443	5.13742	5.644408	5.359593	5.318951
CCDC47	6.64904	5.46689	2.513803	3.958372	4.654893
LIMA1	3.630153	5.926437	4.433989	4.187908	4.322675
MCM3	5.15333	5.730416	4.740339	4.30103	2.251686
EIF5	4.252595	4.66208	4.334015	4.067817	4.837141
RALA	2.673472	5.385117	3.319995	4.480454	5.551128
CBR1	4.910196	4.824799	3.654328	3.62262	3.660259
GDI2	4.133909	5.174894	3.27964	2.9253	5.060906
SRPK1	3.509229	4.74124	3.938798	4.00468	4.173394
ERLIN1	4.741597	3.575653	4.040991	4.015641	3.899485
LTA4H	7.371242		5.524087	4.151017	3.152365
HIST1H1B	5.153372		4.245155	6.479236	4.268037
CBR3	4.652119	4.7428	3.038877	3.437425	3.440067
ANXA6	3.739491	4.35529	3.968379	3.240796	3.430652
FHOD1	3.254352	3.08163	3.575376	3.525703	4.761413
RBM12	3.282013	3.917967	3.733188	3.135187	4.114262
PPM1G	3.614445	3.266735	2.987266	3.633269	4.47674
LYZ	4.050207	7.279965	2.903863		3.640887
HNRNPUL1	3.309534	3.296412	4.089483	3.557651	3.60201
TMED10		5.20399	5.339811	2.053773	5.199549
BASP1	3.070783	4.488964	2.960233	2.693557	4.387876
TPI1	4.536716	3.228897	3.653853	2.26586	3.692306
APEX1	2.423478	3.811718	2.763255	3.836632	4.454563
SRI	3.159661	3.461419	3.887171	3.301778	3.45776
HMGB1P1	2.663372	4.085604	2.788801	3.244377	4.444233
NANS	3.985236	4.016078	3.777602	1.69122	3.484526
PIN4	3.67267	3.855606	2.676915	2.657228	4.092039
ANP32C	2.171014	5.602177	2.783149	2.810144	3.047979
TMCO1	3.686299	3.68318	3.757187	5.152776	
TIMM9	2.999216	5.312369	2.925118		5.002195
GSTP1	3.802009	3.149388	3.483972	2.692793	3.048374
NUDT5	4.856675	4.268794	3.339577		3.395648
GARS	3.973393		3.990592	4.839776	3.031165
SFXN1	3.357896	3.876133	2.544774	2.179812	3.869542
CAND1	3.160578	3.135602	6.565578	2.742497	

ANXA2	2.308101	3.659746	2.700853	2.887266	3.966553
CBS	1.792039			6.011221	7.48202
SNRPD1	2.776892	3.352057	2.792276	2.74869	3.319625
TERF2	1.623507	4.378224	2.412714	3.111162	3.357781
DENR	2.589578	3.110343	3.158672	2.206059	3.798974
PDCD5	2.711168	3.370536	2.413948	2.796229	3.505163
NOP10	2.060649	4.05616	1.938515	3.142824	3.405095
KCTD12	1.901425	3.771931	2.719482	2.728295	3.420602
FKBP8	4.383028	3.488797	4.267455	2.382108	
TOMM20	2.550239	3.245288	2.597236	2.452746	3.519299
SLC25A13	3.23801	3.382695	2.205765	2.339206	3.181024
IQGAP1	2.123156	3.953605	2.301094	5.605098	
CHORDC1	3.321023	3.302629	2.795494	2.221963	2.316037
NSF	2.024865	4.62273	3.971449		3.331813
NNT	4.259772	5.234429	4.391215		
EDF1	2.401201	3.337021	2.380935	2.264412	3.1695
PRDX6	2.872932	4.016294	3.05323		3.566438
RPS16	3.218792	3.35739		3.196021	3.648465
NUCKS1		4.290982	2.29826	2.650762	3.97859
PSMD7	1.545583	2.870938		5.040378	3.569159
ILF3	2.052741	3.563401	2.248815	2.551532	2.465893
AP1B1	2.388375		3.026677	3.517103	3.935037
PEBP1	3.273944	3.194521	2.476725		3.862371
NAP1L4	4.52622		3.119953	5.121954	
STMN2	2.481092	3.505233		2.732641	3.946908
CDC37	3.770923	3.077037	2.712773	3.074254	
SUB1	2.300934	3.356013	3.479484		3.439905
ADSL	2.517647	2.556399	2.701169	2.399824	2.316947
RANBP1	4.90823	2.528558		1.937098	3.090749
COX15	3.338216		2.732697	2.510183	3.813923
SMARCE1				5.413995	6.936602
MTA1	2.548527			4.226531	5.446669
HPRT1	2.003431	3.622363		2.835459	3.756227
PKN2	2.880802		3.065371	3.08492	3.176683
UQCRFS1P1	2.627822		3.050946	2.397009	4.094705
PABPC1	3.376748	3.159149	2.579701	2.9656	
ABCF2	5.811469		6.260582		
NIP7	2.100753	4.442407		2.195083	3.189934
PSMB1	3.184728	3.152704	2.929735	2.612459	
DDX42	1.614998	3.018263	2.059933	2.264568	2.871579
RBM26	2.15901		3.001943	3.444488	3.144235
TPD52	2.30848			4.505419	4.80179
CDKN2A	3.119843	3.60611		2.449068	2.404401
DUT	2.43142	3.879403		1.67067	3.485811
AKR1B1	3.419202	3.869911			4.167174

UCHL1	2.431931	4.250201			4.773927
CSDE1	4.636607	3.033983	3.770317		
ZYX	3.877549			3.453583	3.900944
NUDC	2.978481	2.655575	3.02655	2.557965	
SKP1	2.783513		2.763657	3.245967	2.423788
STARD13	2.630626		2.984814	3.636994	1.929293
SDF4	2.490026		2.78236	3.415768	2.317722
RAB6A		3.447894		2.750617	4.736682
TDRKH	2.751986		2.527383	2.715188	2.820129
GBAS	2.236065			2.268151	6.241393
DSG1		5.979821		2.708999	2.045148
RAB6B		3.447894		2.633126	4.602091
NMT1	1.932869	4.575278	2.209692	1.927036	
FHL1	3.325759	3.400726	3.884692		
CRNKL1	3.096827		2.352975	3.093123	2.05168
USP34	1.735766	3.563102	3.163682	2.11946	
GPS1		7.063828	3.512519		
NCBP1	2.839423		2.479855	2.659017	2.522733
EXOC3L4	2.37739		3.484739	2.351166	2.213082
MPHOSPH6	2.446221		2.333557	2.775983	2.753292
RBM17	2.205135		2.812746	2.703947	2.584915
P4HB	1.518037	3.485762		3.073572	2.195795
ACTR2	2.33097	2.907762	3.106009		1.908381
TIMM13			2.693907	3.364056	4.161816
PFDN2	2.941764	2.868864		2.044244	2.33969
TMCO1				3.699889	6.363362
HMOX2			3.779731	4.239977	2.036748
PDIA3	1.999607		2.751754	2.838457	2.446141
FAM96B	2.238979		2.466421	2.812071	2.501653
ACSL3			2.114419	4.521291	3.28862
SCAF4	2.225364		2.292259	2.153716	3.239632
SRSF7	1.578827			3.826176	4.460825
ALCAM	1.761659	3.420197	2.247822		2.417631
CALM1	2.243569		2.37897	2.954063	2.069066
SAFB	2.388361		2.178701	2.131884	2.907055
CAP1	2.493121		2.35188	2.716527	1.979327
GART	3.024337		2.43422	2.121082	1.915489
DYNLL1	2.417682	3.007906	2.003058		2.007872
SSBP1	1.532669	3.209003		2.393223	2.25948
GNB2L1	2.348412	2.589572	2.593452		1.783433
AKR7A2		4.006895	2.149501	3.14305	
RBM4	2.161582		2.63853	2.561265	1.856834
ALDH1B1	2.727469			3.086537	3.368817
LDHA	2.273628	2.898453		2.15447	1.798486
GSTO1		3.005323		2.537045	3.541855

PSMA5	1.957143			4.056486	3.056311
UQCRQ	3.153111		3.371798	2.510616	
HN1L	2.666766	2.430175		1.88859	2.036972
OCIAD1		3.147393		2.458012	3.410539
PGRMC2	2.008998		2.229268	2.424186	2.334954
DDX18	4.590466		4.310728		
CSTB	2.761996		2.598097	1.623387	1.839498
TIMM10B	2.58872		3.096328	3.107261	
CA2	2.870022		2.295799	1.833711	1.718721
AFG3L2		4.464799			4.228582
TMA16	2.495131		1.950916	2.109972	2.115403
APP	2.310159			2.624403	3.665709
SRCAP	2.436201		2.518214	3.549004	
RBBP4			2.181494	3.446305	2.845
PLS3	1.780727	2.573552	2.46333	1.569557	
PPP1R12A		3.297794	2.374605	2.699813	
CPT2	2.606142		2.640206	3.010849	
TNPO1	2.641196			2.069971	3.500907
MAT2A	1.858518	2.523125		1.753231	2.075498
SNX27			2.795569	2.711297	2.685207
SMARCB1	1.645532		1.956902	2.015545	2.532361
PSMD1	2.787046		2.086426	3.123025	
ETF1	2.917977	3.157897		1.902054	
ZRANB2		3.343242	1.927306	2.635135	
PACSIN3	2.356966		2.507978	3.03769	
YWHAH	2.089552	3.409867	2.333325		
GRHPR		3.392176		2.097906	2.341322
PSMA8	2.054712	3.637178			2.127227
PPA2				3.95158	3.854381
HINT1	2.84078			2.270888	2.674395
NDUFS2	2.26182	3.080232	2.427733		
CPSF6	1.998553	3.034406	2.714343		
HDGFRP2	2.535347		2.21542	2.987821	
MYL12A		2.558908	2.996801		2.007677
MYL9		2.558908	2.996801		2.007677
ANXA5	3.476821				4.081206
UGGT1				3.693257	3.788686
PDHA1		3.938837			3.524289
EPB41L2		3.167772	2.057228	2.234527	
HDAC2		3.140277		2.097403	2.15282
NDUFS5		4.017659			3.362489
SPTLC1	2.486707		2.520369	2.338684	
SLC25A24	3.6445		3.690906		
WDR5		2.575757	2.991702		1.747565
BPTF				3.211157	4.071586

TMEM201	2.151654		2.131159		2.998632
UBA2	4.251832			2.939574	
EIF4H	2.794319	2.41855		1.973526	
EHD4	2.489073	2.69431		1.984306	
SNRNP70	1.826642		3.11858	2.169871	
MTX1	2.394999			2.656181	2.045594
NDUFV2		3.557369		3.500068	
HMGN5	2.602247			2.295152	2.120885
SOD1	2.48908	2.681171			1.767386
SERPINB6	1.701367		2.574172	2.623402	
THOP1	1.83052	3.0669	1.994517		
CNPY2	1.692812			2.535902	2.658029
NDUFS3		3.634004			3.24777
KHSRP	2.337543		2.334367	2.18284	
RGPD8			2.790729	1.993185	2.044556
LUC7L	2.14825			2.646635	2.012103
FAM192A	2.19678		2.101662	2.502472	
NIP30	2.19678		2.101662	2.502472	
FDXR		3.204603			3.569308
DIABLO	3.470922			3.162607	
CTSD	2.764534		2.184228	1.633458	
PDXK		2.768878		3.813	
ENOPH1		3.275681		3.281413	
ADH5		4.438559			2.107903
ATP6V1A	2.616283		3.914687		
BOLA2B	1.949802		2.531919		2.030146
PSMD2	2.575998			1.913183	1.991732
HSPH1	1.861151	2.481301		2.136592	
ESYT1	6.467632				
ANXA11	2.758876			3.679571	
PSMC4	3.62412		2.79761		
MRPL54	1.979787			2.264255	2.176214
DBNL	1.992865	2.598789		1.799021	
COX17		4.138176		2.176945	
NENF		4.063449	2.228286		
HTATSF1				3.434256	2.841531
ERO1L	2.105464		1.902549	2.165832	
TFG		3.504833	2.649044		
ANP32B	2.161234	2.40423		1.576709	
NEDD8	1.710424		2.308731		2.099346
ARHGDIA	2.916074	3.189264			
NACA	2.736439	3.312098			
NACAP1	2.736439	3.312098			
HMGN1				3.839661	2.196214
SMC4	2.875315				3.15906

GNL3L	1.872913			2.058355	2.092061
AP2B1	2.388375			3.517103	
RPS10				1.902229	3.988693
RPL36A				1.932089	3.95545
ATP6V1G1	1.704632			2.084276	2.070331
PHF10		3.305897		2.50985	
YME1L1	2.878041		2.931416		
PSMC2	1.933995			1.554068	2.315774
COX5A	2.388357				3.343177
RAB10	1.929802			1.841796	1.939974
ACTN1	2.743049		2.909647		
PRRC2C	2.79258			2.843893	
SDF2L1			3.044193	2.573121	
MRPL43	2.253248				3.299464
WARS2	1.617425			1.919252	2.003444
PSMA3	2.130522			1.587251	1.821469
C7orf50				2.674377	2.84751
ANXA7				2.082532	3.42387
CHP1			2.296386	3.203726	
MBD3				5.476899	
CCDC51			2.237144		3.233808
ALDOA	3.534905		1.90478		
ALDH3A2			2.556438	2.881385	
MT1G				1.866686	3.544116
MT1X				1.866686	3.544116
MT2A				1.866686	3.544116
ANP32A		3.16617		2.236798	
STRAP	3.161106		2.228816		
ACAT2				2.202422	3.170913
ARL6IP1				2.28348	3.061888
RPP30		3.162947		2.175497	
MRPL28	2.89524	2.435261			
SOAT1		3.393369	1.908336		
PSMD3		2.901293		2.399148	
CDK12				1.934796	3.358473
MTHFD1	2.035142	3.256585			
TMEM109				1.583293	3.660171
TIMM44	2.288551			2.954102	
HMGB1			2.053238		3.185158
AASDHPPT		3.698679		1.525075	
PLAA	3.183263		2.019315		
HOGA1		5.126284			
MTAP				2.186789	2.92956
RRM1	2.889854			2.177119	
GLA	1.70232	3.359821			

TPM1	2.048929	3.011784			
WAC		3.087276			1.966984
IK	2.841029				2.203195
PABPC4	1.94145	3.067632			
CAPNS1	2.211067		2.743208		
RPL6	2.4604		2.489208		
ENAH			2.181123	2.764129	
GOLGB1			2.234753	2.696459	
MAPRE1	1.791286		3.121137		
LDHC	1.794485				3.10659
LDHAL6A	1.789159				3.10659
POLR2C	2.819616				2.066271
DNMT1			2.593665	2.288293	
RPSA	2.881093		1.988933		
RPSAP58	2.881093		1.988933		
DHX16	2.101774				2.768185
PFDN1	2.42461	2.441221			
CALR	1.913297			2.936833	
MGST3				2.011989	2.80992
PRMT1				2.329166	2.443066
PTPN11				2.256195	2.514812
FAM136A	2.424383			2.335816	
HIST1H2AA	2.666481				2.092809
HIST2H2AB	2.666481				2.092809
HSD17B4			2.48396	2.26284	
MYH14		2.91099		1.83238	
GTF2B	1.651819	3.085004			
HSP90AA2P	2.035183			2.69993	
DYNLL2				1.914272	2.783023
MRPL48	2.150361		2.539726		
SRRT	1.979584		2.709848		
ME2				2.436454	2.221229
RPS15A				-2.02694	6.675799
COPS4	1.990373		2.657296		
ZC3H15			2.757377	1.855163	
TOR1AIP2	2.459627		2.117487		
ACTN3	2.252729		2.320214		
IDH3G				2.446435	2.121665
CBX3	2.15162				2.327079
EPHX1	2.42765			2.040366	
UQCRH	2.010752		2.449987		
RPS6			2.411445	2.04335	
DSP	4.434354				
SLC25A12	2.405652		2.026509		
PSPC1			2.215189	2.182588	

GNAI2	2.314332			2.079175	
PAFAH1B1	2.375067			2.002867	
LAGE3	2.554402			1.78	
PHKA1		4.328953			
CHMP2B	2.384917			1.910879	
DNAJC10	2.077288		2.212304		
SF3B5				1.882338	2.406396
PPP4C	1.822557			2.451841	
PGK1	2.342255				1.91809
ASF1A		4.260295			
SMAP1	2.648268			1.562437	
ATP5I	2.209109				1.989552
DOCK7				1.939902	2.249794
ARPC4	1.716356	2.463381			
EIF4A1	1.93007			2.230942	
TTLL12	2.391918			1.752711	
EXD2			1.955702	2.16254	
RPS11	2.593197			1.507423	
OSGEP		4.05778			
KCTD8	1.628728			2.411947	
NUP205			4.011751		
ACIN1				1.894929	2.097997
IQGAP3	2.151935			1.822634	
AP1G1				1.65093	2.316004
SEC62		3.962062			
NUP205		3.948217			
HK1				1.409166	2.506763
UTP14A	1.543426				2.36726
RPS27	1.88114			1.999324	
PPA1	1.871631			2.00716	
BANF1	1.706804				2.151884
KCTD5			1.956284	1.893866	
DNAJB6	1.701296			2.06817	
PLOD2	1.718174		2.000222		
UTP18				1.805531	1.869702
IDH3B					3.656461
BCL2L10	1.669657		1.974647		
NUP205	3.59716				
TUBAL3				1.486187	2.070702
UBXN1		3.527064			
SF3B2	1.581034			1.940455	
GEMIN7		3.502426			
MAGED1	1.546411		1.942262		
UBE2R2	1.658584			1.828429	
PYCR1		3.473145			

HSPA4L		3.456945			
SNX3				1.423574	2.022859
PTGES3L-AARSD1		3.436269			
NUP205				3.431362	
LAMC1			3.42987		
PPP2R2A	1.511792			1.916157	
PPP2R2C	1.511792			1.916157	
PDCD2L		3.42431			
PLS1		3.420443			
CHCHD2		3.419457			
CHCHD2P9		3.419457			
PSAT1	3.387672				
TCEB1			3.377493		
SEPT2					3.360739
CKM					3.357243
PDHB				1.606184	1.70882
FAIM		3.276535			
FAM162A		3.274371			
YARS		3.244497			
PSMD11		3.198749			
MRPS12		3.192674			
CSNK2A3		3.176461			
CSNK2A1		3.155441			
MDH1					3.123632
RANBP3					3.117864
CDK4		3.111454			
ZC3H14		3.100158			
ACAD9		3.09858			
ATP2B1		3.097474			
ATP2B2		3.097474			
ATP2B3		3.097474			
ATP2B4		3.097474			
CDV3		3.088501			
C19orf43				3.070379	
GALNT2		3.04039			
HMG20B				3.038872	
CHD1L				3.032643	
STK24		3.028067			
STK25		3.028067			
RIF1		3.016445			
ACAT1		3.012769			
TPM2		3.011784			
MT1A					3.005103
MT1H					3.005103
PARK7					2.96116

NARS				2.941801	
LMAN2	2.933306				
CAD			2.893146		
CCDC137				2.878167	
CKB	2.859572				
NAE1				2.826385	
MRPL12					2.728866
COPB1			2.664215		
ECM29				2.645276	
KIAA0368				2.645276	
POLR2H			2.606262		
GLS	2.584366				
DDX6			2.573355		
EIF1	2.526719				
EIF1B	2.526719				
PDCD6IP		2.511739			
DDX52					2.502621
PPARGC1A	2.486487				
MGA				2.473859	
MRPL53				2.473469	
ESD	2.470817				
RPL18A					2.453296
HEATR1				2.438567	
DOCK7	2.418229				
TUBA8	2.417177				
LIN7A			2.394971		
NDNL2	2.389726				
LSM14A	2.375981				
NUP54				2.332131	
LMNA					2.318342
ILVBL			2.309169		
PRDX1	2.303121				
PPP1R10				2.299803	
CKS2					2.286614
HN1	2.261527				
CIRH1A			2.256529		
TPT1	2.245111				
hCG_1789329	2.234425				
UBE2L3	2.234425				
MTX2	2.231884				
CALM2				2.228614	
FIP1L1	2.227989				
TOMM22	2.212318				
MAP4					2.212317
DNAJB2	2.206076				

LSS					2.191629
VAT1				2.183554	
NAP1L1	2.17548				
ATP5B	2.159474				
DDOST	2.159271				
TJP1				2.158858	
IDI1				2.156117	
TAGLN3	2.148493				
PFDN4				2.145523	
TRAX				2.136881	
PDE12	2.133018				
TMF1					2.132511
TUBB8				2.128161	
COIL					2.100155
DHX33	2.096235				
SDHB	2.094945				
PRMT8				2.088374	
MTHFD2				2.079437	
ANP32E	2.072497				
LDHB	2.071007				
MRPL46			2.069481		
TMEM9				2.054093	
PFDN5				2.052757	
UQCRC2	2.049927				
CMAS				2.044738	
DBI	2.038908				
TMED9			2.033713		
AGPS	2.032368				
CCT6B				2.028045	
NDUFB9	2.025071				
NCKAP1				2.022791	
AKAP1				2.011758	
FDX1					2.010488
SOD2				2.010397	
TUBA4B				2.008657	
PCK2				2.000648	
MKL1					2.000062
PRCC			1.997925		
PRPF38B					1.975254
NAA50					1.973319
ZNF598				1.962391	
DYNC1I2	1.957454				
DDX19A			1.952081		
HIBADH	1.951777				
PHF3					1.951484

NME1				1.945841	
ATG3			1.94484		
H2AFZ					1.94246
DNAJB1	1.938936				
UBAP2				1.938873	
POLDIP2	1.926464				
POLA2					1.925312
METAP2					1.923882
C9orf78					1.923778
APEH	1.921841				
CISD1	1.91902				
SEPT8			1.912634		
SEPT11			1.912634		
PABPN1	1.911763				
SMEK1				1.901475	
FBL					1.876947
UBE2D3				1.866316	
PFDN6				1.865474	
MARCKSL1	1.865082				
PITHD1				1.864952	
PPP1R12C	1.863474				
HIST1H2AC	1.861164				
SMNDC1				1.851577	
CUL1	1.828719				
IMPA1				1.825188	
TTC9C	1.821079				
DDT				1.814752	
DDTL				1.814752	
TBCD				1.81254	
DCTPP1	1.799434				
MAPKAPK2	1.798719				
MAPKAPK3	1.798719				
TROVE2	1.795647				
PDCD6	1.783259				
AMPD2	1.779243				
RAD50				1.776804	
SYNGR1	1.763476				
POLD1	1.763066				
PFN1	1.743875				
VPS36	1.743385				
C10orf71	1.731166				
NOB1	1.729767				
MRPL32	1.710582				
ANKHD1	1.710544				
CTPS1	1.704521				

NUCB1				1.690761	
APRT	1.686382				
SUZ12	1.685997				
ACOT7	1.685864				
NHP2	1.685038				
RBMXL3	1.676899				
POLR1E	1.666938				
FAM207A	1.652359				
UBA1	1.647073				
DIAPH1	1.633387				
RNH1	1.628907				
U2SURP	1.618614				
PHF23				1.614554	
HNRNPK	1.607343				
PRIM1	1.597759				
H2AFV	1.593994				
EIF3A				1.592466	
FERMT2	1.572889				
ITGB1	1.567502				
PM20D2	1.554792				
PPID	1.547211				
GINS1	1.540723				
PPME1	1.526376				
RRAS2	1.514699				
RFC2	1.506353				
C19orf47				1.505053	
SCFD1				1.472808	
WIBG				1.47246	
PTBP3				1.447429	
TSN				1.432603	
CPOX				1.400861	
RAP1A				-1.5022	
OSBPL11	-1.51357				
DNM3	-1.56321				
SMARCA4	-1.60746				
NOMO3	-1.6223				
TMED2	-1.62377				
RNF40	-1.63349				
MRPS25	-1.63374				
RBBP5	-1.63827				
RING1					-1.70282
RPL35A	-1.71821				
PPP2R1A	-1.72012				
HNRNPL	-1.72141				
DCXR	-1.7558				

PMPCB					-1.76768
CHRAC1	-1.83688				
MRPS31				-1.85732	
POM121				-1.85735	
POM121C				-1.85735	
TXNDC12	-1.85812				
CLPB	-1.88229				
ZNF638	-1.90258				
PAWR			-1.90414		
SYMPK					-1.90478
MED1			-1.9071		
NACC1			-1.90732		
NACC2			-1.90732		
PLD3					-1.90836
CHD3				-1.90974	
HUWE1			-1.9169		
WDR46				-1.91871	
RAB21			-1.9247		
LYPLA2				-1.92699	
CTTN	-1.93615				
RANBP9			-1.94245		
UFD1L					-1.94603
CBX2	-1.96467				
UBE2I			-1.96844		
GOLIM4	-1.98079				
CTNNA1			-1.98626		
PSMD9					-1.99158
CELF1				-1.99764	
KIF4B	-1.99927				
SHMT1				-2.00473	
RPL11				-2.00553	
GBA	-2.0069				
NOL7			-2.00735		
NDUFAB1			-2.01155		
EEFSEC				-2.0158	
TRUB1					-2.01907
PTGES2			-2.02908		
CLNS1A				-2.02988	
CUTC				-2.03018	
NEFM			-2.04794		
PPP2R1B				-2.05802	
KRBA2	-2.06098				
TXNL1					-2.06798
HIST1H2BA				-2.0687	
KMT2C				-2.07202	

ATP2C1				-2.07212	
RPL9	-2.07483				
EIF4E				-2.07665	
PSMG2	-2.07872				
MOV10				-2.07879	
LAP3	-2.08313				
RNF114			-2.08724		
RPL26L1	-2.09442				
GTF3C5	-2.09759				
HSPA8				-2.10432	
HIGD1A			-2.10586		
HIGD1C			-2.10586		
EMC10				-2.10896	
XRCC1	-2.11136				
PPAT				-2.1158	
DAD1	-2.12371				
DDRGK1				-2.12927	
CYB5R3				-2.13262	
LCLAT1			-2.13954		
DBR1			-2.1424		
KRT77	-2.14284				
CCDC58			-2.14546		
PELO					-2.14945
TRMT1					-2.14991
MOGS				-2.15906	
PAPSS1	-2.15919				
WDR82	-2.16014				
ALDH9A1				-2.16259	
KIF5B	-2.16446				
RBM12B				-2.16828	
SDE2	-2.17183				
CDCA7				-2.18226	
SHMT2	-2.19682				
MRPS18B	-2.20733				
SYAP1	-2.21275				
MAGOH				-2.21309	
GIPC1			-2.21413		
RPL36AL			-2.21485		
ARL1			-2.22219		
PGM1	-2.236				
SUCLG1			-2.2409		
DHRS7	-2.24285				
LRRC59				-2.24738	
TTBK1	-2.24854				
ATP2A1	-2.25179				

GPHN					-2.26148
IRAK1			-2.2662		
UROD				-2.27214	
VPS26A			-2.28432		
ACAA1	-2.29947				
PANK4			-2.30295		
MLEC				-2.31137	
BMS1	-2.3124				
DCBLD2			-2.33989		
TBL2				-2.34997	
RAB14	-2.35919				
GRN			-2.36509		
LAMTOR3				-2.37282	
MYL12B				-2.38689	
MRRF			-2.41219		
ABHD14B					-2.41239
PSMB5			-2.41301		
GNL1	-2.42219				
TM9SF3		-2.42335			
SBNO2			-2.42691		
MORF4L1	-2.4343				
CD276			-2.43508		
ATPIF1			-2.43791		
RBMXL2				-2.44121	
NEFH	-2.44445				
XPC		-2.44949			
SLC25A4				-2.4517	
HADH				-2.45223	
NPEPPS				-2.47122	
SCO2			-2.47276		
ETFB		-2.48834			
PGLS	-2.49142				
POLR2A			-2.49721		
NIPBL			-2.50935		
ABCC4				-2.51139	
EIF2A					-2.51465
DHCR24				-2.51668	
Nbla03646				-2.51668	
TMEM41B			-2.52061		
PC		-2.52993			
RBM23					-2.53505
UTP6				-2.53598	
PCBP3					-2.53672
RBX1			-2.54334		
DDB1				-2.54819	

DIDO1			-2.55182		
GK			-2.56566		
GK3P			-2.56566		
EIF3F			-2.57647		
SFN			-2.57736		
SLC7A1	-2.57785				
MCM7				-2.58545	
CPSF1	-2.59122				
CHAMP1					-2.59162
GDI1					-2.59405
HNRNPLL			-2.59659		
HNRPLL			-2.59659		
LRRFIP2			-2.59839		
PSMD10					-2.59897
CCT3	-2.61403				
CHMP2A					-2.61461
STX12			-2.64821		
CAPZA2	-2.67259				
EED		-2.68349			
THOC5	-2.69022				
PSAP	-2.69631				
C17orf85			-2.70602		
RPL5	-2.71899				
VTA1					-2.72539
USF1			-2.73449		
COG4					-2.74649
UTP20	-2.74654				
SACM1L				-2.75981	
ARMC6				-2.76764	
ACAA2			-2.79336		
MYH9				-2.79751	
CCT5				-2.79829	
PSMC6				-2.8007	
GRK6			-2.8225		
SUPT5H	-2.82668				
RBM3			-2.84403		
CLASP1			-2.85597		
BZW2				-2.8614	
HIST3H3				-2.88293	
UBR2				-2.88687	
MSH3		-2.89048			
RTN3	-2.89473				
GMFB				-2.90303	
SH3BGRL3			-2.91687		
CD47	-2.92683				

SERPINH1				-2.93421	
BAG2			-2.93854		
C8orf33	-2.9483				
UPF1	-2.95958				
CHMP1A				-2.97215	
PHIP				-2.97956	
NOP58	-3.00371				
KHDRBS2					-3.0101
MMAB			-3.01343		
TMSB10		-3.01967			
DLST		-3.02289			
CRYZ			-3.02327		
PNO1		-3.02556			
CAPN1		-3.02697			
SARNP				-3.03775	
C21orf59		-3.04659			
MAPK3		-3.04997			
ATIC		-3.05688			
ADNP					-3.06114
IKBKAP					-3.06241
NABP2		-3.064			
CROCC		-3.06424			
COX4I1		-3.06636			
GNL3		-3.06736			
PPAN-P2RY11					-3.0722
SNRPD2		-3.07296			
COPE				-3.07316	
RANGAP1		-3.07439			
ABCE1				-3.08331	
UPF3B					-3.09273
FARSA					-3.09371
SEC23IP	-3.10191				
SRSF2		-3.10301			
HSP90AA5P		-3.10333			
DHX15		-3.10422			
RPS28		-3.1099			
SIN3A		-3.11165			
MRPS30		-3.11307			
UFL1		-3.11457			
MAP2K4			-3.11497		
RARS			-3.11519		
PRKAR2A		-3.1157			
KRT5		-3.11605			
CLTB		-3.12088			
PRKAG1			-3.12308		

TCEA1		-3.12765			
SEC61B		-3.12897			
SRSF5		-3.13381			
EIF6	-3.14464				
RAP1GDS1		-3.14529			
YWHAG		-3.1595			
NUP188		-3.16376			
AATF					-3.1651
PREP		-3.17079			
HBA1		-3.17336			
DHRS7B				-3.18059	
PUM1		-3.18742			
PUM2		-3.18742			
GPD1L				-3.20154	
SYNGR2		-3.20197			
SREK1					-3.20439
ZC3HC1		-3.20576			
RRP7A		-3.20985			
RAB11A		-3.21053			
RAB11B		-3.21053			
CHAF1A		-3.21161			
TRIP13		-3.2142			
SMN1		-3.21942			
SMN2		-3.21942			
GNPDA1		-3.22023			
STRIP1		-3.24008			
VAMP2		-3.24358			
VAMP3		-3.24358			
NUP93		-3.24546			
SEC22B			-3.27461		
SUMF2		-3.27645			
RNF2			-3.28179		
U2AF1L4		-3.28476			
HSD17B10	-3.29486				
RAP1B		-3.29739			
KHDRBS3		-3.31358			
LSM7		-3.32069			
EIF4G1		-3.32379			
CSTF3		-3.32629			
ARMC6			-3.32637		
SLIRP				-3.32717	
RAD21					-3.33092
GOT1	-3.35792				
TOP1MT		-3.36225			
CHCHD1	-3.36453				

ATP6V1C1		-3.36457			
NOSIP		-3.3716			
AK2		-3.37229			
ASNSD1					-3.37668
UBE2S		-3.38723			
LOC102724023		-3.39518			
OXA1L		-3.42674			
NCOA5		-3.44026			
ARMC6	-3.44215				
RRP12		-3.44677			
AKR1E2		-3.44747			
TBCB		-3.45143			
LSM6		-3.45214			
AHSA1			-3.45441		
TIA1		-3.45806			
MYL6B		-3.46164			
AMOTL1		-3.46536			
HNRNPA3		-3.46885			
NDUFA7		-3.4703			
AKAP8		-3.48758			
GRPEL1				-3.49332	
OXCT1				-3.50082	
NPM3				-3.50628	
TCEB2		-3.51285			
GSPT1		-3.53017			
FKBP4		-3.53169			
C21orf33		-3.53852			
KRT17					-3.53999
SMARCD1	-1.61602		-1.92634		
YTHDC2				-3.54384	
MAP2K2		-3.54691			
ZBTB33					-3.54901
TRIM25		-3.55862			
SUMO4		-3.56917			
PSMB7		-3.57013			
PRPS1L1	-3.57737				
LIN7B		-3.58532			
NOL6		-3.58545			
WIZ		-3.58549			
EIF1AD		-3.58561			
TMEM14C		-3.58827			
YWHAQ		-3.60965			
PRMT3		-3.61031			
SRSF8		-3.61854			
SLC7A5	-1.65699		-1.96423		

SCAMP3			-3.62602		
ATAD1	-1.63517			-1.99321	
NDUFAF3				-1.86381	-1.78061
PRDX4				-3.64721	
ITPR3		-3.6474			
PPIH		-3.65588			
ABCB7		-3.67124			
KCTD15		-3.6785			
S100A11		-3.68005			
NRD1		-3.68804			
MRPL38		-3.69609			
ARCN1		-3.69972			
SLC4A1AP		-3.69995			
DKFZp313C1541			-3.70497		
RAB2B			-3.70497		
DNM2	-3.70972				
TBC1D15				-3.72648	
UBQLN1		-3.72751			
UBQLN4		-3.72751			
PSMD13				-3.72753	
AK6	-1.82588		-1.90312		
TAF9	-1.82588		-1.90312		
TAF9B	-1.82588		-1.90312		
PAK2			-3.75914		
SGTA			-3.75919		
PRKCSH		-3.76571			
EMC6		-3.76712			
TCEA2		-3.77435			
CSNK1E		-3.78414			
MAD2L1				-3.78417	
ATP5E		-3.79663			
ATP5EP2		-3.79663			
SMCHD1		-3.80584			
POLR3E	-1.74436		-2.06153		
PGAM2		-3.80782			
ASPSCR1				-3.80926	
SUGP2		-3.81071			
ZNF330		-3.81254			
SNRPB	-3.8186				
SNRPN	-3.8186				
INA	-1.65838			-2.16121	
TIAL1		-3.84441			
MTDH			-3.86663		
IKBIP	-3.87843				
WHSC1		-3.88839			

CSRP2			-3.88878		
FASN					-3.89296
SCPEP1		-3.89582			
KPNA5		-3.89825			
RANBP2		-3.90072			
FOXC2		-3.90175			
FOXG1		-3.90175			
FOXL1		-3.90175			
NEMF		-3.90486			
ELAVL2		-3.90543			
ELAVL4		-3.90543			
RPL34		-3.90934			
DACH2		-3.91386			
HELLS		-3.91615			
SUMO2		-3.91762			
CD2BP2		-3.92739			
LAMP1	-3.93566				
PPHLN1		-3.93576			
ADCK3					-3.94006
NDUFAF4		-3.9432			
CNDP2			-3.95345		
TMX1		-3.9727			
SLC3A2		-3.97392			
FDPS			-3.97661		
MARCKS		-3.99469			
NOP2		-3.99496			
GMPPB		-4.00627			
PCBP2		-4.01707			
DACH1		-4.01833			
RAB2A			-4.02944		
MAX		-4.03099			
NCAPG		-4.03302			
APOOL		-4.0333			
DKC1		-4.04402			
ATP5D		-4.05348			
CLUH				-4.05892	
TUFM		-4.06347			
TAF6			-2.12199	-1.94796	
MAGT1		-4.08106			
GINS2		-4.08285			
GNB1		-4.09386			
NCAPH2	-1.83997		-2.2595		
RSL1D1		-4.1026			
ARL8B		-4.10319			
NUMA1		-4.10964			

CAMK2D		-4.11135			
ST13		-4.1207			
BAG3		-4.13331			
CEP41			-2.31975	-1.81595	
STRBP		-4.138			
BABAM1				-2.07425	-2.06585
HMGA2		-4.14123			
DKFZp434B2017		-4.14403			
SAR1B		-4.14403			
DTYMK		-4.14683			
ATP6V1B1		-4.15742			
KRR1		-4.15838			
PSMB6		-4.16094			
NDUFC2		-4.16122			
SUMO3		-4.16718			
SUCLA2		-4.17274			
RHOA		-4.17416			
RHOC		-4.17416			
C7orf55-LUC7L2				-2.05823	-2.144
RIOK1		-4.20914			
RPS7		-4.20932			
RPS26		-4.21254			
RPS26P11		-4.21254			
STARD9		-4.21639			
TPT1P8	-1.59298		-2.63126		
CHMP4B		-4.23058			
RPF2		-4.23151			
SF3B1		-4.23631			
PAF1		-4.2384			
PPP5C	-2.33335		-1.90666		
PPP5D1	-2.33335		-1.90666		
GLTSCR2		-4.2403			
KRAS		-4.24032			
TARDBP		-4.24187			
PDCL				-4.2444	
RPL31		-4.24767			
FBXO22			-2.19836	-2.0615	
DUSP9	-4.26215				
DHCR7		-4.26841			
RABGGTB	-1.74383				-2.54144
RPS4X		-4.29997			
RPL13		-4.30034			
DYNC1H1			-2.68134	-1.62028	
MKLN1	-4.30843				
DDX39A		-4.30846			

HARS2		-4.30991			
MRPL1		-4.31086			
BLM		-4.32585			
RECQL		-4.32585			
KIAA1429		-4.32936			
RPL29		-4.33445			
PELP1		-4.33547			
PCYOX1	-1.9126			-2.42877	
SNRPF		-4.34219			
ARHGAP17				-4.34453	
HACD3			-2.22003	-2.12692	
RPL23		-4.35232			
VPS53			-4.35441		
PDIA4		-4.35543			
ALG5				-4.35789	
SMC1A		-4.35939			
UHRF2		-4.36837			
WNK1		-4.37825			
WDR89		-4.37898			
MECR		-4.38051			
MEN1		-4.38629			
UTP11L		-4.42287			
ECH1	-2.39458			-2.03307	
40787		-4.43311			
CMTR1	-1.70667		-2.72667		
C1orf131			-2.1067	-2.33745	
WDR18		-4.44914			
MCM2	-4.4692				
NDUFS6		-4.47477			
THOC3		-4.48021			
SPCS2		-4.48593			
STMN1	-1.9903		-2.4957		
COPB2			-1.98277	-2.50439	
BZW1			-2.42891		-2.05958
39692			-4.49969		
SMPD4		-4.50008			
C11orf98		-4.50023			
RBM39		-4.50104			
MRPL34		-4.50409			
HM13		-4.50811			
HP1BP3		-4.50836			
HDLBP			-4.52934		
HECTD1	-4.53146				
ETFA		-4.53205			
HOXA5		-4.53348			

AAMP				-1.84889	-2.69083
ANKLE2		-4.54385			
CTPS2		-4.54411			
AMOTL2		-4.54886			
IMPDH1		-4.54991			
RAP2A		-4.55093			
RAP2B		-4.55093			
RAP2C		-4.55093			
DVL2	-2.15304		-2.4129		
RFC4		-4.56736			
AIMP1	-2.25539		-2.31634		
PPT1	-2.07039				-2.5019
KPNB1		-4.57287			
CUL3		-4.57677			
RAB25		-4.57789			
ANAPC5		-4.5823			
DMAP1		-4.58421			
GCN1L1	-2.33777		-2.24706		
GAPDH			-2.02558	-2.56521	
POLR1B		-4.594			
TSFM			-4.59536		
DPYSL5		-4.59576			
THUMPD3		-4.59677			
PNPT1		-4.59733			
DEGS1					-4.61022
CLGN			-4.61398		
EIF4G3			-4.61573		
SF3A2		-4.62194			
CTBP2		-4.62534			
SRPK3		-4.63375			
SNRPA		-4.64338			
ARPC3		-4.65029			
CHD1		-4.65099			
GHITM				-2.38453	-2.2668
HSP90B2P		-4.66243			
POLR2G				-2.07974	-2.5859
ACTR3B		-4.67257			
AKR1B15		-4.68028			
KPNA3		-4.6915			
KPNA4		-4.6915			
NDUFB11		-4.69716			
KIF5A		-4.6985			
KIF5C		-4.6985			
PDS5A		-4.70084			
GNB3		-4.70233			

GNB4		-4.70233			
TUBB4B			-2.56464	-2.13838	
RPA2		-4.70762			
CTH		-4.71204			
MATR3		-4.71838			
DDX3Y		-4.73052			
CNOT1			-4.7443		
PES1		-4.74698			
NIN		-4.75102			
CALCOCO2			-4.75649		
POR		-4.75861			
MYO1D		-4.7727			
PICALM		-4.77503			
LSM8		-4.77841			
PNN		-4.78213			
USP39		-4.78456			
TEX10		-4.7863			
ALB	-2.73003		-2.05899		
UFM1		-4.78912			
RABIF				-2.46588	-2.33451
TARS2	-2.33506		-2.47315		
RPL8		-4.80848			
YBX3	-2.74366			-2.06569	
REXO2		-4.81755			
CYB5B	-2.81992		-1.99823		
NCAPD2		-4.82017			
LEPRE1		-4.82532			
NOMO2	-1.6223			-3.21712	
ERH		-4.84884			
ALDH7A1	-2.66329			-2.19558	
FAHD2A	-2.2669			-2.59539	
RBX1		-4.86993			
EXOSC4		-4.87591			
KAT7			-2.65347	-2.23367	
DIEXF		-4.88866			
POLR2E				-2.67606	-2.21306
PTGES3		-4.88916			
SCP2			-2.49781	-2.39415	
SNW1		-4.89732			
SP100		-4.89926			
HCFC2		-4.90101			
RAD23A		-4.90182			
PHF5A					-4.90469
ARMC6					-4.91041
UBL5		-4.91138			

CD59		-4.91396			
THOC1	-2.11149	-2.8038			
FAM3C				-2.18272	-2.73686
SUMO1				-2.3563	-2.57072
CLPTM1		-2.50296			-2.4306
KLC1		-4.93843			
DUS1L			-4.94347		
WBP11				-2.37605	-2.56994
ATXN2L	-2.08421				-2.86772
YY1		-4.9533			
SNRPC		-4.97465			
HEATR6			-2.91466		-2.0729
DKFZp686E0752	-2.86777			-2.12242	
RSF1				-3.04494	-1.94852
PPIL1				-2.60748	-2.4619
MAPK8IP3			-5.07467		
RPS23			-3.05825	-2.07181	
HIST2H2AA3		-5.15158			
HIST2H2AC		-5.15158			
PSMA6			-2.72663	-2.43483	
AP2M1	-3.19158		-2.01803		
CDSN			-5.28789		
SLC1A5	-1.64313		-3.6626		
RPL19			-2.17058	-3.13779	
RRS1			-2.87846	-2.44128	
CADM1	-1.7068		-3.61404		
DBT	-2.00068	-3.32128			
BMS1	-2.85731		-2.47589		
MCM4				-2.07439	-3.27649
XPO1		-3.18305			-2.21806
IARS		-3.41016		-2.02828	
PYCR2				-2.4777	-3.00706
PRPF31	-2.02676	-3.48579			
KIAA0101			-5.53152		
UBE3A				-5.56192	
USP14					-5.56857
ACLY	-2.77196		-2.80471		
STAU2		-2.49433	-3.13367		
FKBP1A	-3.57973			-2.06275	
H3F3C		-3.5235		-2.12512	
APIP	-5.66092				
SLC39A7		-2.48208	-3.18378		
TNKS1BP1	-5.6726				
DUSP3	-2.64822				-3.02996
EFTUD2	-1.91489			-1.63057	-2.1464

HIST2H3PS2		-3.56135		-2.16159	
ATXN10	-1.78596		-1.99029		-1.9654
ATF1		-2.54284	-3.19949		
CREB1		-2.54284	-3.19949		
CREM		-2.54284	-3.19949		
TUBA1C		-3.08797		-2.68811	
POLR2F				-2.9935	-2.78556
EIF3D			-2.89	-2.89695	
MMTAG2	-1.68289	-4.12731			
COMMD6			-5.81204		
SLC19A1		-2.69573	-3.12034		
CASC5	-5.8261				
ARF1			-2.69992	-3.14567	
EPHA2			-2.75417	-3.11998	
RPL26	-2.68106		-3.22418		
SPCS1					-5.90688
BRD8		-2.63529	-3.27649		
NUP62				-2.31662	-3.60256
ARG1			-3.21918	-2.7248	
TP53BP1					-5.94788
LARS	-2.4397		-3.51071		
RUVBL2	-1.96323				-3.99436
TP53		-3.89935		-2.07043	
BCLAF1		-3.20723	-2.7887		
ARF6			-2.56111	-3.45671	
HMGN3				-2.61699	-3.42233
IGF2R		-3.87918		-2.18229	
DNTTIP2		-2.92574	-3.17887		
MAP1B		-4.37975		-1.73794	
PGM2			-3.7279		-2.39205
NCAPH					-6.13811
UBXN4		-2.81945	-3.33139		
YBX1				-4.12224	-2.03098
SSU72		-2.75194	-3.4141		
SURF4		-3.35777		-2.81766	
IGF2BP3	-1.62726	-4.55308			
HAX1		-4.04581		-2.13581	
KIAA0415		-6.19186			
TBRG4		-6.20699			
DDX46		-3.39499	-2.82115		
GRB2		-4.11996			-2.09876
TALDO1			-4.04156	-2.19023	
HSP90B1		-4.18381		-2.06155	
WBP2			-1.90798	-2.27402	-2.07031
GBF1			-6.2616		

LLGL1			-6.26829		
FOXK1		-6.27134			
PRPH	-2.25491		-2.13499	-1.88298	
HNRNPD		-3.11219			-3.17615
TPM3		-6.2907			
IMPDH2			-3.99412	-2.30186	
LBR		-4.25659		-2.06485	
DKFZp586I031	-1.73296	-4.60639			
PNP			-2.10191	-2.05808	-2.19873
C12orf43		-3.5816		-2.78515	
STMN3			-6.38517		
VIM	-1.98481		-2.30395	-2.10661	
CTDSPL2	-1.75362		-4.64741		
UBR7		-4.24704			-2.16714
PRRC2A		-2.85792	-3.5603		
SMC6		-2.81204	-3.61219		
RPS2			-1.90566	-2.55842	-1.96352
PSMC5	-2.45609	-3.97963			
WFS1		-4.48343	-1.96365		
CTR9		-2.8666	-3.58104		
HCCS	-4.24395		-2.21337		
RPS15		-4.31127			-2.14704
STIM2	-1.77339	-4.69229			
MYL6		-4.42517	-2.05638		
PTRH2			-3.38068		-3.10879
PSMA2			-2.82485	-3.68476	
SEC23A		-2.89402	-3.63712		
VPS25	-1.66645		-2.10018		-2.76463
RPS27L		-4.68014		-1.85918	
CD99	-2.20646	-4.33527			
TUBB2B				-2.53882	-4.01458
STAU1		-3.48621	-3.13367		
TRAP1			-6.62576		
MTA3		-4.47199		-2.17643	
DES	-1.61197		-2.66424	-2.38222	
PSIP1		-3.50772			-3.15175
MRPL22		-3.29908			-3.36935
PRMT5	-3.6271	-3.087			
AP1M2			-6.72004		
RPL36		-3.59391			-3.14444
CSNK2A2		-3.70084			-3.04023
LTF			-3.71124		-3.06105
DBN1		-4.96934		-1.80413	
FKBP5	-3.09271		-3.69492		
PRPF40A		-4.83092	-1.96283		

PSMA4		-3.16962		-3.62694	
AGPAT9				-2.41722	-4.38844
NAA40		-3.06197	-3.75137		
HMGCS1		-3.3449			-3.47501
PSMA1		-3.17344	-3.65412		
GNG12		-3.39531	-3.44012		
MLLT4	-2.15711	-4.68545			
GEMIN4		-6.8668			
PEPD		-3.16862	-3.7052		
SNX17			-3.13903	-3.76	
WDR43		-3.42906			-3.47083
KRT16		-6.92303			
EIF4B		-4.27218			-2.65227
IMMT		-4.91601	-2.01205		
BCAP31	-2.26236		-2.98617		-1.7062
CLTA		-4.49596	-2.45955		
DDX4		-4.91032		-2.10313	
ACTBL2				-3.57095	-3.44733
RPL37A		-5.01253			-2.03255
TTC19			-2.49252	-2.59786	-1.96251
MAZ	-2.14309			-3.19739	-1.71643
RPS24	-2.2408			-2.70242	-2.11956
ABCF1	-2.11633			-2.50547	-2.5132
HMGN4		-3.94744			-3.25572
CPNE1		-3.72216	-3.48592		
IST1		-3.30526	-3.92534		
STUB1	-4.15864	-3.07293			
LIG1	-2.59695		-2.63314		-2.05609
PRPSAP2			-3.49004	-1.97091	-1.82715
AGAP3		-7.30807			
RCN2		-3.719	-3.63816		
LCP1	-4.06945	-3.29955			
RPL37AP8	-2.38996	-4.98825			
NUDT19			-3.96908		-3.4135
DRG1				-5.52718	-1.88224
IDH1	-3.3467		-4.07365		
LMAN1			-7.43399		
IDH3A		-4.44182	-3.01348		
GLRX5	-2.24875		-3.2112		-1.9961
OGFR	-3.77385			-3.68489	
EZH2	-2.5748	-4.89205			
CTCFL		-4.68022	-2.79005		
NDUFA2			-4.923		-2.54815
SRRM2		-3.3061	-2.31777	-1.85505	
CKAP2		-4.47804	-3.02743		

PHPT1			-3.66874	-3.84566	
YY2		-4.94744	-2.57507		
WARS		-4.89843	-2.63118		
EIF4G1	-3.15117		-2.21606	-2.20032	
SNX5	-2.37434		-2.64313	-2.55127	
C4orf27			-4.1438		-3.43672
SRP68	-3.78651	-3.79622			
MRPS28			-2.18607	-2.68523	-2.75681
UBR4	-3.13122		-2.48306	-2.02164	
RPL30		-4.86955	-2.76793		
PRPF19		-3.04747	-2.13839	-2.46801	
WDR33		-4.14479	-3.50948		
SRPRB		-4.15589			-3.50606
EIF3G		-3.36551	-4.30225		
RPL10		-3.47466	-4.22545		
METAP1		-4.45492		-3.25451	
CNBP		-3.15136		-1.97395	-2.58908
NOLC1		-2.60632	-3.15028	-1.96363	
RAVER1		-2.94495	-4.79086		
EIF4G2		-3.51698		-4.25063	
NASP		-3.1187	-2.55667	-2.11977	
SRP54	-2.7101	-2.43348	-2.65692		
NCL		-4.88912	-2.94944		
SRP19	-2.17421	-5.68217			
USP5	-4.23363	-3.63461			
EIF4A3		-4.88278	-2.98695		
HOXB9		-3.35205	-4.52173		
RBMX		-4.57954	-3.37472		
PTRHD1	-2.33547		-1.9096	-1.83947	-1.90301
CBX1		-5.21183			-2.7781
PLRG1		-4.83618			-3.20582
NOMO1	-2.18268	-2.67801	-3.19347		
CTAGE15				-3.58782	-4.47776
CTAGE5				-3.58782	-4.47776
CTAGE6				-3.58782	-4.47776
TAF15		-4.19254			-3.99674
APMAP	-2.33044		-3.81224	-2.10491	
CPT1A	-2.18924		-6.06365		
PBK	-2.44687			-2.77982	-3.05143
MCCC2	-3.11452		-3.21069	-1.96365	
MCM5			-2.34014	-6.00743	
MPHOSPH10		-8.36237			
CDC42		-3.45373	-1.93861	-3.0633	
HNRNPM		-3.69601		-2.69068	-2.1229
RQCD1	-2.60985		-2.47862	-3.42139	

IDH2		-4.70592	-3.82614		
CTU1			-4.31381	-4.26613	
TRA2B		-4.77632	-3.81167		
CNN3		-3.57681		-2.25604	-2.83736
RUFY2	-2.71467		-4.30194	-1.72434	
TSSC1				-4.17522	-4.60569
NOL11		-3.26638		-2.05178	-3.49127
PSMD12			-2.64917	-6.20851	
PPP6R3	-4.66827		-4.19324		
ALDH2	-2.33637	-4.41162		-2.14713	
RAB1B		-4.65953	-2.18092		-2.09605
MTCH1			-2.18375	-3.48679	-3.27149
POR	-1.81961	-3.25148	-3.92775		
LONP1	-3.83915			-2.54906	-2.61854
MIA3	-3.65132				-5.36818
MDN1	-2.56547		-3.06559	-3.39707	
SF1	-2.05474	-4.69558	-2.28328		
ECI2				-5.24312	-3.87416
GOLGA2		-3.60266	-5.5469		
TES				-3.89731	-5.2811
BLVRB	-2.98371		-2.34786	-3.90642	
SEC31A		-3.27515		-5.99776	
LSM4		-4.12838		-2.45591	-2.69135
ZC3H7A		-4.89656	-4.49299		
ZC3H7B		-4.89656	-4.49299		
GNG5	-1.6705	-3.51748	-4.27021		
KLF12		-4.98045			-4.49282
KLF3		-4.98045			-4.49282
KLF5		-4.98045			-4.49282
KLF7		-4.98045			-4.49282
MYNN		-4.98045			-4.49282
ZSCAN16		-4.98045			-4.49282
ZSCAN2		-4.98045			-4.49282
ATP1A1		-3.84389		-2.45889	-3.18599
VEZF1		-5.17099			-4.41785
FANCI		-3.39482	-2.12464	-1.93601	-2.18894
UBA52		-4.53541		-5.14893	
RPS3	-2.01822	-2.55162	-3.55774	-1.56856	
GLG1	-1.89069	-3.92503	-3.916		
HYPK			-2.36668	-3.75753	-3.61602
EPHA1		-5.85843			-3.92295
HIST1H1C		-4.9117			-4.89486
STT3B		-3.58404		-2.84638	-3.39842
MRPS2		-3.96438	-2.35695		-3.52165
HINT2	-1.64731	-3.33558	-4.86458		

RAE1	-2.294		-4.54597	-3.01205	
TBCA		-4.52328		-2.49885	-2.86716
MAGOHB			-5.15879	-4.81772	
JAGN1	-1.76587	-3.2846	-5.01074		
HAUS5			-5.00363	-5.11961	
HRNR	-1.71872	-4.44977	-3.99967		
HDAC3		-4.78826		-2.79245	-2.63155
OPA1	-1.95731	-2.98096	-3.84387	-1.4548	
EIF3L		-3.27529	-2.32363	-4.66606	
TUBA1B		-3.84488		-3.32749	-3.12603
NFKB1	-2.38713	-4.68233	-3.24169		
NUP214	-1.68229	-4.20825			-4.42111
AHI1	-4.09986		-2.72808	-3.48705	
CTCF		-4.8681	-3.24057	-2.21565	
UQCRHL		-4.96787	-5.36534		
MSH6	-3.1869		-3.83339		-3.34302
TXNRD2	-2.67099		-2.82549	-2.3184	-2.57116
PPP2CB	-3.02403		-2.20384	-2.32244	-2.86148
TKT		-4.78226	-3.45294	-2.20249	
CKMT2	-7.31166	-3.13142			
BTF3		-4.52325	-2.23324	-3.69326	
CCDC88A	-1.92896	-3.70426	-4.82441		
DCTN1	-3.68118		-2.08564	-2.54467	-2.16343
NUDT4	-3.35216		-3.71012	-3.47625	
MRPL45		-4.72601		-3.02012	-2.8789
PTPN1	-2.41264		-6.11431	-2.10032	
PRPF3	-1.64692	-3.75091	-5.24415		
RRP1	-1.81888	-4.05443	-4.83157		
SPC24	-3.18572		-2.63221	-2.65616	-2.26324
PRRC2B	-5.33384		-5.41079		
TRAFD1	-4.65776		-6.09701		
ARF4		-3.89356	-3.36684		-3.50419
PQBP1		-3.5691	-3.19545	-4.01847	
QRSL1		-4.83718		-3.0823	-2.87874
MECP2	-1.65892	-2.9638	-3.79068		-2.40192
PPAN	-2.24208	-4.23519			-4.33856
PRPF8		-4.69843		-3.06528	-3.05385
GIGYF2	-5.23002		-5.59673		
CLIP1	-5.52272		-5.39031		
H2AFY2		-3.33639		-2.34605	-5.30917
MORF4L2	-1.70126	-3.85077	-5.46515		
EEF1D	-3.18076	-4.22798	-3.63028		
BRD2		-4.92208	-1.99984	-2.07824	-2.07841
RBBP7	-1.96283	-4.50157	-4.67233		
LIN28B	-1.99355	-4.2553	-4.92341		

EIF3K		-4.64853	-4.06543		-2.48863
NPLOC4		-4.82177		-6.45841	
FSCN1	-1.74446	-2.76885	-1.97186	-2.95836	-1.85797
AK4	-2.97859		-4.88446	-3.4413	
MAEA	-6.3216		-5.08716		
H1F0	-2.29637	-4.06689	-5.08023		
DNAJA2	-2.16084	-2.8405	-3.11171	-3.41109	
CCDC40	-2.79399		-4.36784	-4.37256	
ME3	-2.76386		-3.08055	-2.54698	-3.14328
ZFP91		-4.0916	-3.78816	-3.68301	
PRPS2	-4.44689		-3.87921	-3.26656	
CTNNA2	-6.41266		-2.42278	-2.80351	
SAP18	-1.76106	-3.69719	-4.28512	-1.93191	
TCOF1	-2.52958	-4.72993	-4.43958		
GPI	-4.13662	-3.30016	-4.39904		
ARFIP2	-1.52336	-4.24947	-2.01632	-1.80654	-2.31031
CEBPZ	-3.38903	-4.01362	-4.52096		
GFAP	-2.56903	-4.53128			-4.93394
PRPS1	-4.5759	-4.57822			-2.88845
SRA1	-4.82263		-7.28302		
FMR1		-4.95257		-2.53209	-4.71424
PPP1CB	-3.42358	-4.65436	-4.42937		
SRFBP1	-1.98805	-3.34978	-7.29198		
HSPB1			-4.73576	-4.36162	-3.8192
EEF1E1		-6.86054			-6.06483
H3F3B		-6.76445		-4.24893	-1.92272
SEC23B	-4.18584		-2.82511	-5.95474	
SND1	-2.71156	-4.81229	-5.49595		
COASY			-4.54123	-4.47389	-4.10802
CNNM3	-4.16759		-4.94109		-4.09574
HIST2H3A		-6.64183	-2.20666	-4.39613	
COX6B1	-2.10898	-4.96415	-4.38356	-1.8648	
COPS7B	-2.27656	-3.36173	-2.5223	-2.48199	-2.72831
PGM3	-2.49373	-4.7844	-3.32577	-2.80093	
CANX	-3.01042	-4.67975	-5.74233		
GLOD4	-4.39466		-4.95115		-4.09903
MMS19	-5.22148		-5.76786		-2.45932
RABGGTA	-4.68242		-4.35548	-4.43311	
DDA1		-3.66045	-4.0701	-3.04059	-2.7321
ACADM			-4.351	-4.66082	-4.52815
ACSS1		-5.01948	-3.97647		-4.68342
EEF1B2	-4.87672			-3.02157	-5.90591
MPI	-5.76336		-5.21043	-2.96149	
ASF1B	-3.65531	-3.21335	-3.26664	-3.81284	
RDX		-4.48743	-3.93859	-5.56478	

SAE1	-2.19875	-4.24549	-4.93982	-2.64291	
TUBB3		-7.05714	-7.00555		
TLN1	-3.63884	-3.06067	-3.12949	-4.33643	
PITRM1		-3.53572	-5.89462	-4.7649	
ANKFY1	-4.80386		-4.51406	-4.91232	
NIFK	-4.20641	-2.60795	-3.10476		-4.37299
WDR26	-3.12993		-3.98745	-3.7618	-3.57455
MGEA5	-2.84603	-3.48507	-2.14835	-2.38809	-3.65902
MRPS11	-2.77407		-2.97123	-5.17664	-3.66401
M6PR	-3.56131		-3.51921	-3.39562	-4.16329
H2AFX		-6.62986			-8.08532
DCAF16	-5.52875		-4.54073	-4.65581	
MRPS7		-3.14333	-3.63385	-3.63196	-4.34917
ASNS	-3.26016	-3.08155	-3.37231	-2.27543	-2.8064
PUS7	-5.16088		-3.97028	-5.66554	
GABPB1	-3.47805	-3.35808	-2.18386	-2.48375	-3.35332
BRAT1	-5.56477		-4.48034	-4.81625	
INIP	-4.27096	-4.9338	-5.69663		
SUCLG2	-3.55896	-3.9432	-5.67396		-1.7967
LIMD1	-4.68498		-5.08561	-5.2122	
VARS	-5.93881	-4.2199	-4.83735		
SRM		-5.48838	-2.14884	-1.92881	-5.55824
BICD2	-4.67942		-5.56768	-4.88815	
GLMN	-1.81899	-4.22006	-2.30759	-4.64907	-2.24806
H3F3A		-6.76445	-2.71014	-4.24893	-1.92272
TIMM8B	-3.98872	-5.30694	-6.35228		
ARHGEF2	-4.53546		-5.31077	-5.82438	
RBM22	-2.14895	-5.78662		-2.1559	-5.59411
TBC1D10A	-5.22328		-5.20582	-5.58217	
ERP44	-2.89223	-3.31769	-4.16772	-5.64274	
ARF3		-4.22191	-3.54668	-4.73116	-3.53436
EPPK1	-5.4896	-3.04698	-4.88533	-2.70167	
LCMT1	-5.21382		-5.56951	-5.42072	
CS	-3.25118	-3.54226	-6.87837	-2.5323	
KPNB1		-8.46766			-7.89599
ARKL1	-4.97984		-5.85681	-5.53419	
C18orf25	-4.97984		-5.85681	-5.53419	
TMEM43		-5.33729	-4.5333	-2.50588	-4.02813
SCRN1	-4.59368	-4.2047	-3.76325	-3.87301	
ARPP19	-5.81093		-6.06673		-4.74266
KARS	-6.11055	-4.55625	-6.04847		
RDH13	-3.98578		-4.49534	-4.0922	-4.31779
EBP	-5.832	-4.91765	-6.21543		
RCCD1	-5.78196		-5.71506	-5.51246	
GFPT2	-3.50895	-4.38267	-2.97668	-2.79141	-3.40927

PRPSAP1	-5.64579	-3.71109	-3.31804	-4.55715	
HNRNPH1		-8.77373			-8.47423
RPS25		-9.25642			-8.20431
BAX	-3.32021	-3.99552	-2.55488	-3.41644	-4.22591
ASMTL	-5.75089		-6.92835		-5.04693
UBE4A	-6.07745		-6.01091	-5.68513	
ZMYM3	-2.01258	-7.44503	-3.11302	-2.37103	-2.87086
UBE2D2	-4.78417	-7.63231	-5.6772		
LPCAT1	-4.15719		-4.33641	-4.55881	-5.23647
TPP2		-4.98151	-4.10636	-4.91478	-4.48483
SETBP1	-4.96562	-3.54104	-3.67386	-2.9208	-3.42441
LRBA	-5.89315		-6.6587	-6.02414	
MARS	-5.27435	-6.04085	-4.33166	-3.00277	
MDC1	-1.99005	-4.15421	-4.53817	-3.85958	-4.46956
HMGN2	-2.09869	-7.09145	-5.01563		-5.13983
FOXK2	-3.71763	-7.63066	-4.11425	-3.92256	
HLA-B	-3.64988	-5.34063	-2.8023	-3.83091	-3.83927
HLA-C	-3.64988	-5.34063	-2.8023	-3.83091	-3.83927
PDCD4	-4.523		-5.51754	-5.28284	-4.8968
RNMTL1	-6.71739	-6.38833	-7.51742		
RPL27A		-10.0358		-2.10736	-8.54864
SMG9	-4.10003	-5.55353	-3.34812	-3.84959	-3.92629
FAF1	-2.89307	-6.4032	-3.65202	-3.2299	-5.08432
CASP3	-3.6092	-6.17586	-3.73259	-3.3312	-4.87781
RARA	-3.94285	-5.86028	-3.90118	-4.41347	-3.91848
GPKOW	-4.74284	-5.34473	-5.16134	-4.29502	-4.04348
TMEM180		-7.20109	-5.82679	-6.75557	-6.37137
