# Supplementary material for: GPR19 Coordinates Multiple Molecular Aspects of Stress Responses Associated with the Aging Process
Source: Int J Mol Sci. 2023 May 9;24(10):8499. doi: 10.3390/ijms24108499 (PMC10218176; doi:10.3390/ijms24108499)
Supplement: Supplementary file 1 [file ijms-24-08499-s001.zip › Data-Supplement-Maudsley-et-al-2023-Table-S4.pdf]

Data Supplement

GPR19 coordinates multiple molecular aspects of stress responses associated with the aging process

Stuart Maudsley [1]*, Claudia Schrauwen[1], İrem Harputluoğlu[1], Deborah Walter[1], Hanne Leysen[1], Patricia McDonald[2]

1. Receptor Biology Lab, Department of Biomedical Science, University of Antwerp.
2. H. Lee Moffitt Cancer Center, Tampa, FL, USA

* Corresponding Author: Prof. Stuart Maudsley – stuartmaudsley@uantwerpen.be: receptorbiologylab@gmail.com

Table S4. Mean GPR19 induced protein expression alterations all compartment extracts. Significantly-regulated proteins (identified by official Gene Symbols) found in cells, differential between mock-transfection and the specific GPR19 expression level (0.5, 1, 2, 5, 10µg), are represented as a log2-transformed and normalized expression ratio between the specific GPR19 expression level and the expression level found in cells receiving empty vector transfection.

Gene Symbol	0.5	1	2	5	10
FBXO41	10.69676	9.083785	10.7104	9.093955	9.55885
PABPC4	7.79277	7.089818	7.918565	6.834981	7.504267
RAD50	8.396166	7.69871	7.083238	6.343929	7.324503
TUBB4A	8.61452	3.305781	7.270007	3.921923	9.986918
PDHA1	6.800228	6.745516	7.592351		7.789457
HDAC2	6.980002	4.572511	7.062519	4.546455	5.417666
DIABLO	5.956536	6.117558	6.896559	6.473151	2.576088
GNL3	6.41779	6.547671	5.554245	7.044593	2.428604
TACC3	5.255857	5.646168	5.230488	6.347601	4.998936
PGM3	5.856697	5.963834	5.085445	5.101395	5.409277
GATC	4.763431	5.843191	5.230535	5.95175	5.139965
HMGA2	6.025469	6.291443	3.564368	4.1637	6.806156
MTHFD1L	5.03443	5.13742	5.644408	5.359593	5.318951
PDAP1	9.222202	8.955604		3.87075	4.442603
G3BP2	6.444117	2.507962	6.417986	5.351643	5.620753
CKS1B	5.350146	4.71393	5.694834	4.757231	5.632189
LYZ	4.050207	7.279965	2.903863	8.270085	3.640887
HIST1H3A	6.705344	5.554681	5.815956	2.535865	5.469338
PCK2	6.563432	4.35029	4.916279	3.959524	5.521555
PRKACA	6.351085	2.344109	5.787345	5.140983	5.545881
ALDH7A1	5.939367	4.592883	5.574678	3.816389	5.122418
MDC1	5.577719	5.234245	4.315547	4.739501	4.836119
MAT2B	4.3955	5.011012	5.450945	4.575029	5.189891
ATP2A3		6.154594	6.655536	5.347379	6.317634
CCDC47	6.64904	5.46689	2.513803	3.958372	4.654893
TRAP1	9.638381		9.579779	3.96138	
PLS1	5.729315	3.552736	5.694644	3.81653	4.259494
DNAJB1	4.243082	4.455654	4.976607	5.043156	4.251115
TMCO1	3.686299	3.68318	3.757187	5.152776	6.363362
LIMA1	3.630153	5.926437	4.433989	4.187908	4.322675
MCM3	5.15333	5.730416	4.740339	4.30103	2.251686
EIF5	4.252595	4.66208	4.334015	4.067817	4.837141
CHCHD2	6.249968	3.22541	5.912626	2.499023	4.102614
CHCHD2P9	6.249968	3.22541	5.912626	2.499023	4.102614
NUP210	5.34126	4.045645	5.126187	2.866908	4.500165
ETFA	4.40973	5.670759	3.73405	4.027265	4.028338
TUBA1A	4.663854	5.565738	4.189228	3.59049	3.775831
COLGALT1	4.255547	4.442226	4.261642	4.248381	4.5388

UBA2	7.754652		6.813613	7.129645	
FH	5.41596	3.047286	5.364222	3.710613	4.096651
RALA	2.673472	5.385117	3.319995	4.480454	5.551128
RPS24	5.218748	5.433923		5.209906	5.444566
FARSB	4.224168	4.234982	4.570312	3.002996	4.990068
M6PR	4.640428	3.09044	4.399486	4.200679	4.571461
CLPB	4.117615	4.983297	3.966944	3.667042	4.131427
THOC3	4.848999	3.51066	4.882007	3.359276	4.258321
CBR1	4.910196	4.824799	3.654328	3.62262	3.660259
GDI2	4.133909	5.174894	3.27964	2.9253	5.060906
SRPK1	3.509229	4.74124	3.938798	4.00468	4.173394
DNMT1	1.964771	5.353118	2.593665	5.631031	4.80777
PPAN	4.893352	3.032209	5.148684	4.845688	2.406189
ERLIN1	4.741597	3.575653	4.040991	4.015641	3.899485
DDX19A	5.441245	6.157138	1.952081		6.622569
HIST1H1B	5.153372		4.245155	6.479236	4.268037
RTN3	2.830226	4.197738	3.638109	4.835835	4.50803
SSBP1	4.081832	4.549111	4.397995	4.679666	2.25948
CBR3	4.652119	4.7428	3.038877	3.437425	3.440067
CHD5	3.454825	2.599169	3.845628	4.005116	5.226618
TALDO1	3.693871	3.549166	4.304534	2.850333	4.534236
ETF1	4.890324	4.824556	5.130607	4.003568	
RIF1	8.395669	3.016445		7.335939	
ANXA6	3.739491	4.35529	3.968379	3.240796	3.430652
DTYMK		4.721363	4.302695	4.702006	4.868732
CCDC6	4.36654	2.398018		3.744082	8.004424
COPS4	3.806237	4.614148	2.396573	2.947247	4.438288
FHOD1	3.254352	3.08163	3.575376	3.525703	4.761413
RBM12	3.282013	3.917967	3.733188	3.135187	4.114262
NAA10	4.573187	5.639501		3.14981	4.746903
GRB2	4.527323	4.485842	4.436292		4.659344
RPN2	3.522472		4.303988	6.208293	3.972253
PPM1G	3.614445	3.266735	2.987266	3.633269	4.47674
KIAA1524	2.59011	4.156684	3.005384	4.310237	3.836749
GRWD1	3.411892	3.875944	3.603543	3.388359	3.596786
HNRNPUL1	3.309534	3.296412	4.089483	3.557651	3.60201
TMED10		5.20399	5.339811	2.053773	5.199549
CDKN2B	2.039445	3.393592	5.046009	3.520885	3.688438
BASP1	3.070783	4.488964	2.960233	2.693557	4.387876
RPLP2	4.250819	3.084179	3.485987	3.446997	3.241341
CIAPIN1	4.272707	3.534425	2.490515	3.02188	4.186033
DCTN1	8.396502		2.100582	3.739887	3.223941
TPI1	4.536716	3.228897	3.653853	2.26586	3.692306
APEX1	2.423478	3.811718	2.763255	3.836632	4.454563
SRI	3.159661	3.461419	3.887171	3.301778	3.45776
HMGB1P1	2.663372	4.085604	2.788801	3.244377	4.444233

COX7C	4.305266	3.275132	3.706722	2.56665	3.152461
NANS	3.985236	4.016078	3.777602	1.69122	3.484526
PIN4	3.67267	3.855606	2.676915	2.657228	4.092039
CLGN		7.256758	-4.61398	6.912912	7.304615
MT-CO2	5.108253	-2.77347	5.634829	3.571318	5.229374
EPB41L2	1.943074	3.25986	4.665453	3.22015	3.361896
ILKAP	4.13834	2.557866	4.19354	2.454376	3.08642
CDKN2A	2.039445	3.172306	4.669283	3.131024	3.415068
ANP32C	2.171014	5.602177	2.783149	2.810144	3.047979
GSTP1	3.802009	3.149388	3.483972	2.692793	3.048374
GMPS	3.904269	4.159791	3.007207	2.588598	2.444054
RBBP7	2.597623	4.844398	2.437794	3.513227	2.590523
SFXN1	3.357896	3.876133	2.544774	2.179812	3.869542
POLR2L	4.858581	2.500192	3.128831	2.422085	2.892313
SRCAP	2.436201	3.916234	2.518214	3.549004	3.249127
CAND1	3.160578	3.135602	6.565578	2.742497	
ANXA2	2.308101	3.659746	2.700853	2.887266	3.966553
LSM14A	8.398797		1.642354	3.232209	2.166069
RRP9	3.0736	2.589998	3.621747	2.618888	3.381173
NUCKS1	2.016247	4.290982	2.29826	2.650762	3.97859
RPS16	3.218792	3.35739	1.813713	3.196021	3.648465
CDV3	4.673244	3.639074	4.588095		2.297464
LTA4H	2.815766	2.892392	3.123152	2.605819	3.758297
ACAA2		2.724134	4.166083	3.749408	4.55429
SNRPD1	2.776892	3.352057	2.792276	2.74869	3.319625
MAGOHB	1.987006	2.927922	3.102642	4.616539	2.323051
STRBP	3.411098	3.678277	3.605837	4.243541	
U2AF1L4	2.651158	3.085298	3.128173	2.599909	3.442912
TERF2	1.623507	4.378224	2.412714	3.111162	3.357781
DENR	2.589578	3.110343	3.158672	2.206059	3.798974
PDCD5	2.711168	3.370536	2.413948	2.796229	3.505163
NHP2	2.533864	2.560516	2.520298	3.648656	3.400519
NOP10	2.060649	4.05616	1.938515	3.142824	3.405095
PSMA3	2.017553	4.06889	3.505596	3.14588	1.821469
KCTD12	1.901425	3.771931	2.719482	2.728295	3.420602
DKFZp313C1541	3.159809	3.905038	3.35076	3.962476	
RAB2B	3.159809	3.905038	3.35076	3.962476	
TOMM20	2.550239	3.245288	2.597236	2.452746	3.519299
SLC25A13	3.23801	3.382695	2.205765	2.339206	3.181024
SSU72	3.557537	3.097957	2.600004	2.923984	2.12216
KRI1	4.524278	3.83669		2.507333	3.299088
HN1	3.702839	3.502694	3.077645		3.847221
CSDE1	4.636607	3.033983	3.770317		2.688017
IQGAP1	2.123156	3.953605	2.301094	5.605098	
RANBP3	3.265385	3.449178	2.209416	1.777415	3.281335
CHORDC1	3.321023	3.302629	2.795494	2.221963	2.316037

ATIC	2.630077	2.766063	3.388891	2.269906	2.89886
NSF	2.024865	4.62273	3.971449		3.331813
TPT1	3.144038	3.478618	2.276382	2.254789	2.753835
TMX1	2.451503	2.554154	2.822177	2.948004	3.108268
THOC2	3.489708	3.496777	3.505909	3.342624	
RPL9	2.252475	4.258341	2.80875		4.503358
PABPN1	3.075848		3.005544	3.910261	3.798488
CALM1	2.243569	4.14236	2.37897	2.954063	2.069066
PRPSAP2	3.077188	2.422165	2.700893	2.202787	3.381976
PRDX4	2.627755	2.508921	2.252693	2.669105	3.692437
C1D		3.28747	3.038958	3.840286	3.575711
HIST2H2AC		2.733706	2.083622	6.078214	2.783803
HSDL1	3.56927	4.081143	3.603531	2.397403	
EEA1	2.85673	2.62576	2.973406	2.73763	2.421728
EDF1	2.401201	3.337021	2.380935	2.264412	3.1695
PRDX6	2.872932	4.016294	3.05323		3.566438
KLF12		3.011002	2.833759	2.274424	5.329515
KLF3		3.011002	2.833759	2.274424	5.329515
KLF5		3.011002	2.833759	2.274424	5.329515
KLF7		3.011002	2.833759	2.274424	5.329515
MYNN		3.011002	2.833759	2.274424	5.329515
ZKSCAN7		3.011002	2.833759	2.274424	5.329515
ZSCAN16		3.011002	2.833759	2.274424	5.329515
ZSCAN2		3.011002	2.833759	2.274424	5.329515
NMT1	1.932869	4.575278	2.209692	1.927036	2.714857
HADHB	4.171443	2.590638	4.564615	6.058642	-4.07987
AHSA1	4.264408	6.039251	-2.17585	5.175856	
ABCF2	2.759438	2.598108	2.845067	2.699784	2.391323
ZC3H14		3.79447	2.767172	3.39016	3.338645
PLD3	3.286029	5.564812	3.587991	2.708124	-1.90836
BRD3		2.670045	4.038248	3.37489	3.008545
BRD4		2.670045	4.038248	3.37489	3.008545
PSMD7	1.545583	2.870938		5.040378	3.569159
RPS10	1.36594	2.068691	2.635922	4.785406	2.155432
ILF3	2.052741	3.563401	2.248815	2.551532	2.465893
AP1B1	2.388375		3.026677	3.517103	3.935037
TOP2B	4.057183	3.388927	2.647692		2.767654
ATP2A2	5.130864	3.4916			4.231958
ANP32A	2.807105	2.850172	2.17328	2.199548	2.798892
EXOC3L4	2.37739	2.386889	3.484739	2.351166	2.213082
PEBP1	3.273944	3.194521	2.476725		3.862371
NAP1L4	4.52622		3.119953	5.121954	
MGEA5	2.911222	3.078775	1.826328	2.369549	2.559235
USP34	1.735766	3.563102	3.163682	2.11946	2.142424
STMN2	2.481092	3.505233		2.732641	3.946908
NUP205	3.59716	3.948217	4.011751	3.431362	-2.33282

CDC37	3.770923	3.077037	2.712773	3.074254	
SUB1	2.300934	3.356013	3.479484		3.439905
PGP	2.84083	2.91989	2.143372	1.975463	2.667839
ADSL	2.517647	2.556399	2.701169	2.399824	2.316947
HIST2H3PS2		3.990903		4.752464	3.734658
RANBP1	4.90823	2.528558		1.937098	3.090749
SRSF9	2.361875		2.755881	3.080814	4.263432
CAD	2.682196	2.644532	2.893146	1.851236	2.342075
COX15	3.338216		2.732697	2.510183	3.813923
YWHAH	2.089552	3.636871	3.743545	2.875597	
HIST2H2AA3		2.733706	2.083622	4.715684	2.783803
BAG6	3.386025	4.516488	3.491089	3.374618	-2.4849
UBE2A	2.149374	3.820374	3.532983		2.745289
HPRT1	2.003431	3.622363		2.835459	3.756227
DYNLRB1	2.232533	1.400357	2.021666	2.199424	4.359371
PKN2	2.880802		3.065371	3.08492	3.176683
AFG3L2		4.464799		3.487026	4.228582
SMNDC1	8.398417		1.918355	1.851577	
TRIP13		2.699176	4.053877	2.308015	3.098892
RBM4	2.161582	2.93607	2.63853	2.561265	1.856834
NVL	4.385069	4.05471	-4.97567	4.065401	4.613088
GNB2L1	2.348412	2.589572	2.593452	2.824897	1.783433
BLVRA	3.132613	3.233212		2.617496	3.08048
NPEPPS	3.345038	3.752324	2.212608	2.704853	
PSMB1	3.184728	3.152704	2.929735	2.612459	
CHCHD4		3.589819		4.102385	4.160435
SDHA		3.395392	3.77281	4.663784	
DDX42	1.614998	3.018263	2.059933	2.264568	2.871579
DBNL	2.477241	2.689188	2.181023	1.799021	2.682106
WDR6	2.815243	3.816346	2.16487	2.989692	
UBL4A	2.872313		2.711075	2.902186	3.283377
MSH6	2.762846	2.957977	2.896963		3.017527
TPD52	2.30848			4.505419	4.80179
LDHA	2.273628	2.898453	2.46049	2.15447	1.798486
CUL4B	3.154114	3.013078		2.628965	2.710734
DDX51				6.285229	5.18558
DUT	2.43142	3.879403		1.67067	3.485811
AKR1B1	3.419202	3.869911			4.167174
UCHL1	2.431931	4.250201			4.773927
P4HB	1.518037	3.485762	1.167048	3.073572	2.195795
FLNB	2.663663	2.754046	1.543499	1.975149	2.407077
EIF4A1	2.112437	2.434614	2.537098	1.611386	2.6457
NNT	4.259772	5.234429	4.391215		-2.61283
TMSB10	2.358047	3.15616		2.113698	3.636859
ZYX	3.877549			3.453583	3.900944
ZC3HC1		3.914305		3.584355	3.730338

NUDC	2.978481	2.655575	3.02655	2.557965	
SKP1	2.783513		2.763657	3.245967	2.423788
STARD13	2.630626		2.984814	3.636994	1.929293
PRMT1	2.142419	2.22586	2.010731	2.329166	2.443066
FKBP8	4.383028	3.488797	4.267455	2.382108	-3.37283
ATAD1	8.39645			2.62197	
SDF4	2.490026		2.78236	3.415768	2.317722
NAE1	2.288575	2.383351		2.826385	3.505206
LIMD1		3.663601		3.801876	3.513859
NUDT5	1.554188	2.607565	1.868897	1.852188	3.08241
CTPS2	5.507572	5.451168			
SPTLC1	2.486707	3.604172	2.520369	2.338684	
RAB6A		3.447894		2.750617	4.736682
CANX	3.60814		3.494015	3.812525	
ACTBL2	2.89498		3.911076	-2.47568	6.579158
H2AFV	1.593994	2.451433		4.183837	2.678711
DDX52	8.395844				2.502621
PSMA8	2.054712	3.637178	3.052534		2.127227
ILK	4.231647			3.172317	3.458382
PSIP1	7.232805	-3.50772			7.136455
TDRKH	2.751986		2.527383	2.715188	2.820129
POLR1C	3.126978		2.681277	2.644198	2.331464
TPX2	2.934783	2.561411		2.611755	2.665921
DSG1		5.979821		2.708999	2.045148
GARS	3.973393	-5.1418	3.990592	4.839776	3.031165
RAB6B		3.447894		2.633126	4.602091
H3F3C	-2.75761	3.990903		5.689182	3.734658
CRNKL1	3.096827		2.352975	3.093123	2.05168
GPS1		7.063828	3.512519		
RBBP4		2.951659	2.043936	3.446305	2.121506
ESD	2.936422	2.585656		2.271308	2.731271
NCBP1	2.839423		2.479855	2.659017	2.522733
MTHFD1	2.933236	3.364453	1.966768	2.130307	
DBI	2.585513	2.694352		2.099327	3.007038
MRPL48	2.150361	3.124901	2.539726	2.550286	
MPHOSPH6	2.446221		2.333557	2.775983	2.753292
ACTR2	2.33097	2.907762	3.106009		1.908381
TSPYL1		4.12942	2.117655		4.002969
TIMM13			2.693907	3.364056	4.161816
NSF		4.198847	-2.13943	4.490125	3.667606
BCAS2	2.825456		3.756229	3.634624	
HACD3			2.746909	4.128474	3.335625
PFDN2	2.941764	2.868864		2.044244	2.33969
COX5A	2.419497			4.391836	3.343177
PDIA3	1.999607		2.751754	2.838457	2.446141
FAM96B	2.238979		2.466421	2.812071	2.501653

ATF1	2.544336	2.539029	2.654205		2.245269
CREB1	2.544336	2.539029	2.654205		2.245269
CREM	2.544336	2.539029	2.654205		2.245269
RGS10	2.78774	2.96248	2.127421	2.082464	
GLO1	2.543639	2.627275		1.79655	2.967073
NSFL1C		2.931847	2.492627	1.815971	2.67758
HSPB1	8.395398		2.782095	2.552177	-3.8192
ALCAM	1.761659	3.420197	2.247822		2.417631
MTA1	1.622628	1.155342		1.597278	5.446669
STRAP	2.467938	2.649202	2.640798	2.001073	
GLYR1	4.547577		3.314392		1.889903
NIP7	2.100753	4.442407	-2.22542	2.195083	3.189934
EIF1	2.369642	2.636721	2.48903		2.201114
RRM1	2.327804	1.518488	1.545126	2.177119	2.123231
ATP1A2			2.322769	4.28073	3.06016
PPP2R1B		2.709558	1.616749	2.719097	2.602009
PFKP	3.171084	3.09114			3.290698
GBAS	2.041613		2.237197	2.837679	2.401106
GART	3.024337		2.43422	2.121082	1.915489
RPL38	2.328322	2.800782	1.950077		2.415705
DYNLL1	2.417682	3.007906	2.003058		2.007872
ANXA5	3.476821		1.864747		4.081206
TRMT6	2.405282	2.789246		1.889109	2.337332
GSTO1	3.12412	3.250886	-2.72243	2.190064	3.541855
PSMC2	1.933995	1.355187	2.211635	1.554068	2.315774
PPP4R2			2.905439	2.930947	3.485517
AKR7A2		4.006895	2.149501	3.14305	
PPIF		2.83708	2.381816	4.037908	
PTBP3	2.255026		2.193605	2.727708	2.072868
PABPC1	3.376748	3.159149	2.579701	2.9656	-2.85646
NUP50	3.44336	2.277269			3.482992
HINT1	2.84078		1.408848	2.270888	2.674395
ALDH1B1	2.727469			3.086537	3.368817
DPY30		2.9679		3.061497	3.137028
SART3	2.044567	2.726008	2.262118	2.105084	
MAP1S	2.48879	2.618711	1.972609		2.046283
VRK1	2.106812		2.177151	2.355274	2.442467
PSMA5	1.957143			4.056486	3.056311
HBS1L		2.322189	1.60252	2.116446	3.003525
HN1L	2.666766	2.430175		1.88859	2.036972
RBM26	2.15901	-2.75517	3.001943	3.444488	3.144235
CALM2		4.14236	2.515178	2.228614	
KDM1A	4.40447		4.438439		
CSTB	2.761996		2.598097	1.623387	1.839498
TIMM10B	2.58872		3.096328	3.107261	
RPL22	2.860579		2.581148		3.348452

RAB11FIP1		2.641627	3.469086	2.678778	
SMC4	2.875315		2.669424	1.960001	1.252565
NRAS	2.910918			2.962181	2.870967
ANAPC7	2.82991		2.982728		2.908205
TFG	2.565857	3.504833	2.649044		
CA2	2.870022		2.295799	1.833711	1.718721
HYOU1			2.750564	2.841358	3.115781
ARL3	2.062439	2.208102		1.794842	2.620009
TMA16	2.495131		1.950916	2.109972	2.115403
PSMC3	2.874807	3.243435		2.506897	
CTNNB1		5.219114	3.403984		
RAB10	1.929802	2.904431		1.841796	1.939974
IWS1	2.580707	2.559899			3.364823
PYCR1	2.341481	3.473145			2.662687
RAB5B	2.637897		3.020582	2.773151	
RPS15A	2.232466	2.609811	2.594986	-2.02694	3.014615
STX18	8.39644				
TOX4	8.395991				
FDFT1	8.395719				
RIC8A	8.395663				
PDE9A	8.395463				
FAM21D	8.395418				
RNF14	8.395376				
HRAS	2.554413			2.962181	2.870967
PLS3	1.780727	2.573552	2.46333	1.569557	
MAEA		2.635836		2.503828	3.234395
WDR3	4.357123	2.050935			1.931114
SIN3A	3.961659	4.372344			
ADNP	2.088494		1.365711	3.057401	1.812595
CPNE3	2.254952	2.291597		1.594939	2.172848
ARHGDIA	1.802579	1.656896	2.485199		2.318728
SAFB	2.388361	-1.3745	2.178701	2.131884	2.907055
TNPO1	2.641196			2.069971	3.500907
MAT2A	1.858518	2.523125		1.753231	2.075498
UBTF		2.775355		2.623433	2.803179
SNX27			2.795569	2.711297	2.685207
SMARCB1	1.645532		1.956902	2.015545	2.532361
TNPO2	4.066277				4.047242
POLR2B	2.680934		2.764865		2.64382
RBM17	2.205135	-2.2254	2.812746	2.703947	2.584915
PSPC1	2.744088		2.898658	2.429015	
ACTA1		2.833885	2.58068		2.646226
ACTA2		2.833885	2.58068		2.646226
ACTC1		2.833885	2.58068		2.646226
PFKL	3.055048	3.022286		1.980592	
RAB35		2.993561		2.345817	2.698869

GNPNAT1		2.907586		2.61611	2.504529
NDUFS3		3.634004	1.139498		3.24777
PSMD1	2.787046		2.086426	3.123025	
PPP1R8			2.613168	2.660304	2.708897
PDHX			1.674125	3.640508	2.664591
PDE12	2.133018	-2.56032	4.53943	3.85359	
STOML2		5.223988		2.711873	
DYNC1LI1		2.781648	2.636195		2.509732
ZRANB2		3.343242	1.927306	2.635135	
GRHPR		3.392176		2.097906	2.341322
NDUFS2	2.26182	3.080232	2.427733		
SART1		2.596741		2.52564	2.645641
PHKA1		4.328953			3.425716
CPSF6	1.998553	3.034406	2.714343		
ATP6V1A	2.616283		3.914687		1.201298
DHCR7	2.814599		2.4997	2.402	
ABHD10	4.413496		-1.69968	4.955009	
WBP11			2.564058	2.540589	2.535269
ZNF740	3.652506	3.932609			
PAK1		3.583922			4.001088
CUL4A	2.822311	2.557187		2.192953	
MRPL15				3.587739	3.977276
MYL12A		2.558908	2.996801		2.007677
MYL9		2.558908	2.996801		2.007677
NACA	2.736439	3.312098	1.50349		
NACAP1	2.736439	3.312098	1.50349		
BLVRB	2.613873	2.691149			2.246742
PCMT1	2.316211			3.081548	2.10608
NAP1L1	2.17548	3.101041			2.195262
STAG2	2.321941		2.577996	2.551638	
SCAF4	2.225364	-2.49679	2.292259	2.153716	3.239632
CDC27				3.722954	3.671455
NDUFS5		4.017659			3.362489
UQCRC2	2.26353		2.066224	3.029847	
STX12			1.953191	2.78968	2.590861
WDR5		2.575757	2.991702		1.747565
RAB2A			3.35076	3.962476	
BPTF				3.211157	4.071586
TMEM201	2.151654		2.131159		2.998632
C12orf43		3.361569	3.543783	-2.78515	3.081793
ACAT2	2.076233			1.947391	3.170913
EIF4H	2.794319	2.41855		1.973526	
TRAX	2.109011	2.937282		2.136881	
EHD4	2.489073	2.69431		1.984306	
ATP5A1		4.529002		2.626821	
CCDC124	2.154575		2.730384	2.260632	

TP53BP1			4.113898		3.021948
MTX1	2.394999			2.656181	2.045594
RAB5A				4.87823	2.154035
FHL1	3.325759	3.400726	3.884692		-3.58849
HMGN5	2.602247			2.295152	2.120885
LMNA	2.9199	3.662488	-5.72582	3.782661	2.318342
SOD1	2.48908	2.681171			1.767386
TPM4	2.154613			2.231268	2.526874
SUPT5H		2.606333		2.052731	2.242365
SERPINB6	1.701367		2.574172	2.623402	
OSBP	3.503244	3.39253			
CNPY2	1.692812			2.535902	2.658029
KHSRP	2.337543		2.334367	2.18284	
TUBA4B	1.969895		2.757484	2.125315	
NOP58	-1.21251		2.190343	3.757845	2.102168
RGPD8			2.790729	1.993185	2.044556
SMS	2.342579	2.89901		1.570894	
PDCD6IP	2.736209	2.827672	1.23835		
FAM192A	2.19678		2.101662	2.502472	
NIP30	2.19678		2.101662	2.502472	
PGRMC2	2.008998	-2.21803	2.229268	2.424186	2.334954
TECR		3.335086		3.398467	
PAFAH1B1	2.375067	2.310399		2.002867	
PSME3	1.675126	1.972155	3.035634		
RPS15A					6.675799
CALM3		4.14236	2.515178		
UBE2D3			1.529338	2.567621	2.527655
PPP1R12A		3.297794	2.374605	2.699813	-1.75707
DOCK7	2.418229			1.939902	2.249794
RCC1	1.954594		1.902196	2.737198	
ENOPH1		3.275681		3.281413	
ADH5		4.438559			2.107903
CERS2		2.302802		4.221438	
BOLA2B	1.949802		2.531919		2.030146
PRPF4B			2.03932	2.298579	2.169365
PSMD2	2.575998			1.913183	1.991732
HSPH1	1.861151	2.481301		2.136592	
SEH1L			3.148777		3.291323
PSMC4	3.62412		2.79761		
MRPL54	1.979787			2.264255	2.176214
IGHA1				6.412257	
IGHA2				6.412257	
ALG10		3.660608			2.749062
NDUFS8				3.055673	3.338904
MRPL4				6.368657	
UBXN1		3.527064		2.838908	

PSMA2	3.189391	3.625871	3.235108	-3.68476	
CAP1	2.493121	-3.17587	2.35188	2.716527	1.979327
USP11			4.007856		2.338879
USP4			4.007856		2.338879
COX17		4.138176		2.176945	
PPP2R2A	2.009355			1.916157	2.386878
NENF		4.063449	2.228286		
TFCP2		3.1238	3.154542		
UCK2	1.61479	1.611933	1.854843		1.19442
GRPEL1		3.194287		3.074856	
RBMXL3	2.045071		1.924122		2.285528
IMPDH2	2.106161	2.519863	1.882199	-2.30186	2.026422
RPS19		4.045033	-2.30321		4.455749
RPIA	2.937837				3.250943
PIN1	2.208864			1.713509	2.240623
ANP32B	2.161234	2.40423		1.576709	
IK	2.841029	-2.64044	1.837014	1.870852	2.203195
HIST2H2AA3				6.078214	
IKBKAP		2.322042			3.750714
MRPS22		3.915969		2.155555	
GNL3L	1.872913			2.058355	2.092061
HMG20B				3.038872	2.902378
FERMT2	2.110109	2.277672		1.52274	
HIST1H2BB				3.775531	2.131237
HIST1H2BJ				3.775531	2.131237
HIST1H2BO				3.775531	2.131237
HIST2H2BC				3.775531	2.131237
HIST2H2BD				3.775531	2.131237
HIST2H2BE				3.775531	2.131237
HIST3H2BB				3.775531	2.131237
RPL3L	2.500626		3.394207		
ATP6V1G1	1.704632			2.084276	2.070331
SUPT6H				2.52148	3.310968
HTATSF1			1.271637	3.434256	1.124999
PHF10		3.305897		2.50985	
YWHAH		3.409867	2.333325		
TUBB	4.624252		1.116309		
NPEPPSL1		2.245471	1.864875	1.628121	
UBBP4	2.932834		2.78376		
CNOT1		2.811339	-2.58404	2.725698	2.745248
TMEM11		3.429974		2.215355	
CSTF1				2.978231	2.65747
RRP8	-1.70817	4.402776			2.926458
SDF2L1			3.044193	2.573121	
USP5	2.758499	2.834105			
CIRH1A		2.703142	2.87378		

WARS2	1.617425			1.919252	2.003444
C7orf50				2.674377	2.84751
CPSF4				2.126074	3.356246
MBD3				5.476899	
ALDOA	3.534905		1.90478		
NDUFAF3	1.924381			1.6416	1.872009
ALDH3A2			2.556438	2.881385	
BID				3.21055	2.225922
TXNDC17	2.625698	2.797193			
ACTG2		2.833885	2.58068		
EIF2S1			1.6592	1.781232	1.970866
ARL6IP1				2.28348	3.061888
RPP30		3.162947		2.175497	
MRPL28	2.89524	2.435261			
SRP72		2.419086			2.911212
ATP2B1	2.2214	3.097474			
ATP2B2	2.2214	3.097474			
PSMD3		2.901293		2.399148	
EIF4G3	2.666532		2.630139		
TFAM	2.850899			2.391975	
HMGB1			2.053238		3.185158
CIAO1			2.643759	2.580158	
AASDHPPT		3.698679		1.525075	
ATP5J		2.302903			2.894686
HSPA8	2.535244	2.767095	1.982832	-2.10432	
MDH1	2.024572				3.123632
HNRNPUL2	2.337714			2.810361	
DNAJC8	2.775094				2.360196
IGF2R		5.019741	2.288653	-2.18229	
MTAP				2.186789	2.92956
TBCB	2.823318	2.290626			
H2AFZ	-2.48235	2.451433	-1.54095	3.994732	2.678711
AK1	2.498222	2.590154			
PITRM1		4.302467	-5.89462	3.160042	3.518041
RPS27L		3.586871		-1.85918	3.352327
TPM1	2.048929	3.011784			
WAC		3.087276			1.966984
DISC1	2.109011	2.937282			
TSNAX	2.109011	2.937282			
ZC3H13			1.737082	3.297638	
CSNK2A1		3.155441	1.87473		
BRIX1			1.222172		3.770992
EIF4EBP1	2.012551	2.974014			
ME1		2.477381		2.508415	
CAPNS1	2.211067		2.743208		
PNPO				2.367809	2.570797

ABCE1				2.435538	2.501304
GOLGB1			2.234753	2.696459	
MAPRE1	1.791286		3.121137		
ALDOC		3.591254	1.291011		
THOP1	1.83052	3.0669	1.994517	-2.01548	
RPSA	2.881093		1.988933		
RPSAP58	2.881093		1.988933		
DHX16	2.101774				2.768185
PFDN1	2.42461	2.441221			
FAU	2.041721	2.805489			
AKAP1		2.829234		2.011758	
RGPD1	1.732433	1.693463			1.362749
PTPN11				2.256195	2.514812
QARS	1.311078	1.369517			2.080461
HSD17B4			2.48396	2.26284	
MYH14		2.91099		1.83238	
GTF2B	1.651819	3.085004			
RBM4B			2.012748		2.704939
FLNA					4.705229
DYNLL2				1.914272	2.783023
ENO2	1.988551	2.703322			
ERO1L	2.105464	-1.49414	1.902549	2.165832	
SNRNP200			1.844081	2.831406	
ZC3H15			2.757377	1.855163	
LIN7A			2.458262	2.149582	
VCP			2.272231		2.321088
APP	2.310159		-4.00934	2.624403	3.665709
TMED9		2.547931	2.033713		
KIF5B	2.305372	2.270194			
ACTN3	2.252729		2.320214		
IDH3G				2.446435	2.121665
TBCE		2.205085			2.339361
COQ9	2.333146			2.18664	
S100A8				4.487279	
CBX3	2.15162				2.327079
EPHX1	2.42765			2.040366	
RPS6			2.411445	2.04335	
DSP	4.434354				
SLC25A12	2.405652		2.026509		
MSH2	1.411942	1.427824			1.584785
ERCC6L	2.299655				2.089868
PPME1				1.582711	2.783411
XPO5				2.242301	2.119539
SMARCA5	1.906775			2.434429	
LCP1			2.250931	2.090113	
ITGB1	1.567502			2.770795	

LAGE3	2.554402			1.78	
NUP153	2.100429				2.220596
TCERG1	1.11115		1.651533		1.556375
GAPVD1	4.297182				
SF3B5				1.882338	2.406396
SMC1A	1.250903	1.348833	1.667524		
PGK1	2.342255				1.91809
ASF1A		4.260295			
DAP					4.253544
NCAPH	2.622689		-2.64633		4.26361
TTC4				2.046218	2.192495
DYNLRB2			2.021666	2.199424	
SMAP1	2.648268			1.562437	
DCTPP1	1.799434				2.404791
DDX39B			1.660506		2.541536
ATP5I	2.209109				1.989552
ARPC4	1.716356	2.463381			
NANS	2.025002				2.132202
TTLL12	2.391918			1.752711	
MRI1	4.136177				
HIST1H2AB				4.125427	
HIST3H2A				4.125427	
CDK4		3.111454	1.008909		
EXD2			1.955702	2.16254	
GPATCH4	1.146351		2.907607		
KCTD8	1.628728			2.411947	
HPS4	4.002302				
ACIN1				1.894929	2.097997
CPT2	2.606142		2.640206	3.010849	-4.26801
CKB	2.859572		1.099887		
PRMT8			1.850692	2.088374	
IDH1	-3.3467	3.180389	1.80409		2.286727
RPS27	1.88114			1.999324	
PPA1	1.871631			2.00716	
BANF1	1.706804				2.151884
KCTD5			1.956284	1.893866	
HAT1	2.655587	2.98603	-4.77386		2.920157
RBM25			1.927971		1.848004
DNAJB6	1.701296			2.06817	
NOP56				3.753756	
IK					3.731015
PLOD2	1.718174		2.000222		
CTTN	-4.30125	-3.56782	2.866922	4.860484	3.817058
UTP18				1.805531	1.869702
ALG10B		3.660608			
IDH3B					3.656461

BCL2L10	1.669657		1.974647		
TSN		2.204109		1.432603	
CTSD	2.764534		2.184228	1.633458	-2.96246
LUC7L	2.14825	-3.20381		2.646635	2.012103
GTF3C5	3.601875				
SLC3A2		-3.97392	2.14787	3.280224	2.145836
DLAT				3.583364	
NEDD8	1.710424	-2.54069	2.308731		2.099346
TUBAL3				1.486187	2.070702
VBP1		1.757927	1.79784		
NASP	1.579377	1.254726	1.565387	-2.11977	1.269363
PGAM5				3.532695	
SF3B2	1.581034			1.940455	
NOL6	-2.55517	3.279881		2.781378	
APEH	1.921841				1.583877
GEMIN7		3.502426			
MAGED1	1.546411		1.942262		
EEF1E1-BLOC1S5		3.488351			
UBE2R2	1.658584			1.828429	
ZNF346					3.468571
CBX5				3.463858	
ELMOD2					3.452888
RFC2	1.818761	1.629611			
SNX3				1.423574	2.022859
PTGES3L-AARSD1		3.436269			
LAMC1			3.42987		
PDCD2L		3.42431			
CHCHD2		3.419457			
CHCHD2P9		3.419457			
DDB1		4.282996	1.675605	-2.54819	
ROCK2				3.410404	
ESF1	1.533084				1.866348
PSAT1	3.387672				
IGF2BP2				2.149886	1.234563
TCEB1			3.377493		
HDAC6			1.776288		1.597609
SEPT2					3.360739
CKM					3.357243
OMA1					3.346786
TOM1				3.336384	
PPIE				3.317107	
SREK1					3.283596
FAIM		3.276535			
ACADVL					3.273028
CISD2	2.705118	2.869896	2.009394		-4.31148
CFL1					3.271146

ALDH6A1	3.265312				
MAFF				3.248084	
YARS		3.244497			
PSMD11		3.198749			
MRPS12		3.192674			
ACOT13				3.184412	
TOP2A				3.179468	
CSNK2A3		3.176461			
CCDC86			1.070258		2.100729
UBP1			3.154542		
NUP88				3.152366	
POLA2			1.216492		1.925312
LDHC					3.10659
MYO1B					3.102331
ERCC1	3.101348				
ATP2B3		3.097474			
ATP2B4		3.097474			
RBMXL2				3.086916	
RGPD2		1.693463			1.362749
NRD1		3.055555			
TPM4					3.030962
STK24		3.028067			
STK25		3.028067			
TPM2		3.011784			
LDHAL6B			2.990718		
UBE2N					2.980724
TXNRD1		2.956062			
TXNRD2		2.956062			
NARS				2.941801	
MYH10	1.556372	1.367202			
CCDC137				2.878167	
TOP1MT	1.105843			1.761203	
WIBG				1.47246	1.392807
HIST1H4A				2.863412	
MKI67			1.527097		1.311735
RPL18A		1.367772			1.468623
UAP1					2.822279
SEPT9					2.814901
XPO7		1.381979			1.420289
DARS	1.134166		1.663665		
SLC9A3R1			1.582212		1.205664
LAMTOR5		2.730291			
AURKB			2.717967		
TFG		2.675364			
COPB1			2.664215		
GANAB					2.659805

ECM29				2.645276	
IARS	2.031098	2.638719		-2.02828	
C3orf17					2.631293
CDH2		2.631163			
CYB5R3				2.61453	
RALYL				2.587561	
ZNF512					2.586006
DCD					2.580951
CCAR2		2.569742			
PPIL4					2.551956
GLUD2				2.535096	
DHFR					2.528393
EIF1B	2.526719				
CALM1			2.515178		
OAT	2.510232				
GOT2				2.484841	
MGA				2.473859	
MRPL53				2.473469	
HEATR1				2.438567	
TUBA8	2.417177				
SEC11A				2.416614	
TOP2B					2.406301
PITPNB					2.401744
NDNL2	2.389726				
NAPA				2.356112	
ADK					2.347189
RPS20					2.342137
PDCD10					2.333198
NUP54				2.332131	
CPSF3	1.123438	1.207329			
ATP5L				2.328549	
HOGA1		5.126284			-2.80046
PSMD6		2.317931			
ILVBL			2.309169		
HMGB2					2.304626
PRDX1	2.303121				
PPP1R10				2.299803	
TUBB6					2.29837
CKS2					2.286614
RPLP0	2.273211				
STIP1	1.106559	1.164233			
PDHB			-1.05296	1.606184	1.70882
COG4					2.25602
SNRPA1					2.251942
PSMA4	2.328312	2.331153	1.218382	-3.62694	
QKI			2.247704		

TES	2.245258				
LRPAP1					2.242278
hCG_1789329	2.234425				
UBE2L3	2.234425				
USP7					2.218452
DAZAP1					2.177688
PSMD10					2.170099
EIF2B2					2.168518
DDOST	2.159271				
IDI1				2.156117	
TAGLN3	2.148493				
PFDN4				2.145523	
TMF1					2.132511
TUBB8				2.128161	
KIAA1033	2.097702				
DHX33	2.096235				
EIF2A		3.045419	1.551684		-2.51465
INCENP				2.078357	
ANP32E	2.072497				
LDHB	2.071007				
HIST3H3				2.064585	
TMEM9				2.054093	
PFDN5				2.052757	
TUBA4A			2.042978		
CCT6B				2.028045	
MRI1				2.01823	
EXOSC2			2.015669		
FDX1					2.010488
MKL1					2.000062
MIF	1.998038				
PRCC			1.997925		
NDUFB10	-1.11476			3.112591	
NAA50					1.973319
KTI12				1.964778	
EIF3D			-2.89	2.323016	2.529778
ZNF598				1.962391	
METAP2					1.923882
NUFIP2				1.920692	
CISD1	1.91902				
POLR2A	2.577901		-2.49721		1.835639
SEPT8			1.912634		
SEPT11			1.912634		
SMEK1				1.901475	
PSMD14	1.901163				
BAZ1B			1.894201		
LDHAL6A	1.789159	-3.01609			3.10659

FBL					1.876947
PFDN6				1.865474	
MARCKSL1	1.865082				
PITHD1				1.864952	
PPP1R12C	1.863474				
HIST1H2AC	1.861164				
NDUFA4	-1.24583			3.105566	
AIMP1	-2.25539	3.074346	-2.31634	3.356424	
RNF40	-1.63349				3.472232
CUL1	1.828719				
IMPA1				1.825188	
TTC9C	1.821079				
TBCD				1.81254	
UHRF1				1.806173	
MAPKAPK2	1.798719				
MAPKAPK3	1.798719				
PDCD6	1.783259				
TXNL1				1.764343	
POLD1	1.763066				
FMR1	2.987731	3.420831	2.589099	-2.53209	-4.71424
PFN1	1.743875				
VPS36	1.743385				
C10orf71	1.731166				
NOB1	1.729767				
MRPL32	1.710582				
ANKHD1	1.710544				
CTPS1	1.704521				
DHX36			1.697293		
APRT	1.686382				
SUZ12	1.685997				
CCDC51			2.237144	-3.81064	3.233808
FKBP1A	-3.57973	3.971498	3.326964	-2.06275	
FAM207A	1.652359				
UBA1	1.647073				
DIAPH1	1.633387				
ARPC5L			1.632602		
CBX8			1.61811		
KIF4A					1.599431
PRIM1	1.597759				
EIF3A				1.592466	
SEC23IP	-3.10191			2.242155	2.451845
FXR1					1.580384
SGTA			1.575902		
COX5B	3.780925	-2.20824			
CARHSP1			1.57065		
CHD3				1.562453	

FAM98A				1.557329	
PM20D2	1.554792				
DNAH17					1.548303
EARS2				1.513715	
PPP2R2A	1.511792				
MAD1L1				1.509956	
C19orf47				1.505053	
POTEKP			1.504923		
LRRC47		1.488033			
SCFD1				1.472808	
PTBP1	-3.70981			2.716732	2.451078
FXR2					1.445578
YME1L1	2.878041		2.931416	-4.38153	
EEF1A1			1.402572		
HIST1H2AA	2.666481		-3.36735		2.092809
HIST2H2AB	2.666481		-3.36735		2.092809
RPS10P5		1.374198			
TRMT112					1.364311
HSPA9			1.335724		
G3BP1	1.290525				
SLC25A6	-1.16807			2.45634	
EIF5AL1			1.276704		
RNH1	1.271124				
EMG1	-2.02582	1.805193			1.483146
DCP1A			1.192596		
PKLR					1.155769
COTL1	1.148505				
HSPA4L		3.456945	-2.31391		
TUBB4B	1.141674				
ACTG1			1.134789		
DNAJC9					1.128284
TUBA3E			1.110002		
GTF3C1			1.093405		
POLR1A			1.088984		
TRIM28			1.076259		
RBM12B	4.264436		-1.02965	-2.16828	
EIF4E		3.139668		-2.07665	
ZNF280C			1.058779		
VAT1			-1.14081	2.183554	
EDC4			1.037882		
ACAT1		3.012769		-1.99768	
UTP14A	1.543426	-2.95243			2.36726
HNRNPK	1.607343			3.125805	-3.77508
FIP1L1	2.227989		-2.95753	1.609962	
PGLS	-2.49142	3.325103			
POLR2C	2.819616			-4.06338	2.066271

SDHB	2.094945		-1.28684		
PSMD2	1.142714	1.114172	-2.77287		1.218004
FDXR		3.204603	-6.12944		3.569308
TMEM109		-4.65048		1.583293	3.660171
CENPV	-2.31943	1.195371			1.713292
DDX27	-3.1148	4.658491	-3.22569	2.207918	
ATP2A1	-2.25179	2.757501			
PPP4C	1.822557			2.451841	-3.78508
HSP90AA2P	2.035183			2.69993	-4.27412
MYL6	2.523746	-4.42517	-2.05638	2.022944	2.364625
FAR1			2.145449		-1.74231
TM9SF3		-2.42335	2.810339		
GOLIM4	-1.98079				2.353958
PHB	-1.37903	-1.10556		2.830857	
POLR2H	-2.29213		2.606262		
PPP2R2C	1.511792			1.916157	-3.11802
MORF4L1	-2.4343	2.730774			
C5orf24	8.395375	-4.25919			-3.90998
DDX23	-2.14659				2.202271
NDUFS1		-2.23322			2.272472
ME2		-2.61488	-2.01857	2.436454	2.221229
PHF3			-1.95916		1.951484
PRRC2C	2.79258		-3.01498	2.843893	-2.65192
AKAP8		-3.48758			3.432349
PRKAG1			-3.12308	3.030451	
ANXA7	-3.08853	-2.58288		2.082532	3.42387
PAK2			-3.75914		3.578705
PPP5D1	-2.33335	4.017922	-1.90666		
EIF3I			-2.59065	2.226455	
SRSF5	2.704261	-3.13381			
POLR1E	1.666938				-2.16166
MTHFD2	-2.65982			2.079437	
CAPN1		-3.02697			2.445969
CCT3	-2.61403			2.007019	
CBR4		-2.60098		1.989354	
SMC2		2.70295	-3.31502		
GALK1			-2.51655	1.890616	
SLC7A1	-2.57785			1.939308	
NCKAP1		-2.67451		2.022791	
PYGL	1.804683	1.732025		-4.2291	
ACOT7	1.685864			-2.37895	
RPL6	2.4604	-2.73123	2.489208		-3.07829
TCEA1		-3.12765			2.178464
PRKDC			-2.57109		1.577578
DKFZp686E0752	-2.86777		2.100582	-2.12242	1.888466
NUP93		-3.01937			2.008665

PPP1R14B			-1.01396		
PPP5C	-2.33335	3.218278	-1.90666		
RUVBL2			-1.04516		
RB1			-1.06257		
SF3B4			-1.09069		
ATG3			1.94484	-3.03867	
DDT			-2.90901	1.814752	
DDTL			-2.90901	1.814752	
SNRNP70	1.826642	-5.57603	3.11858	2.169871	-2.64592
UQCRC2					-1.1091
HSPA13	-1.11566				
SPTBN4			-1.128		
CAMSAP3	-1.14555				
LMNB1					-1.14797
STAMBP			-1.17408		
SEC24C	-1.17618				
NKTR			-1.19658		
MCL1	-4.27492			3.074545	
ERLIN2		-1.21726			
GSR			-1.22906		
LYPLAL1			-1.24517		
COPB1			-1.24529		
TMLHE			-1.27025		
RNPS1					-1.2714
PFDN5			-1.27554		
CGGBP1	-1.28207				
PRSS1		-1.30976			
PRSS2		-1.30976			
PRSS3P2		-1.30976			
CWC15					-1.32896
SPG20					-1.33557
CDC45			-1.40076		
TRO			-1.4076		
PDCD11	-1.41131				
PPP6R1			-1.4252		
PHC2			-1.42766		
MARK3			-1.44908		
MARK4			-1.44908		
NDUFS4			-1.44972		
HCFC1					-1.48259
C19orf52			-1.4936		
RAP1A				-1.5022	
OSBPL11	-1.51357				
GCN1L1				-1.51573	
ACTR3	-3.30561			1.761343	
RGPD5			-1.55343		

ALDH1L2				-1.55708	
DNM3	-1.56321				
NSUN4			-1.60201		
NOMO3	-1.6223				
TMED2	-1.62377				
AIG1				-1.63224	
RING1					-1.70282
CSTF2					-1.7048
PHF23			-3.35671	1.614554	
DCXR	-1.7558				
TTC7A			-1.75771		
ZFR	2.735266			-4.4941	
PMPCB					-1.76768
HIBADH	1.951777			-3.74184	
MAP4				-4.03182	2.212317
ICT1			-1.82779		
DECR1			-1.83704		
POM121				-1.85735	
POM121C				-1.85735	
RGPD3			-1.87203		
RGPD4			-1.87203		
RPL39			-4.28311		2.406652
RPL39P5			-4.28311		2.406652
AHCTF1			-1.9028		
MTDH	-1.90429				
MED1			-1.9071		
NACC1			-1.90732		
NACC2			-1.90732		
CHD3				-1.90974	
LLGL1			-6.26829	4.358447	
WDR46				-1.91871	
RAB21			-1.9247		
LYPLA2				-1.92699	
DDX27				-1.92927	
SPTBN2			-1.95511		
CBX2	-1.96467				
ELF2			-1.96661		
RPL27				-1.96694	
UBE2I			-1.96844		
LYRM7			-1.98578		
SHMT1				-2.00473	
RPL11				-2.00553	
GBA	-2.0069				
PDXK		2.768878	-2.16858	-2.61168	
NDUFAB1			-2.01155		
RNF214				-2.01206	

EEFSEC				-2.0158	
PAIP1			-2.01615		
LRRFIP1				-2.01838	
ELAC2			-2.02737		
CUTC				-2.03018	
FAM49B				-2.03267	
CTNND1					-2.04058
ASCC2					-2.0573
KRBA2	-2.06098				
HIST1H2BA				-2.0687	
KMT2C				-2.07202	
ATP2C1				-2.07212	
PSMG2	-2.07872				
LAP3	-2.08313				
RNF114			-2.08724		
THOC6			-2.08785		
RPL26L1	-2.09442				
GTF3C5	-2.09759				
HIGD1A			-2.10586		
HIGD1C			-2.10586		
C1orf131			-2.1067		
EMC10				-2.10896	
PPAT				-2.1158	
DDRGK1				-2.12927	
CYB5R3				-2.13262	
AP2B1				-2.13341	
KIF2C					-2.13493
HIST3H3				-2.1374	
LCLAT1			-2.13954		
TMPO					-2.14128
DBR1			-2.1424		
KRT77	-2.14284				
CCDC58			-2.14546		
MAN2B1			-2.14592		
TRMT1					-2.14991
MOGS				-2.15906	
PAPSS1	-2.15919				
PAK1IP1		-2.16245			
TMED7					-2.16247
TMED7-TICAM2					-2.16247
ALDH9A1				-2.16259	
ACAD9		3.09858	-2.15766	-3.10984	
LETM1				-2.17562	
SLC25A5					-2.18192
CDCA7				-2.18226	
ANXA4				-2.19009	

SSR4					-2.19183
CORO7-PAM16	-2.19195				
NDUFA3					-2.19232
SYAP1	-2.21275				
KIF16B			-2.21275		
KIF3A			-2.21275		
KIF6			-2.21275		
MAGOH				-2.21309	
XPO1					-2.21806
ARL1			-2.22219		
POLDIP2	1.926464		-4.14988		
RABL6	-2.22525				
HMOX2	-3.79263	-2.68123	-2.03253	4.239977	2.036748
PGM1	-2.236				
LRRC59				-2.24738	
CNIH4		-2.24852			
CRLF3				-2.25463	
NFKBIL1	-2.25674				
DLG1					-2.25945
ATP6V1D				-2.26013	
GPT2		-2.26076			
IRAK1			-2.2662		
UBR4				-2.26954	
PREB			-2.27082		
VPS26A			-2.28432		
C12orf73					-2.29409
CALD1				-2.29589	
ALDH5A1		-2.32574			
KRTCAP2					-2.3308
SYNJ2BP	-2.33112				
NSDHL					-2.33696
FAM114A2					-2.33745
DCBLD2			-2.33989		
HDLBP	2.182006		-4.52934		
TBL2				-2.34997	
NDUFAF2	-2.35618				
NOP16			-2.35907		
RAB14	-2.35919				
LAMTOR3				-2.37282	
SUPT6H				-2.37395	
ANAPC10				-2.37431	
KIF21B					-2.37699
BPNT1			-2.38095		
MANBAL					-2.38513
MYL12B				-2.38689	
AP3M1				-2.39502	

TST					-2.40344
MRRF			-2.41219		
PSMB5			-2.41301		
MT-CO2	-2.41993				
GNL1	-2.42219				
HLA-A					-2.42323
LAMP2		-2.42551			
C7orf55				-2.42772	
CD276			-2.43508		
RFC3					-2.43529
RBMXL2				-2.44121	
VPS33A				-2.44632	
PDIP46				-2.44674	
BUD31					-2.44734
HADH				-2.45223	
NCAPD3			-2.45663		
LPCAT1			-2.47489		
ACTB					-2.48538
PGD					-2.48798
GPX1				-2.49268	
DKFZp586I031	-1.73296	-4.60639		3.84153	
ZC3HAV1				-2.502	
RRP1B				-2.5031	
HNRNPUL2-BSCL2	2.337714	-1.13266		-2.33118	-1.37867
NIPBL			-2.50935		
CYFIP1			-2.51274		
SLC33A1		-2.51406			
DHCR24				-2.51668	
Nbla03646				-2.51668	
KTN1					-2.52017
ARPC4-TTLL3		-2.52035			
TIMM10B		-2.52139			
TMOD3			-2.527		
PC		-2.52993			
CKMT2					-2.52996
RBM23					-2.53505
UTP6				-2.53598	
PCBP3					-2.53672
NEDD8-MDP1		-2.54069			
GATAD2A	-2.54238				
UBAP2L				-2.54363	
TARS					-2.54737
B2M					-2.5549
SYNJ2BP-COX16		-2.55547			
NUDCD1	-2.55761				
SFN			-2.56022		

GLUD1					-2.56539
KPNA1			-2.5723		
SSR1		-2.57662			
DKFZp781M17165				-2.57858	
PPP2R4				-2.57858	
OSBPL8			-2.58392		
MCM7				-2.58545	
GDI1					-2.59405
CSNK1A1L				-2.59422	
CD81	-2.13584	-2.71173			2.252493
HNRNPLL			-2.59659		
HNRPLL			-2.59659		
URB1		-2.59711			
GAR1	-2.59725				
ATP5SL	-1.35189				-1.24605
LRRFIP2			-2.59839		
PSMD10					-2.59897
TBC1D5			-2.62504		
G3BP1		-2.62567			
WBP11					-2.62712
CPOX			-4.03028	1.400861	
ELAVL1		-2.62984			
RAP2C	-2.07935	-4.01827		3.458712	
RAP2A	-2.07935	-4.01827		3.458712	
RAP2B	-2.07935	-4.01827		3.458712	
FAM162A		3.274371		-5.92341	
GNA11			-2.68151		
GNA14			-2.68151		
GNAQ			-2.68151		
EED		-2.68349			
MTX2	-2.68509				
LUC7L3		-2.68672			
C1orf131		-2.68681			
SLC39A9					-2.69056
MRPL46			2.069481		-4.76571
UQCRB					-2.69701
CKAP5	2.486409	3.313976		-3.74204	-4.76417
C17orf85			-2.70602		
SLC25A12	-1.17503		-1.53207		
DKFZp586F0420					-2.7083
ANKFY1					-2.7113
RPL5	-2.71899				
PLOD1					-2.72175
PPIAL4A	-2.72575				
USF1			-2.73449		
B4GAT1					-2.74096

BSG	-2.74244				
COG4					-2.74649
NDUFB9	2.025071	-4.7867			
ZC3H18			-2.76245		
C19orf43			-5.83286	3.070379	
MOB1A	-2.77684				
MOB1B	-2.77684				
HACL1			-2.78187		
ACSL4					-2.79607
MYH9				-2.79751	
CCT5				-2.79829	
ZC3H4					-2.79999
ARID1A		-5.56581		2.76516	
PYGM				-2.81144	
DSTN					-2.81499
A2M		-2.82526			
PVR			-2.83681		
MCM2	-4.4692			1.627222	
ATAD3C			-2.86274		
SPEN					-2.86463
ZC3H11A	-2.87425				
DDHD2					-2.87826
WTAP					-2.87976
KIF21A					-2.88117
HIST3H3				-2.88293	
UBR2				-2.88687	
EMD	-2.89008				
MSH3		-2.89048			
ITM2B					-2.90677
GTPBP4				-2.91561	
SH3BGRL3			-2.91687		
NIPSNAP1			-1.11274		-1.81062
CD47	-2.92683				
HK2			-2.92789		
C8orf33	-2.9483				
RNASET2				-2.95704	
UPF1	-2.95958				
NUP88				-2.96026	
CTR9			-2.97888		
SPIN1					-2.99229
ZNF638	-1.90258	-1.10911			
NUMA1	2.356666	3.318138	-3.38665	2.259956	-7.56077
MRPS31		-1.1554		-1.85732	
XPC		-3.01683			
AMOT				-3.02075	
CRYZ			-3.02327		

MCTS1	-3.03121				
GEMIN6					-3.03303
ERAL1	-1.66199				-1.37161
SARNP				-3.03775	
CHTOP			-1.83287		-1.20788
METTL2A		-3.04348			
METTL2B		-3.04348			
C21orf59		-3.04659			
MAPK3		-3.04997			
NABP2		-3.064			
CROCC		-3.06424			
GADD45GIP1			-1.74697		-1.31996
COPE				-3.07316	
EPRS					-3.07368
RANGAP1		-3.07439			
EIF1AX				-3.07791	
ABCE1				-3.08331	
NT5C3B					-3.08342
UPF3B					-3.09273
KRT5				-3.09786	
HSP90AA5P		-3.10333			
SRSF7		-3.10382			
DHX15		-3.10422			
MAP2K4			-3.11497		
RARS			-3.11519		
KRT5		-3.11605			
HSPBP1					-3.11635
S100A13				-3.11925	
MYH1					-3.12378
MYH13					-3.12378
MYH2					-3.12378
CEP170			-3.12517		
SEC61B		-3.12897			
YIPF5					-3.13226
LSS		-5.32797			2.191629
MRPL49			-1.60293	-1.53817	
U2AF2					-3.14343
EIF6	-3.14464				
CLASP2					-3.15945
HSP90B2P					-3.16336
NUP188		-3.16376			
AATF					-3.1651
RAB8A					-3.16979
PREP		-3.17079			
PPP2R1A	-1.72012		1.30445		-2.76007
BLMH			-1.73566		-1.44336

MAP1LC3B					-3.18523
MAP1LC3B2					-3.18523
GNB2		-3.19418			
SNX18				-3.19827	
GPD1L				-3.20154	
SYNGR2		-3.20197			
SREK1					-3.20439
RSU1					-3.21191
SLC25A10			-1.8962		-1.32037
GNPDA1		-3.22023			
CD3EAP	-3.22076				
STRIP1		-3.24008			
WBP2					-3.24064
RELA					-3.24188
YWHAE				-3.2432	
VAMP2		-3.24358			
VAMP3		-3.24358			
GNB2L1		-1.2349		-2.01328	
SEC22B			-3.27461		
MRPS6	-1.58098	-1.69447			
MCM4					-3.27649
KIAA0368			-5.9256	2.645276	
TIMM22			-1.01472	-2.26711	
MRPS17				-2.03559	-1.24748
TMEM165		-3.28377			
ALDH16A1					-3.28612
CLN6		-4.09617		4.162955	-3.35753
RAP1B		-3.29739			
QPCTL				-3.30868	
RPS5					-3.3094
ACTN2				-3.32098	
SLIRP				-3.32717	
RAD21					-3.33092
ITGB1				-3.33129	
MLEC			-1.03495	-2.31137	
NDUFA13					-3.34779
GOT1	-3.35792				
ACO2					-3.35981
HSP90B1					-3.36368
UTP14A					-3.368
GMPR2					-3.36912
AK2		-3.37229			
ASNSD1					-3.37668
UBE2S		-3.38723			
CWF19L1				-3.39796	
S100A11				-3.40122	

LRRFIP1				-3.41549	
COA4					-3.42305
OXA1L		-3.42674			
CAST					-3.43104
CCAR1	-1.81673	-1.61559			
MRPL44				-3.43332	
SNX6					-3.43502
AKR1E2		-3.44747			
LSM6		-3.45214			
MRPS26				-3.45324	
DDX24				-3.45925	
MYL6B		-3.46164			
POLR1B	1.125633	-4.594			
HNRNPA3		-3.46885			
NDUFA7		-3.4703			
KRT16				-3.47708	
C9orf78		-3.05708		-2.35182	1.923778
ACYP1					-3.48797
TBRG4					-3.48968
WARS2					-3.50336
CDK5RAP3					-3.50359
ZPR1					-3.50776
ZNF24					-3.5127
GSPT1		-3.53017			
KRT17					-3.53999
YTHDC2				-3.54384	
ZBTB33					-3.54901
CSTF2T			-3.555		
TRIM25		-3.55862			
SUMO4		-3.56917			
PRPS1L1	-3.57737				
EIF1AD		-3.58561			
RPS11	2.593197	-4.09635		1.507423	-3.59124
RBBP6			-3.59617		
PRMT3		-3.61031			
ZNF326	-1.97816	-1.64671			
SCAMP3			-3.62602		
RPN1			-3.62885		
AMPD2	1.779243		-5.41338		
DIS3		-1.90975		-1.72691	
HOXA13					-3.64491
HOXC13					-3.64491
ITPR3		-3.6474			
EXOSC5			-1.32081	-2.33523	
TXLNG				-3.66183	
ABCB7		-3.67124			

KCTD15		-3.6785			
MLLT4	-1.41889	-1.13276			-1.13229
NRD1		-3.68804			
MRPL38		-3.69609			
ARCN1		-3.69972			
POLR2E	-2.66165			2.457537	-3.49717
DNM2	-3.70972				
HECTD3					-3.72409
UBQLN4		-3.72751			
AK6	-1.82588		-1.90312		
TAF9	-1.82588		-1.90312		
TAF9B	-1.82588		-1.90312		
FAM208A		-3.74553			
SGTA			-3.75919		
EMC6		-3.76712			
TCEA2		-3.77435			
MAD2L1				-3.78417	
RPS6KA3					-3.79231
ATP5E		-3.79663			
ATP5EP2		-3.79663			
SRRM2	1.516944	-3.3061	-2.31777	-1.85505	2.162365
SMCHD1		-3.80584			
POLR3E	-1.74436		-2.06153		
TRIP12			-2.17492	-1.6313	
PGAM2		-3.80782			
NSMCE2					-3.80838
EIF2AK2					-3.80849
DHFR					-3.80918
ASPSCR1				-3.80926	
PRPF4				-3.8113	
KIAA1143		-3.81667			
INA	-1.65838			-2.16121	
PSMA6		1.333539	-2.72663	-2.43483	
NME4				-3.85053	
RPTOR				-3.85209	
DDX6	-2.09014		-1.7732		
IKBIP	-3.87843				
FASN					-3.89296
KPNA5		-3.89825			
RANBP2		-3.90072			
NEMF		-3.90486			
ELAVL2		-3.90543			
ELAVL4		-3.90543			
AARSD1		-3.91463			
RBM10					-3.92664
FAF2	-2.50296		-1.42749		

PARN				-3.93113	
ADCK3					-3.94006
SYNCRIP	-2.19762	-1.743			
CNDP2			-3.95345		
PPT1					-3.96962
DNAJC10	2.077288	-4.30739	2.212304	-3.96035	
GMPPB		-4.00627			
PPM1A		-4.01172			
PPM1B		-4.01172			
PPID	1.547211		-5.55908		
NCAPG		-4.03302			
APOOL		-4.0333			
TOMM70A	-4.03567				
MEAF6			-4.03853		
EXOC4				-4.05024	
CLUH				-4.05892	
TUFM		-4.06347			
ATP1A4					-4.06888
TAF6			-2.12199	-1.94796	
CIRBP	-1.68602		-2.3889		
CDC20	-4.07679				
ISYNA1		-4.08311			
NCAPH2	-1.83997		-2.2595		
DDX50	-4.15282				
KRR1		-4.15838			
CNN3	2.500506	-3.57681	2.009336	-2.25604	-2.83736
PSMB6		-4.16094			
SCAMP1	-1.95537		-2.21884		
PPA2	-1.49032	-1.38529	-3.93064	3.95158	-1.32398
SLC25A14				-4.18671	
PRKAR1A			-4.19298		
SCAF8				-4.19766	
ACAA1	-1.47217	-1.15649	-1.57531		
TSFM			-4.20755		
RIOK1		-4.20914			
RPS7		-4.20932			
KIF2A				-4.21976	
CHMP4B		-4.23058			
GLTSCR2		-4.2403			
KRAS		-4.24032			
CCDC43		-4.24178			
PDCL				-4.2444	
SUCLG2					-4.24647
RBBP5	-1.63827		-2.61193		
FBXO22			-2.19836	-2.0615	
DUSP9	-4.26215				

CYB5A		-2.33338	-1.9424		
ALDH18A1	-2.34246		-1.93961		
RABGGTB	-1.74383				-2.54144
MMAB	-1.28051		-3.01343		
BOP1		-1.33159	-2.96629		
RPL13		-4.30034			
DYNC1H1			-2.68134	-1.62028	
EXOSC9			-2.83629		-1.47545
NDUFA8		-2.33454		-1.9783	
OXSM	-2.39439			-1.95754	
VPS53			-4.35441		
ALG5				-4.35789	
UQCR10	-2.00223	-2.35803			
SLC25A11	-1.86443	-1.10765			-1.39476
UHRF2		-4.36837			
UQCRFS1		-2.07789	-2.29524		
CTBP1				-2.21661	-2.15835
CHRAC1	-1.27144	-1.74736	-1.36014		
MEN1		-4.38629			
SBNO1	-4.38726				
P4HA1			-1.9399	-2.46303	
MRPL55					-4.40429
MGME1	-4.4175				
RAF1				-2.20154	-2.22375
ECH1	-2.39458			-2.03307	
CMTR1	-1.70667		-2.72667		
WDR18		-4.44914			
URB2	-4.4795				
STMN1	-1.9903		-2.4957		
COPB2			-1.98277	-2.50439	
MRPL34		-4.50409			
CHCHD1	-3.36453		-1.14543		
OSGEP		-2.38376		-2.12675	
LSM7		-3.32069	-1.19163		
HOXA5		-4.53348			
AAMP				-1.84889	-2.69083
JUP			-1.78991		-2.75553
ENAH	-4.71096		2.181123	2.764129	-4.7803
CLASP1			-4.54792		
XPNPEP1					-4.55798
ALCAM				-4.55926	
DVL2	-2.15304		-2.4129		
RPS29					-4.57615
DMAP1		-4.58421			
GCN1L1	-2.33777		-2.24706		
ABCD3		-2.26008	-2.32592		

GALM			-4.58792		
GAPDH			-2.02558	-2.56521	
THUMPD3		-4.59677			
YWHAB				-4.5992	
POLR2F	1.17413			-2.9935	-2.78556
PPIG	-4.60977				
DEGS1					-4.61022
RAP1GDS1		-3.14529	-1.46996		
ZMYND8		-4.6335			
DCAF7		-2.92246		-1.71666	
KRT76				-4.64438	
RABAC1		-4.64624			
ARPC3		-4.65029			
CHD1		-4.65099			
DCAF16				-4.65581	
POLR2G				-2.07974	-2.5859
DDX18	-1.51537	-1.37748		-1.79523	
KPNA3		-4.6915			
KPNA4		-4.6915			
USP14			2.570397	-1.6973	-5.56857
KIF5A		-4.6985			
KIF5C		-4.6985			
TUBB4B			-2.56464	-2.13838	
CTH		-4.71204			
KRT26				-4.71562	
NDUFS7	-4.72753				
CSNK1A1				-2.59422	-2.13942
MYO1D		-4.7727			
TEX10		-4.7863			
ALB	-2.73003		-2.05899		
RABIF				-2.46588	-2.33451
YBX3	-2.74366			-2.06569	
TOR1AIP1					-4.8235
HEBP1			-4.84271		
RBM19		-4.85682			
FAHD2A	-2.2669			-2.59539	
CDK9	-2.35325		-2.50953		
HOOK1	-4.8726				
EXOSC10	-4.87928				
GK			-2.56566	-2.31966	
GK3P			-2.56566	-2.31966	
KAT7			-2.65347	-2.23367	
PROSC				-4.90439	
FAM3C				-2.18272	-2.73686
MYEF2					-4.92664
SUMO1				-2.3563	-2.57072

DIAPH1	-4.92733				
SYNGR1	1.763476	-5.02074	2.398224	-4.07627	
KLC1		-4.93843			
DUS1L			-4.94347		
WBP11				-2.37605	-2.56994
GGCT				-4.96207	
RBM14	-2.04076	-2.92151			
NSA2			-4.9708		
EIF2B3		-4.97185			
RTN1	-3.29104			-1.70514	
RPL35A	-1.71821	-2.52438		1.83632	-2.59259
ARFGAP1				-2.38236	-2.61909
ZNF768		-2.43029		-2.57303	
MTPAP	-5.01812				
ABCF1				-2.50547	-2.5132
DNAJC7			-1.8517	-3.18239	
SLC25A4				-2.4517	-2.59165
OCLN		-5.05016			
TBL1X			-5.05216		
TBL1XR1			-5.05216		
TBL1Y			-5.05216		
MCAT			-5.05467		
RABL3				-5.05571	
NFXL1			-5.05917		
MTMR1				-2.72771	-2.3331
PPIL1				-2.60748	-2.4619
MAPK8IP3			-5.07467		
PDS5A	-5.08281				
MRPS28					-5.08914
SAAL1	-2.53843		-2.56563		
DVL3			-5.13101		
NUCB1	-3.13268			-2.01113	
HIST2H2AC		-5.15158			
ACTR3B	-5.15805				
RAC2	-5.16093				
MMTAG2	-2.63378	-2.57421			
AP2M1	-3.19158		-2.01803		
CORO1C			-3.67221	-1.56106	
HDDC2			-5.23952		
KRT13				-5.25169	
PAPOLA			-5.26077		
ENY2				-2.49698	-2.76663
36951		-5.27676			
37316		-5.27676			
CSNK2B-LY6G5B-562		-2.74885			-2.53074
ABCC4		-2.77553		-2.51139	

CDSN			-5.28789		
RPL19			-2.17058	-3.13779	
GRK6			-2.68347		-2.63429
RRS1			-2.87846	-2.44128	
CADM1	-1.7068		-3.61404		
DBT	-2.00068	-3.32128			
BMS1	-2.85731		-2.47589		
ATP6V1H			-5.33967		
ANXA5		-2.68438		-2.65887	
NUP155	-2.49249	-2.87403			
PSMB3			-2.98889	-2.38387	
MORF4L2		-2.55699	-2.89121	2.684399	-2.61222
ACOT1		-5.37735			
ACOT2		-5.37735			
DDX52		-2.32631			-3.06498
UROD				-2.27214	-3.12246
CPD		-2.70313	-2.70265		
GPN1		-2.66698	-2.74539		
DKFZp566H1924				-5.41626	
NPTN				-5.41626	
TAGAP				-5.42428	
RAB11A			-5.42546		
MAP2K3					-5.43039
MAP2K6					-5.43039
IQGAP3	2.151935		-4.20602	1.822634	-5.21442
ARHGDIA					-5.44885
PYGB		-2.63965		-2.81144	
ETFB		-2.48834	-2.96427		
C1orf158			-5.48694		
DIDO1			-2.9561	-2.54441	
BCL2L13		-3.32675		-2.17674	
RPF2		-3.24805		-2.26113	
CPSF1	-2.59122			-2.92307	
CRELD2		-5.51571			
FLII	3.42069	-4.33417			-4.6052
ATAD3A	-1.60541	-1.15037	-1.52031		-1.24343
ATP6V0C	-2.73912	-2.78425			
KIAA0101			-5.53152		
BCAT2			-1.80275	-1.56661	-2.16978
SCML2		-2.89512			-2.65573
LARP4B			-5.5754		
ACLY	-2.77196		-2.80471		
SERF2			-5.57885		
GPS1	-5.58318				
PURA			-5.59138		
FKBP4		-3.53169	-2.06061		

SLC39A7		-2.48208	-3.18378		
TNKS1BP1	-5.6726				
KHDRBS3	-2.3595	-3.31358			
DUSP3	-2.64822				-3.02996
ADI1	-2.88167	-2.80455			
EFTUD2	-1.91489			-1.63057	-2.1464
PLIN3		-1.11877	-1.985	-2.60175	
TUBA1B	2.256509	-3.84488	2.315176	-3.32749	-3.12603
ATXN10	-1.78596		-1.99029		-1.9654
SCO2	-3.28888		-2.47276		
FN3KRP				-5.77284	
TUBA1C		-3.08797		-2.68811	
PLCG1		-7.96511			2.175216
RUVBL1			-2.66818	-1.8059	-1.33291
NLE1			-5.80896		
NFU1		-5.81277			
ATAD5			-5.81575		
SLC19A1		-2.69573	-3.12034		
CASC5	-5.8261				
ARF1			-2.69992	-3.14567	
EHMT1			-5.84813		
NCOA5		-3.44026			-2.40799
SLC16A1		-5.86328			
EPHA2			-2.75417	-3.11998	
KLC2			-2.66411		-3.23637
RPL26	-2.68106		-3.22418		
BRD8		-2.63529	-3.27649		
HSPD1		-2.79805		-3.12388	
BAG2			-2.93854	-3.00946	
LARS	-2.4397		-3.51071		
DDX47		-2.62691			-3.32607
RUVBL2	-1.96323				-3.99436
C2orf47	-5.96365				
TP53		-3.89935		-2.07043	
ARF6			-2.56111	-3.45671	
CHAF1A		-3.21161		-2.82699	
HMGN3				-2.61699	-3.42233
KDM2A				-6.05091	
PGM2			-3.7279		-2.39205
NPLOC4	2.237215	-4.82177		-6.45841	2.913432
CTNNA1			-1.98626		-4.15357
YBX1				-4.12224	-2.03098
TMED2					-6.16416
SSU72		-2.75194	-3.4141		
CPT1A	-2.18924		-6.06365		2.078497
SRPR	-4.18327		-1.99172		

IGF2BP3	-1.62726	-4.55308			
MANF	-3.84208		-2.3511		
TMEM33			-6.21424		
RPS28		-3.1099		-3.11829	
TMEM230				-3.00098	-3.23006
HOXB9		-3.35205	-4.52173	1.62912	
HSP90B1		-4.18381		-2.06155	
NOP2			-1.54071	-1.62757	-3.07817
DLD		-6.25146			
WBP2			-1.90798	-2.27402	-2.07031
TBL3	-1.54275			-2.70846	-2.00497
GBF1			-6.2616		
TIMM50			-1.08509		-5.17657
FOXK1		-6.27134			
PRPH	-2.25491		-2.13499	-1.88298	
HNRNPD		-3.11219			-3.17615
TIAL1		-3.84441			-2.45179
H2AFY		-3.15706			-3.14491
ACTN4	-1.16988	-2.51537		-2.62115	
DDX3X			-2.91939		-3.43704
PNP			-2.10191	-2.05808	-2.19873
MYDGF	-2.06206	-4.29725			
MRPL48	-2.13757		-1.9629		-2.27709
NUPL1			-2.85985	-3.51822	
STMN3			-6.38517		
HMGCS1		-1.30097		-1.8795	-3.21101
VIM	-1.98481		-2.30395	-2.10661	
CTDSPL2	-1.75362		-4.64741		
TMEM261		-4.09466	-2.30723		
COX7A2L	-2.06724	-2.24255			-2.1101
NOMO2	-1.6223		-1.5839	-3.21712	
SMC6		-2.81204	-3.61219		
RPS2			-1.90566	-2.55842	-1.96352
TRMU				-4.04807	-2.38451
TXN2		-2.421	-1.84366	-2.16848	
PSMC5	-2.45609	-3.97963			
CORO7	-4.30313		-2.1347		
TACC3		-2.85518		-3.59112	
IPO5				-3.14492	-3.30814
HCCS	-4.24395		-2.21337		
FAM21A				-3.17259	-3.29602
FAM21B				-3.17259	-3.29602
FAM21C				-3.17259	-3.29602
PRPF8		-4.69843	4.342136	-3.06528	-3.05385
PTRH2			-3.38068		-3.10879
COX7A2	-2.07222	-2.21869			-2.20402

SRSF11	-2.75083	-3.76632			
RFC5		-5.41493	-1.10643		
VPS25	-1.66645		-2.10018		-2.76463
DIEXF		-1.37618		-2.99869	-2.15878
WDR82	-2.16014	-4.37898			
TOP1MT		-3.36225		-3.1833	
TUBB2B				-2.53882	-4.01458
C7orf55-LUC7L2	-2.37358			-2.05823	-2.144
UBQLN1		-3.72751		-1.72742	-1.12263
SRSF2		-3.10301			-3.48141
SBNO2	-4.16216		-2.42691		
NIT1			-1.2565	-5.35202	
MTCH2		-1.34063	-1.57513	-2.35446	-1.38127
SRRT		-2.5251	-1.86454	-2.26863	
MRPL19		-6.6586			
NOL7			-2.00735	-4.66555	
CMAS	-3.20468		-5.51455	2.044738	
PRMT5	-3.6271	-3.087			
LIN7B		-3.58532			-3.13237
CSTF1				-3.15913	-3.56433
CSRP2	-3.19154		-3.53224		
NAT10	-2.25839		-1.93023	-2.54465	
ESYT1	6.467632	-4.79004		-4.37247	-4.05334
RBM27				-2.6355	-4.12646
ACOT9				-6.76808	
LTF			-3.71124		-3.06105
UBE2G2		-3.98902		-2.78618	
SPAG9				-3.06006	-3.71783
FKBP5	-3.09271		-3.69492		
CHD1L	-3.81441			-2.99114	
AGPAT9				-2.41722	-4.38844
NELFE				-3.10898	-3.69972
MRPS25	-1.63374	-2.49322	-1.56416		-1.12077
KAT7	-2.42405			-4.39631	
SDE2	-2.17183		-4.65022		
MKLN1	-4.30843		-2.53646		
CLTB		-3.12088			-3.73507
MRPS27	-6.8674				
EXOC8				-3.3176	-3.55322
LTV1				-3.31905	-3.55864
PDS5A		-4.70084		-2.19316	
EEF1B2	-4.87672		6.901283	-3.02157	-5.90591
GHITM		-2.25257		-2.38453	-2.2668
UTP11L	-2.48239	-4.42287			
H3F3A	-2.12328	-2.23795	2.158461	-4.71127	
KRT16		-6.92303			

EIF5B	-3.45535	1.418818		-2.43152	-2.47053
DACH2		-3.91386			-3.03316
BCAP31	-2.26236		-2.98617		-1.7062
ASH1L			-2.69874	-4.29071	
DCI	-3.33854				-3.67535
GRN	-1.93226		-2.36509	-2.72064	
TACO1		-2.93617		-4.08373	
ZKSCAN4	-2.231		-2.3353	-2.45775	
UTP20	-2.74654		-2.08717		-2.19909
TROVE2	1.795647	-4.14842			-4.68103
YLPM1	-2.45473	-5.40533		2.529958	-1.71539
XPO1		-3.18305		-3.86861	
AP2B1	2.388375		-2.1287	-3.24324	-4.07281
CMC2	-2.21818	-2.38715			-2.47959
MAP7D3		-2.45673		-2.35687	-2.27601
VTA1	-2.055	-2.33322			-2.72539
CALCOCO2			-4.75649		-2.37972
WDR36		-2.84368		-4.30302	
SRSF8		-3.61854			-3.55126
SUMF2		-3.27645	-2.25588		-1.65465
DACH1		-4.01833			-3.18506
PWP1		-2.4507		-2.51245	-2.24038
SUCLA2		-4.17274			-3.03472
THOC1	-2.11149	-2.8038			-2.34316
NDUFV1	-2.99511		-2.43244	-1.84207	
AGPS	2.032368	-2.50917		-4.24536	-2.55315
LIG1	-2.59695		-2.63314		-2.05609
GRSF1	-4.2567		-3.05144		
PRRC2A	-2.74735	-2.85792	-3.3375	1.633617	
CLNS1A				-3.11551	-4.20326
SNRPB	-3.8186				-3.5004
SNRPN	-3.8186				-3.5004
UQCRH	-1.45192	-1.17514		-4.70018	
DNTTIP2		-2.92574	-3.17887		-1.24395
PPARGC1A	2.486487				-9.84385
LCP1	-4.06945	-3.29955			
FOXC2		-2.31858		-2.20095	-2.85316
FOXG1		-2.31858		-2.20095	-2.85316
FOXL1		-2.31858		-2.20095	-2.85316
BAG3		-4.13331			-3.24052
RTN4		-3.42722			-3.94734
KIF4B	-1.99927			-2.58796	-2.82035
DRG1				-5.52718	-1.88224
EIF3F	-1.62669	-1.40272	-2.57647	-1.80361	
TTBK1	-2.24854		-2.73219		-2.4291
FKBP10			-3.55708	-2.18462	-1.69113

AIP				-3.45806	-3.97713
GLRX5	-2.24875		-3.2112		-1.9961
WDR44				-3.24214	-4.26653
ATP6AP2	-2.33239	-2.67079			-2.53825
MT-ATP6				-2.29688	-5.24547
WDR41		-4.086		-3.47256	
SMARCA4	-1.60746		-2.73311	-3.22152	
SNX5	-2.37434		-2.64313	-2.55127	
ZC3H15			-5.55858		-2.01177
C4orf27			-4.1438		-3.43672
ATP5O			-1.95751	-2.62852	-2.99906
MRPL1		-4.31086			-3.28206
ITPA				-3.45766	-4.16155
ABHD14B				-3.10897	-4.52472
CAPN2	-2.45855		-2.77963	-2.39984	
CELF1		-2.73911		-2.27341	-2.6276
UBE2O	-2.0075		-5.63428		
RPS26P11		-4.21254			-3.43205
ECI1	-3.65732				-3.99413
NDUFV2	-1.26997	-1.36095		-2.57023	-2.45188
PRPF19		-3.04747	-2.13839	-2.46801	
WDR33		-4.14479	-3.50948		
RPS26		-4.21254			-3.44696
RPL36A	-5.04617	-4.42927	-4.07364	1.932089	3.95545
SLC25A3	-1.82749	-1.57369	-4.26902		
ANKLE2		-4.54385		-3.13059	
SSRP1		-7.67899			
STUB1	-3.92477	-3.73239		3.419912	-3.46567
UFD1L			-4.36121		-3.35071
DKFZp434D199	-2.7179		-5.00503		
MDH2		-4.8577			-2.87118
KRI1				-4.63576	-3.09851
COX4I1	-2.17487	-2.96579	-2.59582		
IMP3		-3.68472			-4.09142
PAF1		-4.00318			-3.78314
RAVER1	5.249719	-2.94495	-4.79086		-5.30869
PSMC6		-4.99497		-2.8007	
MYBBP1A	-2.88105		-3.26175		-1.65342
TMEM205		-2.86064		-2.63632	-2.30021
PPAN-P2RY11				-4.7386	-3.0722
PNO1		-3.02556		-4.78687	
PML		-2.82674		-2.45596	-2.53436
SLC1A5	-1.64313		-3.6626		-2.52232
DES	-2.06782		-1.24522	-2.38222	-2.13424
TARS2	-2.33506		-2.47315		-3.02639
COMMD6	-2.03996		-5.81204		

DYNC1I2	-4.2873			-3.56886	
COIL		-4.41916	-2.05517	-3.48631	2.100155
AP3B2		-4.5168			-3.34889
ARFGAP3		-2.69574		-2.76914	-2.41465
SMARCD1	-1.61602		-1.92634	-4.34978	
SPCS2		-4.48593		-3.41001	
KPNB1					-7.89599
ILF3		-1.70511		-4.70543	-1.49148
FTO		-2.94594		-2.62215	-2.34294
SPCS1	-2.00694				-5.90688
SMPD4		-4.50008			-3.4164
UBAP2	-5.29387		-4.57933	1.938873	
PHF5A		-3.03547			-4.90469
CREBBP			-5.27437		-2.66661
EP300			-5.27437		-2.66661
GTPBP6				-3.39885	-4.54457
RAB33B		-2.92535		-2.85588	-2.16395
RAB39A		-2.92535		-2.85588	-2.16395
RAB39B		-2.92535		-2.85588	-2.16395
RAB3A		-2.92535		-2.85588	-2.16395
RAB3C		-2.92535		-2.85588	-2.16395
TRIP4		-2.74188		-2.72748	-2.49144
TCEB2		-3.51285			-4.46867
OSBP2	-5.40918				-2.57241
PTRHD1	-2.33547		-1.9096	-1.83947	-1.90301
ARL8B		-4.10319		-3.89402	
NCAPD2		-4.82017			-3.21349
TIMM23			-1.28665	-6.76123	
TIMM23B			-1.28665	-6.76123	
SLC25A31	-2.19658		-2.91153		-2.93997
SLAIN1				-3.95419	-4.09425
PTTG1IP				-3.8285	-4.23669
CTAGE15				-3.58782	-4.47776
CTAGE5				-3.58782	-4.47776
CTAGE6				-3.58782	-4.47776
GMFB	-5.18809			-2.90303	
NSUN5P1		-2.62258		-2.66368	-2.81084
NSUN5P2		-2.62258		-2.66368	-2.81084
DKC1	-2.46678	-2.63244	-3.00273		
GPR180		-4.13587			-3.96937
YWHAZ				-4.42053	-3.69965
ATP5H			-4.38387	-3.7431	
GALNT2		-4.19211			-3.93557
MAZ	-2.14309		-1.07808	-3.19739	-1.71643
ZNF629		-2.80244		-2.8021	-2.55906
POLR3C			-4.69821	-3.46868	

LIG3		-2.98997		-2.61098	-2.57777
HELLS		-3.91615	-4.28862		
DNAJB12	-3.35783		-4.88741		
APMAP	-2.33044		-3.81224	-2.10491	
WDR43		-3.42906	-1.36443		-3.47083
SPTBN1		-4.44553			-3.82598
SYMPK	-6.37155				-1.90478
PBK	-2.44687			-2.77982	-3.05143
CDK12			-2.66903	-3.15126	-2.47049
PRPF3	-1.64692	-3.75091	-5.24415	2.341057	
RECQL		-4.32585			-3.98184
BLM		-4.32585			-3.98184
RABGGTB				-3.53706	-4.7778
SRP9				-4.74538	-3.58857
NDUFC2		-4.16122			-4.18403
CSNK2A2		-3.70084	-1.61239		-3.04023
MPHOSPH10		-8.36237			
NPM3		-2.73259	-2.13896	-3.50628	
RPS19BP1		-4.11694		-4.26478	
VHL			-4.81411		-3.66072
PANK4		-3.28267	-2.30295	-2.89746	
COX6C	-2.81842		-1.71594		-3.96451
BCKDHA		-5.63313			-2.87448
BAZ1A		-3.60975		-2.43816	-2.47076
NUP62		-3.2391		-2.36404	-2.92661
CTR9		-2.52744	-3.58104		-2.44747
GUK1		-4.49596			-4.06162
CTU1			-4.31381	-4.26613	
TRA2B		-4.77632	-3.81167		
40787	-4.17009	-4.43311			
COX6A1	-3.98046	-4.65076			
UBXN4		-2.81945	-3.33139		-2.49551
AGO1				-8.65215	
AGO2				-8.65215	
SRSF7			-1.13101	-3.43668	-4.08921
ARHGAP17				-4.34453	-4.31711
PSMD13			-4.94403	-3.72753	
DHX40		-2.69857		-6.00532	
U2AF1	-4.24577	-4.47581			
HSD17B10	-3.29486		-2.03831		-3.39934
HSP90B2P	-4.07171	-4.66243			
CCNB1		-8.77883			
MAP2K2		-4.05906			-4.71988
TSSC1				-4.17522	-4.60569
SF3B1		-4.23631			-4.57216
SETDB1		-4.0699	1.727491	-3.3368	-3.14468

38961		-3.35582		-2.91379	-2.56173
THYN1			-2.58107	-3.2886	-2.96259
H3F3B	-2.12328	-2.23795	2.158461	-4.71127	-1.92272
SCPEP1		-3.89582	-2.0222	-2.919	
CBLL1		-2.73404		-3.92555	-2.17972
CYC1	-2.15197	-2.23469		-1.92864	-2.52988
PPP6R3	-4.66827		-4.19324		
TMUB1		-5.05682		-3.82673	
MRPS14			-2.12948	-3.51832	-3.25495
AFF1		-5.42578			-3.48461
SFXN4	-4.25723		-4.65462		
MTCH1			-2.18375	-3.48679	-3.27149
CDK9		-4.87376		-4.0882	
ANAPC5	-4.38424	-4.5823			
PZP		-3.01686		-3.52433	-2.44594
XRCC1	-2.11136	-3.29197	-1.1455	-2.49524	
GTF3C2		-4.60952		-4.45098	
TFIP11		-3.01145		-3.41337	-2.65848
ECI2				-5.24312	-3.87416
OGT		-4.92156	-4.24194		
TES				-3.89731	-5.2811
NEFH	-2.87052	-4.17303			-2.16106
NEFL	-2.87052	-4.17303			-2.16106
TRIAP1		-4.18304		-5.03002	
ATP5B	-2.63928		-4.45187		-2.13315
PYCR2			-1.55106	-4.66974	-3.00706
HBA1	-2.50671	-2.53685		-1.56821	-2.62687
SEC23A		-2.89402	-3.63712		-2.73867
ADO	-2.57567		-2.82383	-3.90859	
CAMK2D	-5.1999	-4.11135			
ECHS1		-2.89894		-3.86367	-2.56153
VDAC2	-2.30841	-2.82183	-1.81102		-2.38939
NDUFA9				-5.76442	-3.57634
SRPRB		-4.15589	-1.68789		-3.50606
WDR3		-3.82924		-3.03418	-2.49719
VAPB		-3.53373		-3.29584	-2.54122
ZC3H7A		-4.89656	-4.49299		
ZC3H7B		-4.89656	-4.49299		
VDAC1	-2.53362	-2.92457	-1.78329		-2.15886
SOAT1		-3.49965	-3.48529		-2.46681
KLF12		-4.98045			-4.49282
AP2A2	-2.97922	-3.48256		-3.02029	
GPATCH11					-9.49448
CHP1		-5.28051	2.296386	-3.95732	-2.56045
RBM39		-4.49496	-3.17059	-1.83783	
TNPO1	-3.24849	-3.65085		-2.61968	

RAC2		-5.57203		-3.96436	
PRPF38B		-3.54769		-3.40695	-2.62022
VEZF1		-5.17099			-4.41785
MCM5	-2.79547		-2.34014	-2.01653	-2.44096
BABAM1			-5.48066	-2.07425	-2.06585
USP39		-4.78456		-4.84904	
GMPPA	-4.97451			-4.66765	
CYP51A1		-3.73038		-5.96196	
CPVL		-5.4424			-4.2537
HBA2	-2.72749	-2.92646		-1.61603	-2.4367
LMAN2	1.141305	-2.34893	-2.28961	-3.50628	-2.70462
PBRM1	-4.78234				-4.92936
GLG1	-1.89069	-3.92503	-3.916		
HM13		-5.05068			-4.69261
MGST3	-3.14725	-2.9052		-1.57709	-2.11466
MCCC2	-3.11452	-1.47809	-3.21069	-1.96365	
LIN7C	-2.84649	-3.059			-3.89662
PRKCSH	-2.0408	-2.62216		-3.88253	-1.26171
TMA7	-2.73546			-3.1863	-3.88632
STT3B		-3.58404		-2.84638	-3.39842
MRPS2		-3.96438	-2.35695		-3.52165
DNAJC11	-3.65274	-3.00881			-3.1818
HINT2	-1.64731	-3.33558	-4.86458		
RAE1	-2.294		-4.54597	-3.01205	
PDS5B	-2.29458	-3.72636	-3.83438		
PARK7	-3.00919	-3.22959	-2.84157	1.811951	-2.59614
VPS36		-3.10364		-3.30757	-3.46168
SF3B3				-4.62915	-5.25897
WIZ	-6.98563	-2.90547			
NQO2	-4.66075		-5.23061		
KHDRBS2	-2.10822			-4.77704	-3.0101
CUL2	-5.26537		-4.64689		
CHD1	-4.99269		-4.93317		
RBM15		-3.47773	-3.20078		-3.26179
SURF4	-2.70421	-3.35777	-1.09424	-2.81766	
TBC1D15			-5.3761	-4.60841	
NOSIP		-3.3716		-3.5572	-3.06229
HECTD1	-4.53146		-5.46885		
MAP1B		-4.05975		-1.73794	-4.20494
NCDN	-4.05425			-3.49103	-2.48503
CNBP		-3.15136	-2.31735	-1.97395	-2.58908
PHIP				-2.97956	-7.05966
UBR4	-3.13122	-2.40717	-2.48306	-2.02164	
ABCF1	-5.11395		-4.95057		
CANX	-3.01042	-4.67975	-5.74233		3.360125
RBM28				-3.00508	-7.07548

STAU1		-3.48621	-3.13367		-3.4887
HAUS5			-5.00363	-5.11961	
UTP14C		-3.05368		-4.04766	-3.02347
PMPCA	-5.08401		-5.04885		
SON		-2.75123	-1.91385	-2.41511	-3.08456
SEC23B	-4.18458		-2.82511	-3.16532	
HUWE1	-5.18949		-4.9915		
GLA	-3.52324		-4.1116	-2.55036	
TMEM33		-5.39322		-4.82711	
RP2		-4.73142		-5.49054	
PPWD1		-4.07591		-3.85409	-2.29802
AP3S1	-5.43045			-4.81824	
RAB1C	-5.91716		-2.5459	-1.79511	
EIF3L		-3.27529	-2.32363	-4.66606	
BMI1		-3.34419		-3.53182	-3.40366
COMMD3-BMI1		-3.34419		-3.53182	-3.40366
CALU		-6.45784			-3.82231
AHI1	-4.09986		-2.72808	-3.48705	
38412	-5.51515				-4.82794
C21orf33		-2.84652		-4.76611	-2.7338
SRP54	-2.7101	-2.43348	-2.65692		-2.55618
SLC25A22	-4.37147			-5.98652	
UBR7		-4.24704		-3.95849	-2.16714
CDCA8			-5.2425		-5.13705
CHAMP1	-2.23695		-2.9058	-2.21022	-3.02848
TXNRD2	-2.67099		-2.82549	-2.3184	-2.57116
XPO6		-3.04183	-1.55413	-3.58472	-2.21336
PPP2CB	-3.02403		-2.20384	-2.32244	-2.86148
PTGES2			-2.02908	-3.66506	-4.72322
YWHAG		-3.1595		-3.39118	-3.87865
NAA40	-5.09673	-5.35421			
CCDC88A	-1.92896	-3.70426	-4.82441		
ERP29		-1.11872	-2.98785	-3.59091	-2.76059
NOMO1	-2.18268	-2.67801	-3.19347		-2.41687
FAM175B			-5.17507	-2.74936	-2.54773
DCTN1	-3.68118		-2.08564	-2.54467	-2.16343
DDX56			-5.34119	-5.17294	
RSL1D1		-4.1026		-3.17901	-3.25101
RRBP1	-4.40824			-2.38138	-3.77629
MRPL17			-1.97804	-3.95169	-4.64126
TOR1AIP2	-2.49563			-4.8882	-3.19497
FANCD2	-4.39534	-3.39663		-2.78915	
LMNB2	-2.59598	-2.65637		-1.89723	-3.4507
PTPN1	-2.41264		-6.11431	-2.10032	
WBP2	-5.15898		-5.48695		
POLDIP3	-2.49825	-3.22718		-2.60937	-2.32863

PPFIA1	-4.48538			-3.82195	-2.36631
OGFR	-3.77385			-3.68489	-3.22563
RRP1	-1.81888	-4.05443	-4.83157		
SPC24	-3.18572		-2.63221	-2.65616	-2.26324
PRRC2B	-5.33384		-5.41079		
IDH3G				-6.39724	-4.35597
SLC1A5		-5.35869			-5.40056
RPL34		-3.90934		-3.53235	-3.33301
PQBP1		-3.5691	-3.19545	-4.01847	
QRSL1		-4.83718		-3.0823	-2.87874
SUMO2		-3.91762		-3.14314	-3.74358
SLC25A1	-1.50294	-5.60842	-1.60133		-2.09547
MECP2	-1.65892	-2.9638	-3.79068		-2.40192
DLST	-2.48366	-2.4251	-2.5143		-3.39986
COPS3		-5.48223	-5.36776		
MPP2		-3.76485		-3.67235	-3.44247
TCOF1	-1.45164	-4.49974			-4.93249
GNAI2		-3.16008		-3.74314	-3.99447
RALY	-2.60518	-1.28507	-3.12544	-1.75179	-2.13179
STARD9		-4.21639		-3.06675	-3.61982
FAM136A	-2.05619	-4.64206	-1.89956	2.335816	-4.64167
CLIP1	-5.52272		-5.39031		
MRPL18		-5.24861		-5.67797	
POLB		-7.79241	-3.1356		
U2SURP	-2.10316	-2.99074		-3.62931	-2.23401
TAMM41		-3.36405		-4.66698	-2.93195
GNG12		-3.39531	-3.44012	-4.15533	
H2AFY2		-3.33639		-2.34605	-5.30917
TIA1		-3.45806		-3.12812	-4.4088
PEPD		-2.76497	-5.4764		-2.75626
EIF2D	-5.40286		-5.60344		
MORF4L2	-1.70126	-3.85077	-5.46515		
POFUT1				-5.75475	-5.26284
NDUFA10	-3.24752	-3.60031			-4.17017
RPL36		-3.59391		-4.2835	-3.14444
CHMP4A	-5.48369		-5.55014		
EEF1D	-3.18076	-4.22798	-3.63028		
RHOT1		-3.74272		-7.31573	
MRPS18B	-2.90244	-2.86728		-2.89248	-2.41458
MAGT1		-4.08106		-3.27053	-3.77396
SAFB2		-3.06912	-4.60543	-3.46055	
SNX17		-2.58648	-3.13903	-2.83425	-2.59391
HK1		-4.09056		-3.54464	-3.55586
PNPT1	2.721037	-5.0439		-4.17426	-4.70661
TTC19		-4.16135	-2.49252	-2.59786	-1.96251
NOL11	-2.47084	-3.26638		-2.05178	-3.49127

MIA3	-3.65132	-2.27298			-5.36818
FSCN1	-1.74446	-2.76885	-1.97186	-2.95836	-1.85797
CSNK1E		-3.78414		-3.12889	-4.39302
DHRS7	-2.28325	-4.54783		-2.20695	-2.26981
SAMM50	-2.2448	-2.26097	-1.07063	-3.56661	-2.17178
GOLGA2	-2.7806	-2.51629	-2.91372		-3.10505
RPS15		-4.31127		-4.86189	-2.14704
ORC3	-6.17445	-5.20798			
PSAP	-2.69631		-3.71244	-2.40718	-2.56886
RHOA		-4.17416		-3.13484	-4.07863
RHOC		-4.17416		-3.13484	-4.07863
HSPE1	-4.14218	-5.73122	1.111033		-2.63475
GNS		-3.14873	-2.4517	-2.61484	-3.19029
PTMS	-4.00092			-3.7107	-3.70088
NIFK	-4.20641	-2.60795	-3.10476	2.860583	-4.37299
MCMBP	-2.62091	-2.63354	-3.32203		-2.88382
RAB11A		-3.21053		-5.04698	-3.23679
DNAJA2	-2.16084	-2.8405	-3.11171	-3.41109	
TAF15	-1.11609	-4.19254		-2.22342	-3.99674
TIMM44		-2.8853		-4.98545	-3.66437
ZFP91		-4.0916	-3.78816	-3.68301	
NOC3L	-4.32896		-3.1959	-4.05198	
SNTB2		-3.39082	-1.19795	-3.46232	-3.55232
RFC4		-4.56736		-3.41543	-3.62315
UFL1	-4.8813	-3.11457			-3.62016
ESYT2	-4.70622			-3.42465	-3.49604
CTNNA2	-6.41266		-2.42278	-2.80351	
PRKAR2A	-4.4237	-3.1157	-4.10646		
GINS2		-4.08285		-3.3788	-4.20023
INTS7	-4.39934			-4.07894	-3.1932
FANCI	-2.04489	-3.39482	-2.12464	-1.93601	-2.18894
TEX264		-5.50303		-4.673	-1.51501
HMGN1	-3.5904	-3.4589	-5.82133	3.839661	-2.69414
CD2BP2		-3.92739		-3.43418	-4.45231
RPS4X		-4.29997		-3.34056	-4.19123
GPI	-4.13662	-3.30016	-4.39904		
RPL23		-4.35232		-3.24746	-4.25723
RPL29		-4.33445		-3.28229	-4.2508
GPHN	-5.13424		-4.47534		-2.26148
RQCD1	-2.60985		-2.47862	-3.42139	-3.37351
DDX54		-4.23121		-3.12502	-4.52775
ZNF330	-4.89762	-3.81254		-3.17876	
NOL8	-2.55287	-2.71579	-4.00582		-2.62134
ARFIP2	-1.52336	-4.24947	-2.01632	-1.80654	-2.31031
RAB25		-4.57789		-3.17212	-4.15641
NUP35		-5.65169		-6.26674	

CEBPZ	-3.38903	-4.01362	-4.52096		
AMOTL1		-3.46536	-4.04102		-4.42853
P3H1		-4.0658		-3.24516	-4.64703
LEPRE1		-4.0658		-3.49117	-4.41562
C1orf177				-6.14604	-5.85764
PSMD9	-3.35861		-3.03762	-3.61609	-1.99158
PUM1		-3.18742		-4.8133	-4.01103
PUM2		-3.18742		-4.8133	-4.01103
NOP2	-4.02532	-3.99496	-4.00229		
WNK1		-4.37825		-3.36277	-4.29584
PRPS1	-4.5759	-4.57822			-2.88845
YWHAQ		-3.60965		-4.08177	-4.35623
ALDH2	-2.33637	-4.41162		-2.14713	-3.17587
SHMT2	-2.19682	-4.88627	-2.4464		-2.5612
MARCKS		-4.3254		-3.57386	-4.22823
CD99	-2.20646	-4.33527	-5.59922		
NAA40		-3.06197	-3.75137		-5.33189
COMMD2	-4.68347		-5.19203		-2.27032
ERGIC1		-3.71089		-4.85396	-3.58105
NIN		-4.08223		-3.45684	-4.63058
NUDT19	-4.80841		-3.96908		-3.4135
CYB5B	-2.13833	-6.36358	-1.99823	-1.69774	
KIAA1429		-4.32936		-3.8027	-4.09367
TTF2		-4.62721		-4.06148	-3.55086
GNL2	-4.0141	-4.31173		-3.93002	
NUP98		-5.01518		-5.07406	-2.18152
ANXA11	-4.83932	-6.09241		3.679571	-5.02165
RCN2	-2.22305	-3.719	-3.63816		-2.73821
PEX19			-4.26432	-3.47768	-4.5816
MPP6		-4.22852		-3.31354	-4.81562
TXNL1	-2.86852	-2.8697	-3.61911		-3.0023
SCO1		-3.83004		-4.48657	-4.05078
RPS3	-2.01822	-2.55162	-3.55774	-1.56856	-2.67984
TMEM9		-3.89816		-3.98584	-4.49315
TMEM9B		-3.89816		-3.98584	-4.49315
REXO2		-4.14807		-3.63487	-4.6216
GFM1		-3.92401		-4.14499	-4.35653
PPT1	-2.07039	-3.74877		-4.11602	-2.5019
NUP214	-1.68229	-4.20825		-2.22845	-4.42111
LAMP1	-3.93566	-4.47683	-4.13091	-4.07177	-4.80425
EPS15L1		-2.87605	-7.09056	-2.61561	
AP1M2		-2.68188	-6.72004		-3.19183
TSPAN6	-3.15139	-3.43666	-2.16084		-3.84898
CSTF3	-2.3532	-2.50969	-3.10436	-2.30972	-2.34044
MINOS1	-2.92028	-3.00144	-1.87482	-1.84714	-2.97948
RRAS2	1.514699		-4.79039	-4.17827	-5.17082

TOMM40	-3.06733	-2.23684	-4.87165		-2.46254
AKR1B15		-4.68028		-3.45482	-4.53834
PSMD4	-4.45316			-4.61839	-3.62627
TIMM9	-2.7743	-3.23198	-1.96532	-1.54536	-3.19724
MRPS28		-5.12944	-2.18607	-2.68523	-2.75681
RCN1		-5.86473	-5.18567	-1.7513	
NDUFA2			-4.923	-5.37595	-2.54815
MCCC2	-4.84016		-2.54862		-5.47081
SEC63		-4.76043		-3.75306	-4.40971
EEF1E1		-6.86054			-6.06483
NDUFA6				-6.66898	-6.27734
UQCRHL		-2.44412	-5.36534	-5.14707	
BRD2	-2.34625	-2.28683	-3.44393	-1.57751	-3.31565
LMAN1		-3.12265	-7.43399	-2.44027	
CHEK1		-4.51662	-4.10878	-4.38117	
AMOTL2		-4.54886	-4.04102		-4.42853
SND1	-2.71156	-4.81229	-5.49595		
USP5	-4.23363	-3.63461	-5.16359		
CTCF	-2.71075	-4.8681	-3.24057	-2.21565	
MT1A	-2.05102		-5.83746	-2.62587	-2.52434
MT1H	-2.05102		-5.83746	-2.62587	-2.52434
HEATR6		-3.60184	-2.91466	-3.18711	-3.36476
ATP1A1	-2.03129	-3.84389	-1.60389	-2.45889	-3.18599
NDUFAB1	-5.81197		-2.67535	-2.03737	-2.66465
GOSR2		-3.95512	-1.65214	-3.88284	-3.72695
HIST2H3A		-6.64183	-2.20666	-4.39613	
EIF4G1	-3.15117	-3.32379	-2.21606	-2.20032	-2.36824
FARSA		-2.86129	-4.02699	-4.1994	-2.18969
DHRS7B	-4.58462			-4.33758	-4.38677
LBR	-4.26928	-4.25659		-2.06485	-2.73026
COX6B1	-2.10898	-4.96415	-4.38356	-1.8648	
PRPF31	-2.02676	-3.48579		-3.39862	-4.41143
PAWR	-4.58111		-1.90414	-3.30653	-3.54287
DHCR7		-4.39681		-4.15341	-4.79363
RUFY2	-2.71467	-4.60552	-4.30194	-1.72434	
WFS1		-4.48343	-1.96365	-3.12814	-3.78196
GINS1	-4.55183	-4.85955		-3.95648	
COPS7B	-2.27656	-3.36173	-2.5223	-2.48199	-2.72831
MRPL22	-2.65908	-3.29908		-4.05526	-3.36935
UGGT1	-5.11683	-2.76358	-3.05656		-2.46279
MRPL43	2.253248	-5.66423		-4.75568	-5.23463
AKAP12		-4.41787		-4.0751	-4.91019
HSPE1-MOB4	-3.23368	-4.0111		-3.12631	-3.05904
GLOD4	-4.39466		-4.95115		-4.09903
HMG20A		-4.67572		-4.56977	-4.19991
MMS19	-5.22148		-5.76786		-2.45932

RABGGTA	-4.68242		-4.35548	-4.43311	
UBA52	-2.76689	-2.88389	-2.68362	-5.14893	
DDA1		-3.66045	-4.0701	-3.04059	-2.7321
ST13		-4.1207		-4.78378	-4.63474
HP1BP3		-4.50836	-3.14241	-5.88943	
PSMD12	-2.98997	-2.80551	-2.71074	-2.4264	-2.61373
NDRG3		-6.44112	-4.13843	-2.97375	
GNAI2		-5.05678		-3.81338	-4.69627
MRPL45	-2.96006	-4.72601		-3.02012	-2.8789
MECR		-4.9728		-3.75859	-4.87063
OXCT1		-4.63161		-4.13724	-4.86413
ACSS1		-5.01948	-3.97647		-4.68342
KIAA0415		-6.19186		-3.64867	-3.89251
CLPX		-4.63526		-4.93575	-4.20839
NGDN		-4.45889		-4.68392	-4.64443
RNF2	-2.33388	-2.36074	-3.28179	-1.98143	-3.86897
SLC4A1AP	-2.93603	-3.69995		-3.00956	-4.1833
TFAP4	-4.22933	-5.57836	-4.03547		
PPME1	-2.64273		-5.17564	-2.53922	-3.48733
UGDH		-5.01554		-3.7477	-5.08656
SLC7A5	-4.36013		-2.18399	-3.17012	-4.14879
TOMM22	-2.28308	-4.30146	-2.08185		-5.20308
EIF4B		-4.7178		-4.38881	-4.78759
CALR	-5.35053	-2.69299	-4.64149	2.936833	-4.15868
GAA		-4.06222	-2.74431	-3.24698	-3.85976
MPI	-5.76336		-5.21043	-2.96149	
MAX		-4.74957		-4.84326	-4.35054
EIF3K		-4.64853	-4.06543	-2.74588	-2.48863
RFC5	-5.43826	-2.47188		-2.7125	-3.33095
SUCLG1		-3.46448	-2.2409	-3.96362	-4.35338
SAE1	-2.19875	-4.24549	-4.93982	-2.64291	
MMS19		-4.49643		-4.49933	-5.03348
ATP6V1C1	-4.76679	-3.36457	-5.94906		
TIMM10	-4.3286	-3.09454		-3.16109	-3.49763
UTP15		-4.58118	-4.53769	-4.98248	
TRUB1		-4.43838		-4.2708	-5.41943
TIMM13		-3.68461		-4.36592	-6.07823
ARF4		-3.89356	-3.36684	-3.37664	-3.50419
TLN1	-3.63884	-3.06067	-3.12949	-4.33643	
CKAP2		-4.47804	-3.02743	-3.47532	-3.19921
CLPTM1		-4.89544		-4.36164	-4.96857
ANKFY1	-4.80386		-4.51406	-4.91232	
TFRC	-4.18854	-5.57094		-4.49902	
SERPINH1		-4.26912		-4.57537	-5.41792
TXNDC12	-3.01486	-6.43455	-4.87861		
PTGES3		-4.88916		-4.39515	-5.07585

RPA2		-4.81456		-4.22974	-5.32955
CHMP2B		-5.13502	-2.03458	-3.13415	-4.08104
TPT1P8	-4.77998	-5.22209	-4.39875		
LOC102724023	-4.06112	-2.84652		-4.76611	-2.7338
ARMC6	-3.44215		-3.32637	-2.76764	-4.91041
WDR26	-3.12993		-3.98745	-3.7618	-3.57455
ARG1		-3.17529	-3.21918	-2.7248	-5.36024
CDC42	-2.32122	-3.98768	-1.93861	-3.0633	-3.18881
SLC35A4		-5.3384		-4.1907	-5.00722
HGS		-5.25833	-4.68167	-4.5978	
AIFM1	-3.06203	-5.37535			-6.11288
MRPS11	-2.77407		-2.97123	-5.17664	-3.66401
M6PR	-3.56131		-3.51921	-3.39562	-4.16329
RHOJ	-5.16093	-5.57203		-3.96436	
RHOQ	-5.16093	-5.57203		-3.96436	
SMARCD2		-2.59053	-4.82123	-3.05095	-4.23525
DNAJC5	-4.00914		-4.0936		-6.61822
MRPS7		-3.14333	-3.63385	-3.63196	-4.34917
MAVS	-4.0558	-5.39016			-5.33162
RBM34	-3.8921	-3.50372	-2.50876	-2.1255	-2.74909
ASNS	-3.26016	-3.08155	-3.37231	-2.27543	-2.8064
PUS7	-5.16088		-3.97028	-5.66554	
PELP1	-3.34478	-4.07796		-3.23482	-4.15884
NFKB1	-2.38713	-4.68233	-3.24169		-4.53294
BCLAF1		-4.0649	-2.94167	-3.36038	-4.48002
GABPB1	-3.47805	-3.35808	-2.18386	-2.48375	-3.35332
BRAT1	-5.56477		-4.48034	-4.81625	
ACTN1	-3.48866	-2.7941	-3.21103	-2.44676	-2.9254
MSN	-2.83167	-3.36124	-3.56515	-2.47958	-2.64647
STIM2	-1.77339	-4.69229		-3.65666	-4.77094
SUCLG2	-3.55896	-3.9432	-5.67396		-1.7967
LIMD1	-4.68498		-5.08561	-5.2122	
AHSG	-5.48552		-4.61205	-4.88612	
HDAC3		-4.78826	-4.79266	-2.79245	-2.63155
SAP18	-1.76106	-3.69719	-4.28512	-1.93191	-3.35724
RSF1	-6.21257	-3.85022		-3.04494	-1.94852
NOLC1	-1.35961	-5.73305	-3.15028	-4.85054	
SRM		-5.48838	-2.14884	-1.92881	-5.55824
BICD2	-4.67942		-5.56768	-4.88815	
39692		-5.68145	-4.49969	-4.96534	
PEF1	-4.96097	-5.20455	-4.98645		
ZNF512B		-5.338		-5.09391	-4.76411
NT5DC1	-5.17546		-5.34986	-4.67206	
THUMPD3	-5.17619		-5.33398	-4.71389	
GLMN	-1.81899	-4.22006	-2.30759	-4.64907	-2.24806
LIN28B	-1.99355	-4.2553	-4.92341		-4.07615

DDX39A	-4.00151	-4.30846		-3.01825	-3.94828
GIGYF2	-5.23002		-5.59673	-2.3379	-2.16514
UBE2M	-4.24912	-2.81917	-4.09508	-4.1678	
MRPS5		-5.56579		-4.61271	-5.15872
SKIV2L2	-5.28725			-4.69759	-5.35572
CRYZL1	-5.3252			-4.83561	-5.18759
SRP19	-3.37202	-3.93829	-4.89042		-3.15809
PRCP	-4.76167		-3.354	-2.67971	-4.58639
DKFZp586I031	-4.15888	-4.361	-3.68584		-3.20434
NPM1		-5.08287	-2.22782	-3.04395	-5.05716
RPL31		-4.06908	-4.00043	-3.75689	-3.62684
ACTR3B		-5.7271		-4.39891	-5.32881
UQCRQ	-2.11449	-4.29262	-4.67424	-1.56468	-2.81289
GNB1	-5.49391	-4.09386	-5.87767		
COMT		-5.71595		-4.57047	-5.18447
C1orf131		-4.53457	-4.21106	-2.33745	-4.39226
RRP7A	-4.3798	-2.91847	-4.43268		-3.74879
EIF2B4	-5.3329		-5.46787	-4.70733	
CKMT1A	-2.3101	-5.85969	-1.84		-5.50847
CKMT1B	-2.3101	-5.85969	-1.84		-5.50847
OPA1	-1.95731	-2.98096	-3.84387	-1.4548	-5.28601
CKMT2	-2.3101	-5.85969	-1.89499		-5.50847
DEK	-3.77993	-3.6507	-3.95312	-2.04949	-2.14392
GNG5	-1.6705	-3.51748	-4.27021	-4.84613	-1.30281
SACM1L		-5.55736		-4.73477	-5.32369
TKT	-5.18734	-4.78226	-3.45294	-2.20249	
H3F3A		-6.76445	-2.71014	-4.24893	-1.92272
TIMM8B	-3.98872	-5.30694	-6.35228		
TPM3		-6.2907	-4.55812		-4.82147
RBM22	-2.14895	-5.78662		-2.1559	-5.59411
CCDC40	-2.79399		-4.36784	-4.37256	-4.16978
YTHDF1		-5.5768		-4.77046	-5.37453
IST1	-4.64282	-3.30526	-3.92534	-3.8535	
PRPS2	-4.44689		-3.87921	-3.26656	-4.18426
DNAJB2	-6.03464	-4.84711		-2.38711	-2.54064
DNAJB8	-6.03464	-4.84711		-2.38711	-2.54064
BTF3	-5.36551	-4.52325	-2.23324	-3.69326	
USP47		-5.63435		-4.90651	-5.32244
GFAP	-2.64665	-4.53738	-3.99645	-2.26319	-2.4291
DHX29	-5.42581		-5.41687	-5.04615	
BZW1	-5.1836	-2.7509	-5.3713		-2.63846
WHSC1		-5.16936	-5.41773		-5.36385
SNW1	-5.55327	-5.64928		-4.80456	
PRPSAP1	-5.64579	-3.71109	-3.31804	-4.55715	1.221887
TBC1D10A	-5.22328		-5.20582	-5.58217	
ERP44	-2.89223	-3.31769	-4.16772	-5.64274	

ARF3		-4.22191	-3.54668	-4.73116	-3.53436
TMEM14C		-3.58827	-5.22974	-7.21915	
LONP1	-3.83915	-2.86443	-4.1874	-2.54906	-2.61854
SNRPB2		-4.62129	-4.71635	-2.01677	-4.7376
DKFZp434B2017		-4.14403	-6.64535	-2.69994	-2.61401
EPPK1	-5.4896	-3.04698	-4.88533	-2.70167	
GEMIN4	-5.29151	-5.54959	-5.2825		
MAEA	-6.3216		-5.08716	-4.72551	
VASP		-5.70242	-5.6392	-4.8337	
LCMT1	-5.21382		-5.56951	-5.42072	
TRIP6	-4.20755	-4.24489	-4.03938	-3.7342	
SAR1B		-4.30769	-6.64535	-2.69994	-2.61401
TBCA	-4.20508	-4.52328	-2.27609	-2.49885	-2.86716
ARKL1	-4.97984		-5.85681	-5.53419	
C18orf25	-4.97984		-5.85681	-5.53419	
TMEM43		-5.33729	-4.5333	-2.50588	-4.02813
RBMX	-5.33708	-2.61962	-5.25035		-3.21317
SCRN1	-4.59368	-4.2047	-3.76325	-3.87301	
MRPL12	-2.38675	-3.73615	-2.8855	-5.06965	-2.40367
TUBB3	-4.73396	-7.05714	-4.78903		
RPE	-3.28054	-3.64313	-3.82479	-2.60527	-3.23376
CHMP2A			-8.38675	-5.12912	-3.0744
COASY		-4.06602	-4.54123	-3.46803	-4.55141
VDAC3	-4.53121	-5.48841	-1.49345		-5.14844
MT1X	-2.9782	-2.78665	-5.79498	-2.85535	-2.26362
MT2A	-2.9782	-2.78665	-5.79498	-2.85535	-2.26362
CD63	-3.44268	-3.69317	-3.23669	-2.8397	-3.47028
ASF1B	-3.65531	-3.21335	-3.26664	-3.81284	-2.7483
DPP9		-4.628	-4.66818	-3.3206	-4.09365
SOD2	-5.91184		-3.26194	-5.34618	-2.19625
FAH		-5.1045	-4.88911	-3.01662	-3.75371
DAD1	-2.12371	-4.53747		-5.35563	-4.79011
SCP2	-4.91417	-2.95046	-2.49781	-3.12866	-3.32114
NKAPL	-4.29071	-4.88678	-4.20526	-3.46807	
NDUFB6	-5.42338	-5.46366			-5.98308
SRA1	-4.82263		-7.28302	-4.76655	
RDH13	-3.98578		-4.49534	-4.0922	-4.31779
LDHC		-4.32451	-4.04382	-3.8309	-4.71956
MT1G	-2.9782	-2.78665	-5.79498	-2.85535	-2.52434
CBS	-5.30031	-4.80775			-6.83911
RCCD1	-5.78196		-5.71506	-5.51246	
VARS	-5.93881	-4.2199	-4.83735		-2.02383
TARDBP	-4.3339	-4.24187	-4.27821		-4.18102
SBDS	-4.29414	-4.84746	-4.18501	-3.72805	
GFPT2	-3.50895	-4.38267	-2.97668	-2.79141	-3.40927
DACH2		-3.80242	-5.92202	-3.71579	-3.65165

ELP2		-5.4057	-5.79084	-5.932	
MTA3	-6.1469	-3.67048		-3.38313	-3.92985
SLC9A3R2	-4.40207	-4.60387		-4.32164	-3.81287
ME3	-2.76386	-5.64892	-3.08055	-2.54698	-3.14328
METAP1	-4.06419	-4.45492	-2.49851	-3.25451	-2.91982
CPSF7		-5.77997	-4.6965	-4.5391	-2.20088
SUB1	-5.39265	-3.46832	-5.5043		-2.87185
HNRNPH1		-8.77373			-8.47423
HRNR	-1.71872	-4.44977	-3.99967	-2.31803	-4.81883
MTDH		-4.74009	-3.86663	-3.49204	-5.22578
POR	-1.81961	-3.25148	-3.92775	-3.72029	-4.60571
SMN2		-4.68197	-5.42319	-3.89829	-3.36153
HMOX1		-5.7123	-2.73206	-4.51003	-4.48586
CLTA	-5.15511	-5.30729	-2.45955	-4.54192	
UTS2	-4.86828	-4.74526		-3.31108	-4.57745
BAX	-3.32021	-3.99552	-2.55488	-3.41644	-4.22591
ACADM	-4.719		-5.41325	-2.90126	-4.52815
EZH2	-2.5748	-4.89205	-4.01851	-6.07954	
C11orf98	-4.09917	-4.16106		-4.73401	-4.58209
CPNE1	-5.57377	-3.72216	-5.46115		-2.86431
PTMA	-1.395	-5.75057	-5.44764	-5.03521	
RRP12	-5.36632	-3.05996	-4.28759	-2.77763	-2.16175
SLC25A24	-9.53643	-5.10331	3.690906	-4.55124	-2.16841
UQCRFS1P1		-5.66671	-2.21142	-4.53859	-5.26609
RANBP9	-6.40128		-1.94245	-4.73833	-4.60493
DBN1	-1.28424	-5.11847	-2.24577	-4.05272	-5.01118
ASMTL	-5.75089		-6.92835		-5.04693
RUFY1		-4.60552	-1.68794	-4.88068	-6.5686
RUFY3		-4.60552	-1.68794	-4.88068	-6.5686
SMN1		-4.68197	-5.42319	-3.89829	-3.79806
ZMYM3	-2.01258	-7.44503	-3.11302	-2.37103	-2.87086
ATPIF1	-2.46938	-5.39197		-4.93296	-5.06111
TSC2		-4.64575	-3.29363	-4.72018	-5.23535
DCAF16	-5.52875	-2.60806	-4.54073	-3.0766	-2.1851
RPL36AL	-5.08422	-5.67877	-2.21485	-4.96381	
RPL8	-4.67955	-4.84678	-4.26895	-4.20286	
MCM4	-2.25471	-5.59441	-5.47762	-2.07439	-2.6051
CEP41	-4.30699	-5.141	-2.31975	-1.81595	-4.42394
MRPS9		-3.85657	-5.63076	-3.95807	-4.58373
RAB1B	-4.18665	-4.65953	-2.18092	-5.01685	-2.09605
NCL	-5.1951	-4.88912	-2.94944		-5.13221
GLS	-3.29643	-2.92437	-3.29424	-4.94899	-3.70304
ARL2BP	-4.18843	-4.7356	-4.28252		-4.99929
RPS23	-4.84824	-5.02267	-3.05825	-2.07181	-3.22179
CHMP1A		-4.42856	-4.27688	-4.70928	-4.82755
BZW2	-5.19822	-3.51046	-3.67222	-3.30828	-2.56986

MEPCE		-5.42665	-5.49277	-4.63997	-2.70672
LPCAT1	-4.15719		-4.33641	-4.55881	-5.23647
SDAD1	-4.46135	-4.25271		-5.14734	-4.45345
DKFZp434B156	-4.5162	-4.63979	-4.4711		-4.71204
WDR92	-4.5162	-4.63979	-4.4711		-4.71204
MDN1	-2.56547	-4.54175	-3.78285	-3.27771	-4.18968
OCIAD1		-2.57664	-4.65574	-6.09395	-5.08529
TPP2		-4.98151	-4.10636	-4.91478	-4.48483
HYPK	-3.71704	-4.22669	-4.34784	-2.91311	-3.29858
PRPF40A	-1.68373	-5.18964	-1.96283	-4.61975	-5.10291
FTH1	-5.75942	-5.22908		-2.72126	-4.86123
KPNB1	-3.16513	-8.46766		-3.0591	-3.90285
IMMT	-1.36714	-5.81508	-1.31111	-5.04069	-5.14899
PPP1CB	-4.02021	-3.71616	-4.05608	-3.04163	-3.94469
DDI2	-5.39539	-3.08091	-5.28125	-2.02811	-3.08391
SF1	-2.05474	-5.22209	-2.19975	-4.36768	-5.07027
TSFM	-7.0763		-7.48703	-4.38379	
CNN2	-6.17768		-5.64357	-3.18052	-3.95121
APOBEC3C	-4.1035	-4.30468	-3.84372	-3.01634	-3.69735
SF3A2	-5.23048	-2.97988	-5.14375	-2.63277	-3.10627
TLE4		-4.46563	-5.29177	-4.7632	-4.59071
NEFM	-2.54426	-3.61926	-5.20928	-3.61604	-4.13612
RAB11B		-3.21053	-5.42546	-5.04698	-5.45634
CNNM3	-4.66971	-5.70257	-4.31566	-2.33552	-2.15286
ATPIF1	-2.51958	-3.37858	-2.92975	-5.32616	-5.05754
NKRF	-3.70094	-4.08181	-4.49043	-3.79031	-3.15827
TPM2	-8.14504		-6.30169	-4.82843	
MT1E	-1.90839	-5.65408	-5.72035	-3.45283	-2.58518
MT1M	-1.90839	-5.65408	-5.72035	-3.45283	-2.58518
HMGN2	-2.09869	-7.09145	-5.01563		-5.13983
REEP5	-2.59855	-3.84474	-3.26312	-4.27733	-5.3644
PLRG1	-8.04049	-2.78957	-1.86596	-3.37825	-3.3182
RPL28	-4.05301	-4.23497	-4.12877	-3.82166	-3.18338
HNRNPM	-4.6232	-4.70853	-2.98092	-3.54394	-3.60499
HLA-B	-3.64988	-5.34063	-2.8023	-3.83091	-3.83927
HLA-C	-3.64988	-5.34063	-2.8023	-3.83091	-3.83927
LSM4	-5.11358	-4.12838	-5.12604	-2.45591	-2.69135
LRBA	-4.64861	-4.51033	-4.36293	-6.02414	
ASPH	-5.77709	-2.63497		-5.86088	-5.28466
ATXN2L	-2.08421	-5.6015	-5.16924	-4.98815	-1.71639
INIP	-4.27096	-4.9338	-5.69663		-4.70403
GIPC1	-4.06366	-4.5397	-2.21413	-3.82047	-4.97017
HEL-S-109		-4.69888	-4.88773	-5.72282	-4.31641
NUCB2		-4.69888	-4.88773	-5.72282	-4.31641
SETBP1	-4.63812	-4.44592	-2.86359	-2.62184	-5.06662
SUMO3	-4.09943	-4.06265	-4.40691	-3.18767	-3.89006

NDUFA5	-4.13452	-4.9973		-5.39188	-5.12436
PPHLN1	-6.32944	-5.10481		-3.76064	-4.57532
KARS	-6.11055	-4.55625	-6.04847	-3.11038	
HSDL2	-4.27402	-3.4536	-4.56017	-3.42502	-4.17366
HAX1	-4.00028	-4.31192		-6.11799	-5.4582
SNRPD2		-4.05667	-5.92684	-5.22175	-4.72206
LMAN2L	-4.01871		-4.91898	-5.42342	-5.61509
ATP5F1	-2.94982	-5.58179	-4.8878		-6.55835
AGK	-1.17916	-4.36255	-4.83768	-3.98762	-5.63657
ERH	-1.20376	-5.72022	-3.42412	-4.36445	-5.29725
WDR18	-5.0344	-5.24793	-4.58206		-5.16225
PTBP2	-6.16541			-7.02535	-6.8366
CBS	-1.63566	-5.75762	-5.59167	-5.88416	-1.23532
CECR5	-4.88664	-5.25284		-5.70863	-4.30323
RPL37A	-4.46198	-5.01253	-4.38399	-4.30362	-2.03255
PDCD4	-4.523		-5.51754	-5.28284	-4.8968
AK4	-2.97859	-4.62678	-4.88446	-3.77172	-3.96586
IDH3A	-5.18459	-4.44182	-3.14165	-3.30479	-4.18567
CD59	-4.75384	-4.91396	-4.78659	-2.14399	-3.67455
RDX	-5.77313	-4.14441	-1.64209	-4.38709	-4.40888
PPIH	-4.31308	-4.06963	-4.02571	-4.36541	-3.63771
H2AFX		-6.62986	-5.72369		-8.08532
COA3	-5.10828		-5.69513	-4.55435	-5.16686
RPL10	-5.51928	-3.34035	-5.69653	-3.86441	-2.13874
SH3BGRL3	-5.28028	-5.44046	-5.37256	-4.52144	
LSM8		-4.77841	-5.66161	-4.97028	-5.25816
RPL27A		-10.0358		-2.10736	-8.54864
STMN4	-4.76667	-4.16353	-5.02247	-3.33121	-3.44606
IDH2		-4.05946	-7.86899	-4.09984	-4.72868
TBRG4		-6.20699	-8.01511	-6.55018	
SMG9	-4.10003	-5.55353	-3.34812	-3.84959	-3.92629
SEC62	-4.54965	-5.2489		-6.45647	-4.55023
PES1	-5.21455	-5.55959	-5.60867	-4.42919	
TCOF1	-5.05379	-2.69368	-6.64848	-3.11296	-3.3235
NDUFAF4	-5.72997	-3.9432	-4.83802	-6.33355	
STX7	-5.22226	-5.65907	-5.34347	-4.68951	
DPM3	-4.90599	-4.34918	-4.40864	-4.13019	-3.14252
FDPS	-2.66969	-7.34411	-4.13682	-3.0395	-3.78747
PHPT1	-5.3406	-3.65272	-5.20599	-2.7562	-4.02919
C14orf166		-5.6902	-5.2139	-4.79525	-5.35687
LARP1	-5.21268	-4.44767	-4.39428	-3.35169	-3.66781
PELO	-4.51194	-3.94831	-4.43919	-4.3095	-3.89211
EIF3M	-4.33676	-4.41178	-4.45814	-3.87372	-4.03554
RBX1	-5.28425	-4.86993	-5.51745		-5.44682
AP1G1	-5.0688	-5.35742	-5.50152		-5.22496
SRPK3	-4.18494	-4.3332	-4.19158	-3.73565	-4.73814

TRAFD1	-4.65776	-4.05569	-6.09701	-3.31801	-3.11694
FAF1	-2.89307	-6.4032	-3.65202	-3.2299	-5.08432
MRPS30	-6.23161	-4.6515	-5.9626		-4.49192
SEC31A	-6.20277	-2.68174	-6.57541	-2.74045	-3.16096
MOV10	-5.30182	-5.68855	-5.50666	-4.93964	
NUDT4	-3.35216	-5.64325	-3.71012	-3.47625	-5.37497
GSTM2	-4.19059	-4.6047	-4.29938	-3.66107	-4.8046
GSTM5	-4.19059	-4.6047	-4.29938	-3.66107	-4.8046
PLAA	-6.78559	-3.09967	-4.882	-3.17408	-3.64067
NDUFS6	-2.35716	-6.13723	-2.40031	-6.16306	-4.64045
EPHA1	-4.15138	-5.85843	-3.58684	-4.0743	-4.03002
CASP3	-3.6092	-6.17586	-3.73259	-3.3312	-4.87781
ATP5D	-4.66927	-4.71738	-4.36929	-3.42953	-4.59229
RPL37AP8	-2.38996	-4.98825	-5.08944	-4.16979	-5.14358
ZMYND8		-7.02508	-4.37559	-4.91241	-5.49429
EDC3	-5.48378	-5.75713	-5.68883	-4.90637	
PDIA4	-4.5461	-4.35543	-4.26975	-3.93264	-4.74989
SRFBP1	-4.66159	-4.33369	-4.68749	-4.13513	-4.07435
PTCD3	-5.70367	-6.49356	-4.04004		-5.68621
DDX46	-5.43041	-3.39499	-5.50861	-4.77444	-2.8412
MAGED2	-5.40322	-3.85146	-5.4058	-3.21653	-4.07487
RARA	-3.94285	-5.86028	-3.90118	-4.41347	-3.91848
EIF3B	-4.47637	-4.32124	-5.30535	-3.38505	-4.56041
APIP	-5.66092	-5.75006		-4.36831	-6.28807
ARHGEF2	-4.53546	-2.78455	-5.31077	-5.82438	-3.7023
SNRPC	-5.58285	-5.67795	-5.52619		-5.37456
DDX4	-5.38665	-4.91032	-4.50033	-2.10313	-5.36272
AGAP3	-5.16439	-7.30807	-5.42309	-4.52213	
CS	-5.22042	-4.53609	-5.55184	-2.87752	-4.26252
TRAP1		-5.62501	-6.62576	-4.88721	-5.33105
PCBP2	-4.77925	-4.24451	-4.556	-4.34613	-4.57209
PSMA1	-5.42478	-3.17344	-3.65412	-4.84956	-5.43485
EIF3G	-5.6313	-3.55223	-5.87966	-3.61441	-3.93971
PCYOX1	-1.9126	-5.28724	-4.95108	-5.72388	-4.78503
HARS2	-4.55522	-4.30991	-4.66192	-4.04506	-5.09817
MATR3	-5.40709	-4.71838	-5.56565	-4.42417	-2.56844
SEC61A2	-4.78279	-4.72953	-4.13156	-4.28688	-4.76385
RBM3	-2.81892	-14.1762	-2.84403	-2.85664	
REXO4	-6.15304	-3.13372	-6.20833	-2.7367	-4.49684
RPL13	-5.46717	-2.58319	-4.99943	-4.83607	-4.87123
FOXK2	-3.71763	-7.63066	-4.71455	-3.53532	-3.1841
JAGN1	-1.59523	-5.78354	-5.01074	-4.99844	-5.42537
PHB2	-1.86453	-5.74685	-5.20613	-4.81701	-5.25121
HIST1H1C	-6.91493	-4.9117	-2.40476	-6.08997	-2.60484
SEC61A1	-5.1352	-4.66178	-4.13156	-4.28688	-4.71282
PNN	-2.17213	-5.66261	-4.80563	-4.92362	-5.37639

HMGN4		-5.96091	-5.89779	-2.72288	-4.44234	-3.95113
CAPZA2		-2.67259	-5.48623	-5.16442	-4.60675	-5.21904
SMARCE1		-2.51099	-4.75258	-4.76505	-5.00203	-6.13952
UQCRB		-4.84067	-6.32612		-6.16547	-5.84958
ARPP19		-5.81093	-3.38029	-6.06673	-3.18593	-4.74266
RNMTL1		-5.13713	-4.86181	-4.63101	-4.26204	-4.334
EIF4G2		-5.14859	-3.51698	-5.24274	-4.25063	-5.08561
RPS25		-5.80139	-9.25642			-8.20431
NME1		-4.43498	-5.17076	-5.52859	-3.06628	-5.12523
SUGP2			-5.40913	-7.67616	-5.24679	-5.00569
TJP1		-9.28902	-1.67118	-1.93635	-5.88508	-4.65555
HDGFRP2		-5.26517	-5.70614	-5.47177	-4.50539	-2.60655
GPKOW		-4.74284	-5.34473	-5.16134	-4.29502	-4.04348
RPL30		-5.276	-5.54595	-2.76793	-4.76832	-5.32568
CUL3		-5.01355	-4.38649	-5.16701	-4.38975	-4.7468
UBE2Z		-5.49358	-5.697	-5.29869	-4.94406	-2.27474
CBX1		-9.43251	-4.51135	-3.71741	-3.66025	-2.40244
WARS		-5.15572	-4.75253	-4.37515	-4.6263	-4.86475
SRP68		-3.78651	-3.79622	-8.22924	-8.11074	
THOC5		-4.52908	-4.64561	-5.09969	-4.86277	-4.91247
H1F0		-2.29637	-4.06689	-5.08023	-5.40733	-7.22845
YY2		-2.58352	-5.71604	-5.46823	-4.96666	-5.42228
ATXN2		-5.20544	-4.52649	-5.15133	-4.69733	-4.66636
IMPDH1		-5.02296	-4.54991	-5.06672	-4.47254	-5.21175
NEMF		-4.42756	-5.28942	-5.01646	-4.66339	-4.98257
EIF4A3		-5.44501	-5.60138	-2.98695	-4.8479	-5.51706
TARS		-4.72172	-5.45412	-4.6589	-4.40651	-5.16896
DDX3Y		-4.87728	-5.08661	-4.9927	-4.54167	-4.99445
PICALM		-4.98764	-4.77503	-5.20744	-4.56811	-4.96402
UFM1		-4.89767	-5.19118	-4.76829	-4.40005	-5.2638
NDUFB11		-5.03812	-5.20045	-5.10792	-4.18187	-5.01165
CTBP2		-5.18113	-4.62534	-5.14157	-4.49793	-5.16507
TNPO3		-2.03511	-5.51842	-5.94533	-5.99495	-5.15353
RAD23A		-4.9263	-4.90182	-5.12115	-4.46506	-5.29901
ARPC3		-4.98152	-5.09544	-5.16532	-4.36831	-5.15753
ACSL3		-4.63473	-5.94915	-4.2796	-4.96273	-4.9775
UBE2C		-4.96723	-5.14944	-5.05212	-4.42236	-5.25073
PSMB7		-4.99691	-4.96915	-5.02693	-4.41089	-5.45651
HCFC2		-5.11933	-5.26044	-5.17764	-4.33127	-5.08168
SP100		-4.73354	-5.49745	-4.66319	-4.88202	-5.29697
DPYSL5		-5.00661	-5.27055	-5.10738	-4.44352	-5.27812
UBE2D2		-4.78417	-2.62528	-8.24164	-2.14884	-7.38529
EXOSC4		-5.24928	-4.87591	-5.35449	-4.48563	-5.23036
	40057	-5.05453	-5.46499	-5.06188	-4.50708	-5.14838
LCP1			-6.12033	-6.77008	-5.44633	-6.90431
HGH1		-5.36044	-5.43261	-5.21663	-4.53145	-5.07159

UBL5	-5.24881	-5.33386	-5.3062	-4.69828	-5.3341
SNRPF	-5.4403	-5.73926	-4.31862	-5.14189	-5.33137
TMEM41B		-5.69977	-8.445	-5.62613	-6.30196
TMEM180		-7.20109	-5.82679	-6.75557	-6.37137
ADRM1	-5.33097	-5.44569	-5.4056	-4.69544	-5.391
ARF5	-5.33067	-5.65143	-5.11866	-4.79241	-5.38654
POLRMT	-4.86626	-5.38373	-4.42114	-6.32853	-5.37705
MARS	-5.36	-5.21882	-6.09765	-3.98115	-5.75213
SNRPA	-5.30764	-5.56998	-5.46036	-4.84685	-5.24271
GNB3	-5.49391	-5.3995	-5.87767	-4.58742	-5.11406
GNB4	-5.49391	-5.3995	-5.87767	-4.58742	-5.11406
STAU2	-5.36044	-5.60862	-5.47655	-4.83702	-5.37716
YY1	-5.38705	-5.7204	-5.44456	-5.05237	-5.42446
YARS2		-5.91674	-8.7079	-6.18342	-6.25421
TCEA2	-5.39512	-5.79077	-5.65493	-5.05073	-5.49063
EBP	-5.832	-4.91765	-6.21543	-8.51453	-2.17
PDCD5	-5.4503	-6.92339	-5.15005	-5.4894	-6.41734
HNRNPL	-2.15736	-8.75538	-10.8127	-4.99085	-2.96078
EIF3H	-5.35421	-7.169	-5.42644	-5.96185	-7.1057
