# Supplementary material for: GPR19 Coordinates Multiple Molecular Aspects of Stress Responses Associated with the Aging Process
Source: Int J Mol Sci. 2023 May 9;24(10):8499. doi: 10.3390/ijms24108499 (PMC10218176; doi:10.3390/ijms24108499)
Supplement: Supplementary file 1 [file ijms-24-08499-s001.zip › Data-Supplement-Maudsley-et-al-2023-Table-S5.pdf]

Data Supplement

GPR19 coordinates multiple molecular aspects of stress responses associated with the aging process

Stuart Maudsley [1*], Claudia Schrauwen[1], İrem Harputluoğlu[1], Deborah Walter[1], Hanne Leysen[1], Patricia McDonald[2]

1. Receptor Biology Lab, Department of Biomedical Science, University of Antwerp.
2. H. Lee Moffitt Cancer Center, Tampa, FL, USA

* Corresponding Author: Prof. Stuart Maudsley – stuartmaudsley@uantwerpen.be: receptorbiologylab@gmail.com

Table S5. Gene Ontology term enrichment of the leading-edge 'Cellular Response to Stress'. For each significantly-enriched Gene Ontology (GO) biological process term the degree of enrichment (R) and the negative log10 of the enrichment probability (P) is shown. The associated hybrid score (H) is generated for each GO term via multiplication of R*P.

Gene Ontology Term	Enrichment (R)	$-\log_{10}$ P (P)	Hybrid Score (H)
DNA damage response, signal transduction by p53 class mediator	19.82758621	23.05061	457.0379568
Signal transduction in response to DNA damage	18.75	23.446117	439.6146933
DNA damage checkpoint	17.6056338	23.716699	417.5475136
DNA integrity checkpoint	16.55629139	23.02365	381.1862586
Mitotic cell cycle checkpoint	19.59459459	28.9914	568.0747264
Cell cycle checkpoint	12.90322581	29.402305	379.3845782
S phase of mitotic cell cycle	16.78321678	22.241845	373.2897126
G1/S transition of mitotic cell cycle	13.46153846	23.257275	313.0787002
Chromatin assembly	18.11023622	22.099633	400.2295717
Nucleosome assembly	18.91891892	20.59176	389.5738385
