# Supplementary material for: GPR19 Coordinates Multiple Molecular Aspects of Stress Responses Associated with the Aging Process
Source: Int J Mol Sci. 2023 May 9;24(10):8499. doi: 10.3390/ijms24108499 (PMC10218176; doi:10.3390/ijms24108499)
Supplement: Supplementary file 1 [file ijms-24-08499-s001.zip › Data-Supplement-Maudsley-et-al-2023-Table-S6.pdf]

Data Supplement

GPR19 coordinates multiple molecular aspects of stress responses associated with the aging process

Stuart Maudsley [1*], Claudia Schrauwen[1], İrem Harputluoğlu[1], Deborah Walter[1], Hanne Leysen[1], Patricia McDonald[2]

1. Receptor Biology Lab, Department of Biomedical Science, University of Antwerp.
2. H. Lee Moffitt Cancer Center, Tampa, FL, USA

* Corresponding Author: Prof. Stuart Maudsley – stuartmaudsley@uantwerpen.be: receptorbiologylab@gmail.com

Table S6. MSigDB enrichment analysis of the total mean GPR19 perturbagen response dataset. For each significantly-enriched MSigDB category the nature of the enrichment (enriched or depleted) and the negative log10 of the enrichment probability (P) is shown.

Type	Category Name	$-\log_{10}$ P
enriched	PUJANA BRCA1 PCC NETWORK	109.9878422
enriched	DIAZ CHRONIC MEYLOGENOUS LEUKEMIA UP	96.02042431
enriched	PUJANA CHEK2 PCC NETWORK	74.31972886
enriched	CAIRO HEPATOBLASTOMA CLASSES UP	64.26817119
enriched	BLALOCK ALZHEIMERS DISEASE DN	60.37106675
enriched	PILON KLF1 TARGETS DN	53.25915275
enriched	KRIGE RESPONSE TO TOSEDOSTAT 24HR DN	53.01761342
enriched	LEE BMP2 TARGETS DN	48.58381899
enriched	DANG BOUND BY MYC	48.01514653
enriched	HSIAO HOUSEKEEPING GENES	47.20091321
depleted	DODD NASOPHARYNGEAL CARCINOMA UP	-8.594991917
depleted	FLORIO NEOCORTEX BASAL RADIAL GLIA UP	-11.48977278
depleted	PEREZ TP53 TARGETS	-11.69433914
depleted	MEISSNER BRAIN HCP WITH H3K4ME3 AND H3K27ME3	-12.26247182
depleted	MIKKELSEN MEF HCP WITH H3K27ME3	-13.44346111
depleted	BENPORATH PRC2 TARGETS	-13.58275091
depleted	BENPORATH SUZ12 TARGETS	-15.18727042
depleted	MARTENS TRETINOIN RESPONSE UP	-18.46239622
depleted	BENPORATH ES WITH H3K27ME3	-20.31364173
depleted	ZWANG TRANSIENTLY UP BY 2ND EGF PULSE ONLY	-20.94223503
