# Supplementary material for: GPR19 Coordinates Multiple Molecular Aspects of Stress Responses Associated with the Aging Process
Source: Int J Mol Sci. 2023 May 9;24(10):8499. doi: 10.3390/ijms24108499 (PMC10218176; doi:10.3390/ijms24108499)
Supplement: Supplementary file 1 [file ijms-24-08499-s001.zip › Data-Supplement-Maudsley-et-al-2023-Table-S7.pdf]

Data Supplement

GPR19 coordinates multiple molecular aspects of stress responses associated with the aging process

Stuart Maudsley [1]*, Claudia Schrauwen[1], İrem Harputluoğlu[1], Deborah Walter[1], Hanne Leysen[1], Patricia McDonald[2]

1. Receptor Biology Lab, Department of Biomedical Science, University of Antwerp.
2. H. Lee Moffitt Cancer Center, Tampa, FL, USA

* Corresponding Author: Prof. Stuart Maudsley – stuartmaudsley@uantwerpen.be: receptorbiologylab@gmail.com

Table S7. De novo generated unbiased energy metabolism protein network. Using multiple applications: GLAD4U, GeneShot, and PubPular, with the following input interrogator terms: 'energy metabolism', 'oxidative phosphorylation', and 'glucose metabolism' – a significantly-associated protein list was assembled.

Gene Symbol	Description
INS	Insulin; Insulin decreases blood glucose concentration. It increases cell permeability to monosaccharides, amino acids and fatty acids. It accelerates glycolysis, the pentose phosphate cycle, and glycogen synthesis in liver
AKT1	RAC-alpha serine/threonine-protein kinase; AKT1 is one of 3 closely related serine/threonine- protein kinases (AKT1, AKT2 and AKT3) called the AKT kinase, and which regulate many processes including metabolism, proliferation, cell survival, growth and angiogenesis. This is mediated through serine and/or threonine phosphorylation of a range of downstream substrates. Over 100 substrate candidates have been reported so far, but for most of them, no isoform specificity has been reported. AKT is responsible of the regulation of glucose uptake by mediating insulin-induced translocation of th [...]
LEP	Leptin; Key player in the regulation of energy balance and body weight control. Once released into the circulation, has central and peripheral effects by binding LEPR, found in many tissues, which results in the activation of several major signaling pathways. In the hypothalamus, acts as an appetite-regulating factor that induces a decrease in food intake and an increase in energy consumption by inducing anorexinogenic factors and suppressing orexigenic neuropeptides, also regulates bone mass and secretion of hypothalamo-pituitary-adrenal hormones. In the periphery, increases basal met [...]
HIF1A	Hypoxia-inducible factor 1-alpha; Functions as a master transcriptional regulator of the adaptive response to hypoxia. Under hypoxic conditions, activates the transcription of over 40 genes, including erythropoietin, glucose transporters, glycolytic enzymes, vascular endothelial growth factor, HILPDA, and other genes whose protein products increase oxygen delivery or facilitate metabolic adaptation to hypoxia. Plays an essential role in embryonic vascularization, tumor angiogenesis and pathophysiology of ischemic disease. Binds to core DNA sequence 5'-[AG]CGTG-3' within the hypoxia res [...]
PPARA	Peroxisome proliferator-activated receptor alpha; Ligand-activated transcription factor. Key regulator of lipid metabolism. Activated by the endogenous ligand 1-palmitoyl- 2-oleoyl-sn-glycerol-3-phosphocholine (16:0/18:1-GPC). Activated by oleylethanolamide, a naturally occurring lipid that regulates satiety. Receptor for peroxisome proliferators such as hypolipidemic drugs and fatty acids. Regulates the peroxisomal beta-oxidation pathway of fatty acids. Functions as transcription activator for the ACOX1 and P450 genes. Transactivation activity requires heterodimerization with RXRA and [...]
CASP3	Caspase-3; Involved in the activation cascade of caspases responsible for apoptosis execution. At the onset of apoptosis it proteolytically cleaves poly(ADP-ribose) polymerase (PARP) at a '216-Asp-|-Gly-217' bond. Cleaves and activates sterol regulatory element binding proteins (SREBPs) between the basic helix-loop- helix leucine zipper domain and the membrane attachment domain. Cleaves and activates caspase-6, -7 and -9. Involved in the cleavage of huntingtin. Triggers cell adhesion in sympathetic neurons through RET cleavage
PPARG	Peroxisome proliferator-activated receptor gamma; Nuclear receptor that binds peroxisome proliferators such as hypolipidemic drugs and fatty acids. Once activated by a ligand, the nuclear receptor binds to DNA specific PPAR response elements (PPRE) and modulates the transcription of its target genes, such as acyl-CoA oxidase. It therefore controls the peroxisomal beta-oxidation pathway of fatty acids. Key regulator of adipocyte differentiation and glucose homeostasis. ARF6 acts as a key regulator of the tissue-specific adipocyte P2 (aP2) enhancer. Acts as a critical regulator of gut ho [...]
SLC2A1	Solute carrier family 2, facilitated glucose transporter member 1; Facilitative glucose transporter. This isoform may be responsible for constitutive or basal glucose uptake. Has a very broad substrate specificity; can transport a wide range of aldoses including both pentoses and hexoses; Belongs to the major facilitator superfamily. Sugar transporter (TC 2.A.1.1) family. Glucose transporter subfamily
TNF	Tumor necrosis factor; Cytokine that binds to TNFRSF1A/TNFR1 and TNFRSF1B/TNFBR. It is mainly secreted by macrophages and can induce cell death of certain tumor cell lines. It is potent pyrogen causing fever by direct action or by stimulation of interleukin-1 secretion and is implicated in the induction of cachexia, Under certain conditions it can stimulate cell proliferation and induce cell differentiation. Impairs regulatory T-cells (Treg) function in individuals with rheumatoid arthritis via FOXP3 dephosphorylation. Upregulates the expression of protein phosphatase 1 (PP1), which de [...]
UCP1	Mitochondrial brown fat uncoupling protein 1; Mitochondrial protein responsible for thermogenic respiration, a specialized capacity of brown adipose tissue and beige fat that participates to non-shivering adaptive thermogenesis to temperature and diet variations and more generally to the regulation of energy balance (By similarity). Functions as a long-chain fatty acid/LCFA and proton symporter, simultaneously transporting one LCFA and one proton through the inner mitochondrial membrane. However, LCFAs remaining associated with the transporter via their hydrophobic tails, it results in [...]
ADIPOQ	Adiponectin, c1q and collagen domain containing; Adiponectin; Important adipokine involved in the control of fat metabolism and insulin sensitivity, with direct anti-diabetic, anti-atherogenic and anti-inflammatory activities. Stimulates AMPK phosphorylation and activation in the liver and the skeletal muscle, enhancing glucose utilization and fatty-acid combustion. Antagonizes TNF-alpha by negatively regulating its expression in various tissues such as liver and macrophages, and also by counteracting its effects. Inhibits endothelial NF-kappa-B signaling through a cAMP-dependent pathw [...]
GPX4	Phospholipid hydroperoxide glutathione peroxidase, mitochondrial; Protects cells against membrane lipid peroxidation and cell death. Required for normal sperm development and male fertility. Could play a major role in protecting mammals from the toxicity of ingested lipid hydroperoxides. Essential for embryonic development. Protects from radiation and oxidative damage. Essential for maturation and survival of photoreceptor cells. Plays a role in a primary T cell response to viral and parasitic infection by protecting T cells from ferroptosis, a cell death resulting from an iron-depende [...]
HRAS	GTPase HRas; Involved in the activation of Ras protein signal transduction. Ras proteins bind GDP/GTP and possess intrinsic GTPase activity; Belongs to the small GTPase superfamily. Ras family

IL1B	Interleukin-1 beta; Potent proinflammatory cytokine. Initially discovered as the major endogenous pyrogen, induces prostaglandin synthesis, neutrophil influx and activation, T-cell activation and cytokine production, B-cell activation and antibody production, and fibroblast proliferation and collagen production. Promotes Th17 differentiation of T-cells
IL6	Interleukin-6; Cytokine with a wide variety of biological functions. It is a potent inducer of the acute phase response. Plays an essential role in the final differentiation of B-cells into Ig- secreting cells Involved in lymphocyte and monocyte differentiation. Acts on B-cells, T-cells, hepatocytes, hematopoietic progenitor cells and cells of the CNS. Required for the generation of T(H)17 cells. Also acts as a myokine. It is discharged into the bloodstream after muscle contraction and acts to increase the breakdown of fats and to improve insulin resistance. It induces myeloma and plas [...]
POMC	Pro-opiomelanocortin; Met-enkephalin: Endogenous opiate; Belongs to the POMC family
SIRT1	NAD-dependent protein deacetylase sirtuin-1; NAD-dependent protein deacetylase that links transcriptional regulation directly to intracellular energetics and participates in the coordination of several separated cellular functions such as cell cycle, response to DNA damage, metobolism, apoptosis and autophagy. Can modulate chromatin function through deacetylation of histones and can promote alterations in the methylation of histones and DNA, leading to transcriptional repression. Deacetylates a broad range of transcription factors and coregulators, thereby regulating target gene expres [...]
MYC	Myc proto-oncogene protein; Transcription factor that binds DNA in a non-specific manner, yet also specifically recognizes the core sequence 5'- CAC[GA]TG-3'. Activates the transcription of growth-related genes. Binds to the VEGFA promoter, promoting VEGFA production and subsequent sprouting angiogenesis; Basic helix-loop-helix proteins
FN1	Fibronectin 1; Fibronectin type III domain containing; Endogenous ligands
FNDC5	Fibronectin type III domain-containing protein 5; Irisin: Contrary to mouse, may not be involved in the beneficial effects of muscular exercise, nor in the induction of browning of human white adipose tissue; Fibronectin type III domain containing
GCG	Glucagon; Glicentin may modulate gastric acid secretion and the gastro-pyloro-duodenal activity. May play an important role in intestinal mucosal growth in the early period of life; Belongs to the glucagon family
GPT	Glutamic--pyruvic transaminase; Alanine aminotransferase 1; Catalyzes the reversible transamination between alanine and 2-oxoglutarate to form pyruvate and glutamate. Participates in cellular nitrogen metabolism and also in liver gluconeogenesis starting with precursors transported from skeletal muscles (By similarity); Belongs to the class-I pyridoxal-phosphate-dependent aminotransferase family. Alanine aminotransferase subfamily
PKM	Pyruvate kinase m1/2; Pyruvate kinase PKM; Glycolytic enzyme that catalyzes the transfer of a phosphoryl group from phosphoenolpyruvate (PEP) to ADP, generating ATP. Stimulates POU5F1-mediated transcriptional activation. Plays a general role in caspase independent cell death of tumor cells. The ratio between the highly active tetrameric form and nearly inactive dimeric form determines whether glucose carbons are channeled to biosynthetic processes or used for glycolytic ATP production. The transition between the 2 forms contributes to the control of glycolysis and is important for tumo [...]
PPARGC1A	Peroxisome proliferator-activated receptor gamma coactivator 1-alpha; Transcriptional coactivator for steroid receptors and nuclear receptors. Greatly increases the transcriptional activity of PPARG and thyroid hormone receptor on the uncoupling protein promoter. Can regulate key mitochondrial genes that contribute to the program of adaptive thermogenesis. Plays an essential role in metabolic reprogramming in response to dietary availability through coordination of the expression of a wide array of genes involved in glucose and fatty acid metabolism. Induces the expression of PERM1 in [...]
SIRT3	NAD-dependent protein deacetylase sirtuin-3, mitochondrial; NAD-dependent protein deacetylase. Activates or deactivates mitochondrial target proteins by deacetylating key lysine residues. Known targets include ACSS1, IDH, GDH, SOD2, PDHA1, LCAD, SDHA and the ATP synthase subunit ATP5O. Contributes to the regulation of the cellular energy metabolism. Important for regulating tissue-specific ATP levels; Sirtuins
ACE	Angiotensin-converting enzyme; Converts angiotensin I to angiotensin II by release of the terminal His-Leu, this results in an increase of the vasoconstrictor activity of angiotensin. Also able to inactivate bradykinin, a potent vasodilator. Has also a glycosidase activity which releases GPI-anchored proteins from the membrane by cleaving the mannose linkage in the GPI moiety; CD molecules
ACE2	Angiotensin-converting enzyme 2; Carboxypeptidase which converts angiotensin I to angiotensin 1-9, a peptide of unknown function, and angiotensin II to angiotensin 1-7, a vasodilator. Also able to hydrolyze apelin- 13 and dynorphin-13 with high efficiency. May be an important regulator of heart function
AGER	Advanced glycosylation end product-specific receptor; Mediates interactions of advanced glycosylation end products (AGE). These are nonenzymatically glycosylated proteins which accumulate in vascular tissue in aging and at an accelerated rate in diabetes. Acts as a mediator of both acute and chronic vascular inflammation in conditions such as atherosclerosis and in particular as a complication of diabetes. AGE/RAGE signaling plays an important role in regulating the production/expression of TNF- alpha, oxidative stress, and endothelial dysfunction in type 2 diabetes. Interaction with S [...]
AGRP	Agouti-related protein; Plays a role in weight homeostasis. Involved in the control of feeding behavior through the central melanocortin system. Acts as alpha melanocyte-stimulating hormone antagonist by inhibiting cAMP production mediated by stimulation of melanocortin receptors within the hypothalamus and adrenal gland. Has very low activity with MC5R (By similarity). Is an inverse agonist for MC3R and MC4R being able to suppress their constitutive activity. It promotes MC3R and MC4R endocytosis in an arrestin-dependent manner; Endogenous ligands
ALB	Serum albumin; Serum albumin, the main protein of plasma, has a good binding capacity for water, Ca(2+), Na(+), K(+), fatty acids, hormones, bilirubin and drugs. Its main function is the regulation of the colloidal osmotic pressure of blood. Major zinc transporter in plasma, typically binds about 80% of all plasma zinc; Belongs to the ALB/AFP/VDB family
APP	Amyloid-beta A4 protein; N-APP binds TNFRSF21 triggering caspase activation and degeneration of both neuronal cell bodies (via caspase-3) and axons (via caspase-6); Endogenous ligands
BCL2	Apoptosis regulator Bcl-2; Suppresses apoptosis in a variety of cell systems including factor-dependent lymphohematopoietic and neural cells. Regulates cell death by controlling the mitochondrial membrane permeability. Appears to function in a feedback loop system with caspases. Inhibits caspase activity either by preventing the release of cytochrome c from the mitochondria and/or by binding to the apoptosis-activating factor (APAF-1). May attenuate inflammation by impairing NLRP1-inflammasome activation, hence CASP1 activation and IL1B release; BCL2 family
BDNF	Brain-derived neurotrophic factor; During development, promotes the survival and differentiation of selected neuronal populations of the peripheral and central nervous systems. Participates in axonal growth, pathfinding and in the modulation of dendritic growth and morphology. Major regulator of synaptic transmission and plasticity at adult synapses in many regions of the CNS. The versatility

	of BDNF is emphasized by its contribution to a range of adaptive neuronal responses including long-term potentiation (LTP), long-term depression (LTD), certain forms of short-term synaptic plastic [...]
CD4	T-cell surface glycoprotein CD4; Integral membrane glycoprotein that plays an essential role in the immune response and serves multiple functions in responses against both external and internal offenses. In T-cells, functions primarily as a coreceptor for MHC class II molecule:peptide complex. The antigens presented by class II peptides are derived from extracellular proteins while class I peptides are derived from cytosolic proteins. Interacts simultaneously with the T-cell receptor (TCR) and the MHC class II presented by antigen presenting cells (APCs). In turn, recruits the Src kina [...]
CDH1	Cadherin-1; Cadherins are calcium-dependent cell adhesion proteins. They preferentially interact with themselves in a homophilic manner in connecting cells; cadherins may thus contribute to the sorting of heterogeneous cell types. CDH1 is involved in mechanisms regulating cell-cell adhesions, mobility and proliferation of epithelial cells. Has a potent invasive suppressor role. It is a ligand for integrin alpha-E/beta-7
CDH2	Cadherin-2; Cadherins are calcium-dependent cell adhesion proteins. They preferentially interact with themselves in a homophilic manner in connecting cells; cadherins may thus contribute to the sorting of heterogeneous cell types. Acts as a regulator of neural stem cells quiescence by mediating anchorage of neural stem cells to ependymocytes in the adult subependymal zone: upon cleavage by MMP24, CDH2-mediated anchorage is affected, leading to modulate neural stem cell quiescence. CDH2 may be involved in neuronal recognition mechanism. In hippocampal neurons, may regulate dendritic spi [...]
CYBB	Cytochrome b-245 heavy chain; Critical component of the membrane-bound oxidase of phagocytes that generates superoxide. It is the terminal component of a respiratory chain that transfers single electrons from cytoplasmic NADPH across the plasma membrane to molecular oxygen on the exterior. Also functions as a voltage-gated proton channel that mediates the H(+) currents of resting phagocytes. It participates in the regulation of cellular pH and is blocked by zinc
CYCS	Cytochrome c, somatic; Cytochrome c; Electron carrier protein. The oxidized form of the cytochrome c heme group can accept an electron from the heme group of the cytochrome c1 subunit of cytochrome reductase. Cytochrome c then transfers this electron to the cytochrome oxidase complex, the final protein carrier in the mitochondrial electron-transport chain
ENSP00000387760	Angiotensin-converting enzyme; Uncharacterized protein
FOS	Proto-oncogene c-Fos; Nuclear phosphoprotein which forms a tight but non- covalently linked complex with the JUN/AP-1 transcription factor. In the heterodimer, FOS and JUN/AP-1 basic regions each seems to interact with symmetrical DNA half sites. On TGF-beta activation, forms a multimeric SMAD3/SMAD4/JUN/FOS complex at the AP1/SMAD- binding site to regulate TGF-beta-mediated signaling. Has a critical function in regulating the development of cells destined to form and maintain the skeleton. It is thought to have an important role in signal transduction, cell proliferation and different [...]
GIPR	Gastric inhibitory polypeptide receptor; This is a receptor for GIP. The activity of this receptor is mediated by G proteins which activate adenylyl cyclase; Belongs to the G-protein coupled receptor 2 family
GLP1R	Glucagon-like peptide 1 receptor; G-protein coupled receptor for glucagon-like peptide 1 (GLP-1). Ligand binding triggers activation of a signaling cascade that leads to the activation of adenylyl cyclase and increased intracellular cAMP levels. Plays a role in regulating insulin secretion in response to GLP-1 (By similarity)
HSPA4	Heat shock protein family A member 4; Belongs to the heat shock protein 70 family
IDH2	Isocitrate dehydrogenase [NADP], mitochondrial; Plays a role in intermediary metabolism and energy production. It may tightly associate or interact with the pyruvate dehydrogenase complex; Belongs to the isocitrate and isopropylmalate dehydrogenases family
IGF1	Insulin-like growth factor I; The insulin-like growth factors, isolated from plasma, are structurally and functionally related to insulin but have a much higher growth-promoting activity. May be a physiological regulator of [1-14C]-2-deoxy-D-glucose (2DG) transport and glycogen synthesis in osteoblasts. Stimulates glucose transport in bone-derived osteoblastic (PyMS) cells and is effective at much lower concentrations than insulin, not only regarding glycogen and DNA synthesis but also with regard to enhancing glucose uptake. May play a role in synapse maturation. Ca(2+)-dependent exoc [...]
IGFBP1	Insulin-like growth factor-binding protein 1; IGF-binding proteins prolong the half-life of the IGFs and have been shown to either inhibit or stimulate the growth promoting effects of the IGFs on cell culture. They alter the interaction of IGFs with their cell surface receptors. Promotes cell migration
IL10	Interleukin-10; Inhibits the synthesis of a number of cytokines, including IFN-gamma, IL-2, IL-3, TNF and GM-CSF produced by activated macrophages and by helper T-cells; Belongs to the IL-10 family
JUN	Transcription factor AP-1; Transcription factor that recognizes and binds to the enhancer heptamer motif 5'-TGA[CG]TCA-3'. Promotes activity of NR5A1 when phosphorylated by HIPK3 leading to increased steroidogenic gene expression upon cAMP signaling pathway stimulation. Involved in activated KRAS-mediated transcriptional activation of USP28 in colorectal cancer (CRC) cells. Binds to the USP28 promoter in colorectal cancer (CRC) cells; Basic leucine zipper proteins
KRAS	GTPase KRas; Ras proteins bind GDP/GTP and possess intrinsic GTPase activity. Plays an important role in the regulation of cell proliferation. Plays a role in promoting oncogenic events by inducing transcriptional silencing of tumor suppressor genes (TSGs) in colorectal cancer (CRC) cells in a ZNF304-dependent manner; Belongs to the small GTPase superfamily. Ras family
LDHA	L-lactate dehydrogenase a chain; Lactate dehydrogenase A; Belongs to the LDH/MDH superfamily. LDH family
MB	Myoglobin; Serves as a reserve supply of oxygen and facilitates the movement of oxygen within muscles
MCU	Calcium uniporter protein, mitochondrial; Mitochondrial inner membrane calcium uniporter that mediates calcium uptake into mitochondria. Constitutes the pore-forming and calcium-conducting subunit of the uniporter complex (uniplex). Activity is regulated by MICU1 and MICU2. At low Ca(2+) levels MCU activity is down- regulated by MICU1 and MICU2; at higher Ca(2+) levels MICU1 increases MCU activity. Mitochondrial calcium homeostasis plays key roles in cellular physiology and regulates cell bioenergetics, cytoplasmic calcium signals and activation of cell death pathways. Involved in buff [...]
MFN2	Mitofusin-2; Mitochondrial outer membrane GTPase that mediates mitochondrial clustering and fusion. Mitochondria are highly dynamic organelles, and their morphology is determined by the equilibrium between mitochondrial fusion and fission events. Overexpression induces the formation of mitochondrial networks. Membrane clustering requires GTPase activity and may involve a major rearrangement of the coiled coil domains (Probable). Plays a central role in mitochondrial metabolism and may be associated with obesity and/or apoptosis processes (By similarity). Plays an important role in the [...]

NDUFB8	NADH dehydrogenase [ubiquinone] 1 beta subcomplex subunit 8, mitochondrial; Accessory subunit of the mitochondrial membrane respiratory chain NADH dehydrogenase (Complex I), that is believed not to be involved in catalysis. Complex I functions in the transfer of electrons from NADH to the respiratory chain. The immediate electron acceptor for the enzyme is believed to be ubiquinone
NPY	Pro-neuropeptide Y; NPY is implicated in the control of feeding and in secretion of gonadotrophin-release hormone; Belongs to the NPY family
NRAS	Nras proto-oncogene, gtpase; GTPase NRas; Ras proteins bind GDP/GTP and possess intrinsic GTPase activity
PNPLA2	Patatin-like phospholipase domain-containing protein 2; Catalyzes the initial step in triglyceride hydrolysis in adipocyte and non-adipocyte lipid droplets. Also has acylglycerol transacylase activity. May act coordinately with LIPE/HLS within the lipolytic cascade. Regulates adiposome size and may be involved in the degradation of adiposomes. May play an important role in energy homeostasis. May play a role in the response of the organism to starvation, enhancing hydrolysis of triglycerides and providing free fatty acids to other tissues to be oxidized in situations of energy depletio [...]
PPIG	Peptidyl-prolyl cis-trans isomerase G; PPIases accelerate the folding of proteins. It catalyzes the cis-trans isomerization of proline imidic peptide bonds in oligopeptides. May be implicated in the folding, transport, and assembly of proteins. May play an important role in the regulation of pre-mRNA splicing; Cyclophilin peptidylprolyl isomerases
PRDM16	PR domain zinc finger protein 16; Binds DNA and functions as a transcriptional regulator. Functions in the differentiation of brown adipose tissue (BAT) which is specialized in dissipating chemical energy in the form of heat in response to cold or excess feeding while white adipose tissue (WAT) is specialized in the storage of excess energy and the control of systemic metabolism. Together with CEBPB, regulates the differentiation of myoblastic precursors into brown adipose cells. Functions also as a repressor of TGF-beta signaling. Isoform 4 may regulate granulocytes differentiation; L [...]
SCD	Stearoyl-coa desaturase (delta-9 desaturase); Acyl-CoA desaturase; Stearyl-CoA desaturase that utilizes O(2) and electrons from reduced cytochrome b5 to introduce the first double bond into saturated fatty acyl-CoA substrates. Catalyzes the insertion of a cis double bond at the delta-9 position into fatty acyl-CoA substrates including palmitoyl-CoA and stearoyl-CoA. Gives rise to a mixture of 16:1 and 18:1 unsaturated fatty acids. Plays an important role in lipid biosynthesis. Plays an important role in regulating the expression of genes that are involved in lipogenesis and in regulati [...]
SIRT2	NAD-dependent protein deacetylase sirtuin-2; NAD-dependent protein deacetylase, which deacetylates internal lysines on histone and alpha-tubulin as well as many other proteins such as key transcription factors. Participates in the modulation of multiple and diverse biological processes such as cell cycle control, genomic integrity, microtubule dynamics, cell differentiation, metabolic networks, and autophagy. Plays a major role in the control of cell cycle progression and genomic stability. Functions in the antephase checkpoint preventing precocious mitotic entry in response to microtu [...]
SIRT4	NAD-dependent protein lipoamidase sirtuin-4, mitochondrial; Acts as NAD-dependent protein lipoamidase, ADP-ribosyl transferase and deacetylase. Catalyzes more efficiently removal of lipoyl- and biotinyl- than acetyl-lysine modifications. Inhibits the pyruvate dehydrogenase complex (PDH) activity via the enzymatic hydrolysis of the lipoamide cofactor from the E2 component, DLAT, in a phosphorylation-independent manner. Catalyzes the transfer of ADP-ribosyl groups onto target proteins, including mitochondrial GLUD1, inhibiting GLUD1 enzyme activity. Acts as a negative regulator of mitoch [...]
SIRT5	NAD-dependent protein deacylase sirtuin-5, mitochondrial; NAD-dependent lysine demalonylase, desuccinylase and deglutarylase that specifically removes malonyl, succinyl and glutaryl groups on target proteins. Activates CPS1 and contributes to the regulation of blood ammonia levels during prolonged fasting: acts by mediating desuccinylation and deglutarylation of CPS1, thereby increasing CPS1 activity in response to elevated NAD levels during fasting. Activates SOD1 by mediating its desuccinylation, leading to reduced reactive oxygen species. Modulates ketogenesis through the desuccinyl [...]
SIRT6	NAD-dependent protein deacetylase sirtuin-6; NAD-dependent protein deacetylase. Has deacetylase activity towards histone H3K9Ac and H3K56Ac. Modulates acetylation of histone H3 in telomeric chromatin during the S-phase of the cell cycle. Deacetylates histone H3K9Ac at NF-kappa-B target promoters and may down-regulate the expression of a subset of NF- kappa-B target genes. Acts as a corepressor of the transcription factor HIF1A to control the expression of multiple glycolytic genes to regulate glucose homeostasis. Required for genomic stability. Regulates the production of TNF protein. [...]
SIRT7	NAD-dependent protein deacetylase sirtuin-7; NAD-dependent protein deacetylase that specifically mediates deacetylation of histone H3 at 'Lys-18' (H3K18Ac). In contrast to other histone deacetylases, displays selectivity for a single histone mark, H3K18Ac, directly linked to control of gene expression. H3K18Ac is mainly present around the transcription start site of genes and has been linked to activation of nuclear hormone receptors. SIRT7 thereby acts as a transcription repressor. Moreover, H3K18 hypoacetylation has been reported as a marker of malignancy in various cancers and seems [...]
SLC2A14	Solute carrier family 2, facilitated glucose transporter member 14; Facilitative glucose transporter (By similarity). May have a specific function related to spermatogenesis; Solute carriers
SLC2A3	Solute carrier family 2, facilitated glucose transporter member 3; Facilitative glucose transporter that can also mediate the uptake of various other monosaccharides across the cell membrane. Mediates the uptake of glucose, 2-deoxyglucose, galactose, mannose, xylose and fucose, and probably also dehydroascorbate. Does not mediate fructose transport; Belongs to the major facilitator superfamily. Sugar transporter (TC 2.A.1.1) family. Glucose transporter subfamily
STAT3	Signal transducer and activator of transcription 3; Signal transducer and transcription activator that mediates cellular responses to interleukins, KITLG/SCF, LEP and other growth factors. Once activated, recruits coactivators, such as NCOA1 or MED1, to the promoter region of the target gene. May mediate cellular responses to activated FGFR1, FGFR2, FGFR3 and FGFR4. Binds to the interleukin-6 (IL-6)-responsive elements identified in the promoters of various acute-phase protein genes. Activated by IL31 through IL31RA. Acts as a regulator of inflammatory response by regulating differenti [...]
STK11	Serine/threonine-protein kinase STK11; Tumor suppressor serine/threonine-protein kinase that controls the activity of AMP-activated protein kinase (AMPK) family members, thereby playing a role in various processes such as cell metabolism, cell polarity, apoptosis and DNA damage response. Acts by phosphorylating the T-loop of AMPK family proteins, thus promoting their activity: phosphorylates PRKAA1, PRKAA2, BRSK1, BRSK2, MARK1, MARK2, MARK3, MARK4, NUAK1, NUAK2, SIK1, SIK2, SIK3 and SNRK but not MELK. Also phosphorylates non- AMPK family proteins such as STRADA, PTEN and possibly p53/T [...]
TFAM	Transcription factor A, mitochondrial; Binds to the mitochondrial light strand promoter and functions in mitochondrial transcription regulation. Required for accurate and efficient promoter recognition by the mitochondrial RNA polymerase. Promotes transcription initiation from the HSP1 and the light strand promoter by binding immediately upstream of transcriptional start sites. Is able to

	unwind DNA. Bends the mitochondrial light strand promoter DNA into a U-turn shape via its HMG boxes. Required for maintenance of normal levels of mitochondrial DNA. May play a role in organizing and c [...]
TP53	Cellular tumor antigen p53; Acts as a tumor suppressor in many tumor types; induces growth arrest or apoptosis depending on the physiological circumstances and cell type. Involved in cell cycle regulation as a trans-activator that acts to negatively regulate cell division by controlling a set of genes required for this process. One of the activated genes is an inhibitor of cyclin-dependent kinases. Apoptosis induction seems to be mediated either by stimulation of BAX and FAS antigen expression, or by repression of Bcl-2 expression. In cooperation with mitochondrial PPIF is involved in [...]
TYMS	Thymidylate synthetase; Thymidylate synthase; Contributes to the de novo mitochondrial thymidylate biosynthesis pathway
VCAM1	Vascular cell adhesion protein 1; Important in cell-cell recognition. Appears to function in leukocyte-endothelial cell adhesion. Interacts with integrin alpha-4/beta-1 (ITGA4/ITGB1) on leukocytes, and mediates both adhesion and signal transduction. The VCAM1/ITGA4/ITGB1 interaction may play a pathophysiologic role both in immune responses and in leukocyte emigration to sites of inflammation; C2-set domain containing
HEATR5B	HEAT repeat-containing protein 5B; Armadillo-like helical domain containing
HMOX1	Heme oxygenase 1; Heme oxygenase cleaves the heme ring at the alpha methene bridge to form biliverdin. Biliverdin is subsequently converted to bilirubin by biliverdin reductase. Under physiological conditions, the activity of heme oxygenase is highest in the spleen, where senescent erythrocytes are sequestrated and destroyed. Exhibits cytoprotective effects since excess of free heme sensitizes cells to undergo apoptosis
GAPDH	Glyceraldehyde-3-phosphate dehydrogenase; Has both glyceraldehyde-3-phosphate dehydrogenase and nitrosylase activities, thereby playing a role in glycolysis and nuclear functions, respectively. Participates in nuclear events including transcription, RNA transport, DNA replication and apoptosis. Nuclear functions are probably due to the nitrosylase activity that mediates cysteine S-nitrosylation of nuclear target proteins such as SIRT1, HDAC2 and PRKDC. Modulates the organization and assembly of the cytoskeleton. Facilitates the CHP1-dependent microtubule and membrane associations throu [...]
ACTB	Actin, cytoplasmic 1; Actins are highly conserved proteins that are involved in various types of cell motility and are ubiquitously expressed in all eukaryotic cells
NFE2L2	Nuclear factor erythroid 2-related factor 2; Transcription activator that binds to antioxidant response (ARE) elements in the promoter regions of target genes. Important for the coordinated up-regulation of genes in response to oxidative stress. May be involved in the transcriptional activation of genes of the beta-globin cluster by mediating enhancer activity of hypersensitive site 2 of the beta-globin locus control region; Basic leucine zipper proteins
MAPK3	Mitogen-activated protein kinase 3; Serine/threonine kinase which acts as an essential component of the MAP kinase signal transduction pathway. MAPK1/ERK2 and MAPK3/ERK1 are the 2 MAPKs which play an important role in the MAPK/ERK cascade. They participate also in a signaling cascade initiated by activated KIT and KITLG/SCF. Depending on the cellular context, the MAPK/ERK cascade mediates diverse biological functions such as cell growth, adhesion, survival and differentiation through the regulation of transcription, translation, cytoskeletal rearrangements. The MAPK/ERK cascade plays a [...]
MTOR	Serine/threonine-protein kinase mTOR; Serine/threonine protein kinase which is a central regulator of cellular metabolism, growth and survival in response to hormones, growth factors, nutrients, energy and stress signals. MTOR directly or indirectly regulates the phosphorylation of at least 800 proteins. Functions as part of 2 structurally and functionally distinct signaling complexes mTORC1 and mTORC2 (mTOR complex 1 and 2). Activated mTORC1 up-regulates protein synthesis by phosphorylating key regulators of mRNA translation and ribosome synthesis. This includes phosphorylation of EIF [...]
KEAP1	Kelch-like ECH-associated protein 1; Acts as a substrate adapter protein for the E3 ubiquitin ligase complex formed by CUL3 and RBX1 and targets NFE2L2/NRF2 for ubiquitination and degradation by the proteasome, thus resulting in the suppression of its transcriptional activity and the repression of antioxidant response element-mediated detoxifying enzyme gene expression. Retains NFE2L2/NRF2 and may also retain BPTF in the cytosol. Targets PGAM5 for ubiquitination and degradation by the proteasome; BTB domain containing
ANXA5	Annexin A5; This protein is an anticoagulant protein that acts as an indirect inhibitor of the thromboplastin-specific complex, which is involved in the blood coagulation cascade; Annexins
NFKBIA	NF-kappa-B inhibitor alpha; Inhibits the activity of dimeric NF-kappa-B/REL complexes by trapping REL dimers in the cytoplasm through masking of their nuclear localization signals. On cellular stimulation by immune and proinflammatory responses, becomes phosphorylated promoting ubiquitination and degradation, enabling the dimeric RELA to translocate to the nucleus and activate transcription
TLR4	Toll-like receptor 4; Cooperates with LY96 and CD14 to mediate the innate immune response to bacterial lipopolysaccharide (LPS). Acts via MYD88, TIRAP and TRAF6, leading to NF-kappa-B activation, cytokine secretion and the inflammatory response. Also involved in LPS-independent inflammatory responses triggered by free fatty acids, such as palmitate, and Ni(2+). Responses triggered by Ni(2+) require non-conserved histidines and are, therefore, species-specific. Both M.tuberculosis HSP70 (dnaK) and HSP65 (groEL-2) act via this protein to stimulate NF-kappa-B expression. In complex with T [...]
NQO1	NAD(P)H dehydrogenase [quinone] 1; The enzyme apparently serves as a quinone reductase in connection with conjugation reactions of hydroquinons involved in detoxification pathways as well as in biosynthetic processes such as the vitamin K-dependent gamma-carboxylation of glutamate residues in prothrombin synthesis; Belongs to the NAD(P)H dehydrogenase (quinone) family
CXCL8	Interleukin-8; IL-8 is a chemotactic factor that attracts neutrophils, basophils, and T-cells, but not monocytes. It is also involved in neutrophil activation. It is released from several cell types in response to an inflammatory stimulus. IL-8(6-77) has a 5-10-fold higher activity on neutrophil activation, IL-8(5-77) has increased activity on neutrophil activation and IL-8(7-77) has a higher affinity to receptors CXCR1 and CXCR2 as compared to IL-8(1-77), respectively; Chemokine ligands
BECN1	Beclin-1; Plays a central role in autophagy. Acts as core subunit of the PI3K complex that mediates formation of phosphatidylinositol 3-phosphate; different complex forms are believed to play a role in multiple membrane trafficking pathways: PI3KC3-C1 is involved in initiation of autophagosomes and PI3KC3-C2 in maturation of autophagosomes and endocytosis. Involved in regulation of degradative endocytic trafficking and required for the abcission step in cytokinesis, probably in the context of PI3KC3-C2. Essential for the formation of PI3KC3-C2 but not PI3KC3-C1 PI3K complex forms. Invo [...]
PINK1	Serine/threonine-protein kinase PINK1, mitochondrial; Protects against mitochondrial dysfunction during cellular stress by phosphorylating mitochondrial proteins. Involved in the clearance of damaged mitochondria via selective autophagy (mitophagy) by mediating activation and translocation of PRKN. Targets PRKN to dysfunctional depolarized mitochondria through the

	phosphorylation of MFN2. Activates PRKN in 2 steps: (1) by mediating phosphorylation at 'Ser-65' of PRKN and (2) mediating phosphorylation of ubiquitin, converting PRKN to its fully-active form. Required for ubiquinone reduct [...]
CCL2	C-C motif chemokine 2; Chemotactic factor that attracts monocytes and basophils but not neutrophils or eosinophils. Augments monocyte anti-tumor activity. Has been implicated in the pathogenesis of diseases characterized by monocytic infiltrates, like psoriasis, rheumatoid arthritis or atherosclerosis. May be involved in the recruitment of monocytes into the arterial wall during the disease process of atherosclerosis; Belongs to the intercrine beta (chemokine CC) family
SOD2	Superoxide dismutase [Mn], mitochondrial; Destroys superoxide anion radicals which are normally produced within the cells and which are toxic to biological systems
CTNNB1	Catenin beta-1; Key downstream component of the canonical Wnt signaling pathway. In the absence of Wnt, forms a complex with AXIN1, AXIN2, APC, CSNK1A1 and GSK3B that promotes phosphorylation on N-terminal Ser and Thr residues and ubiquitination of CTNNB1 via BTRC and its subsequent degradation by the proteasome. In the presence of Wnt ligand, CTNNB1 is not ubiquitinated and accumulates in the nucleus, where it acts as a coactivator for transcription factors of the TCF/LEF family, leading to activate Wnt responsive genes. Involved in the regulation of cell adhesion, as component of an [...]
NOX4	NADPH oxidase 4; Constitutive NADPH oxidase which generates superoxide intracellularly upon formation of a complex with CYBA/p22phox. Regulates signaling cascades probably through phosphatases inhibition. May function as an oxygen sensor regulating the KCNK3/TASK-1 potassium channel and HIF1A activity. May regulate insulin signaling cascade. May play a role in apoptosis, bone resorption and lipolysaccharide-mediated activation of NFKB. May produce superoxide in the nucleus and play a role in regulating gene expression upon cell stimulation. Isoform 3 is not functional. Isoform 5 and is [...]
CD8A	T-cell surface glycoprotein CD8 alpha chain; Integral membrane glycoprotein that plays an essential role in the immune response and serves multiple functions in responses against both external and internal offenses. In T-cells, functions primarily as a coreceptor for MHC class I molecule:peptide complex. The antigens presented by class I peptides are derived from cytosolic proteins while class II derived from extracellular proteins. Interacts simultaneously with the T-cell receptor (TCR) and the MHC class I proteins presented by antigen presenting cells (APCs). In turn, recruits the Sr [...]
RPS6KB1	Ribosomal protein S6 kinase beta-1; Serine/threonine-protein kinase that acts downstream of mTOR signaling in response to growth factors and nutrients to promote cell proliferation, cell growth and cell cycle progression. Regulates protein synthesis through phosphorylation of EIF4B, RPS6 and EEF2K, and contributes to cell survival by repressing the pro-apoptotic function of BAD. Under conditions of nutrient depletion, the inactive form associates with the EIF3 translation initiation complex. Upon mitogenic stimulation, phosphorylation by the mammalian target of rapamycin complex 1 (mTO [...]
PTGS2	Prostaglandin G/H synthase 2; Converts arachidonate to prostaglandin H2 (PGH2), a committed step in prostanoid synthesis. Constitutively expressed in some tissues in physiological conditions, such as the endothelium, kidney and brain, and in pathological conditions, such as in cancer. PTGS2 is responsible for production of inflammatory prostaglandins. Up-regulation of PTGS2 is also associated with increased cell adhesion, phenotypic changes, resistance to apoptosis and tumor angiogenesis. In cancer cells, PTGS2 is a key step in the production of prostaglandin E2 (PGE2), which plays imp [...]
CASP9	Caspase-9; Involved in the activation cascade of caspases responsible for apoptosis execution. Binding of caspase-9 to Apaf- 1 leads to activation of the protease which then cleaves and activates caspase-3. Promotes DNA damage-induced apoptosis in a ABL1/c-Abl-dependent manner. Proteolytically cleaves poly(ADP- ribose) polymerase (PARP); Apoptosome
DNM1L	Dynamin-1-like protein; Functions in mitochondrial and peroxisomal division. Mediates membrane fission through oligomerization into membrane- associated tubular structures that wrap around the scission site to constrict and sever the mitochondrial membrane through a GTP hydrolysis-dependent mechanism. Through its function in mitochondrial division, ensures the survival of at least some types of postmitotic neurons, including Purkinje cells, by suppressing oxidative damage. Required for normal brain development, including that of cerebellum. Facilitates developmentally regulated apoptos [...]
PTEN	Phosphatase and tensin homolog; Tumor suppressor. Acts as a dual-specificity protein phosphatase, dephosphorylating tyrosine-, serine- and threonine- phosphorylated proteins. Also acts as a lipid phosphatase, removing the phosphate in the D3 position of the inositol ring from phosphatidylinositol 3,4,5-trisphosphate, phosphatidylinositol 3,4-diphosphate, phosphatidylinositol 3- phosphate and inositol 1,3,4,5-tetrakisphosphate with order of substrate preference in vitro PtdIns(3,4,5)P3 > PtdIns(3,4)P2 > PtdIns3P > Ins(1,3,4,5)P4. The lipid phosphatase activity is critical for its tumor [...]
SRC	Proto-oncogene tyrosine-protein kinase Src; Non-receptor protein tyrosine kinase which is activated following engagement of many different classes of cellular receptors including immune response receptors, integrins and other adhesion receptors, receptor protein tyrosine kinases, G protein- coupled receptors as well as cytokine receptors. Participates in signaling pathways that control a diverse spectrum of biological activities including gene transcription, immune response, cell adhesion, cell cycle progression, apoptosis, migration, and transformation. Due to functional redundancy be [...]
CREB1	Cyclic AMP-responsive element-binding protein 1; Phosphorylation-dependent transcription factor that stimulates transcription upon binding to the DNA cAMP response element (CRE), a sequence present in many viral and cellular promoters. Transcription activation is enhanced by the TORC coactivators which act independently of Ser-133 phosphorylation. Involved in different cellular processes including the synchronization of circadian rhythmicity and the differentiation of adipose cells; Basic leucine zipper proteins
SOD1	Superoxide dismutase, cu-zn family; Superoxide dismutase [Cu-Zn]; Destroys radicals which are normally produced within the cells and which are toxic to biological systems
IL4	Interleukin-4; Participates in at least several B-cell activation processes as well as of other cell types. It is a costimulator of DNA-synthesis. It induces the expression of class II MHC molecules on resting B-cells. It enhances both secretion and cell surface expression of IgE and IgG1. It also regulates the expression of the low affinity Fc receptor for IgE (CD23) on both lymphocytes and monocytes. Positively regulates IL31RA expression in macrophages (By similarity); Interleukins
VEGFA	Vascular endothelial growth factor A; Growth factor active in angiogenesis, vasculogenesis and endothelial cell growth. Induces endothelial cell proliferation, promotes cell migration, inhibits apoptosis and induces permeabilization of blood vessels. Binds to the FLT1/VEGFR1 and KDR/VEGFR2 receptors, heparan sulfate and heparin. NRP1/Neuropilin-1 binds isoforms VEGF-165 and VEGF-145. Isoform VEGF165B binds to KDR but does not activate downstream signaling pathways, does not activate angiogenesis and inhibits tumor growth. Binding to NRP1 receptor initiates a signaling pathway needed fo [...]
EGFR	Epidermal growth factor receptor; Receptor tyrosine kinase binding ligands of the EGF family and activating several signaling cascades to convert extracellular cues into appropriate cellular responses. Known ligands include EGF, TGFA/TGF-alpha, amphiregulin, epigen/EPGN, BTC/betacellulin, epiregulin/EREG and HBEGF/heparin-binding EGF. Ligand binding triggers receptor

	homo- and/or heterodimerization and autophosphorylation on key cytoplasmic residues. The phosphorylated receptor recruits adapter proteins like GRB2 which in turn activates complex downstream signaling cascades. Activates [...]
EIF2AK3	Eukaryotic translation initiation factor 2-alpha kinase 3; Metabolic-stress sensing protein kinase that phosphorylates the alpha subunit of eukaryotic translation initiation factor 2 (eIF-2-alpha/EIF2S1) on 'Ser-52' during the unfolded protein response (UPR) and in response to low amino acid availability. Converts phosphorylated eIF-2-alpha/EIF2S1 either in a global protein synthesis inhibitor, leading to a reduced overall utilization of amino acids, or to a translation initiation activator of specific mRNAs, such as the transcriptional activator ATF4, and hence allowing ATF4-mediated [...]
COX4I1	Cytochrome c oxidase subunit 4 isoform 1, mitochondrial; This protein is one of the nuclear-coded polypeptide chains of cytochrome c oxidase, the terminal oxidase in mitochondrial electron transport
EIF2S1	Eukaryotic translation initiation factor 2 subunit 1; Functions in the early steps of protein synthesis by forming a ternary complex with GTP and initiator tRNA. This complex binds to a 40S ribosomal subunit, followed by mRNA binding to form a 43S pre-initiation complex. Junction of the 60S ribosomal subunit to form the 80S initiation complex is preceded by hydrolysis of the GTP bound to eIF-2 and release of an eIF-2- GDP binary complex. In order for eIF-2 to recycle and catalyze another round of initiation, the GDP bound to eIF-2 must exchange with GTP by way of a reaction catalyzed b [...]
MT-CYB	Cytochrome b; Component of the ubiquinol-cytochrome c reductase complex (complex III or cytochrome b-c1 complex) that is part of the mitochondrial respiratory chain. The b-c1 complex mediates electron transfer from ubiquinol to cytochrome c. Contributes to the generation of a proton gradient across the mitochondrial membrane that is then used for ATP synthesis
MAP1LC3B	Microtubule-associated proteins 1A/1B light chain 3B; Ubiquitin-like modifier involved in formation of autophagosomal vacuoles (autophagosomes). Plays a role in mitophagy which contributes to regulate mitochondrial quantity and quality by eliminating the mitochondria to a basal level to fulfill cellular energy requirements and preventing excess ROS production. Whereas LC3s are involved in elongation of the phagophore membrane, the GABARAP/GATE-16 subfamily is essential for a later stage in autophagosome maturation. Promotes primary ciliogenesis by removing OFD1 from centriolar satellit [...]
FOXO3	Forkhead box protein O3; Transcriptional activator which triggers apoptosis in the absence of survival factors, including neuronal cell death upon oxidative stress. Recognizes and binds to the DNA sequence 5'-[AG]TAAA[TC]A-3'. Participates in post-transcriptional regulation of MYC: following phosphorylation by MAPKAPK5, promotes induction of miR-34b and miR-34c expression, 2 post-transcriptional regulators of MYC that bind to the 3'UTR of MYC transcript and prevent its translation; Forkhead boxes
NOS3	Nitric-oxide synthase, endothelial; Nitric oxide synthase, endothelial; Produces nitric oxide (NO) which is implicated in vascular smooth muscle relaxation through a cGMP-mediated signal transduction pathway. NO mediates vascular endothelial growth factor (VEGF)-induced angiogenesis in coronary vessels and promotes blood clotting through the activation of platelets
SDHB	Succinate dehydrogenase [ubiquinone] iron-sulfur subunit, mitochondrial; Iron-sulfur protein (IP) subunit of succinate dehydrogenase (SDH) that is involved in complex II of the mitochondrial electron transport chain and is responsible for transferring electrons from succinate to ubiquinone (coenzyme Q)
BCL2L1	Bcl-2-like protein 1; Potent inhibitor of cell death. Inhibits activation of caspases. Appears to regulate cell death by blocking the voltage- dependent anion channel (VDAC) by binding to it and preventing the release of the caspase activator, CYC1, from the mitochondrial membrane. Also acts as a regulator of G2 checkpoint and progression to cytokinesis during mitosis; Belongs to the Bcl-2 family
FOXO1	Forkhead box protein O1; Transcription factor that is the main target of insulin signaling and regulates metabolic homeostasis in response to oxidative stress. Binds to the insulin response element (IRE) with consensus sequence 5'-TT[G/A]TTTTG-3' and the related Daf-16 family binding element (DBE) with consensus sequence 5'- TT[G/A]TTTAC-3'. Activity suppressed by insulin. Main regulator of redox balance and osteoblast numbers and controls bone mass. Orchestrates the endocrine function of the skeleton in regulating glucose metabolism. Acts synergistically with ATF4 to suppress osteocal [...]
IRS1	Insulin receptor substrate 1; May mediate the control of various cellular processes by insulin. When phosphorylated by the insulin receptor binds specifically to various cellular proteins containing SH2 domains such as phosphatidylinositol 3-kinase p85 subunit or GRB2. Activates phosphatidylinositol 3-kinase when bound to the regulatory p85 subunit (By similarity)
MMP9	Matrix metalloproteinase-9; May play an essential role in local proteolysis of the extracellular matrix and in leukocyte migration. Could play a role in bone osteoclastic resorption. Cleaves KiSS1 at a Gly-|-Leu bond. Cleaves type IV and type V collagen into large C-terminal three quarter fragments and shorter N-terminal one quarter fragments. Degrades fibronectin but not laminin or Pz-peptide; M10 matrix metallopeptidases
NLRP3	NACHT, LRR and PYD domains-containing protein 3; As the sensor component of the NLRP3 inflammasome, plays a crucial role in innate immunity and inflammation. In response to pathogens and other damage-associated signals, initiates the formation of the inflammasome polymeric complex, made of NLRP3, PYCARD and CASP1 (and possibly CASP4 and CASP5). Recruitment of proCASP1 to the inflammasome promotes its activation and CASP1- catalyzed IL1B and IL18 maturation and secretion in the extracellular milieu. Activation of NLRP3 inflammasome is also required for HMGB1 secretion. The active cytoki [...]
H2AFX	H2A histone family member X; Histone H2AX; Variant histone H2A which replaces conventional H2A in a subset of nucleosomes. Nucleosomes wrap and compact DNA into chromatin, limiting DNA accessibility to the cellular machineries which require DNA as a template. Histones thereby play a central role in transcription regulation, DNA repair, DNA replication and chromosomal stability. DNA accessibility is regulated via a complex set of post-translational modifications of histones, also called histone code, and nucleosome remodeling. Required for checkpoint-mediated arrest of cell cycle progre [...]
SQSTM1	Sequestosome-1; Autophagy receptor that interacts directly with both the cargo to become degraded and an autophagy modifier of the MAP1 LC3 family. Along with WDFY3, involved in the formation and autophagic degradation of cytoplasmic ubiquitin-containing inclusions (p62 bodies, ALIS/aggresome-like induced structures). Along with SQSTM1, required to recruit ubiquitinated proteins to PML bodies in the nucleus. May regulate the activation of NFKB1 by TNF-alpha, nerve growth factor (NGF) and interleukin-1. May play a role in titin/TTN downstream signaling in muscle cells. May regulate sign [...]
APOE	Apolipoprotein E; Mediates the binding, internalization, and catabolism of lipoprotein particles. It can serve as a ligand for the LDL (apo B/E) receptor and for the specific apo-E receptor (chylomicron remnant) of hepatic tissues; Apolipoproteins
SLC2A4	Solute carrier family 2, facilitated glucose transporter member 4; Insulin-regulated facilitative glucose transporter; Solute carriers
CS	Citrate synthase, mitochondrial; Citrate synthase
SDHA	Succinate dehydrogenase [ubiquinone] flavoprotein subunit, mitochondrial; Flavoprotein (FP) subunit of succinate dehydrogenase (SDH) that is involved in complex II of the mitochondrial electron transport chain and is responsible for transferring electrons from

	succinate to ubiquinone (coenzyme Q). Can act as a tumor suppressor; Belongs to the FAD-dependent oxidoreductase 2 family. FRD/SDH subfamily
HSPA5	78 kDa glucose-regulated protein; Plays a role in facilitating the assembly of multimeric protein complexes inside the endoplasmic reticulum. Involved in the correct folding of proteins and degradation of misfolded proteins via its interaction with DNAJC10, probably to facilitate the release of DNAJC10 from its substrate (By similarity); Belongs to the heat shock protein 70 family
TXN	Thioredoxin; Participates in various redox reactions through the reversible oxidation of its active center dithiol to a disulfide and catalyzes dithiol-disulfide exchange reactions. Plays a role in the reversible S-nitrosylation of cysteine residues in target proteins, and thereby contributes to the response to intracellular nitric oxide. Nitrosylates the active site Cys of CASP3 in response to nitric oxide (NO), and thereby inhibits caspase-3 activity. Induces the FOS/JUN AP-1 DNA-binding activity in ionizing radiation (IR) cells through its oxidation/reduction status and stimulates A [...]
VDAC1	Voltage-dependent anion-selective channel protein 1; Forms a channel through the mitochondrial outer membrane and also the plasma membrane. The channel at the outer mitochondrial membrane allows diffusion of small hydrophilic molecules; in the plasma membrane it is involved in cell volume regulation and apoptosis. It adopts an open conformation at low or zero membrane potential and a closed conformation at potentials above 30-40 mV. The open state has a weak anion selectivity whereas the closed state is cation-selective. May participate in the formation of the permeability transition p [...]
ESR1	Estrogen receptor; Nuclear hormone receptor. The steroid hormones and their receptors are involved in the regulation of eukaryotic gene expression and affect cellular proliferation and differentiation in target tissues. Ligand-dependent nuclear transactivation involves either direct homodimer binding to a palindromic estrogen response element (ERE) sequence or association with other DNA-binding transcription factors, such as AP-1/c-Jun, c-Fos, ATF-2, Sp1 and Sp3, to mediate ERE-independent signaling. Ligand binding induces a conformational change allowing subsequent or combinatorial a [...]
MPO	Myeloperoxidase; Part of the host defense system of polymorphonuclear leukocytes. It is responsible for microbicidal activity against a wide range of organisms. In the stimulated PMN, MPO catalyzes the production of hypohalous acids, primarily hypochlorous acid in physiologic situations, and other toxic intermediates that greatly enhance PMN microbicidal activity; Belongs to the peroxidase family. XPO subfamily
MT-CO1	Mitochondrially encoded cytochrome c oxidase i; Cytochrome c oxidase subunit 1; Cytochrome c oxidase is the component of the respiratory chain that catalyzes the reduction of oxygen to water. Subunits 1- 3 form the functional core of the enzyme complex. CO I is the catalytic subunit of the enzyme. Electrons originating in cytochrome c are transferred via the copper A center of subunit 2 and heme A of subunit 1 to the bimetallic center formed by heme A3 and copper B
JAK2	Tyrosine-protein kinase JAK2; Non-receptor tyrosine kinase involved in various processes such as cell growth, development, differentiation or histone modifications. Mediates essential signaling events in both innate and adaptive immunity. In the cytoplasm, plays a pivotal role in signal transduction via its association with type I receptors such as growth hormone (GHR), prolactin (PRLR), leptin (LEPR), erythropoietin (EPOR), thrombopoietin (THPO); or type II receptors including IFN-alpha, IFN-beta, IFN-gamma and multiple interleukins. Following ligand-binding to cell surface receptors, [...]
CCND1	G1/S-specific cyclin-D1; Regulatory component of the cyclin D1-CDK4 (DC) complex that phosphorylates and inhibits members of the retinoblastoma (RB) protein family including RB1 and regulates the cell-cycle during G(1)/S transition. Phosphorylation of RB1 allows dissociation of the transcription factor E2F from the RB/E2F complex and the subsequent transcription of E2F target genes which are responsible for the progression through the G(1) phase. Hypophosphorylates RB1 in early G(1) phase. Cyclin D-CDK4 complexes are major integrators of various mitogenenic and antimitogenic signals. A [...]
ATM	Serine-protein kinase ATM; Serine/threonine protein kinase which activates checkpoint signaling upon double strand breaks (DSBs), apoptosis and genotoxic stresses such as ionizing ultraviolet A light (UVA), thereby acting as a DNA damage sensor. Recognizes the substrate consensus sequence [ST]-Q. Phosphorylates 'Ser-139' of histone variant H2AX/H2AFX at double strand breaks (DSBs), thereby regulating DNA damage response mechanism. Also plays a role in pre-B cell allelic exclusion, a process leading to expression of a single immunoglobulin heavy chain allele to enforce clonality and mon [...]
EGF	Pro-epidermal growth factor; EGF stimulates the growth of various epidermal and epithelial tissues in vivo and in vitro and of some fibroblasts in cell culture. Magnesiotropic hormone that stimulates magnesium reabsorption in the renal distal convoluted tubule via engagement of EGFR and activation of the magnesium channel TRPM6. Can induce neurite outgrowth in motoneurons of the pond snail Lymnaea stagnalis in vitro
IL17A	Interleukin-17A; Ligand for IL17RA and IL17RC. The heterodimer formed by IL17A and IL17F is a ligand for the heterodimeric complex formed by IL17RA and IL17RC. Involved in inducing stromal cells to produce proinflammatory and hematopoietic cytokines; Belongs to the IL-17 family
IL2	Interleukin-2; Produced by T-cells in response to antigenic or mitogenic stimulation, this protein is required for T-cell proliferation and other activities crucial to regulation of the immune response. Can stimulate B-cells, monocytes, lymphokine- activated killer cells, natural killer cells, and glioma cells; Interleukins
CD36	Platelet glycoprotein 4; Multifunctional glycoprotein that acts as receptor for a broad range of ligands. Ligands can be of proteinaceous nature like thrombospondin, fibronectin, collagen or amyloid-beta as well as of lipidic nature such as oxidized low-density lipoprotein (oxLDL), anionic phospholipids, long-chain fatty acids and bacterial diacylated lipopeptides. They are generally multivalent and can therefore engage multiple receptors simultaneously, the resulting formation of CD36 clusters initiates signal transduction and internalization of receptor-ligand complexes. The dependen [...]
HK2	Hexokinase-2; Hexokinase 2; Belongs to the hexokinase family
MAPK8	Mitogen-activated protein kinase 8/9/10 (c-jun n-terminal kinase); Mitogen-activated protein kinase 8; Serine/threonine-protein kinase involved in various processes such as cell proliferation, differentiation, migration, transformation and programmed cell death. Extracellular stimuli such as proinflammatory cytokines or physical stress stimulate the stress-activated protein kinase/c-Jun N-terminal kinase (SAP/JNK) signaling pathway. In this cascade, two dual specificity kinases MAP2K4/MKK4 and MAP2K7/MKK7 phosphorylate and activate MAPK8/JNK1. In turn, MAPK8/JNK1 phosphorylates a numbe [...]
SNCA	Alpha-synuclein; May be involved in the regulation of dopamine release and transport. Induces fibrillization of microtubule-associated protein tau. Reduces neuronal responsiveness to various apoptotic stimuli, leading to a decreased caspase-3 activation; Parkinson disease associated genes
TOMM20	Mitochondrial import receptor subunit TOM20 homolog; Central component of the receptor complex responsible for the recognition and translocation of cytosolically synthesized mitochondrial preproteins. Together with TOM22 functions as the transit peptide

	receptor at the surface of the mitochondrion outer membrane and facilitates the movement of preproteins into the TOM40 translocation pore (By similarity); Belongs to the Tom20 family
CASP1	Caspase-1; Thiol protease that cleaves IL-1 beta between an Asp and an Ala, releasing the mature cytokine which is involved in a variety of inflammatory processes. Important for defense against pathogens. Cleaves and activates sterol regulatory element binding proteins (SREBPs). Can also promote apoptosis. Upon inflammasome activation, during DNA virus infection but not RNA virus challenge, controls antiviral immunity through the cleavage of MB21D1/cGAS, rendering it inactive; Belongs to the peptidase C14A family
ICAM1	Intercellular adhesion molecule 1; ICAM proteins are ligands for the leukocyte adhesion protein LFA-1 (integrin alpha-L/beta-2). During leukocyte trans- endothelial migration, ICAM1 engagement promotes the assembly of endothelial apical cups through ARHGEF26/SGEF and RHOG activation; CD molecules
MT-ND1	NADH-ubiquinone oxidoreductase chain 1; Core subunit of the mitochondrial membrane respiratory chain NADH dehydrogenase (Complex I) that is believed to belong to the minimal assembly required for catalysis. Complex I functions in the transfer of electrons from NADH to the respiratory chain. The immediate electron acceptor for the enzyme is believed to be ubiquinone (By similarity); NADH:ubiquinone oxidoreductase core subunits
PTPRC	Receptor-type tyrosine-protein phosphatase C; Protein tyrosine-protein phosphatase required for T-cell activation through the antigen receptor. Acts as a positive regulator of T-cell coactivation upon binding to DPP4. The first PTPase domain has enzymatic activity, while the second one seems to affect the substrate specificity of the first one. Upon T-cell activation, recruits and dephosphorylates SKAP1 and FYN. Dephosphorylates LYN, and thereby modulates LYN activity (By similarity); Belongs to the protein-tyrosine phosphatase family. Receptor class 1/6 subfamily
SCARB1	Scavenger receptor class B member 1; Receptor for different ligands such as phospholipids, cholesterol ester, lipoproteins, phosphatidylserine and apoptotic cells. Receptor for HDL, mediating selective uptake of cholesteryl ether and HDL-dependent cholesterol efflux. Also facilitates the flux of free and esterified cholesterol between the cell surface and apoB-containing lipoproteins and modified lipoproteins, although less efficiently than HDL. May be involved in the phagocytosis of apoptotic cells, via its phosphatidylserine binding activity; Scavenger receptors
UCP2	Mitochondrial uncoupling protein 2; UCP are mitochondrial transporter proteins that create proton leaks across the inner mitochondrial membrane, thus uncoupling oxidative phosphorylation from ATP synthesis. As a result, energy is dissipated in the form of heat; Belongs to the mitochondrial carrier (TC 2.A.29) family
DDIT3	DNA damage-inducible transcript 3 protein; Multifunctional transcription factor in ER stress response. Plays an essential role in the response to a wide variety of cell stresses and induces cell cycle arrest and apoptosis in response to ER stress. Plays a dual role both as an inhibitor of CCAAT/enhancer-binding protein (C/EBP) function and as an activator of other genes. Acts as a dominant-negative regulator of C/EBP-induced transcription: dimerizes with members of the C/EBP family, impairs their association with C/EBP binding sites in the promoter regions, and inhibits the expression [...]
SIRT7	NAD-dependent protein deacetylase sirtuin-7; NAD-dependent protein deacetylase that specifically mediates deacetylation of histone H3 at 'Lys-18' (H3K18Ac). In contrast to other histone deacetylases, displays selectivity for a single histone mark, H3K18Ac, directly linked to control of gene expression. H3K18Ac is mainly present around the transcription start site of genes and has been linked to activation of nuclear hormone receptors. SIRT7 thereby acts as a transcription repressor. Moreover, H3K18 hypoacetylation has been reported as a marker of malignancy in various cancers and seems [...]
SREBF1	Sterol regulatory element-binding protein 1; Transcriptional activator required for lipid homeostasis. Regulates transcription of the LDL receptor gene as well as the fatty acid and to a lesser degree the cholesterol synthesis pathway (By similarity). Binds to the sterol regulatory element 1 (SRE-1) (5'-ATCACCCCAC-3'). Has dual sequence specificity binding to both an E-box motif (5'-ATCACGTGA-3') and to SRE-1 (5'-ATCACCCCAC-3'); Basic helix-loop-helix proteins
MAPT	Microtubule-associated protein tau; Promotes microtubule assembly and stability, and might be involved in the establishment and maintenance of neuronal polarity. The C-terminus binds axonal microtubules while the N- terminus binds neural plasma membrane components, suggesting that tau functions as a linker protein between both. Axonal polarity is predetermined by TAU/MAPT localization (in the neuronal cell) in the domain of the cell body defined by the centrosome. The short isoforms allow plasticity of the cytoskeleton whereas the longer isoforms may preferentially play a role in its s [...]
MFN1	Mitofusin-1; Mitochondrial outer membrane GTPase that mediates mitochondrial clustering and fusion. Membrane clustering requires GTPase activity. It may involve a major rearrangement of the coiled coil domains. Mitochondria are highly dynamic organelles, and their morphology is determined by the equilibrium between mitochondrial fusion and fission events. Overexpression induces the formation of mitochondrial networks (in vitro). Has low GTPase activity
SCARB2	Lysosome membrane protein 2; Acts as a lysosomal receptor for glucosylceramidase (GBA) targeting; Belongs to the CD36 family
SMAD2	Mothers against decapentaplegic homolog 2; Receptor-regulated SMAD (R-SMAD) that is an intracellular signal transducer and transcriptional modulator activated by TGF-beta (transforming growth factor) and activin type 1 receptor kinases. Binds the TRE element in the promoter region of many genes that are regulated by TGF-beta and, on formation of the SMAD2/SMAD4 complex, activates transcription. May act as a tumor suppressor in colorectal carcinoma. Positively regulates PDPK1 kinase activity by stimulating its dissociation from the 14-3-3 protein YWHAQ which acts as a negative regulator
CPT1A	Carnitine O-palmitoyltransferase 1, liver isoform; Catalyzes the transfer of the acyl group of long-chain fatty acid-CoA conjugates onto carnitine, an essential step for the mitochondrial uptake of long-chain fatty acids and their subsequent beta-oxidation in the mitochondrion. Plays an important role in triglyceride metabolism
FIS1	Mitochondrial fission 1 protein; Involved in the fragmentation of the mitochondrial network and its perinuclear clustering. Plays a minor role in the recruitment and association of the fission mediator dynamin- related protein 1 (DNM1L) to the mitochondrial surface and mitochondrial fission. Can induce cytochrome c release from the mitochondrion to the cytosol, ultimately leading to apoptosis. Also mediates peroxisomal fission; Belongs to the FIS1 family
GPX1	Glutathione peroxidase 1; Protects the hemoglobin in erythrocytes from oxidative breakdown; Belongs to the glutathione peroxidase family
FASN	Fatty acid synthase; Fatty acid synthetase catalyzes the formation of long- chain fatty acids from acetyl-CoA, malonyl-CoA and NADPH. This multifunctional protein has 7 catalytic activities and an acyl carrier protein; Seven-beta-strand methyltransferase motif containing
MT-ND2	NADH-ubiquinone oxidoreductase chain 2; Core subunit of the mitochondrial membrane respiratory chain NADH dehydrogenase (Complex I) that is believed to belong to the minimal assembly required for catalysis. Complex I functions in the transfer of electrons

	from NADH to the respiratory chain. The immediate electron acceptor for the enzyme is believed to be ubiquinone (By similarity); NADH:ubiquinone oxidoreductase core subunits
SMAD3	Mothers against decapentaplegic homolog 3; Receptor-regulated SMAD (R-SMAD) that is an intracellular signal transducer and transcriptional modulator activated by TGF-beta (transforming growth factor) and activin type 1 receptor kinases. Binds the TRE element in the promoter region of many genes that are regulated by TGF-beta and, on formation of the SMAD3/SMAD4 complex, activates transcription. Also can form a SMAD3/SMAD4/JUN/FOS complex at the AP-1/SMAD site to regulate TGF-beta-mediated transcription. Has an inhibitory effect on wound healing probably by modulating both growth and mi [...]
PDK4	[Pyruvate dehydrogenase (acetyl-transferring)] kinase isozyme 4, mitochondrial; Kinase that plays a key role in regulation of glucose and fatty acid metabolism and homeostasis via phosphorylation of the pyruvate dehydrogenase subunits PDHA1 and PDHA2. This inhibits pyruvate dehydrogenase activity, and thereby regulates metabolite flux through the tricarboxylic acid cycle, down-regulates aerobic respiration and inhibits the formation of acetyl-coenzyme A from pyruvate. Inhibition of pyruvate dehydrogenase decreases glucose utilization and increases fat metabolism in response to prolonge [...]
UQCRC2	Cytochrome b-c1 complex subunit 2, mitochondrial; This is a component of the ubiquinol-cytochrome c reductase complex (complex III or cytochrome b-c1 complex), which is part of the mitochondrial respiratory chain. The core protein 2 is required for the assembly of the complex; M16 metallopeptidases
MT-ATP6	ATP synthase subunit a; Mitochondrial membrane ATP synthase (F(1)F(0) ATP synthase or Complex V) produces ATP from ADP in the presence of a proton gradient across the membrane which is generated by electron transport complexes of the respiratory chain. F-type ATPases consist of two structural domains, F(1) - containing the extramembraneous catalytic core and F(0) - containing the membrane proton channel, linked together by a central stalk and a peripheral stalk. During catalysis, ATP synthesis in the catalytic domain of F(1) is coupled via a rotary mechanism of the central stalk subuni [...]
PDK1	[Pyruvate dehydrogenase (acetyl-transferring)] kinase isozyme 1, mitochondrial; Kinase that plays a key role in regulation of glucose and fatty acid metabolism and homeostasis via phosphorylation of the pyruvate dehydrogenase subunits PDHA1 and PDHA2. This inhibits pyruvate dehydrogenase activity, and thereby regulates metabolite flux through the tricarboxylic acid cycle, down-regulates aerobic respiration and inhibits the formation of acetyl-coenzyme A from pyruvate. Plays an important role in cellular responses to hypoxia and is important for cell proliferation under hypoxia. Protect [...]
CD44	CD44 antigen; Receptor for hyaluronic acid (HA). Mediates cell-cell and cell-matrix interactions through its affinity for HA, and possibly also through its affinity for other ligands such as osteopontin, collagens, and matrix metalloproteinases (MMPs). Adhesion with HA plays an important role in cell migration, tumor growth and progression. In cancer cells, may play an important role in invadopodia formation. Also involved in lymphocyte activation, recirculation and homing, and in hematopoiesis. Altered expression or dysfunction causes numerous pathogenic phenotypes. Great protein hete [...]
ERBB2	Receptor tyrosine-protein kinase erbB-2; Protein tyrosine kinase that is part of several cell surface receptor complexes, but that apparently needs a coreceptor for ligand binding. Essential component of a neuregulin-receptor complex, although neuregulins do not interact with it alone. GP30 is a potential ligand for this receptor. Regulates outgrowth and stabilization of peripheral microtubules (MTs). Upon ERBB2 activation, the MEMO1-RHOA-DIAPH1 signaling pathway elicits the phosphorylation and thus the inhibition of GSK3B at cell membrane. This prevents the phosphorylation of APC and [...]
GSK3B	Glycogen synthase kinase-3 beta; Constitutively active protein kinase that acts as a negative regulator in the hormonal control of glucose homeostasis, Wnt signaling and regulation of transcription factors and microtubules, by phosphorylating and inactivating glycogen synthase (GYS1 or GYS2), EIF2B, CTNNB1/beta-catenin, APC, AXIN1, DPYSL2/CRMP2, JUN, NFATC1/NFATC, MAPT/TAU and MACF1. Requires primed phosphorylation of the majority of its substrates. In skeletal muscle, contributes to insulin regulation of glycogen synthesis by phosphorylating and inhibiting GYS1 activity and hence glyc [...]
EIF4EBP1	Eukaryotic translation initiation factor 4E-binding protein 1; Repressor of translation initiation that regulates EIF4E activity by preventing its assembly into the eIF4F complex: hypophosphorylated form competes with EIF4G1/EIF4G3 and strongly binds to EIF4E, leading to repress translation. In contrast, hyperphosphorylated form dissociates from EIF4E, allowing interaction between EIF4G1/EIF4G3 and EIF4E, leading to initiation of translation. Mediates the regulation of protein translation by hormones, growth factors and other stimuli that signal through the MAP kinase and mTORC1 pathways
MCL1	Induced myeloid leukemia cell differentiation protein Mcl-1; Involved in the regulation of apoptosis versus cell survival, and in the maintenance of viability but not of proliferation. Mediates its effects by interactions with a number of other regulators of apoptosis. Isoform 1 inhibits apoptosis. Isoform 2 promotes apoptosis; Belongs to the Bcl-2 family
MMP2	Matrix metalloproteinase-2 (gelatinase a); 72 kDa type IV collagenase; Ubiquitinous metalloproteinase that is involved in diverse functions such as remodeling of the vasculature, angiogenesis, tissue repair, tumor invasion, inflammation, and atherosclerotic plaque rupture. As well as degrading extracellular matrix proteins, can also act on several nonmatrix proteins such as big endothelial 1 and beta-type CGRP promoting vasoconstriction. Also cleaves KISS at a Gly-|-Leu bond. Appears to have a role in myocardial cell death pathways. Contributes to myocardial oxidative stress by regulat [...]
MT-ND4	Mitochondrially encoded nadh:ubiquinone oxidoreductase core subunit 4; NADH-ubiquinone oxidoreductase chain 4; Core subunit of the mitochondrial membrane respiratory chain NADH dehydrogenase (Complex I) that is believed to belong to the minimal assembly required for catalysis. Complex I functions in the transfer of electrons from NADH to the respiratory chain. The immediate electron acceptor for the enzyme is believed to be ubiquinone (By similarity)
IL18	Interleukin-18; Augments natural killer cell activity in spleen cells and stimulates interferon gamma production in T-helper type I cells; Belongs to the IL-1 family
PARK2	E3 ubiquitin-protein ligase parkin; Functions within a multiprotein E3 ubiquitin ligase complex, catalyzing the covalent attachment of ubiquitin moieties onto substrate proteins, such as BCL2, SYT11, CCNE1, GPR37, RHOT1/MIRO1, MFN1, MFN2, STUB1, SNCAIP, SEPT5, TOMM20, USP30, ZNF746 and AIMP2. Mediates monoubiquitination as well as 'Lys-6', 'Lys-11', 'Lys-48'-linked and 'Lys-63'-linked polyubiquitination of substrates depending on the context. Participates in the removal and/or detoxification of abnormally folded or damaged protein by mediating 'Lys-63'-linked polyubiquitination of misf [...]
TGFB1	Transforming growth factor beta-1; Multifunctional protein that controls proliferation, differentiation and other functions in many cell types. Many cells synthesize TGFB1 and have specific receptors for it. It positively and negatively regulates many other growth factors. It plays an important role in bone remodeling as it is a potent stimulator of osteoblastic bone formation, causing chemotaxis, proliferation and differentiation in committed osteoblasts (By similarity). Stimulates sustained production of collagen through the activation of CREB3L1 by regulated intramembrane proteolysi [...]

XBP1	X-box-binding protein 1; Functions as a transcription factor during endoplasmic reticulum (ER) stress by regulating the unfolded protein response (UPR). Required for cardiac myogenesis and hepatogenesis during embryonic development, and the development of secretory tissues such as exocrine pancreas and salivary gland (By similarity). Involved in terminal differentiation of B lymphocytes to plasma cells and production of immunoglobulins. Modulates the cellular response to ER stress in a PIK3R-dependent manner. Binds to the cis-acting X box present in the promoter regions of major histoc [...]
CDKN2A	Cyclin-dependent kinase inhibitor 2A; Acts as a negative regulator of the proliferation of normal cells by interacting strongly with CDK4 and CDK6. This inhibits their ability to interact with cyclins D and to phosphorylate the retinoblastoma protein
FOXP3	Forkhead box protein P3; Transcriptional regulator which is crucial for the development and inhibitory function of regulatory T-cells (Treg). Plays an essential role in maintaining homeostasis of the immune system by allowing the acquisition of full suppressive function and stability of the Treg lineage, and by directly modulating the expansion and function of conventional T-cells. Can act either as a transcriptional repressor or a transcriptional activator depending on its interactions with other transcription factors, histone acetylases and deacetylases. The suppressive activity of T [...]
HPGDS	Hematopoietic prostaglandin D synthase; Bifunctional enzyme which catalyzes both the conversion of PGH2 to PGD2, a prostaglandin involved in smooth muscle contraction/relaxation and a potent inhibitor of platelet aggregation, and the conjugation of glutathione with a wide range of aryl halides and organic isothiocyanates. Also exhibits low glutathione-peroxidase activity towards cumene hydroperoxide; Belongs to the GST superfamily. Sigma family
NANOG	Homeobox protein NANOG; Transcription regulator involved in inner cell mass and embryonic stem (ES) cells proliferation and self-renewal. Imposes pluripotency on ES cells and prevents their differentiation towards extraembryonic endoderm and trophectoderm lineages. Blocks bone morphogenetic protein-induced mesoderm differentiation of ES cells by physically interacting with SMAD1 and interfering with the recruitment of coactivators to the active SMAD transcriptional complexes. Acts as a transcriptional activator or repressor. Binds optimally to the DNA consensus sequence 5'-TAAT[GT][GT] [...]
NOX1	NADPH oxidase 1; NOH-1S is a voltage-gated proton channel that mediates the H(+) currents of resting phagocytes and other tissues. It participates in the regulation of cellular pH and is blocked by zinc. NOH-1L is a pyridine nucleotide-dependent oxidoreductase that generates superoxide and might conduct H(+) ions as part of its electron transport mechanism, whereas NOH-1S does not contain an electron transport chain
BNIP3	BCL2/adenovirus E1B 19 kDa protein-interacting protein 3; Apoptosis-inducing protein that can overcome BCL2 suppression. May play a role in repartitioning calcium between the two major intracellular calcium stores in association with BCL2. Involved in mitochondrial quality control via its interaction with SPATA18/MIEAP: in response to mitochondrial damage, participates in mitochondrial protein catabolic process (also named MALM) leading to the degradation of damaged proteins inside mitochondria. The physical interaction of SPATA18/MIEAP, BNIP3 and BNIP3L/NIX at the mitochondrial outer [...]
CASP8	Caspase-8; Most upstream protease of the activation cascade of caspases responsible for the TNFRSF6/FAS mediated and TNFRSF1A induced cell death. Binding to the adapter molecule FADD recruits it to either receptor. The resulting aggregate called death-inducing signaling complex (DISC) performs CASP8 proteolytic activation. The active dimeric enzyme is then liberated from the DISC and free to activate downstream apoptotic proteases. Proteolytic fragments of the N-terminal propeptide (termed CAP3, CAP5 and CAP6) are likely retained in the DISC. Cleaves and activates CASP3, CASP4, CASP6, [...]
PARP1	Poly [ADP-ribose] polymerase 1; Involved in the base excision repair (BER) pathway, by catalyzing the poly(ADP-ribosyl)ation of a limited number of acceptor proteins involved in chromatin architecture and in DNA metabolism. This modification follows DNA damages and appears as an obligatory step in a detection/signaling pathway leading to the reparation of DNA strand breaks. Mediates the poly(ADP-ribosyl)ation of APLF and CHFR. Positively regulates the transcription of MTUS1 and negatively regulates the transcription of MTUS2/TIP150. With EEF1A1 and TXK, forms a complex that acts as a T [...]
POU5F1	POU domain, class 5, transcription factor 1; Transcription factor that binds to the octamer motif (5'-ATTTGCAT-3'). Forms a trimeric complex with SOX2 on DNA and controls the expression of a number of genes involved in embryonic development such as YES1, FGF4, UTF1 and ZFP206. Critical for early embryogenesis and for embryonic stem cell pluripotency
NOTCH1	Neurogenic locus notch homolog protein 1; Functions as a receptor for membrane-bound ligands Jagged1, Jagged2 and Delta1 to regulate cell-fate determination. Upon ligand activation through the released notch intracellular domain (NICD) it forms a transcriptional activator complex with RBPJ/RBPSUH and activates genes of the enhancer of split locus. Affects the implementation of differentiation, proliferation and apoptotic programs. Involved in angiogenesis; negatively regulates endothelial cell proliferation and migration and angiogenic sprouting. Involved in the maturation of both CD4+ [...]
ERN1	Serine/threonine-protein kinase/endoribonuclease IRE1; Senses unfolded proteins in the lumen of the endoplasmic reticulum via its N-terminal domain which leads to enzyme auto- activation. The active endoribonuclease domain splices XBP1 mRNA to generate a new C-terminus, converting it into a potent unfolded-protein response transcriptional activator and triggering growth arrest and apoptosis
FGF21	Fibroblast growth factor 21; Stimulates glucose uptake in differentiated adipocytes via the induction of glucose transporter SLC2A1/GLUT1 expression (but not SLC2A4/GLUT4 expression). Activity requires the presence of KLB; Belongs to the heparin-binding growth factors family
IL13	Interleukin-13; Cytokine. Inhibits inflammatory cytokine production. Synergizes with IL2 in regulating interferon-gamma synthesis. May be critical in regulating inflammatory and immune responses. Positively regulates IL31RA expression in macrophages (By similarity); Interleukins
STAT1	Signal transducer and activator of transcription 1-alpha/beta; Signal transducer and transcription activator that mediates cellular responses to interferons (IFNs), cytokine KITLG/SCF and other cytokines and other growth factors. Following type I IFN (IFN-alpha and IFN-beta) binding to cell surface receptors, signaling via protein kinases leads to activation of Jak kinases (TYK2 and JAK1) and to tyrosine phosphorylation of STAT1 and STAT2. The phosphorylated STATs dimerize and associate with ISGF3G/IRF-9 to form a complex termed ISGF3 transcription factor, that enters the nucleus. ISGF [...]
ACLY	ATP-citrate synthase; ATP-citrate synthase is the primary enzyme responsible for the synthesis of cytosolic acetyl-CoA in many tissues. Has a central role in de novo lipid synthesis. In nervous tissue it may be involved in the biosynthesis of acetylcholine; In the N-terminal section; belongs to the succinate/malate CoA ligase beta subunit family
CD34	Hematopoietic progenitor cell antigen CD34; Possible adhesion molecule with a role in early hematopoiesis by mediating the attachment of stem cells to the bone marrow extracellular matrix or directly to stromal cells. Could act as a scaffold for the attachment of lineage specific glycans, allowing stem cells to bind to lectins expressed by stromal cells or other marrow components. Presents carbohydrate ligands to selectins; CD molecules

MT-CO2	Mitochondrially encoded cytochrome c oxidase ii; Cytochrome c oxidase subunit 2; Cytochrome c oxidase is the component of the respiratory chain that catalyzes the reduction of oxygen to water. Subunits 1- 3 form the functional core of the enzyme complex. Subunit 2 transfers the electrons from cytochrome c via its binuclear copper A center to the bimetallic center of the catalytic subunit 1
DNTT	DNA nucleotidylexotransferase; Template-independent DNA polymerase which catalyzes the random addition of deoxynucleoside 5'-triphosphate to the 3'-end of a DNA initiator. One of the in vivo functions of this enzyme is the addition of nucleotides at the junction (N region) of rearranged Ig heavy chain and T-cell receptor gene segments during the maturation of B- and T-cells
HSP90AA1	Heat shock protein HSP 90-alpha; Molecular chaperone that promotes the maturation, structural maintenance and proper regulation of specific target proteins involved for instance in cell cycle control and signal transduction. Undergoes a functional cycle that is linked to its ATPase activity which is essential for its chaperone activity. This cycle probably induces conformational changes in the client proteins, thereby causing their activation. Interacts dynamically with various co-chaperones that modulate its substrate recognition, ATPase cycle and chaperone function. Engages with a ra [...]
OPA1	Dynamin-like 120 kDa protein, mitochondrial; Dynamin-related GTPase that is essential for normal mitochondrial morphology by regulating the equilibrium between mitochondrial fusion and mitochondrial fission. Coexpression of isoform 1 with shorter alternative products is required for optimal activity in promoting mitochondrial fusion. Binds lipid membranes enriched in negatively charged phospholipids, such as cardiolipin, and promotes membrane tubulation. The intrinsic GTPase activity is low, and is strongly increased by interaction with lipid membranes. Plays a role in remodeling crist [...]
SLC16A3	Mfs transporter, mct family, solute carrier family 16 (monocarboxylic acid transporters), member 3; Monocarboxylate transporter 4; Proton-linked monocarboxylate transporter. Catalyzes the rapid transport across the plasma membrane of many monocarboxylates such as lactate, pyruvate, branched-chain oxo acids derived from leucine, valine and isoleucine, and the ketone bodies acetoacetate, beta-hydroxybutyrate and acetate (By similarity); Belongs to the major facilitator superfamily. Monocarboxylate porter (TC 2.A.1.13) family
SLC16A4	Mfs transporter, mct family, solute carrier family 16 (monocarboxylic acid transporters), member 4; Monocarboxylate transporter 5; Proton-linked monocarboxylate transporter. Catalyzes the rapid transport across the plasma membrane of many monocarboxylates such as lactate, pyruvate, branched-chain oxo acids derived from leucine, valine and isoleucine, and the ketone bodies acetoacetate, beta-hydroxybutyrate and acetate (By similarity); Solute carriers
SOX2	Transcription factor SOX-2; Transcription factor that forms a trimeric complex with OCT4 on DNA and controls the expression of a number of genes involved in embryonic development such as YES1, FGF4, UTF1 and ZFP206 (By similarity). Critical for early embryogenesis and for embryonic stem cell pluripotency. May function as a switch in neuronal development. Downstream SRRT target that mediates the promotion of neural stem cell self-renewal (By similarity). Keeps neural cells undifferentiated by counteracting the activity of proneural proteins and suppresses neuronal differentiation (By si [...]
BACE1	Beta-site app-cleaving enzyme 1 (memapsin 2); Beta-secretase 1; Responsible for the proteolytic processing of the amyloid precursor protein (APP). Cleaves at the N-terminus of the A-beta peptide sequence, between residues 671 and 672 of APP, leads to the generation and extracellular release of beta-cleaved soluble APP, and a corresponding cell-associated C-terminal fragment which is later released by gamma-secretase; Belongs to the peptidase A1 family
ESRRA	Steroid hormone receptor ERR1; Binds to an ERR-alpha response element (ERRE) containing a single consensus half-site, 5'-TNAAGGTCA-3'. Can bind to the medium-chain acyl coenzyme A dehydrogenase (MCAD) response element NRRE-1 and may act as an important regulator of MCAD promoter. Binds to the C1 region of the lactoferrin gene promoter. Requires dimerization and the coactivator, PGC-1A, for full activity. The ERRalpha/PGC1alpha complex is a regulator of energy metabolism. Induces the expression of PERM1 in the skeletal muscle; Nuclear hormone receptors
GSR	Glutathione reductase, mitochondrial; Maintains high levels of reduced glutathione in the cytosol
MYH6	Myosin heavy chain 6/7; Myosin-6; Muscle contraction; Belongs to the TRAFAC class myosin-kinesin ATPase superfamily. Myosin family
SNAI1	Zinc finger protein SNAI1; Involved in induction of the epithelial to mesenchymal transition (EMT), formation and maintenance of embryonic mesoderm, growth arrest, survival and cell migration. Binds to 3 E-boxes of the E-cadherin/CDH1 gene promoter and to the promoters of CLDN7 and KRT8 and, in association with histone demethylase KDM1A which it recruits to the promoters, causes a decrease in dimethylated H3K4 levels and represses transcription. Associates with EGR1 and SP1 to mediate tetradecanoyl phorbol acetate (TPA)-induced up- regulation of CDKN2B, possibly by binding to the CDKN2 [...]
ATG5	Autophagy protein 5; Involved in autophagic vesicle formation. Conjugation with ATG12, through a ubiquitin-like conjugating system involving ATG7 as an E1-like activating enzyme and ATG10 as an E2-like conjugating enzyme, is essential for its function. The ATG12-ATG5 conjugate acts as an E3-like enzyme which is required for lipidation of ATG8 family proteins and their association to the vesicle membranes. Involved in mitochondrial quality control after oxidative damage, and in subsequent cellular longevity. Plays a critical role in multiple aspects of lymphocyte development and is esse [...]
CSF2	Granulocyte-macrophage colony-stimulating factor; Cytokine that stimulates the growth and differentiation of hematopoietic precursor cells from various lineages, including granulocytes, macrophages, eosinophils and erythrocytes; Belongs to the GM-CSF family
GCLC	Glutamate--cysteine ligase catalytic subunit; Glutamate-cysteine ligase catalytic subunit
CTLA4	Cytotoxic T-lymphocyte protein 4; Inhibitory receptor acting as a major negative regulator of T-cell responses. The affinity of CTLA4 for its natural B7 family ligands, CD80 and CD86, is considerably stronger than the affinity of their cognate stimulatory coreceptor CD28
KIT	Mast/stem cell growth factor receptor Kit; Tyrosine-protein kinase that acts as cell-surface receptor for the cytokine KITLG/SCF and plays an essential role in the regulation of cell survival and proliferation, hematopoiesis, stem cell maintenance, gametogenesis, mast cell development, migration and function, and in melanogenesis. In response to KITLG/SCF binding, KIT can activate several signaling pathways. Phosphorylates PIK3R1, PLCG1, SH2B2/APS and CBL. Activates the AKT1 signaling pathway by phosphorylation of PIK3R1, the regulatory subunit of phosphatidylinositol 3-kinase. Activat [...]
PPIF	Peptidyl-prolyl cis-trans isomerase F, mitochondrial; PPIases accelerate the folding of proteins. It catalyzes the cis-trans isomerization of proline imidic peptide bonds in oligopeptides. Involved in regulation of the mitochondrial permeability transition pore (mPTP). It is proposed that its association with the mPTP is masking a binding site for inhibiting inorganic phosphate (Pi) and promotes the open

	probability of the mPTP leading to apoptosis or necrosis; the requirement of the PPIase activity for this function is debated. In cooperation with mitochondrial TP53 is involved in act [...]
TXNIP	Thioredoxin-interacting protein; May act as an oxidative stress mediator by inhibiting thioredoxin activity or by limiting its bioavailability. Interacts with COPS5 and restores COPS5-induced suppression of CDKN1B stability, blocking the COPS5-mediated translocation of CDKN1B from the nucleus to the cytoplasm. Functions as a transcriptional repressor, possibly by acting as a bridge molecule between transcription factors and corepressor complexes, and over- expression will induce G0/G1 cell cycle arrest. Required for the maturation of natural killer cells. Acts as a suppressor of tumor [...]
FGF2	Fibroblast growth factor 2; Plays an important role in the regulation of cell survival, cell division, angiogenesis, cell differentiation and cell migration. Functions as potent mitogen in vitro. Can induce angiogenesis; Belongs to the heparin-binding growth factors family
MT-ND6	NADH-ubiquinone oxidoreductase chain 6; Core subunit of the mitochondrial membrane respiratory chain NADH dehydrogenase (Complex I) that is believed to belong to the minimal assembly required for catalysis. Complex I functions in the transfer of electrons from NADH to the respiratory chain. The immediate electron acceptor for the enzyme is believed to be ubiquinone (By similarity); NADH:ubiquinone oxidoreductase core subunits
TJP1	Tight junction protein ZO-1; The N-terminal may be involved in transducing a signal required for tight junction assembly, while the C-terminal may have specific properties of tight junctions. The alpha domain might be involved in stabilizing junctions. Plays a role in the regulation of cell migration by targeting CDC42BPB to the leading edge of migrating cells; Belongs to the MAGUK family
GADD45GIP1	Growth arrest and DNA damage-inducible proteins-interacting protein 1; Acts as a negative regulator of G1 to S cell cycle phase progression by inhibiting cyclin-dependent kinases. Inhibitory effects are additive with GADD45 proteins but occurs also in the absence of GADD45 proteins. Acts as a repressor of the orphan nuclear receptor NR4A1 by inhibiting AB domain-mediated transcriptional activity. May be involved in the hormone-mediated regulation of NR4A1 transcriptional activity. May play a role in mitochondrial protein synthesis
NDUFS6	NADH dehydrogenase [ubiquinone] iron-sulfur protein 6, mitochondrial; Accessory subunit of the mitochondrial membrane respiratory chain NADH dehydrogenase (Complex I), that is believed not to be involved in catalysis. Complex I functions in the transfer of electrons from NADH to the respiratory chain. The immediate electron acceptor for the enzyme is believed to be ubiquinone
MTERF4	Transcription termination factor 4, mitochondrial; Regulator of mitochondrial ribosome biogenesis and translation. Binds to mitochondrial ribosomal RNAs 16S, 12S and 7S and targets NSUN4 RNA methyltransferase to the mitochondrial large ribosomal subunit (39S); Belongs to the mTERF family
NDUFB7	NADH dehydrogenase [ubiquinone] 1 beta subcomplex subunit 7; Accessory subunit of the mitochondrial membrane respiratory chain NADH dehydrogenase (Complex I), that is believed not to be involved in catalysis. Complex I functions in the transfer of electrons from NADH to the respiratory chain. The immediate electron acceptor for the enzyme is believed to be ubiquinone
FTSJ2	rRNA methyltransferase 2, mitochondrial; S-adenosyl-L-methionine-dependent 2'-O-ribose methyltransferase that catalyzes the formation of 2'-O- methyluridine at position 1369 (Um1369) in the 16S mitochondrial large subunit ribosomal RNA (mtLSU rRNA), a universally conserved modification in the peptidyl transferase domain of the mtLSU rRNA; Belongs to the class I-like SAM-binding methyltransferase superfamily. RNA methyltransferase RlmE family
MTG2	Mitochondrial ribosome-associated GTPase 2; Plays a role in the regulation of the mitochondrial ribosome assembly and of translational activity. Displays GTPase activity. Involved in the ribosome maturation process
NSUN4	5-methylcytosine rRNA methyltransferase NSUN4; Involved in mitochondrial ribosome assembly. 5- methylcytosine rRNA methyltransferase that probably is involved in mitochondrial ribosome small subunit (SSU) maturation by methylation of mitochondrial 12S rRNA; the function is independent of MTERFD2/MTERF4 and assembled mitochondrial ribosome large subunit (LSU). Targeted to LSU by MTERFD2/MTERF4 and probably is involved in a final step in ribosome biogenesis to ensure that SSU and LSU are assembled. In vitro can methylate 16S rRNA of the LSU; the methylation is enhanced by MTERFD/MTERF4; [...]
GTPBP10	GTP-binding protein 10; May be involved in the ribosome maturation process. Complements an ObgE(CgtA) function in E.coli ribosome maturation. Plays a role of GTPase in vitro. When missing, disorganization of the nucleolar architecture is observed; Belongs to the TRAFAC class OBG-HflX-like GTPase superfamily. OBG GTPase family
MALSU1	Mitochondrial assembly of ribosomal large subunit protein 1; May function as a ribosomal silencing factor. Addition to isolated mitochondrial ribosomal subunits partially inhibits translation. Interacts with mitochondrial ribosomal protein L14 (MRPL14), probably blocking formation of intersubunit bridge B8, preventing association of the 28S and 39S ribosomal subunits and the formation of functional ribosomes, thus repressing translation. May also participate in the assembly and/or regulation of the stability of the large subunit of the mitochondrial ribosome; Belongs to the Iojap/RsfS family
TIMMDC1	Translocase of inner mitochondrial membrane domain containing 1; Complex I assembly factor TIMMDC1, mitochondrial; Chaperone protein involved in the assembly of the mitochondrial NADH:ubiquinone oxidoreductase complex (complex I). Participates in constructing the membrane arm of complex I
C12orf73	Uncharacterized protein C12orf73; Chromosome 12 open reading frame 73
FASTKD5	FAST kinase domain-containing protein 5, mitochondrial; Plays an important role in the processing of non- canonical mitochondrial mRNA precursors; FASTK mitochondrial RNA binding family
MRPL44	39S ribosomal protein L44, mitochondrial; Component of the 39S subunit of mitochondrial ribosome. May have a function in the assembly/stability of nascent mitochondrial polypeptides exiting the ribosome; Mitochondrial ribosomal proteins
CHCHD1	Coiled-coil-helix-coiled-coil-helix domain-containing protein 1; Mitochondrial coiled-coil-helix-coiled-coil-helix domain containing proteins
COA1	Cytochrome c oxidase assembly factor 1 homolog; Component of the MITRAC (mitochondrial translation regulation assembly intermediate of cytochrome c oxidase complex) complex, that regulates cytochrome c oxidase assembly. MITRAC complexes regulate both translation of mitochondrial encoded components and assembly of nuclear-encoded components imported in mitochondrion. Required for assembly of mitochondrial respiratory chain complex I and complex IV
COQ10A	Coenzyme Q-binding protein COQ10 homolog A, mitochondrial; Required for the function of coenzyme Q in the respiratory chain. May serve as a chaperone or may be involved in the transport of Q6 from its site of synthesis to the catalytic sites of the respiratory complexes (Probable); Belongs to the COQ10 family
HSDL1	Inactive hydroxysteroid dehydrogenase-like protein 1; Short chain dehydrogenase/reductase superfamily; Belongs to the short-chain dehydrogenases/reductases (SDR) family. 17-beta-HSD 3 subfamily

MRPS36	28S ribosomal protein S36, mitochondrial; Mitochondrial ribosomal protein S36
PRSS37	Probable inactive serine protease 37; Plays a role in male fertility (By similarity). May have a role in sperm migration or binding to zona-intact eggs (By similarity). Involved in the activation of the proacrosin/acrosin system; Belongs to the peptidase S1 family
SLC25A51	Solute carrier family 25 member 51
TMEM186	Transmembrane protein 186
TMEM223	Transmembrane protein 223
ATP5L2	ATP synthase subunit g 2, mitochondrial; Mitochondrial membrane ATP synthase (F(1)F(0) ATP synthase or Complex V) produces ATP from ADP in the presence of a proton gradient across the membrane which is generated by electron transport complexes of the respiratory chain. F-type ATPases consist of two structural domains, F(1) - containing the extramembraneous catalytic core, and F(0) - containing the membrane proton channel, linked together by a central stalk and a peripheral stalk. During catalysis, ATP synthesis in the catalytic domain of F(1) is coupled via a rotary mechanism of the ce [...]
C4orf48	Neuropeptide-like protein C4orf48; Chromosome 4 open reading frame 48
CCDC51	Coiled-coil domain-containing protein 51; Mitochondrial potassium channel located in the mitochondrial inner membrane . Together with ABCB8/MITOSUR, forms a protein complex localized in the mitochondria that mediates ATP- dependent potassium currents across the inner membrane (that is, mitoK(ATP) channel) . May contribute to the homeostatic control of cellular metabolism under stress conditions by regulating the mitochondrial matrix volume
METTL12	Methyltransferase-like protein 12, mitochondrial; Protein-lysine methyltransferase that selectively trimethylates citrate synthase (CS) in mitochondria Seems to conduct trimethylation in a highly distributive manner rather than in a processive manner, and thus introduces a single methly group per binding event
DALRD3	DALR anticodon-binding domain-containing protein 3; DALR anticodon binding domain containing 3
DCAF10	DDB1- and CUL4-associated factor 10; May function as a substrate receptor for CUL4-DDB1 E3 ubiquitin-protein ligase complex; DDB1 and CUL4 associated factors
PRR25	Proline-rich protein 25; Proline rich 25
GTPBP8	GTP binding protein 8; Belongs to the TRAFAC class TrmE-Era-EngA-EngB-Septin- like GTPase superfamily. EngB GTPase family
HPDL	4-hydroxyphenylpyruvate dioxygenase-like protein; May have dioxygenase activity
L3HYPDH	Trans-3-hydroxy-L-proline dehydratase; Catalyzes the dehydration of trans-3-hydroxy-L-proline to Delta(1)-pyrroline-2-carboxylate (Pyr2C). May be required to degrade trans-3-hydroxy-L-proline from the diet and originating from the degradation of proteins such as collagen-IV that contain it
LDLRAD1	Low-density lipoprotein receptor class a domain-containing protein 1; Low density lipoprotein receptor class A domain containing 1
MANBAL	Protein MANBAL; Mannosidases type beta; Belongs to the UPF0239 family
METTL17	Methyltransferase-like protein 17, mitochondrial; May be a component of the mitochondrial small ribosomal subunit; Belongs to the methyltransferase superfamily. Rsm22 family
MROH7	Maestro heat-like repeat-containing protein family member 7; Maestro heat like repeat family member 7
MS4A15	Membrane-spanning 4-domains subfamily A member 15; May be involved in signal transduction as a component of a multimeric receptor complex; Membrane spanning 4-domains
C6orf203	Mitochondrial transcription rescue factor 1; Uncharacterized protein C6orf203; Chromosome 6 open reading frame 203
MTRNR2L8	MT-RNR2 like 8; Plays a role as a neuroprotective and antiapoptotic factor; Belongs to the humanin family
OR1F1	Olfactory receptor 1F1; Odorant receptor; Olfactory receptors, family 1
PHGR1	Proline, histidine and glycine-rich protein 1; Proline, histidine and glycine rich 1
PUSL1	Pseudouridylate synthase-like 1; Belongs to the tRNA pseudouridine synthase TruA family
RPUSD2	Rna pseudouridylate synthase domain-containing protein 2; RNA pseudouridylate synthase domain containing 2
SMCO1	Single-pass membrane and coiled-coil domain-containing protein 1; Single-pass membrane protein with coiled-coil domains 1
SMIM4	Small integral membrane protein 4
C9orf9	Sperm acrosome-associated protein 9; Chromosome 9 open reading frame 9
ST20-MTHFS	5-formyltetrahydrofolate cyclo-ligase; ST20-MTHFS readthrough; Belongs to the 5-formyltetrahydrofolate cyclo-ligase family
TMEM177	Transmembrane protein 177; Plays a role in the early steps of cytochrome c oxidase subunit II (MT-CO2/COX2) maturation and is required for the stabilization of COX20 and the newly synthesized MT-CO2/COX2 protein
UQCRHL	Ubiquinol-cytochrome c reductase hinge protein like; May be a component of the ubiquinol-cytochrome c reductase complex (complex III or cytochrome b-c1 complex), which is part of the mitochondrial respiratory chain. This protein may mediate formation of the complex between cytochromes c and c1
ZNF319	Zinc finger protein 319; May be involved in transcriptional regulation; Zinc fingers C2H2-type
hsa-miR-3928-5p	Homo sapiens miR-3928 stem-loop
hsa-miR-4499	Homo sapiens miR-4499 stem-loop
hsa-miR-548e-5p	hsa-miR-548e-5p
hsa-miR-6510-5p	Homo sapiens miR-6510 stem-loop

hsa_circ_000826	Hom sapiens hsa_circ_000826
hsa_circ_000871	Homo sapiens hsa_circ_000871
hsa_circ_002140	Homo sapiens hsa_circ_002140
